# Synthesis of the 6-Substituted Imidazo[1,2-a]Pyridine-3-yl-2- Phosphonopropionic Acids as Potential Inhibitors of Rab Geranylgeranyl Transferase

**DOI:** 10.3389/fchem.2020.596162

**Published:** 2021-01-06

**Authors:** Damian Kusy, Aleksandra Marchwicka, Joanna Małolepsza, Katarzyna Justyna, Edyta Gendaszewska-Darmach, Katarzyna Magdalena Błażewska

**Affiliations:** ^1^Faculty of Chemistry, Institute of Organic Chemistry, Lodz University of Technology, Łódź, Poland; ^2^Faculty of Biotechnology and Food Sciences, Institute of Molecular and Industrial Biotechnology, Lodz University of Technology, Łódź, Poland

**Keywords:** phosphonocarboxylate, phosphonocarboxylic acid, phosphonopropionate, phosphonopropionic acid, RGGT, Rab geranylgeranyl transferase, prenylation, imidazo[1, 2-a]pyridine

## Abstract

Twelve phosphonopropionates derived from 2-hydroxy-3-imidazo[1,2-*a*]pyridin-3-*yl*-2-phosphonopropionic acid (3-IPEHPC) were synthesized and evaluated for their activity as inhibitors of protein geranylgeranylation. The nature of the substituent in the C6 position of imidazo[1,2-*a*]pyridine ring was responsible for the compound's activity against Rab geranylgeranyl transferase (RGGT). The most active inhibitors disrupted Rab11A prenylation in the human cervical carcinoma HeLa cell line. The esterification of carboxylic acid in the phosphonopropionate moiety turned the inhibitor into an inactive analog.

**Graphical Abstract:**
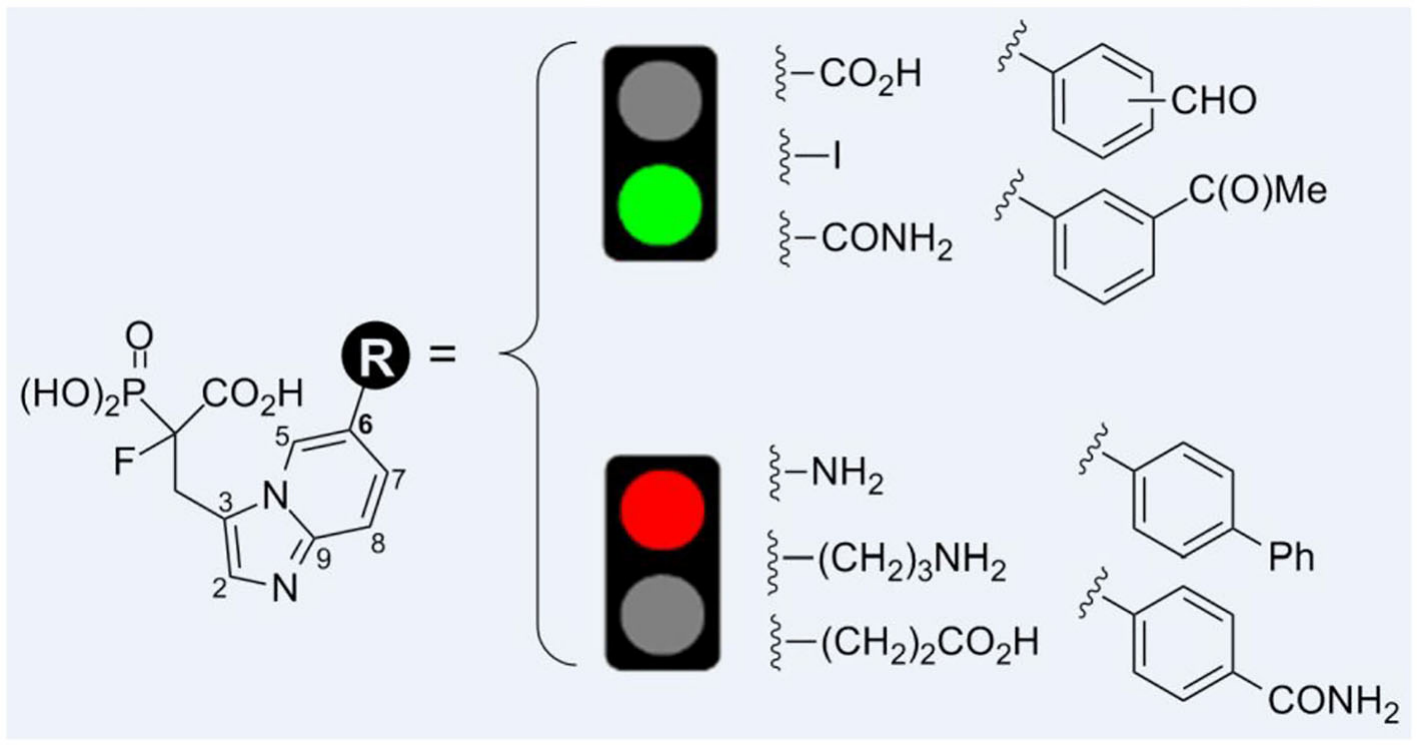


## Introduction

Rab geranylgeranyl transferase (RGGT) constitutes a potential therapeutic target, as it is responsible for posttranslational prenylation of Rab GTPases (Kelly et al., 2012). The perturbed balance of Rabs' modification is observed in several diseases (Mitra et al., 2011; Banworth and Li, 2018), due to their involvement in a wide variety of critical cell processes connected with membrane and vesicle trafficking. The RGGT transfers a geranylgeranyl moiety derived from the mevalonate pathway, which is targeted by two blockbuster classes of drugs: statins and bisphosphonates (Istvan and Deisenhofer, 2001; Russell, 2011). Therefore, the inhibitors of RGGT, as the more downstream enzyme, are gaining on importance as invaluable tools necessary for in-depth studies of molecular bases of these validated drugs' activity (Ali et al., 2015; Jeong et al., 2018).

Currently, a small number of RGGT inhibitors have been identified (Watanabe et al., 2008; Tan et al., 2009; McKenna et al., 2010; Bon et al., 2011; Deraeve et al., 2012; Stigter et al., 2012; Kazmierczak et al., 2017). Among them, only phosphonocarboxylic acids bearing heterocyclic motif were demonstrated to prevent the introduction of the second geranylgeranyl group into Rab GTPases (Baron et al., 2009). Two first representatives of this class, 3-PEHPC and 3-IPEHPC, were identified as a mixed-type inhibitor with respect to GGPP and an uncompetitive inhibitor to Rab substrates (Rab11A was tested in the study) (Baron et al., 2009).

Up to now, the most potent inhibitors of this class are derived from imidazo[1,2-*a*]pyridines (Kazmierczak et al., 2017) and *N1* and/or *C5*-substituted imidazole analogs (Joachimiak et al., 2018). The analogs bearing C3-substituted pyridine (Coxon et al., 2001; Marma et al., 2007; Baron et al., 2009) and *N*-substituted benzimidazole (Bhuiyan et al., 2019) are less active against RGGT, although the latter were tested only as the so-called desoxy analogs, which are known to be less active than Cα-fluorinated analogs (Marma et al., 2007; Coxon et al., 2014). As these compounds bear two ionic groups, we have also undertaken the effort to synthesize their prodrug analogs. However, all thus obtained compounds showed unsatisfactory stability, leading only to trace amounts of the particular drug under studied conditions (Joachimiak et al., 2015). Future efforts would require the development of other prodrug design strategies.

We have previously demonstrated that phosphonocarboxylates with imidazo[1,2-*a*]pyridine ring substituted in the C6 position of the heterocycle show activity toward RGGT (Kazmierczak et al., 2017). Here, we elaborated on the synthesis of analogs equipped with diverse groups in this privileged position. Besides the varied character of substituents in terms of bulkiness, length, and geometry, we studied the influence of the basic and acidic nature of thus introduced groups on the activity of these compounds against RGGT.

## Results and Discussion

### Synthesis

**Figure 1:**
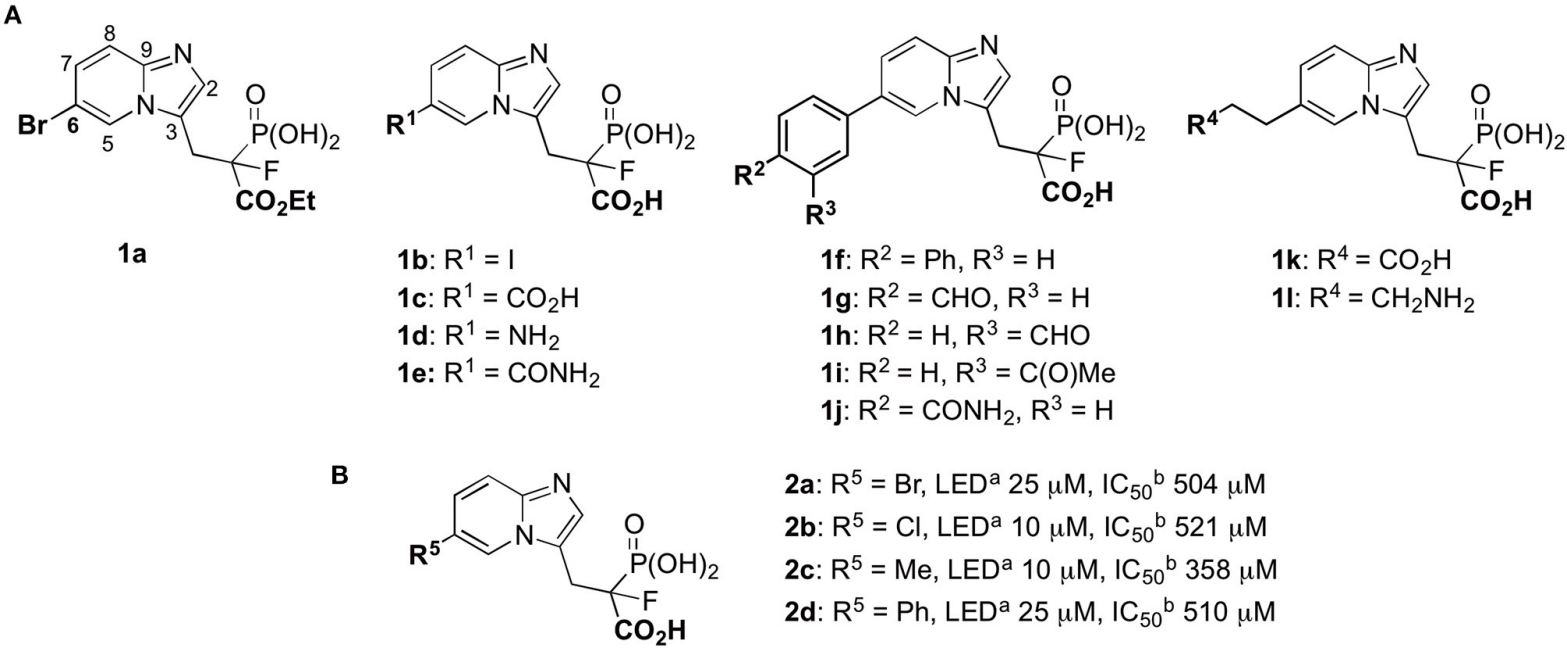
Phosphonocarboxylate inhibitors: **(A)** structures of the studied compounds; **(B)** known inhibitors of RGGT (Kazmierczak et al., 2017). ^a^LED = lowest effective dose (inhibition of Rab11A prenylation); ^b^half-maximal inhibitory concentration in the HeLa cell line.

In these studies, we synthesized a set of 12 new phosphonocarboxylate derivatives of a previously described potent RGGT inhibitor, namely 3-IPEHPC (McKenna et al., 2010; Błazewska et al., 2011). Novel compounds **1a–l**, all bearing a substituent in the privileged C6 position of imidazo[1,2-*a*]pyridine ring (Kazmierczak et al., 2017), were divided into three groups depending on the type of the functional group (Figure 1). The first group was constituted by five compounds (**1a–e**), in which the modification is directly attached to the imidazo[1,2-*a*]pyridine ring. One of the representatives of this group, compound **1a**, is equipped with carboxyester group in phosphonopropionate moiety. The second group consisted of five compounds (**1f–j**) in which the functional group is connected with heterocycle via the phenyl group, disubstituted either in *para* or *meta* positions. The third group (**1k–l**) consisted of compounds bearing either an amine or a carboxylic group connected with the imidazo[1,2-*a*]pyridine via the two- or three-carbon long linker, respectively.

**Scheme 1:**
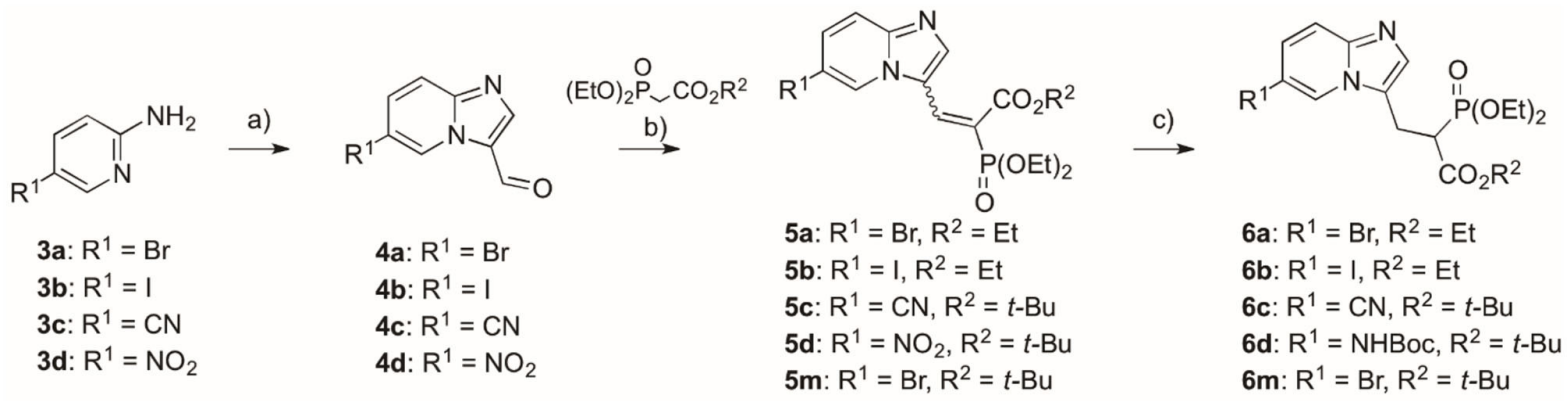
Reagents and conditions: (a) bromomanoloaldehyde, microwave irradiation, 10 min, 110°C, yield 54–80% (Kusy et al., 2019); (b) *tert*-butyl diethylphosphonoacetate or triethyl phosphonoacetate, TiCl_4_, TEA, DCM, overnight, rt, yield 42–66%; (c) for compounds **6a–c**, **m**: NaBH_4_, NiCl_2_·6H_2_O, MeOH, −40°C, yield 74–87%; for compound **6d**: (I) **5d**, H_2_, Pd/C, 24 h, rt, (II) Boc_2_O, DCM, 24 h, rt, (III) NaBH_4_, NiCl_2_·6H_2_O, MeOH, −40°C, yield (three steps) 48%.

**Scheme 2:**
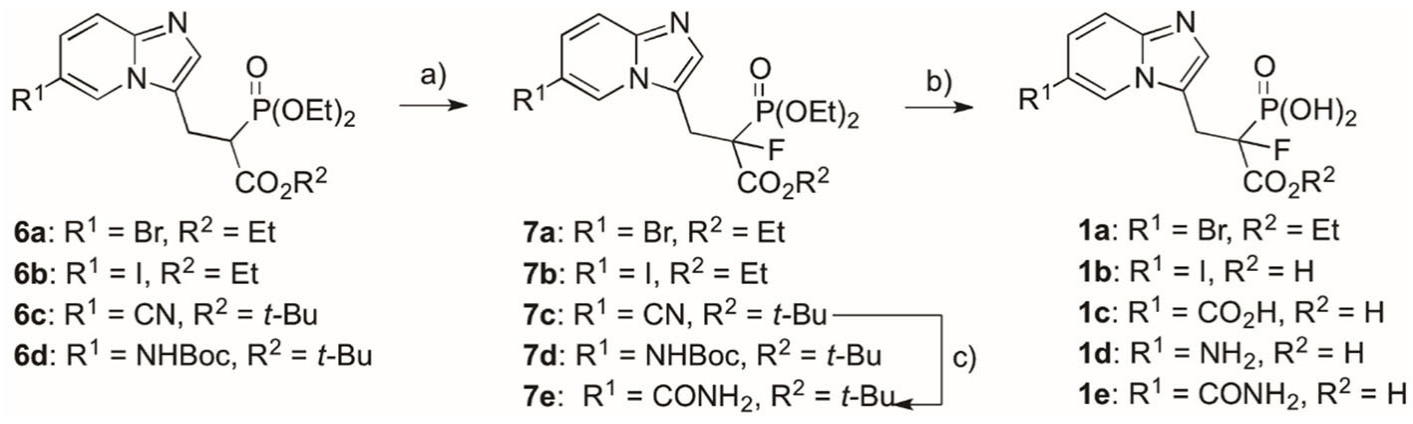
Reagents and conditions: (a) NaH, NFSI, THF, −70°C/20 min, then rt/overnight, yield 44–90%; (b) for compound **1a**: (I) **7a**, BTMS, ACN, 2 h, rt, (II) EtOH, 15 min, rt, yield 98%; for compounds **1b–d**: **7b–d**, 12 M HCl, 5 h, reflux, yield 48–73%; for compound **1e**: (I) **7e**, BTMS, ACN, 24 h, 35°C, (II) EtOH, 15 min, rt, (III) trifluoroacetic acid, 2 h, rt, yield 68%; (c) **7c**, H_2_O_2_, DMSO:EtOH 4:1 (*v*/*v*), 24 h, rt, yield 72%.

The synthesis of compounds representing the first group followed our previously reported strategy (Kazmierczak et al., 2017), which starts from appropriate commercially available 5-substituted-2-aminopyridines **3a–d**, which are transferred into imidazo[1,2-*a*]pyridine aldehydes **4a–d** (Kusy et al., 2019), the latter being subject to Knoevenagel condensation with trialkyl phospohonocarboxylate in the presence of TiCl_4_ and TEA (Scheme 1, details in Supplementary Materials) (Kazmierczak et al., 2017; Joachimiak et al., 2018). Thus, the obtained compounds **5a–c, m** were subjected to the reaction with NaBH_4_ and NiCl_2_ hydrate in MeOH, giving products **6a–c, m** with yields 48–87%. In the case of the synthesis of **6d**, it required a three-step procedure, in which reduction of the nitro group (H_2_, Pd/C), assisted by the partial hydrogenolysis of the double bonds, was followed by protection with Boc_2_O of the amine group, and then the use of NaBH_4_ and NiCl_2_x6H_2_O. Thus, the obtained compounds **6a–d** were subjected to fluorination using *N*-fluorobenzenesulfonimide (NFSI) and sodium hydride. Only compound **7c** was subjected to further transformation of the C6-substituent upon fluorination. In this case, the nitrile group was converted into an amide group using hydrogen peroxide in EtOH (compound **7e**, Scheme 2) (Ouvry et al., 2016).

The standard procedure of hydrolysis of the ester groups was applied for most of the compounds **7**, and it involved the use of 12 M hydrochloric acid to afford target products **1** with 48–73% yields (Scheme 2). Only compounds **7a** and **7e** required more cautious conditions in order to retain the carboxyester (**7a**) or amide groups (**7e**). Here, we used neutral conditions of McKenna's reaction, subjecting the compound to reaction with BTMS (McKenna and Schmidhuser, 1979; Justyna et al., 2020), followed by TFA-mediated deprotection of *tert*-butyl carboxyester group, whenever necessary (compound **7e**).

The route described above was also used for the synthesis of analogs representing the second and third groups. However, in these cases, the C6-substituents were introduced via Pd-mediated reaction at the later stages of the synthesis, using bromo substituted phosphonoacetate analogs, **5a**/**5m** or **6a**/**6m**.

**Scheme 3:**
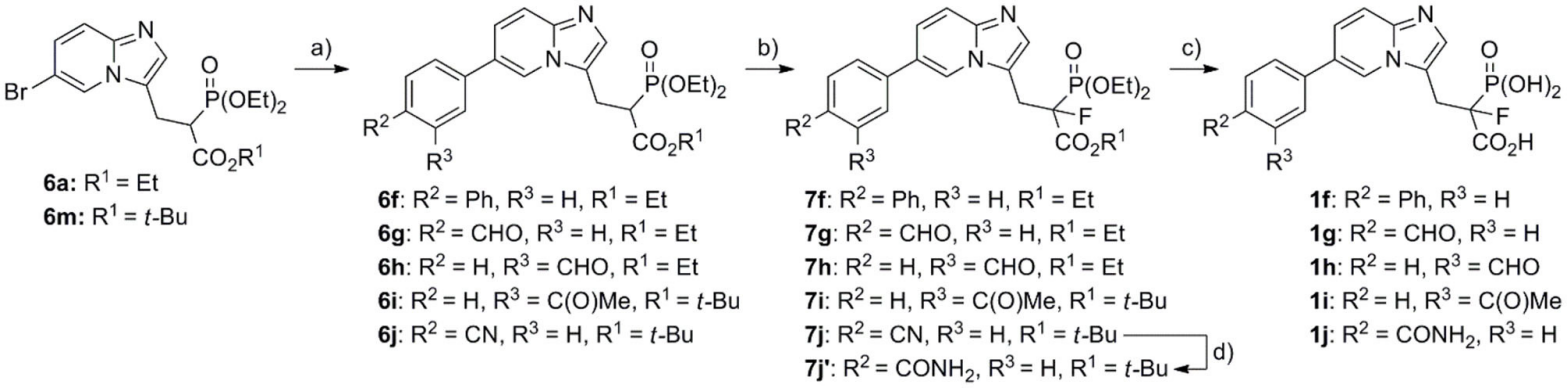
Reagents and conditions: (a) appropriate boronic acid, Na_2_CO_3_, Pd(PPh_3_)_4_, toluene:EtOH:H_2_O (2:1:2), yield 67–83%; (b) NaH, NFSI, THF, −70°C/20 min, then rt/overnight, yield 20–77%; (c) for compounds **1f−1i**: 12 M HCl, 5 h, reflux, yield 65–77%; for compound **1j**: (I) **7j′**, BTMS, ACN, 24 h, 35°C, (II) EtOH, 15 min, rt, (III) trifluoroacetic acid, 2 h, rt, yield 66%; (d) H_2_O_2_, DMSO, EtOH, 24 h, rt, yield 57%.

Compounds **1f–j**, bearing the benzene ring in the C6 position, were obtained in the Miyaura–Suzuki reaction between appropriate aryl boronic acid and phosphonoacetates **6a** and **6m**, in the presence of Na_2_CO_3_, Pd(PPh_3_)_4_, and toluene:EtOH:H_2_O (2:1:2), under microwave conditions (Scheme 3). Thus, the obtained compounds **6f–j** were subjected to fluorination using the procedure mentioned above, giving appropriate fluoro analogs **7f−7j**. The nitrile group in compound **7j** was transferred into amide **7j′**, using hydrogen peroxide in EtOH. The final acids **1f−1j** were obtained as above, either upon hydrolysis in 12 M HCl or using BTMS, followed by TFA (compound **7j′**), for dealkylation of phosphonate ester and the cleavage of *t*-butyl carboxyester, respectively.

**Scheme 4:**
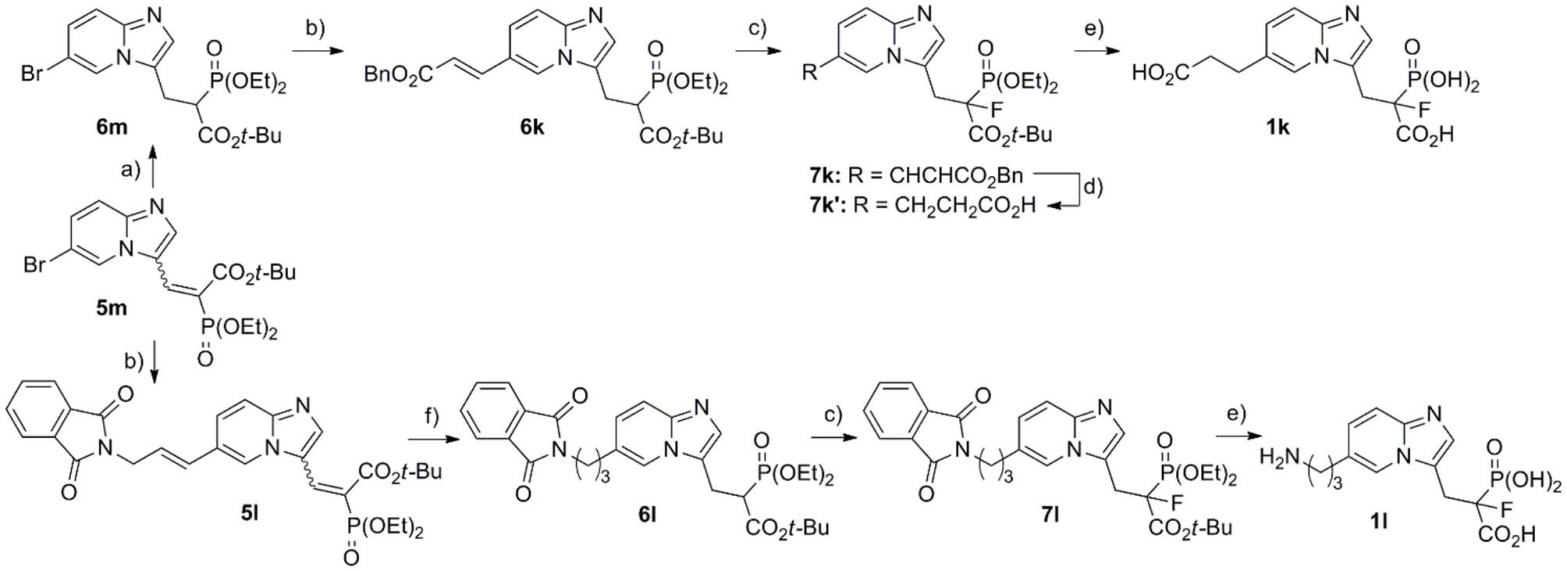
Reagents and conditions: (a) NaBH_4_, NiCl_2_·6H_2_O, MeOH, −40°C, yield 52–66%; (b) benzyl acrylate or 2-allylisoindoline-1,3-dione, Pd(OAc)_2_, tri(*o*-tolyl)phosphine, DIPEA, PCN, microwave irradiation, yield 63–65%, Bn = benzyl; (c) NaH, NFSI, THF, −70°C/20 min, then rt/overnight, yield 52–74%; (d) H_2_, Pd/C, MeOH, 24 h, rt, yield 85%; (e) **7k′** or **7l**, 12 M HCl, 5 h, reflux; (f) (I) H_2_, Pd/C, 24 h, rt, MeOH (II) NaBH_4_, NiCl_2_·6H_2_O, MeOH, −40°C, yield (two steps) 83%.

In order to obtain compounds **1k** and **1l**, equipped with an alkyl chain, the Mizoroki–Heck reaction was applied (Scheme 4), using Pd(OAc)_2_ and DIPEA and microwave irradiation (Kusy et al., 2020). As we have previously shown, such functionalization with appropriate olefin can be carried out for both types of compounds **5a**/**5m** and **6a**/**6m**. However, depending on the olefin, the type of the functional group present, and its sensitivity to reducing conditions, we chose to use **5m** (in the synthesis of amine bearing analog **1l**) and **6m** (for the synthesis of compound **1k** equipped with carboxylic acid). After the Mizoroki–Heck reaction, we subject compound **5l** to two-step reduction, obtaining fully saturated analog **6l**, which was subject to fluorination with NFSI and NaH (giving compound **7l**), followed by hydrolysis in 12 M HCl (see above) (Kazmierczak et al., 2017). In the case of analog with a benzyl carboxylate group, saturated analog **6m** was used in the Mizoroki–Heck reaction leading to compound **6k**. This product was subject to fluorination (giving compound **7k**, Scheme 4), and the newly created double bond was reduced simultaneously with the cleavage of benzyl ester, using hydrogenolytic conditions and Pd/C (giving compound **7k′**). The final acids **1k,l** were obtained upon hydrolysis (12 M HCl), with the yields 52 and 93%, respectively.

### Biological Studies

#### Cytotoxicity Assay

Inhibition of geranylgeranylation is known to reduce cell viability (Wiemer et al., 2009; Kazmierczak et al., 2017). Therefore, 6-substituted imidazo[1,2-*a*]pyridine analogs of α-phosphonocarboxylates generated in this work were initially screened using the human cervical carcinoma HeLa cell line by PrestoBlue® fluorescent viability assay. To find the most active compounds, HeLa cells were exposed to the incremental concentrations (25–2,000 μM) of phosphonocarboxylates to determine the half-maximal inhibitory concentration (IC_50_) (Supplementary Figure 1). Half of the 12 studied compounds turned out to be highly cytotoxic (IC_50_ < 150 μM or below), while three of them (**1a**, **1d**, and **1l**) displayed negligible inhibitory effects. The remaining analogs demonstrated a cytotoxic effect with IC_50_ values from 386 to 735 μM (Table 1).

**Table 1:** Cytotoxic activity^a^ of novel 6-substituted imidazo[1,2-*a*]pyridine analogs of α-phosphonocarboxylates and their influence on Rab11A, Rap1A/Rap1B, and Ras prenylation^b^ in the HeLa cell line.

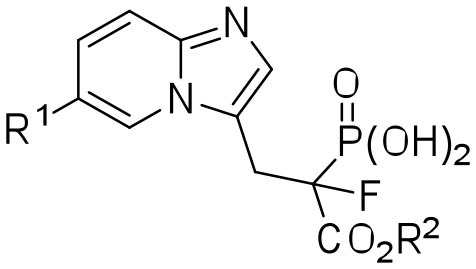					
	**Compound**	**R**^**1**^**/R**^**2**^	**Reduction of HeLa cell viability (IC**_**50**_**/μM)**	**Inhibition of Rab11A prenylation (LED/μM)**	**Inhibition of Rap1A/Rap1B prenylation (LED/μM)**	**Inhibition of Ras prenylation (LED/μM)**
1	**1a**	*R*^1^ = Br, *R*^2^ = Et	NE	NE	n.d.	n.d.
2	**1b**	*R*^1^ = I, *R*^2^ = H	47	50	NE	NE
3	**1c**	*R*^1^ = CO_2_H, *R*^2^ = H	386	100	NE	NE
4	**1d**	*R*^1^ = NH_2_, *R*^2^ = H	1,985	NE	n.d.	n.d.
5	**1e**	*R*^1^ = CONH_2_, *R*^2^ = H	510	100	NE	NE
6	**1f**	*R*^1^ = *p*-Ph-C_6_H_4_, *R*^2^ = H	70	NE	n.d.	n.d.
7	**1g**	*R*^1^ = *p*-CHO-C_6_H_4_, *R*^2^ = H	95	25	NE	NE
8	**1h**	*R*^1^ = *m*-CHO-C_6_H_4_, *R*^2^ = H	105	50	NE	NE
9	**1i**	*R*^1^ = *m*-CH_3_C(O)-C_6_H_4_, *R*^2^ = H	49	100	NE	NE
10	**1j**	*R*^1^ = *p*-CONH_2_-C_6_H_4_, *R*^2^ = H	143	NE	n.d.	n.d.
11	**1k**	*R*^1^ = (CH_2_)_2_CO_2_H, *R*^2^ = H	735	NE	n.d.	n.d.
12	**1l**	*R*^1^ = (CH_2_)_3_NH_2_, *R*^2^ = H	NE	NE	n.d.	n.d.
13	**2c**	*R*_1_ = Me, *R*_2_ = H	222	10	NE	n.d.
14	**3-IPEHPC**	Kazmierczak et al., 2017	167	50	100	n.d.

a HeLa cells were treated with the compounds for 72 h, and then a viable cell number was determined using the PrestoBlue^®^ assay. Data were calculated from the means of eight tested concentrations from at least three independent experiments. “NE” indicates that the IC_50_ parameter was not within up to 2 mM concentration of inhibitor.

b HeLa cells were treated in triplicate with test compounds for 48 h at six concentrations up to 100 μM. After incubation, cells were lysed, and isolation of cytosolic and membrane proteins was performed. Cytosolic fractions were western blotted for Rab11A, Rap1A/Rap1B, Ras, and β-actin. “LED” (lowest effective dose) indicates the lowest tested concentration of compounds for which the band corresponding to Rab11A, Rap1A/Rap1B, or Ras possessed higher intensity compared with the untreated control. “NE” stands for the lack of response in cells treated with up to 100 μM concentration of inhibitor. For compounds inactive on Rab11A prenylation at 100 μM concentration, further analysis was not performed (“n.d.”—not determined).

#### Structure–Activity Relationship (SAR)

Previously, we have identified the C6 position in the imidazo[1,2-*a*]pyridine ring of phosphonocarboxylates **2a–d** as the privileged one (Figure 1). Modifications only in this specific position are allowed to retain the activity against RGGT (Kazmierczak et al., 2017). Therefore, here we have extended the library of phosphonocarboxylate analogs, studying the effect of different bulkiness, length, and geometry as well as the acidic/basic character of the introduced substituents. Indeed, the nature of the substituents evaluated in this study influenced their activity.

**Figure 2:**
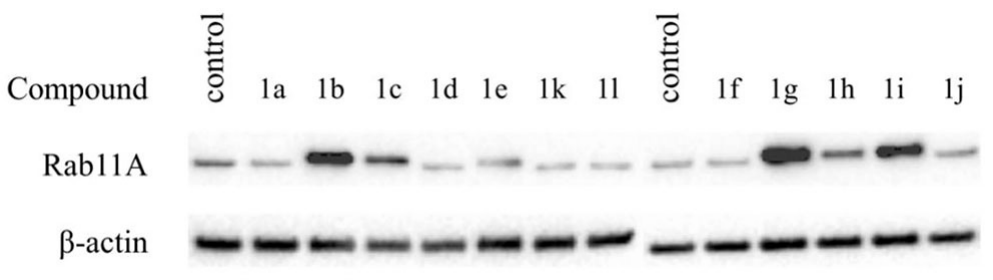
Effect of the studied compounds (**1a–l**) on Rab11A prenylation in HeLa cells. Cells were treated with phosphonocarboxylates at 100 μM for 48 h, lysed, and fractionated into cytosolic and membrane-rich fractions. Cytosolic fractions containing unprenylated proteins were separated by electrophoresis and western blotted for Rab11A and β-actin.

Cytotoxic effect was correlated with inhibition of Rab11A prenylation, except for compounds **1f**, **1j**, and **1k**, bearing biphenyl, 3-carbamoyl phenyl moiety, or a carboxylic group attached via a two-carbon linker, respectively (Table 1, Figure 2). The latter three compounds showed a cytotoxic effect resulted from different than RGGT inhibition mode of action. Among the non-active and non-cytotoxic compounds, we can differentiate compound **1a**, the only example in which carboxylic acid from phosphonoacetate residue was in the form of an ester, and analogs **1d** and **1l**, both equipped with a free amine group, either directly or via a three-carbon chain connected with imidazo[1,2-*a*]pyridine ring. Compounds that showed no inhibition of Rab11A prenylation at 100 μM screen (**1a**, **1d**, **1f**, **1j**, **1k**, **1l**) were excluded from further analysis.

The active analogs were selected for full panel six-dose assay to determine the lowest effective dose of inhibition of Rab11A, Rap1A/Rap1B, and Ras prenylation (Table 1), proteins modified by three prenyl transferases, RGGT, GGT-I (geranylgeranyl transferase type I), and FT (farnesyl transferase), respectively. This assessment allowed us to define the potency and selectivity of inhibitors against RGGT. Lovastatin, a hydroxymethylglutaryl (HMG)-coenzyme A (CoA) reductase inhibitor, was included as a positive control. Competitive displacement of the natural substrate by lovastatin prevents the downstream biosynthesis of cholesterol and prenylation with the geranylgeranyl or farnesyl moieties (Greenwood et al., 2006).

**Figure 3:**
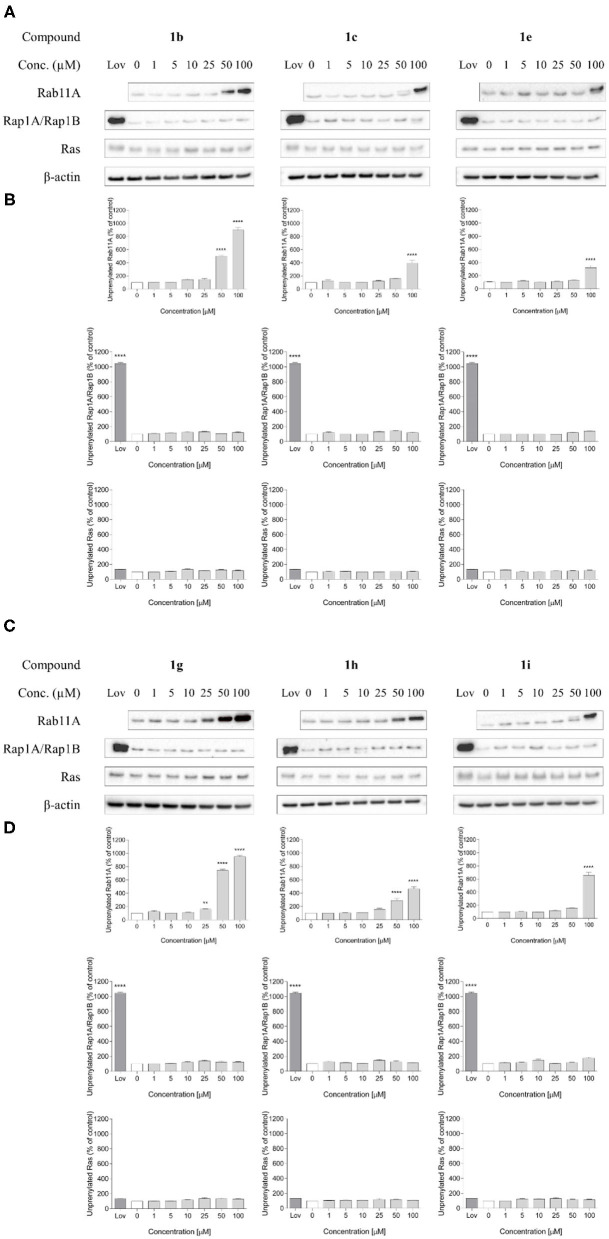
Effect of 6-substituted 3-IPEHPC analogs with iodo (**1b**), carboxyl (**1c**), amide (**1e**), 3- and 4-formylphenyl (**1g–h**), or 3-acetylphenyl (**1i**) group on Rab11A, Rap1A/Rap1B, and Ras prenylation in HeLa cells. Cells were exposed to indicated concentrations of compounds or 10 μM lovastatin (Lov) for 48 h. Subsequently, cells were lysed and isolation of cytosolic and membrane proteins was performed. Cytosolic fractions containing unprenylated proteins were western blotted for Rab11A, Rap1A/Rap1B, Ras, and β-actin **(A,C)**, and the immunoblot bands were quantified by densitometry analysis, normalized to β-actin, and presented as a percentage of controls **(B,D)**. Lovastatin was used as control. Even though it is the inhibitor of more upstream enzyme of mevalonate pathway, HMG-CoA reductase, and it is expected that treatment of cells with lovastatin should prevent the Ras accumulation at the membrane, we observed the failure of lovastatin to cause mislocalization of Ras. However, our observations of the lack of Ras inhibition by lovastatin in HeLa cells is in accordance with the results of Tennakoon et al. (2019). They compared how different statins (fluvastatin, atorvastatin, lovastatin) attenuate prenylation of Ras superfamily members in HeLa cells, showing that HeLa cells exposed to lovastatin exhibited negligible inhibition of both Ras members. Data represented mean ± SEM from at least three independent experiments; *p ≤ 0.05, **p ≤ 0.01, ***p ≤ 0.001, and ****p ≤ 0.0001.

Compound **1g**, which possessed a formyl group at the *para* position of the phenyl ring, appeared to be the most potent inhibitor of RGGT in the presented set of compounds, as measured by the lowest effective dose (LED). It inhibited Rab11A prenylation at a concentration of 25 μM (Figure 3), which makes this compound more potent than 3-IPEHPC, but slightly less potent than the previously reported most active compound of this class, **2c** (Kazmierczak et al., 2017). Shifting a formyl group to the *meta* position resulted in a two-fold weaker inhibition (**1h** LED = 50 μM; Figure 3), while the potency of methyl ketone analog **1i** was even lower, decreasing to 100 μM (Figure 3). The analog with an amide group in the *para* position of the phenyl ring, compound **1j**, was not active up to 100 μM concentration. This result might imply that either the amide substituent in this position is too bulky (compared with the formyl group) or the electron-dispersing character of the amide group does not promote interaction with the active site.

Three other active analogs possessed functional groups attached directly to the C6 of imidazo[1,2-*a*]pyridine ring. Compounds **1c** and **1e** with carboxylic acid and amide substituent, respectively, influenced RGGT activity only at the highest concentration tested: LED = 100 μM (Figure 3). The third inhibitor, compound **1b**, with iodine atom in the C6 position, exhibited a weaker activity toward inhibition of Rab11A prenylation compared to chlorine (**2b**) and bromine (**2a**) analogs (Figures 3A,B) (Kazmierczak et al., 2017), setting a trend of decreasing activity with increasing size and polarizability of the electronegative substituent. Its lowest effective dose was 50 μM (Figures 3A,B).

All tested compounds did not influence GGT-I or FT activity, because they affected neither Rap1A/Rap1B nor Ras prenylation (LED > 100 μM, Table 1, Figure 3), which makes them selective inhibitors of RGGT.

## Conclusions

A series of 12 novel 6-substituted imidazo[1,2-*a*]pyridine analogs of α-phosphonocarboxylates bearing structurally different moieties were designed as new RGGT inhibitors. In order to obtain such structurally different compounds, we designed three distinct synthetic routes, including such that allowed late-stage functionalization of the scaffold already bearing phosphonoacetate moiety.

Among the synthesized compounds, nine derivatives, **1b–c**, **1e−1k**, exhibited cytotoxic activity against cancer HeLa cells. Six compounds, **1b–c**, **1e**, and **1g–i**, displayed inhibitory properties toward Rab11A prenylation with LED ranging from 25 to 100 μM.

The most active compound bears *para*-substituted formylphenyl substituent. Since its *meta*-substituted analogs, bearing either formyl or methylketone groups, were also active, it may imply that there is an additional stabilization of the interaction between the inhibitor and an enzyme, thanks to a carbonyl group, possibly via arginine moiety in the active side (Kazmierczak et al., 2017). Other *para*-substituted phenyl analogs did not show the activity against RGGT in the studied concentration range. The studies clearly show that both aryl and alkyl amine residues have a detrimental effect on the activity against RGGT. On the other hand, the substituent's acidic character in this position may promote the activity (when directly connected with imidazo[1,2-*a*]pyridine ring) or suppress it (when connected with heterocyclic ring via alkyl chain). Interestingly, the trend observed previously for an electronegative spherical halogen substituent in the C6 position was confirmed, showing retainment of activity, which decreases with the increasing size of the atom. Irrespective of the activity of the synthesized analogs, most of them constitute an interesting point of departure for their further functionalization, thanks to the presence of easily derivatized amine, carboxylic, or carbonyl groups.

## Experimental Section

### General Synthesis

All reagents were purchased from commercial sources and used as obtained unless specified otherwise. Thin-layer chromatography was performed using silica gel 60 with F254 indicator on alumina plates. Compounds were purified using flash chromatography and 40–63 μm silica gel. Solvent ratios for the purification of compounds by flash chromatography are reported as volume ratios (*v*/*v*). Preparative HPLC for purification of compounds **1** was performed using Gilson Prep equipped with a UV–vis-156 detector and semipreparative column Kromasil 100–5-C18 (5 μm, 10 × 250 mm). NMR spectra were measured at 250.13 or 700 MHz for ^1^H NMR, 62.90 or 170 MHz for ^13^C NMR, and 283 or 101.30 MHz for ^31^P NMR on Bruker Avance DPX 250 and Bruker Avance II Plus 700 spectrometers, respectively. Chemical shifts (δ) are reported in parts per million (ppm) relative to internal residual CHCl_3_ in CDCl_3_ (δ 7.26 ^1^H NMR) or CDCl_3_ signal in ^13^C NMR (δ 77.16) or external 85% H_3_PO_4_ (δ 0 ppm ^31^P NMR). ^31^P NMR and ^13^C NMR spectra were proton-decoupled. Coupling constants (*J*) are quoted in hertz. The assignment of the signals in ^1^H NMR and ^13^C NMR was supported by two-dimensional experiments (COZY, HMQC, HMBC, DEPT-135).

### General Procedure for the Deprotection of Phosphonate Ester Groups via McKenna Reaction

#### Synthesis of Compounds **1a**, **1e**, and **1j**

Compounds (**7a**, **7e**, or **7j′)** were dissolved in acetonitrile. Then, bromotrimethylsilane (8–10 equiv.) was added. The mixture was stirred for 24 h at room temperature. Then, the solvent was evaporated under reduced pressure and EtOH (1 ml) was added. After 15 min of stirring, the solvent was evaporated. For compounds **1e** and **1j**, an additional step was required for deprotection of *tert*-butyl ester. Trifluoroacetic acid (2 ml) was added and the mixture was stirred for 1 h at 30°C. The product was concentrated under reduced pressure using co-evaporation with toluene. Products **1a**, **1e**, and **1j** were isolated by crystallization.

### General Procedure for the Hydrolysis With HCl

#### Synthesis of Compounds **1b–d**, **1f–i**, and **1k–l**

Compounds (**7b–d**, **7f–i**, or **7k–l**) (35 mg) were dissolved in 12 M HCl (2 ml). After 4–5 h of reflux, the mixture was evaporated to dryness. The products were obtained as white solids with 48–93% yields upon precipitation using water (2–4 ml). If needed, HPLC was performed, as detailed below.

##### (3-(6-Bromoimidazo[1,2-a]pyridin-3-yl)-1-ethoxy-2-fluoro-1-oxopropan-2-yl)phosphonic acid (1a)

Scale of the reaction: compound **7a** 80 mg. Product **1a** was purified by crystallization from H_2_O. Yield 98%. *HRMS m/z*: calculated 394.9802 (C_12_H_13_BrFN_2_O_5_P+H)^+^, found 394.9800. ^1^*H NMR* (700 MHz, CD_3_OD) δ 1.26 (t, ^3^*J*_HH_ = 7.1, CO_2_CH_2_CH_3_, 3H), 3.87–4.20 (m, CH_2_CPF, 2H), 4.26 (q, ^3^*J*_HH_ = 7.1, CO_2_CH_2_, 2H), 7.93 (dd, ^3^*J*_HH_ = 9.5, ^5^*J*_HH_ = 0.9, CH_Ar(8)_, 1H), 8.00 (s, CH_Ar(2)_, 1H), 8.13 (dd, ^3^*J*_HH_ = 9.5, ^4^*J*_HH_ = 1.7, CH_Ar(7)_, 1H), 9.10 [bs, CH_Ar(5)_, 1H]. ^31^*P NMR* (284 MHz, CD_3_OD) δ 6.01 (d, ^2^*J*_PF_ = 70.5). ^13^*C NMR* (176 MHz, CD_3_OD) δ 13.2 (s, CO_2_CH_2_CH_3_, 1C), 27.5 (bd, ^2^*J*_FC_ = 20.6, CH_2_CPF, 1C), 63.8 (s, CO_2_CH_2_, 1C), 96.8 (dd, ^1^*J*_FC_ = 193.9, ^1^*J*_PC_ = 143.2, CPF, 1C), 111.7 [s, C_Ar(6)_, 1C], 112.9 [s, CH_Ar(8)_, 1C], 121.5 [d, ^3^*J*_PC_ = 13.5, C_Ar(3)_, 1C], 122.1 [s, CH_Ar(2)_, 1C], 127.1 [bs, CH_Ar(5)_, 1C], 136.7 [s, CH_Ar(7)_, 1C], 138.9 [, C_Ar(9)_, 1C], 169.7 (d, ^2^*J*_PC_ = 24.1, CO_2_, 1C).

##### (3-(6-Iodoimidazo[1,2-a]pyridin-3-yl)-1-ethoxy-2-fluoro-1-oxopropan-2-yl)phosphonic acid (1b)

Scale of the reaction: compound **7b** 60 mg. Yield 48%. *H NMR* (700 MHz, D_2_O, pH 7) δ 3.59 (dt, ^2^*J*_HH_ = 16.0, ^3^*J*_FH_ = ^3^*J*_PH_ = 6.4, CH_2_CFP, 1H), 3.89 (dd, ^3^*J*_FH_ = 41.9, ^2^*J*_HH_ = 16.0, CH_2_CFP, 1H), 7.38 [d, ^3^*J*_HH_ = 9.4, CH_Ar(7)_, 1H], 7.41 [s, CH_Ar(2)_, 1H], 7.56 [m, ^3^*J*_HH_ = 9.4, CH_Ar(8)_, 1H], 8.84 [s, CH_Ar(5)_, 1H]. ^31^*P NMR* (283 MHz, D_2_O, pH 7) δ 10.08 (d, ^2^*J*_PF_ = 70.2). ^13^*C NMR* (176 MHz, D_2_O, pH 7) δ 29.1 (dd, ^2^*J*_FC_ = 21.3, ^2^*J*_PC_ = 3.4, CH_2_CFP, 1C), 75.0 [s, CH_Ar(6)_, 1C], 100.8 (dd, ^1^*J*_FC_ = 190.3, ^1^*J*_PC_ = 138.1, CH_2_CFP, 1C), 116.7 [s, CH_Ar(7)_, 1C], 122.0 [d, ^3^*J*_PC_ = 13.9, C_Ar(3)_, 1C], 130.5 [d, ^5^*J*_FC_ = 4.4, CH_Ar(5)_, 1C], 131.2 [s, CH_Ar(8)_, 1C], 133.2 [s, C_Ar(2)_, 1C], 144.2 [s, C_Ar(9)_, 1C], 176.5 (dd, ^2^*J*_FC_ = 21.0, ^2^*J*_PC_ = 3.4, CO_2_H, 1C).

##### 3-(2-Carboxy-2-fluoro-2-phosphonoethyl)imidazo[1,2-a]pyridine-6-carboxylic acid (1c)

Scale of the reaction: compound **7c**: 100 mg. Yield 73%. ^1^*H NMR* (700 MHz, D_2_O, pH 8) δ 3.71–3.78 (m, CH_2_CPF, 1H), 3.99 (ddd, ^3^*J*_FH_ = 39.9, ^2^*J*_HH_ = 16.3, ^3^*J*_PH_ = 2.7, CH_2_CPF, 1H), 7.65 [s, CH_Ar(2)_, 1H], 7.70 [d, ^3^*J*_HH_ = 9.3, ^5^*J*_HH_ = 0.8, CH_Ar(8)_, 1H], 8.00 [dd, ^3^*J*_HH_ = 9.3, ^4^*J*_HH_ = 1.5 CH_Ar(7)_, 1H], 9.00 [bs, CH_Ar(5)_, 1H]. ^31^*P NMR* (284 MHz, D_2_O, pH 8) δ 10.18 (d, ^2^*J*_PF_ = 75.2). ^13^*C NMR* (176 MHz, D_2_O) δ 28.3 (dd, ^2^*J*_FC_ = 21.3, ^2^*J*_PC_ = 3.7, CH_2_CPF, 1C), 99.1 (dd, ^1^*J*_FC_ = 191.8, ^1^*J*_PC_ = 144.0, CPF, 1C), 113.2 [s, CH_Ar(8)_, 1C], 122.7 [d, ^3^*J*_PC_ = 15.1, C_Ar(3)_, 1C], 124.2 [s, C_Ar(6)_, 1C], 127.4 [bs, CH_Ar(2)_, 1C], 128.1 [d, ^5^*J*_FC_ = 4.6 CH_Ar(5)_, 1C], 129.2 [s, CH_Ar(7)_, 1C], 143.5 [s, C_Ar(9)_, 1C], 171.9 [s, C_Ar(6)_CO_2_H, 1C], 174.9 (d, ^2^*J*_PC_ = 20.7, FCCO_2_H, 1C). ^19^*F NMR* (659 MHz, D_2_O, pH 8) δ −165.29 (ddd, ^2^*J*_PF_ = 75.2, ^3^*J*_HH_ = 39.9, ^3^*J*_HH_ = 7.5, 1F).

##### 3-(2-Carboxy-2-fluoro-2-phosphonoethyl)imidazo[1,2-a]pyridin-6-aminium chloride (1d)

Scale of the reaction: compound **7d** 60 mg. Yield 63%. Product **1d** was purified by preparative HPLC (eluent A: H_2_O:ACN:TFA 95:5:0.1 (*v*/*v*/*v*), isocratic, retention time 2.8 min) as eluent followed by lyophilization from 0.1 M HCl (repeated three times). ^1^*H NMR* (700 MHz, D_2_O, pH 2) δ 3.89 (ddd, ^2^*J*_HH_ = 17.1, ^3^*J*_FH_ = 11.1, ^3^*J*_PH_ = 6.6, CH_2_C(F)P, 1H), 4.04 (ddd, ^3^*J*_FH_ = 37.4, ^2^*J*_HH_ = 16.6, ^3^*J*_PH_ = 4.8, CH_2_C(F)P, 1H), 7.85 [d, ^3^*J*_HH_ = 9.6, CH_Ar(8)_, 1H], 7.92 [s, CH_Ar(2)_, 1H], 7.96 [d, ^3^*J*_HH_ = 9.6, CH_Ar(7)_, 1H], 8.64 [s, CH_Ar(5)_, 1H]. ^13^*C NMR* (176 MHz, D_2_O, pH 2) δ 27.6 (dd, ^2^*J*_FC_ = 20.3, ^2^*J*_PC_ = 2.9, CH_2_C(F)P, 1C), 96.9 (dd, ^1^*J*_FC_ = 193.2, ^1^*J*_PC_ = 144.1, C(F)P, 1C), 113.2 [s, CH_Ar(7)_, 1C], 117.9 [s, CH_Ar(5)_, 1C], 121.9 [d, ^3^*J*_PC_ = 12.8, C_Ar(3)_, 1C], 122.4 [s, CH_Ar(2)_, 1C], 128.4 [s, C_Ar(6)_, 1C], 128.4 [s, CH_Ar(8)_, 1C], 138.0 [s, C_Ar(9)_, 1C], 171.6 (d, ^2^*J*_FC_ = 23.9, CO_2_, 1C). ^31^*P NMR* (284 MHz, D_2_O, pH 2) δ 6.79 (d, ^2^*J*_PF_ = 67.1). *HRMS m*/*z*: (C_10_H_12_FN_3_O_5_P)^+^ calculated 304.0493, found 304.0485.

##### 3-(6-Carbamoylimidazo[1,2-a]pyridin-3-yl)-2-fluoro-2-phosphonopropanoic acid (1e)

Scale of the reaction: compound **7e** 100 mg. Product **1e** was purified by crystallization from H_2_O. Yield 68%. ^1^*H NMR* (700 MHz, D_2_O, pH 7) δ 3.74 (ddd, ^2^*J*_HH_ = 15.7, ^3^*J*_FH_ = 7.7, ^3^*J*_PH_ = 7.7, CH_2_CFP, 1H), 4.01 (ddd, ^3^*J*_FH_ = 39.4, ^2^*J*_HH_ = 16.3, ^3^*J*_PH_ = 3.0, CH_2_CFP, 1H), 7.71 [s, CH_Ar(2)_, 1H], 7.77 (d, ^3^*J*_HH_ = 9.4, CH_Ar_, 1H), 7.94 (d, ^3^*J*_HH_ = 9.1, CH_Ar_, 1H), 9.10 [bs, CH_Ar(5)_, 1H]. ^31^*P NMR* (283 MHz, D_2_O, pH 7) δ 10.19 (d, ^2^*J*_PF_ = 76.7). ^13^*C NMR* (176 MHz, D_2_O, pH 7) δ 28.4 (d, ^2^*J*_FC_ = 21.3, ^2^*J*_PC_ = 4.0, CH_2_CFP, 1C), 98.58 (dd, ^1^*J*_FC_ = 192.3, ^1^*J*_PC_ = 146.9, CH_2_CFP, 1C), 114.05 (s, CH_Ar_, 1C), 121.11 [s, CH_Ar(6)_, 1C], 122.85 [d, ^3^*J*_PC_ = 15.3, C_Ar(3)_, 1C], 127.3 [d, ^5^*J*_FC_ = 4.4, CH_Ar(5)_, 1C], 127.5 (s, CH_Ar_, 1C), 127.6 [s, CH_Ar(2)_, 1C], 143.2 [s, C_Ar(9)_, 1C], 166.2 (s, CONH_2_, 1C), 174.2 (dd, ^2^*J*_FC_ = 20.3, ^2^*J*_PC_ = 3.3, CO_2_, 1C). ^19^*F NMR* (659 MHz, D_2_O, pH 7) δ −165.91 (bdd, ^2^*J*_PF_ = 75, ^3^*J*_FH_ = 41, CH_2_C*F*P, 1F).

##### 3-(6-([1,1′-Biphenyl]-4-yl)imidazo[1,2-a]pyridin-3-yl)-2-fluoro-2-phosphonopropanoic acid (1f)

Scale of the reaction: compound **7f** 47 mg. Yield 77%. *HRMS m*/*z*: calculated 441.1011 (C_22_H_18_FN_2_O_5_P+H)^+^, found 441.1005. ^1^*H NMR* (700 MHz, D_2_O, pH 8) δ 3.71–3.80 (m, CH_2_CPF, 1H), 4.01 (ddd, ^3^*J*_FH_ = 40.4, ^2^*J*_HH_ = 16.1, ^3^*J*_PH_ = 1.7, CH_2_CPF, 1H), 7.42 (t, ^3^*J*_HH_ = 7.4, CH_Ar_, 1H), 7.50 (t, ^3^*J*_HH_ = 7.4, CH_Ar_, 2H), 7.56 [s, CH_Ar(2)_, 1H], 7.64–7.73 (m, CH_Ar_, 3H), 7.73–7.79 (m, CH_Ar_, 4H), 7.86 (bd, ^3^*J*_HH_ = 9.3, CH_Ar_, 1H), 8.74 [bs, CH_Ar(5)_, 1H]. ^31^*P NMR* (284 MHz, D_2_O, pH 8) δ 10.06 (d, ^2^*J*_PF_ = 72.9).

##### 2-Fluoro-3-(6-(4-formylphenyl)imidazo[1,2-a]pyridin-3-yl)-2-phosphonopropanoic acid (1g)

Scale of the reaction: compound **7g** 35 mg. Yield 65%. *HRMS m*/*z*: calculated 393.0647 (C_17_H_14_FN_2_O_6_P+H)^+^, found 393.0632. ^1^*H NMR* (700 MHz, D_2_O, pH 8) δ 3.67–3.77 (m, CH_2_CPF, 1H), 4.00 (bdd, ^3^*J*_FH_ = 41.9, ^2^*J*_HH_ = 16.2, CH_2_CPF, 1H), 7.51 [s, CH_Ar(2)_, 1H], 7.65 [bd, ^3^*J*_HH_ = 9.1, CH_Ar(7)_, 1H], 7.73 [bd, ^3^*J*_HH_ = 9.1, CH_Ar(8)_, 1H], 7.96–8.02 (m, C_6_H_4_, 2H), 8.05–8.09 (m, C_6_H_4_, 2H), 8.84 [bs, CH_Ar(5)_, 1H], 10.00 (s, CHO), 1H). ^31^*P NMR* (284 MHz, D_2_O, pH 8) δ 10.16 (d, ^2^*J*_PF_ = 70.3).

##### 2-Fluoro-3-(6-(3-formylphenyl)imidazo[1,2-a]pyridin-3-yl)-2-phosphonopropanoic acid (1h)

Scale of the reaction: compound **7h** 40 mg. Yield 61%. *HRMS m*/*z*: calculated 393.0647 (C_17_H_14_FN_2_O_6_P+H)^+^, found 393.0630. ^1^*H NMR* (700 MHz, D_2_O, pH 8) δ 3.64–3.72 (m, CH_2_CFP, 1H), 3.91–4.04 (m, CH_2_CFP, 1H), 7.55 [s, CH_Ar(2)_, 1H], 7.57–7.65 [m, CH_Ar(14, 8)_, 1H], 7.71–7.77 [m, CH_Ar(7)_, 1H], 7.82 [d, ^3^*J*_HH_ = 7.5, CH_Ar(15)_, 1H], 7.94 [d, ^3^*J*_HH_ = 7.5, CH_Ar(13)_, 1H], 7.99–8.02 [m, CH_Ar(11)_, 1H], 8.65 [bs, CH_Ar(5)_, 1H], 9.89 (s, CHO, 1H). ^31^*P NMR* (283 MHz, D_2_O, pH 8) δ 10.11 (d, ^2^*J*_PF_ = 73.5). ^13^*C NMR* (176 MHz, D_2_O, pH 8) δ 28.8 (dd, ^2^*J*_FC_ = 21.3, ^2^*J*_PC_ = 3.4, CH_2_CPF, 1C), 99.5 (dd, ^1^*J*_FC_ = 191.7, ^1^*J*_PC_ = 142.0, CPF, 1C), 114.2 [s, CH_Ar(8)_, 1C], 122.5 [d, ^3^*J*_PC_ = 14.8, C_Ar(3)_, 1C], 123.0 [d, ^5^*J*_FC_ = 3.9, CH_Ar(5)_, 1C], 126.5 [s, C_Ar(6)_, 1C], 127.2 [s, CH_Ar(2)_, 1C], 128.2 [s, CH_Ar(7, 11)_, 2C], 129.3 [s, CH_Ar(15)_, 1C], 129.9 [s, CH_Ar(14)_, 1C], 133.4 [s, CH_Ar(13)_, 1C], 136.1 [s, C_Ar(12)_, 1C], 136.5 [s, C_Ar(10)_, 1C], 141.9 [s, C_Ar(9)_, 1C], 175.2 (dd, ^2^*J*_FC_ = 20.3, ^2^*J*_PC_ = 2.7, CO_2_H, 1C), 195.9 (s, CHO, 1C).

##### 3-(6-(3-Acetylphenyl)imidazo[1,2-a]pyridin-3-yl)-2-fluoro-2-phosphonopropanoic acid (1i)

Scale of the reaction: compound **7i** 60 mg. Yield 70%. ^1^*H NMR* (700 MHz, D_2_O, pH 7) δ 3.70 (dt, ^2^*J*_HH_ = 16.0, ^3^*J*_FH_ = ^3^*J*_PH_ = 6.7, CH_2_CFP, 1H), 3.99 (dd, ^3^*J*_FH_ = 42.1, ^2^*J*_HH_ = 16.0, CH_2_CFP, 1H), 7.51 [s, CH_Ar(2)_, 1H], 7.63–7.71 [m, CH_Ar(7, 8, 15)_, 3H], 8.03 [d, ^3^*J*_HH_ = 7.8 Hz, CH_Ar(13)_, 1H], 8.05 [d, ^3^*J*_HH_ = 7.7 Hz, CH_Ar(14)_, 1H], 8.28 [t, ^4^*J*_HH_ = 1.8 Hz, CH_Ar(11)_, 1H], 8.75 [s, CH_Ar(5)_, 1H]. ^31^*P NMR* (283 MHz, D_2_O, pH 7) δ 10.22 (d, ^2^*J*_PF_ = 70.0). ^13^*C NMR* (176 MHz, D_2_O, pH 7) δ 29.2 (m, CH_2_CFP, 1C), 99.8–101.8 (m, CH_2_CFP, 1C), 115.9 [s, CH_Ar(8)_, 1C], 122.6 [d, ^3^*J*_PC_ = 14.5, C_Ar(3)_, 1C], 123.1 [d, ^5^*J*_FC_ = 3.5, CH_Ar(5)_, 1C], 125.4 [s, CH_Ar(12)_, 1C], 125.6 (s, CH_Ar_, 1C), 127.0 [s, CH_Ar(11)_, 1C], 127.8 [s, CH_Ar(13)_, 1C], 129.5 (s, CH_Ar_, 1C), 131.6 [s, CH_Ar(2)_, 1C], 132.6 [s, CH_Ar(14)_, 1C], 137.1 [s, C_Ar(6)_, 1C], 137.8 [s, C_Ar(10)_, 1C], 144.8 [s, C_Ar(9)_, 1C], 176.7 (dd, ^2^*J*_FC_ = 20.8, ^2^*J*_PC_ = 3.2, CO_2_H, 1C), 203.9 (s, C(O)CH_3_, 1C).

##### 3-(6-(4-Carbamoylphenyl)imidazo[1,2-a]pyridin-3-yl)-2-fluoro-2-phosphonopropanoic acid (1j)

Scale of the reaction: compound **7j′** 40 mg. Product **1j** was purified by crystallization from a mixture of MeOH:H_2_O 2:1 (*v*/*v*). Yield 66%. ^1^*H NMR* (700 MHz, D_2_O, pH 7) δ 3.67 (ddd, ^2^*J*_HH_ = 16.1, ^3^*J*_FH_ = 6.7, ^3^*J*_PH_ = 6.7, CH_2_CFP, 1H), 3.96 (ddd, ^3^*J*_FH_ = 42.0, ^2^*J*_HH_ = 16.4, ^3^*J*_PH_ = 1.8, CH_2_CFP, 1H), 7.46 [s, CH_Ar(2)_, 1H], 7.53 (d, ^3^*J*_HH_ = 9.3, CH_Ar_, 1H), 7.59 (dd, ^3^*J*_HH_ = 9.3, ^5^*J*_HH_ = 1.7, CH_Ar_, 1H), 7.75 [d, ^3^*J*_HH_ = 8.2, CH_Ar(11)_, 2H], 7.80 [d, ^3^*J*_HH_ = 8.4, CH_Ar(12)_, 2H], 8.65 [bs, CH_Ar(5)_, 1H]. ^31^*P NMR* (284 MHz, D_2_O, pH 7) δ 10.28 (d, ^2^*J*_PF_ = 70.2). ^13^*C NMR* (176 MHz, D_2_O, pH 7) δ 29.2 (dd, ^2^*J*_FC_ = 21.2, ^2^*J*_PC_ = 3.1, CH_2_CPF, 1C), 100.8 (dd, ^1^*J*_FC_ = 190.6, ^1^*J*_PC_ = 138.1, CPF, 1C), 115.7 (s, CH_Ar_, 1C), 122.5 [d, ^3^*J*_PC_ = 14.0, C_Ar(3)_, 1C], 123.0 [d, ^5^*J*_FC_ = 3.8, CH_Ar(5)_, 1C], 124.9 (s, CH_Ar_, 1C), 125.3 [s, C_Ar(6)_, 1C], 126.8 [s, CH_Ar(11)_, 2C], 127.9 [s, CH_Ar(12)_, 2C], 131.2 [s, C_Ar(10)_, 1C], 131.4 [s, CH_Ar(2)_, 1C], 140.7 [s, C_Ar(13)_, 1C], 144.6 [s, C_Ar(9)_, 1C], 172.2 (s, CONH_2_, 1C), 176.6 (dd, ^2^*J*_FC_ = 20.8, ^2^*J*_PC_ = 3.3, CO_2_, 1C). ^19^*F NMR* (659 MHz, D_2_O, pH 7) δ −164.52 (ddd, ^2^*J*_PF_ = 70.2, ^3^*J*_FH_ = 42.0, ^3^*J*_FH_ = 6.4, CH_2_CFP, 1F).

##### 3-(6-(2-Carboxyethyl)imidazo[1,2-a]pyridin-3-yl)-2-fluoro-2-phosphonopropanoic acid (1k)

Scale of the reaction: compound **7k′** 40 mg. Yield 52%. ^1^*H NMR* (700 MHz, D_2_O, pH 8) δ 2.78 (t, ^3^*J*_HH_ = 7.2, CH_2_CO_2_, 2H), 3.07 (t, ^3^*J*_HH_ = 7.2, CH_2_CH_2_CO_2_, 2H), 3.71–4.22 (m, CH_2_CPF, 2H), 7.69–7.89 [m, CH_Ar(2, 7, 8)_, 3H], 8.54 [bs, CH_Ar(5)_, 1H]. ^31^*P NMR* (284 MHz, D_2_O, pH 8) δ 7.05 (d, ^2^*J*_PF_ = 73.7). ^13^*C NMR* (176 MHz, D_2_O, pH 8) δ 27.0 (s, CH_2_CH_2_CO_2_, 1C), 27.6 (bd, ^2^*J*_FC_ = 21.3, CH_2_CPF, 1C), 34.4 (s, CH_2_CO_2_, 1C), 97.5 (m, CPF, 1C), 111.7 [s, CH_Ar(8)_, 1C], 121.2 [d, ^3^*J*_PC_ = 14.7, C_Ar(3)_, 1C], 121.3 [s, CH_Ar(2)_, 1C], 124.8 [d, ^5^*J*_FC_ = 4.2, CH_Ar(5)_, 1C], 130.2 [s, CH_Ar(6)_, 1C], 135.2 [s, CH_Ar(7)_, 1C], 139.1 [s, C_Ar(9)_, 1C], 171.6 (bs, FCCO_2_H, 1C), 176.9 (s, CH_2_CO_2_H, 1C).

##### 3-(3-(2-Carboxy-2-fluoro-2-phosphonoethyl)imidazo[1,2-a]pyridin-6-yl)propan-1-aminium chloride (1l)

Scale of the reaction: compound **7l** 75 mg. Product **1l** was purified by preparative HPLC (eluent A: H_2_O:ACN:TFA 95:5:0.1 (*v*/*v*/*v*), isocratic, retention time 3.3 min) as eluent followed by lyophilization from 0.1 M HCl (repeated three times). Yield 93%. ^1^*H NMR* (700 MHz, D_2_O, pH 2) δ 2.10 [tt, ^3^*J*_HH_ = 7.4 C_Ar(6)_CH_2_CH_2_, 2H], 2.93 [dd, ^3^*J*_HH_ = 7.5, 6.5 C_Ar(6)_CH_2_, 1H], 3.08 (t, ^3^*J*_HH_ = 7.8, CH_2_NH_2_, 2H), 3.89 (ddd, ^2^*J*_HH_ = 16.6, ^3^*J*_FH_ = 10.1, ^3^*J*_PH_ = 6.7, CH_2_C(F)P, 1H), 4.05 (ddd, ^3^*J*_FH_ = 38.0, ^2^*J*_HH_ = 16.5, ^3^*J*_PH_ = 4.1, CH_2_C(F)P, 1H), 7.84 [d, ^3^*J*_HH_ = 9.1, CH_Ar(8)_, 1H], 7.84 [s, CH_Ar(2)_, 1H], 7.88 [d, ^3^*J*_HH_ = 9.2, CH_Ar(7)_, 1H], 8.60 [s, CH_Ar(5)_, 1H]. ^13^*C NMR* (176 MHz, D_2_O, pH 2) δ 27.5 (s, CH_2_CH_2_NH_2_, 1C), 27.5 (d, ^2^*J*_FC_ = 19.5, CH_2_C(F)P, 1C), 28.6 [s, C_Ar(6)_CH_2_, 1C], 38.6 (s, CH_2_NH_2_, 1C), 97.0 (dd, ^1^*J*_FC_ = 192.8, ^1^*J*_PC_ = 144.2, C(F)P, 1C), 111.8 [s, CH_Ar(8)_, 1C], 121.1 [d, ^3^*J*_PC_ = 13.7, C_Ar(3)_, 1C], 121.4 [s, CH_Ar(2)_, 1C], 124.6 [d, *J*_FC_ = 3.4, CH_Ar(5)_, 1C], 130.2 [s, C_Ar(6)_, 1C], 135.1 [s, CH_Ar(7)_, 1C], 139.1 [s, C_Ar(9)_, 1C], 171.7 (d, ^2^*J*_FC_ = 23.7, CO_2_, 1C). ^31^*P NMR* (284 MHz, D_2_O, pH 2) δ 6.79 (d, ^2^*J*_PF_ = 72.0). *HRMS m*/*z*: (C_13_H_18_FN_3_O_5_P)^+^ calculated 346.0963, found 346.3316.

### Biological Studies—General

PrestoBlue® Cell Viability Reagent, Mem-PER™ Plus Membrane Protein Extraction Kit, and all reagents for cell culture were purchased from Life Technologies (Carlsbad, CA, USA). Bradford Protein Assay and Clarity™ Western ECL Substrate were obtained from Bio*-*Rad *(*Hercules, CA, USA). Protease inhibitor cocktail and lovastatin were purchased from Sigma (Saint Louis, MO, USA). Primary antibodies against Rab11A, Rap1A/Rap1B, and Ras were obtained from Abcam (Cambridge, UK), whereas primary antibodies against β-actin and secondary HRP-linked antibodies were purchased from Cell Signaling Technology (Beverly, MA, USA).

### HeLa Cell Culture

The cervical epithelial carcinoma HeLa cell line was purchased from the American Type Cell Collection (ATCC). Cells were cultured in Dulbecco's modified Eagle's medium (DMEM) with 10% fetal bovine serum (FBS), 100 IU/ml penicillin, 0.25 μg/ml amphotericin B, and 50 μg/ml neomycin. Cells were incubated in a humidified incubator at 37°C and 5% CO_2_. Stock solutions of all compounds have been prepared freshly in phosphate-buffered saline (PBS) (10 mM) and directly diluted in culture medium.

### *In vitro* Assessment of Cytotoxicity

HeLa cells were seeded into 96-well plates at a density of 4 × 10^3^ cells/well in 100 μl of culture medium. After 24 h, cells were washed with PBS and 100 μl of serum-free medium was added. Subsequently, HeLa cells were treated with compounds at eight concentrations (25, 50, 100, 250, 500, 750, 1,000, and 2,000 μM) for 72 h. After incubation, PrestoBlue® Cell Viability Reagent was applied to each well and fluorescence at Ex/Em = 530/590 nm was measured in a Synergy 2 Microplate Reader (BioTek, Vermont, USA) following 50 min incubation. The obtained fluorescence magnitudes were used to calculate cell viability expressed as a percentage of the untreated control cells' viability. The data expressed as the mean of at least three independent experiments were used to calculate the IC_50_ parameter.

### Determination of RGGT, GGT-I, and FT Activity Inhibition

HeLa cells were seeded into six-well cell culture plates at a density of 4 × 10^5^ cells/well in 3 ml of culture medium. On the following day, 1.5 ml of fresh serum-free medium was added after washing cells with 1 ml of PBS. Subsequently, cells were supplemented with studied compounds as well as lovastatin, which served as a positive control. After 48 h of incubation, cell monolayers were rinsed with PBS and detached using a trypsin–EDTA solution. To obtain cell fractions enriched with cytosolic or membrane proteins, containing protease inhibitor cocktail, Mem-PER™ Plus Membrane Protein Extraction Kit was applied according to the manufacturer's instructions. The concentration of proteins in both fractions was measured using the Bradford protein assay. Equal amounts of cytosolic proteins (30 μg) were resolved by 12% SDS-PAGE gels and transferred on 0.2 μm nitrocellulose membrane. Membranes were incubated with β-actin, Rab11A, Rap1A/Rap1B, or Ras primary antibodies overnight, and then an appropriate HRP-conjugated secondary antibody was applied. Anti-Ras antibodies are predicted to react with H-, N-, and K-Ras. Visualization of protein bands was obtained using enhanced chemiluminescence (ECL) method and was performed using the ChemiDoc™ MP Imaging System (Bio-Rad). Densitometry analysis was performed with ImageLab™ Software (Bio-Rad), and relative unprenylated protein band intensity was normalized to β-actin and quantified relative to controls (untreated cells).

### Statistical Analysis

Unless stated otherwise, biological results are presented as means ± standard error of the mean (SEM) from at least three independent experiments. Statistical analysis was performed using GraphPad Prism software (version 6.01 for Windows, GraphPad Software, La Jolla, CA, USA, www.graphpad.com). Statistical differences between mean values of inhibitor-treated and untreated control samples were evaluated using one-way analysis of variance (ANOVA) followed by Dunnett's multiple comparisons test. Confidence *p* levels are indicated by asterisks, with ^*^ denoting *p* ≤ 0.05, ^**^ denoting *p* ≤ 0.01, ^***^ denoting *p* ≤ 0.001, and ^****^ denoting *p* ≤ 0.0001.

## Data Availability Statement

The raw data supporting the conclusions of this article will be made available by the authors, without undue reservation.

## Author Contributions

KB conceived the study and was in charge of overall direction and planning. KB and EG-D supervised the project. DK, AM, JM, and KJ carried out the experiments. DK, JM, and KJ performed the synthesis and analyzed the NMR spectra. AM performed the biological studies. JM drafted the manuscript. JM and AM designed the figures and schemes. KB and JM took the lead in writing the manuscript, while EG-D and AM provided the biological part. All authors provided critical feedback and helped shape the research, analysis, and manuscript.

## Conflict of Interest

The authors declare that the research was conducted in the absence of any commercial or financial relationships that could be construed as a potential conflict of interest.
