# Supplementary material for: Synthesis of the 6-Substituted Imidazo[1,2-a]Pyridine-3-yl-2- Phosphonopropionic Acids as Potential Inhibitors of Rab Geranylgeranyl Transferase
Source: Front Chem. 2021 Jan 6;8:596162. doi: 10.3389/fchem.2020.596162 (PMC7815931; doi:10.3389/fchem.2020.596162)
Supplement: Supplementary file 1 [file Data_Sheet_1.PDF]

Supplementary Material

Synthesis of the 6-substituted imidazo[1,2-*a*]pyridine-3-yl-2-phosphonopropionic acids as potential inhibitors of Rab Geranylgeranyl Transferase

Damian Kusy,[a] Aleksandra Marchwicka,[b] Joanna Małolepsza,[a] Katarzyna Justyna,[a] Edyta Gendaszewska-Darmach,[b] Katarzyna M. Błażewska [a]*

[a] Institute of Organic Chemistry, Faculty of Chemistry, Lodz University of Technology, Żeromskiego Street 116, 90-924 Łódź, Poland;

[b] Institute of Molecular and Industrial Biotechnology, Faculty of Biotechnology and Food Sciences, Lodz University of Technology, Stefanowskiego Street 4/10, 90-924 Łódź, Poland;

*Corresponding author: katarzyna.blazewska@p.lodz.pl

Table of contents

1. Figure S1. The cytotoxic efficacy of 6-substituted imidazo[1,2-*a*]pyridine analogs of α-phosphonocarboxylates against HeLa cell line. 5

2. General information for the synthesis of compounds 4-7 6

2.1. General procedure for Knovenagel condensation - compounds **5a-d, m** 6

2.2. General procedure for the synthesis of compounds **6a-c, d*, m, l*** 8

2.3. General procedure for Suzuki cross-coupling - compounds **6e-h** 11

2.4. General procedure for Heck reaction - compounds **6k, 5l** 14

3.5. General procedure for the fluorination - compounds **7a-l** 15

3.6. Transformation of the nitrile group into amide – synthesis of compounds **7e** and **7j'** 20

3. Copies of ^{1}H NMR, ^{13}C NMR and ^{31}P NMR spectra 21

Figure S2. ^{1}H NMR of compound **1a** (700 MHz, CD_3OD) 21

Figure S3. ^{31}P NMR of compound **1a** (284 MHz, CD_3OD). 22

Figure S4. ^{13}C NMR of compound **1a** (176 MHz, CD_3OD). 22

Figure S6. ^{31}P NMR of compound **1b** (286 MHz, D_2O pH 7). 23

Figure S7. ^{13}C NMR of compound **1b** (176 MHz, D_2O pH 7). 24

Figure S9. ^{31}P NMR of compound **1c** (284 MHz, D_2O pH 8). 25

Figure S10. ^{13}C NMR of compound **1c** (176 MHz, D_2O pH 8). 25

Figure S11. ^{19}F NMR of compound **1c** (659 MHz, D_2O pH 8). 26

Figure S12. ^{1}H NMR of compound **1d** (700 MHz, D_2O pH 2). 26

Figure S13. ^{31}P NMR of compound **1d** (283 MHz, D_2O pH 2). 27

Figure S14. ^{13}C NMR of compound **1d** (176 MHz, D_2O pH 2). 27

Figure S15. ^{1}H NMR of compound **1e** (700 MHz, D_2O pH 7). 28
Figure S16. ^{31}P NMR of compound **1e** (283 MHz, D_2O pH 7). 28
Figure S17. ^{13}C NMR of compound **1e** (176 MHz, D_2O pH 8). 29
Figure S18. ^{19}F NMR of compound **1e** (659 MHz, D_2O pH 8). 29
Figure S19. ^{1}H NMR of compound **1f** (700 MHz, D_2O pH 8). 30
Figure S20. ^{31}P NMR of compound **1f** (284 MHz, D_2O pH 8). 30
Figure S21. ^{1}H NMR of compound **1g** (700 MHz, D_2O pH 8). 31
Figure S22. ^{31}P NMR of compound **1g** (284 MHz, D_2O pH 8). 31
Figure S23. ^{1}H NMR of compound **1h** (700 MHz, D_2O pH 8). 32
Figure S24. ^{31}P NMR of compound **1h** (283 MHz, D_2O pH 8). 32
Figure S25. ^{13}C NMR of compound **1h** (176 MHz, D_2O pH 8). 33
Figure S26. ^{1}H NMR of compound **1i** (700 MHz, D_2O pH 8). 33
Figure S27. ^{31}P NMR of compound **1i** (284 MHz, D_2O pH 8). 34
Figure S28. ^{13}C NMR of compound **1i** (176 MHz, D_2O pH 8). 34
Figure S29. ^{1}H NMR of compound **1j** (700 MHz, D_2O pH 7). 35
Figure S30. ^{31}P NMR of compound **1j** (284 MHz, D_2O pH 7). 35
Figure S31. ^{13}C NMR of compound **1j** (176 MHz, D_2O pH 7). 36
Figure S32. ^{1}H NMR of compound **1k** (700 MHz, D_2O pH 8). 36
Figure S33. ^{31}P NMR of compound **1k** (284 MHz, D_2O pH 8). 37
Figure S34. ^{13}C NMR of compound **1k** (176 MHz, D_2O pH 8). 37
Figure S35. ^{1}H NMR of compound **1l** (700 MHz, D_2O pH 2). 38
Figure S36. ^{31}P NMR of compound **1l** (283 MHz, D_2O pH 2). 38
Figure S37. ^{13}C NMR of compound **1l** (176 MHz, D_2O pH 2). 39
Figure S38. ^{1}H NMR of compound **5b** (700 MHz, $CDCl_3$). 39
Figure S39. ^{31}P NMR of compound **5b** (283 MHz, $CDCl_3$). 40
Figure S40. ^{13}C NMR of compound **5b** (176 MHz, $CDCl_3$). 40
Figure S41. ^{1}H NMR of compound **5c** (700 MHz, $CDCl_3$). 41
Figure S42. ^{31}P NMR of compound **5c** (283 MHz, $CDCl_3$). 41
Figure S43. ^{13}C NMR of compound **5c** (176 MHz, $CDCl_3$). 42
Figure S44. ^{1}H NMR of compound **5d** (700 MHz, $CDCl_3$). 42
Figure S45. ^{31}P NMR of compound **5d** (101 MHz, $CDCl_3$). 43
Figure S46. ^{13}C NMR of compound **5d** (176 MHz, $CDCl_3$). 43
Figure S47. ^{1}H NMR of compound **5l** (700 MHz, $CDCl_3$). 44
Figure S48. ^{31}P NMR of compound **5l** (283 MHz, $CDCl_3$). 44
Figure S49. ^{13}C NMR of compound **5l** (176 MHz, $CDCl_3$). 45
Figure S50. ^{1}H NMR of compound **6b** (700 MHz, $CDCl_3$). 45
Figure S51. ^{31}P NMR of compound **6b** (283 MHz, $CDCl_3$). 46

Figure S52. ^{13}C NMR of compound **6b** (176 MHz, $CDCl_3$). 46
Figure S53. ^{1}H NMR of compound **6c** (700 MHz, $CDCl_3$). 47
Figure S54. ^{31}P NMR of compound **6c** (283 MHz, $CDCl_3$). 47
Figure S55. ^{13}C NMR of compound **6c** (176 MHz, $CDCl_3$). 48
Figure S56. ^{1}H NMR of compound **6d** (250 MHz, $CDCl_3$). 48
Figure S57. ^{31}P NMR of compound **6d** (101 MHz, $CDCl_3$). 49
Figure S58. ^{1}H NMR of compound **6f** (700 MHz, $CDCl_3$). 49
Figure S59. ^{31}P NMR of compound **6f** (101 MHz, $CDCl_3$). 50
Figure S60. ^{13}C NMR of compound **6f** (176 MHz, $CDCl_3$). 50
Figure S61. ^{1}H NMR of compound **6g** (700 MHz, $CDCl_3$). 51
Figure S62. ^{31}P NMR of compound **6g** (101 MHz, $CDCl_3$). 51
Figure S63. ^{13}C NMR of compound **6g** (176 MHz, $CDCl_3$). 52
Figure S64. ^{1}H NMR of compound **6h** (700 MHz, $CDCl_3$). 52
Figure S65. ^{31}P NMR of compound **6h** (283 MHz, $CDCl_3$). 53
Figure S66. ^{13}C NMR of compound **6h** (176 MHz, $CDCl_3$). 53
Figure S67. ^{1}H NMR of compound **6i** (700 MHz, $CDCl_3$). 54
Figure S68. ^{31}P NMR of compound **6i** (283 MHz, $CDCl_3$). 54
Figure S69. ^{13}C NMR of compound **6i** (176 MHz, $CDCl_3$). 55
Figure S70. ^{1}H NMR of compound **6j** (700 MHz, $CDCl_3$). 55
Figure S71. ^{31}P NMR of compound **6j** (283 MHz, $CDCl_3$). 56
Figure S72. ^{1}H NMR of compound **6k** (700 MHz, $CDCl_3$). 56
Figure S73. ^{31}P NMR of compound **6k** (283 MHz, $CDCl_3$). 57
Figure S74. ^{13}C NMR of compound **6k** (176 MHz, $CDCl_3$). 57
Figure S75. ^{1}H NMR of compound **6l** (700 MHz, $CDCl_3$). 58
Figure S76. ^{31}P NMR of compound **6l** (101 MHz, $CDCl_3$). 58
Figure S77. ^{13}C NMR of compound **6l** (176 MHz, $CDCl_3$). 59
Figure S78. ^{1}H NMR of compound **7b** (700 MHz, $CDCl_3$). 59
Figure S79. ^{31}P NMR of compound **7b** (283 MHz, $CDCl_3$). 60
Figure S80. ^{13}C NMR of compound **7b** (176 MHz, $CDCl_3$). 60
Figure S81. ^{1}H NMR of compound **7c** (700 MHz, $CDCl_3$). 61
Figure S82. ^{31}P NMR of compound **7c** (283 MHz, $CDCl_3$). 61
Figure S83. ^{13}C NMR of compound **7c** (176 MHz, $CDCl_3$). 62
Figure S84. ^{19}F NMR of compound **7c** (659 MHz, $CDCl_3$). 62
Figure S85. ^{1}H NMR of compound **7d** (250 MHz, $CDCl_3$). 63
Figure S86. ^{31}P NMR of compound **7d** (101 MHz, $CDCl_3$). 63
Figure S87. ^{1}H NMR of compound **7e** (700 MHz, $CDCl_3$). 64
Figure S88. ^{31}P NMR of compound **7e** (284 MHz, $CDCl_3$). 64

Figure S89. ^{13}C NMR of compound **7e** (176 MHz, $CDCl_3$)........ 65
Figure S90. ^{1}H NMR of compound **7f** (700 MHz, $CDCl_3$)........ 65
Figure S91. ^{31}P NMR of compound **7f** (284 MHz, $CDCl_3$)........ 66
Figure S92. ^{13}C NMR of compound **7f** (176 MHz, $CDCl_3$)........ 66
Figure S93. ^{1}H NMR of compound **7g** (700 MHz, $CDCl_3$)........ 67
Figure S94. ^{31}P NMR of compound **7g** (284 MHz, $CDCl_3$)........ 67
Figure S95. ^{13}C NMR of compound **7g** (176 MHz, $CDCl_3$)........ 68
Figure S96. ^{1}H NMR of compound **7h** (700 MHz, $CDCl_3$)........ 68
Figure S97. ^{31}P NMR of compound **7h** (283 MHz, $CDCl_3$)........ 69
Figure S98. ^{13}C NMR of compound **7h** (176 MHz, $CDCl_3$)........ 69
Figure S99. ^{1}H NMR of compound **7i** (700 MHz, $CDCl_3$)........ 70
Figure S100. ^{31}P NMR of compound **7i** (283 MHz, $CDCl_3$)........ 70
Figure S101. ^{13}C NMR of compound **7i** (176 MHz, $CDCl_3$)........ 71
Figure S102. ^{1}H NMR of compound **7j** (700 MHz, $CDCl_3$)........ 71
Figure S103. ^{31}P NMR of compound **7j** (283 MHz, $CDCl_3$)........ 72
Figure S104. ^{13}C NMR of compound **7j** (176 MHz, $CDCl_3$)........ 72
Figure S105. ^{1}H NMR of compound **7j’** (700 MHz, $CDCl_3$)........ 73
Figure S106. ^{31}P NMR of compound **7j’** (283 MHz, $CDCl_3$)........ 73
Figure S107. ^{13}C NMR of compound **7j’** (176 MHz, $CDCl_3$)........ 74
Figure S108. ^{1}H NMR of compound **7k** (700 MHz, $CDCl_3$)........ 74
Figure S109. ^{31}P NMR of compound **7k** (283 MHz, $CDCl_3$)........ 75
Figure S110. ^{13}C NMR of compound **7k** (176 MHz, $CDCl_3$)........ 75
Figure S111. ^{1}H NMR of compound **7k’** (700 MHz, $CDCl_3$)........ 76
Figure S112. ^{31}P NMR of compound **7k’** (283 MHz, $CDCl_3$)........ 76
Figure S113. ^{13}C NMR of compound **7k’** (176 MHz, $CDCl_3$)........ 77
Figure S114. ^{1}H NMR of compound **7l** (700 MHz, $CDCl_3$)........ 77
Figure S115. ^{31}P NMR of compound **7l** (283 MHz, $CDCl_3$)........ 78
Figure S116. ^{13}C NMR of compound **7l** (176 MHz, $CDCl_3$)........ 78

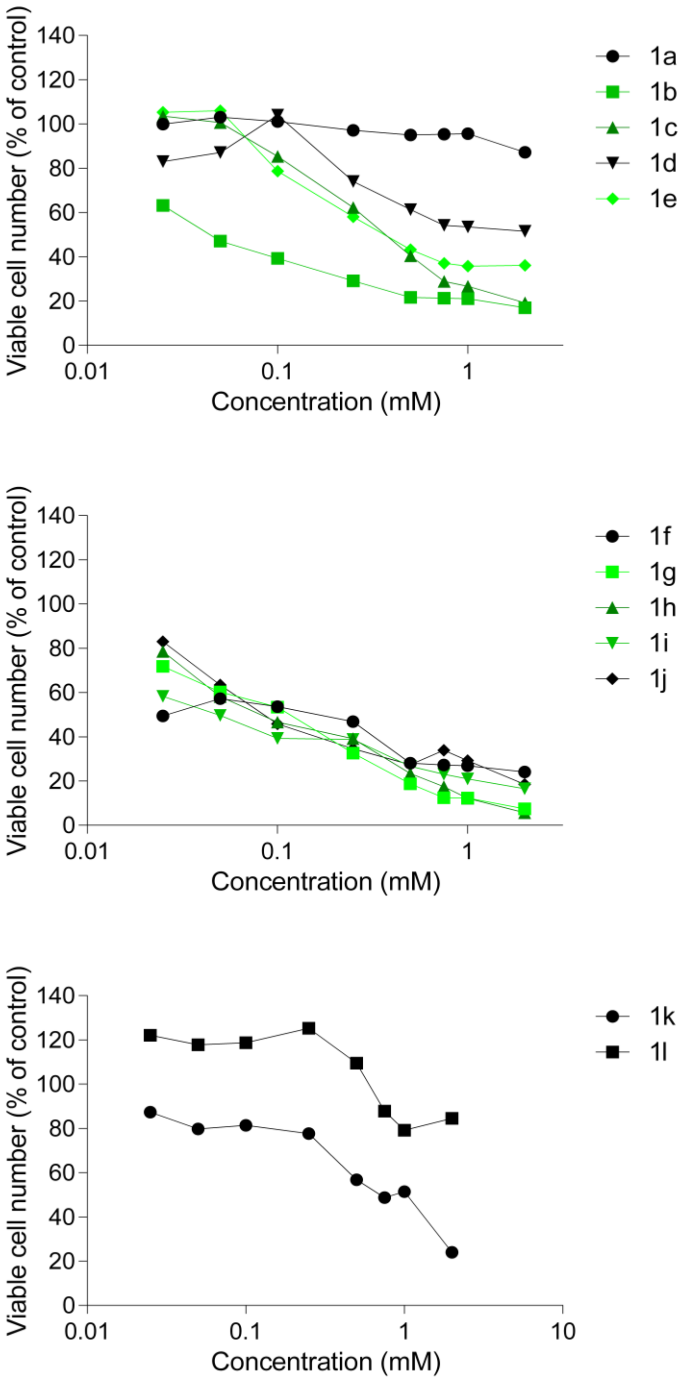

1. Figure S1. The cytotoxic efficacy of 6-substituted imidazo[1,2-*a*]pyridine analogs of α-phosphonocarboxylates against HeLa cell line.

HeLa cells were treated with the compounds for 72 h and cell viability was determined using the resazurin-based assay. Compounds that possessed the ability to inhibit RGGT activity are highlighted in shades of green.

2. General information for the synthesis of compounds 4-7

NMR spectra were measured at 250.13 or 700 MHz for ^{1}H NMR, 62.90 or 170 MHz for ^{13}C NMR, 283 or 101.30 MHz for ^{31}P NMR on Bruker Avance DPX 250 and Bruker Avance II Plus 700 spectrometrs, respectively. Chemical shifts (δ) are reported in parts per million (ppm) relative to internal residual $CHCl_3$ in $CDCl_3$ (δ 7.26 ^{1}H NMR) or $CDCl_3$ signal in ^{13}C NMR (δ 77.16) or external 85% H_3PO_4 (δ 0 ppm ^{31}P NMR). ^{31}P NMR and ^{13}C NMR spectra were proton-decoupled. Coupling constants (J) are quoted in Hz. The assignment of the signals in ^{1}H NMR and ^{13}C NMR was supported by two-dimensional experiments (COSY, HMQC, HMBC, DEPT-135). A monomode microwave reactor (CEM Discover SP) equipped with an IntelliVent pressure control system was used. The standard method was applied and maximum pressure was set to 250 psi. Temperatures of the reaction mixtures were measured with an external infrared sensor.

Low-resolution mass spectrometry (LRMS) measurements were performed using ASAP (Atmospheric solids analysis probe) Advion Expression S CMS (Compact Mass Spectrometer) equipped with an APCI source. Mass spectrometry parameters were as follows: m/z range 100-800, gas temp 254 °C, Capillary temperature 220 °C, Capillary Voltage 150V, APCI Corona Discharge 5uA. The spectra of the main fractions after Flash Chromatography purification were given.

6-bromoimidazo[1,2-*a*]pyridine-3-carbaldehyde (4a): obtained with the yield 80%, according to the procedure published earlier.[1] Structure was confirmed by the spectral data.

6-iodoimidazo[1,2-a]pyridine-3-carbaldehyde (4b): obtained with the yield 60%, according to the procedure published earlier.[1] Structure was confirmed by the spectral data.

3-formylimidazo[1,2-a]pyridine-6-carbonitrile (4c): obtained with the yield 73%, according to the procedure published earlier.[1] Structure was confirmed by the spectral data.

6-nitroimidazo[1,2-a]pyridine-3-carbaldehyde (4d): obtained with the yield 54%, according to the procedure published earlier.[1] Structure was confirmed by the spectral data.

2.1. General procedure for Knovenagel condensation - compounds 5a-d, m

Reaction was carried out under argon atmosphere using oven-dried glassware. A 100 mL two-neck round-bottom flask was charged with *tert*-butyl diethylphosphonoacetate (8.0 mmol, 1.2 equiv) dissolved in dichloromethane (20 mL). The solution was cooled down below -30 °C and $TiCl_4$ (0.88 mL, 8 mmol, 1.2 equiv) was slowly added *via* syringe, followed by Et_3N (2.63 mL, 18.7 mmol, 2.8 equiv). The reaction mixture was stirred for 15 minutes at -30 °C. Then, solution of appropriate aldehyde **(4a-d)** (6.67 mmol, 1 equiv) in DCM (20 mL) was dropwisely added for 15 minutes at the temperature below -30 °C. The resulting mixture was stirred at room temperature for 16 h and then quenched with 25 mL of water and was made basic with saturated Na_2CO_3 aqueous solution to pH>9. The water layer was extracted with diethyl ether (5 x 25

[1] Kusy, D.; Maniukiewicz, W.; Błażewska, K. M. Microwave-assisted synthesis of 3-formyl substituted imidazo[1,2-*a*]pyridines. *Tetrahedron Lett.* **2019**, *60*, 151244.

mL). Organic layer was dried over anhydrous Mg_2SO_4, solvent was evaporated and the residue was subjected to column chromatography using as eluent DCM:Acetone (85:15) to give product as (*Z*)/(*E*) mixture in the form of orange oil which slowly crystallizes.

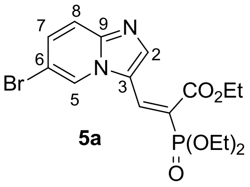

5a

Ethyl 3-(6-bromoimidazo[1,2-*a*]pyridin-3-yl)-2-(diethoxyphosphoryl)acrylate (**5a**). obtained from triethylphosphonoacetate, according to the procedure published earlier.[2] Obtained as a mixture of *E* and *Z* isomers (*E*/*Z* ratio: 50/1), with the yield 52%, Structure was confirmed by the spectral data.

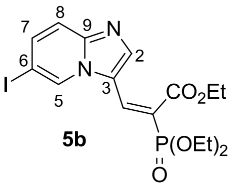

5b

Ethyl (*E*)-3-(6-cyanoimidazo[1,2-*a*]pyridin-3-yl)-2-(diethoxyphosphoryl)acrylate (5b): obtained from triethyl phosphonoacetate. Scale of compound **4b**: 500 mg. Yield: 42%.**^{1}H NMR** (700 MHz, $CDCl_3$) δ 1.31-1.37 (m, $CO_2CH_2CH_3$, CH_3CH_2OP, 9H), 4.10-4.22 (m, CH_2OP, 4H), 4.35 (q, $^3J_{HH}$ = 7.1, $COOCH_2$, 2H), 7.47 (dd, $^3J_{HH}$ = 9.3, $^5J_{HH}$ = 0.8, $CH_{Ar(8)}$, 1H), 7.53 (dd, $^3J_{HH}$ = 9.3, $^4J_{HH}$ = 1.6, $CH_{Ar(7)}$, 1H), 7.88 (bd, $^3J_{PC}$ = 23.7, CH=CP, 1H), 8.38 (bs, $CH_{Ar(2)}$, 1H), 8.56 (bm, $CH_{Ar(5)}$, 1H). **^{31}P NMR** (283 MHz, $CDCl_3$) δ 15.7. **^{13}C NMR** (176 MHz, $CDCl_3$) δ 14.2 (s, $CO_2CH_2CH_3$, 1C), 16.4 (d, $^3J_{PC}$ = 6.5, CH_3CH_2OP, 2C), 61.8 (s, CO_2CH_2, 1C), 62.8 (d, $^2J_{PC}$ = 5.2, CH_2OP, 2C), 77.2 (s, $CH_{Ar(6)}$, 1C), 117.5 (d, $^1J_{PC}$ = 184.3, CP, 1C), 119.4 (s, $CH_{Ar(8)}$, 1C), 120.3 (d, $^3J_{PC}$ = 24.2, $CH_{Ar(3)}$, 1C), 128.9 (s, $CH_{Ar(5)}$, 1C), 133.2 (d, $^2J_{PC}$ = 10.0, CH=CP, 1C), 135.3 (s, $CH_{Ar(7)}$, 1C), 141.0 (s, $CH_{Ar(2)}$, 1C), 146.4 (s, $C_{Ar(9)}$, 1C), 165.7 (d, $^2J_{PC}$ = 11.6, CO_2, 1C).

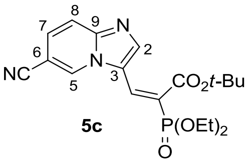

5c

***Tert*-butyl (*E*)-3-(6-cyanoimidazo[1,2-*a*]pyridin-3-yl)-2-(diethoxyphosphoryl)acrylate (5c):** obtained from *tert*-butyl diethylphosphonoacetate. Scale of reaction: compound **4c** 1.0 g. Yield: 66%. **HRMS m/z**: calculated 406.1527 ($C_{19}H_{24}N_3O_5P$ + H)$^+$, found 406.1528, **^{1}H NMR** (700 MHz, $CDCl_3$) δ 1.30 (t, $^3J_{HH}$ = 7.1, CH_3CH_2OP, 6H), 1.50 (s, $C(CH_3)_3$, 9H), 4.03 – 4.16 (m, CH_2OP, 4H), 7.39 (dd, $^3J_{HH}$ = 9.2, $^4J_{HH}$ = 1.6, $CH_{Ar(7)}$, 1H), 7.69-7.75 (m, CH=CP, $CH_{Ar(8)}$, 2H), 8.42 (s, $CH_{Ar(2)}$, 1H), 8.76 (bs, $CH_{Ar(5)}$, 1H), **^{31}P NMR** (283 MHz, $CDCl_3$) δ 14.82. **^{13}C NMR** (176 MHz, $CDCl_3$) δ 16.3 (d, $^3J_{PC}$ = 6.8, CH_3CH_2OP, 2C), 28.0 (s, $C(CH_3)_3$, 3C), 62.6 (d, $^2J_{PC}$ = 5.2, CH_2OP, 2C), 83.1 (s, $C(CH_3)_3$, 1C), 100.3 (s, $C_{Ar(6)}$, 1C), 115.8 (s, CN, 1C), 119.3 (s, $CH_{Ar(8)}$, 1C), 121.3 (d, $^3J_{PC}$ = 25.0, $C_{Ar(3)}$, 1C), 122.4 (d, $^1J_{PC}$ = 180.6, CP, 1C), 126.4 (s,

[2] Kaźmierczak, A.; Kusy, D.; Niinivehmas, S. P.; Gmach, J.; Joachimiak, Ł.; Pentikäinen, O. T.; Gendaszewska-Darmach, E.; Błazewska, K. M. Identification of the Privileged Position in the Imidazo[1,2-a]pyridine Ring of Phosphonocarboxylates for Development of Rab Geranylgeranyl Transferase (RGGT) Inhibitors. *J. Med. Chem.* **2017**, *60*, 8781–8800.

$\underline{C}H_{Ar(7)}$, 1C), 130.0 (s, $\underline{C}H_{Ar(5)}$, 1C), 130.5 (d, $^{2}J_{PC}$ = 10.0, $\underline{C}$H=CP, 1C), 141.3 (s, $\underline{C}H_{Ar(2)}$, 1C), 146.4 (s, $C_{Ar(9)}$, 1C), 164.5 (d, $^{2}J_{PC}$ = 10.4, $\underline{C}O_2$, 1C).

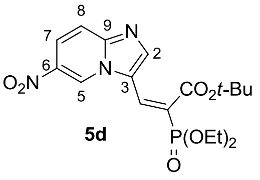

5d

***Tert*-butyl (*E*)-2-(diethoxyphosphoryl)-3-(6-nitroimidazo[1,2-*a*]pyridin-3-yl)acrylate (5d):** obtained from *tert*-butyl diethylphosphonoacetate. Scale of reaction: compound **4d** 500 mg. Yield: 49%. **^{1}H NMR** (700 MHz, $CDCl_3$) δ 1.33 (t, $^{3}J_{HH}$ = 7.2, $C\underline{H}_3CH_2OP$, 6H), 1.52 (s, $C(C\underline{H}_3)_3$, 9H), 4.09 – 4.20 (m, $C\underline{H}_2OP$, 4H), 7.73 (dd, $^{3}J_{HH}$ = 9.8, $^{5}J_{HH}$ = 0.8, $C\underline{H}_{Ar(8)}$, 1H), 7.76 (d, $^{3}J_{PH}$ = 23.6, C=C$\underline{H}$, 1H), 8.05 (dd, $^{3}J_{HH}$ = 9.8, $^{4}J_{HH}$ = 2.1, $C\underline{H}_{Ar(7)}$, 1H), 8.45 (s, $C\underline{H}_{Ar(2)}$, 1H), 9.35 (dd, $^{4}J_{HH}$ = 2.1, $^{5}J_{HH}$ = 0.8, $C\underline{H}_{Ar(5)}$, 1H); **^{13}C NMR** (176 MHz, $CDCl_3$) δ 16.3 (d, $^{3}J_{PC}$ = 6.4, $\underline{C}H_3CH_2OP$, 2C), 28.0 (s, $C(\underline{C}H_3)_3$, 3C), 62.8 (d, $^{2}J_{PC}$ = 5.1, $\underline{C}H_2OP$, 2C), 83.4 (s, $\underline{C}(CH_3)_3$, 1C), 118.2 (s, $\underline{C}H_{Ar(8)}$, 1C), 120.5 (s, $\underline{C}H_{Ar(7)}$, 1C), 122.6 (d, $^{3}J_{PC}$ = 24.7, $\underline{C}H_{Ar(3)}$, 1C), 123.4 (d, $^{1}J_{PC}$ = 180.0, P$\underline{C}$, 1C), 123.9 (s, $\underline{C}H_{Ar(5)}$, 1C), 130.0 (d, $^{2}J_{PC}$ = 10.1, PC=$\underline{C}$H, 1C), 138.3 (s, $\underline{C}_{Ar(6)}$, 1C), 142.2 (s, $\underline{C}H_{Ar(2)}$, 1C), 147.2 (s, $\underline{C}H_{Ar(9)}$, 1C), 164.6 (d, $^{2}J_{PC}$ = 10.5, $\underline{C}O_2$, 1C). **^{31}P NMR** (283 MHz, $CDCl_3$) δ 16.41.

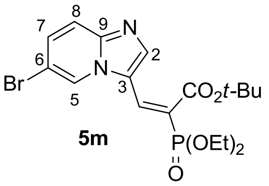

5m

***Tert*-Butyl 3-(6-bromoimidazo[1,2-*a*]pyridin-3-yl)-2-(diethoxyphosphoryl)acrylate** (**5m**): obtained with the yield 66%, according to the procedure published earlier.[3] Structure was confirmed by the spectral data.

2.2. General procedure for the synthesis of compounds 6a-c, d*, m, l*:

A 50 mL two-neck round-bottom flask was charged with **5a-c** or **5m** (1.83 mmol, 1 equiv.), $NiCl_2$ x 6 H_2O (0.52 g, 2.2 mmol, 1.2 equiv.) and dissolved in methanol (10 mL). Flask was submerged in a dry ice/acetone cooling bath (-40 °C) and then $NaBH_4$ (0.083 g, 2.2 mmol, 1.2 equiv.) was added. The mixture was stirred at (-40° C) for 8 min then quenched with 4 mL of NH_4Cl. Solution was made basic with saturated Na_2CO_3 solution to pH 9. The water layer was extracted with dichloromethane (4 x 10 mL). Organic layer was dried over anhydrous $MgSO_4$ solvent was evaporated and residue was subjected to column chromatography using DCM: acetone (100:20) system as eluent to give product as orange oil.

** Synthesis of compounds **6d** and **6l** required the use of additional steps the details of which are given below.*

[3] Kusy, D.; Wojciechowska, A.; Małolepsza, J.; Błażewska, K.M. Functionalization of the imidazo[1,2-*a*]pyridine ring in α-phosphonoacrylates and α-phosphonopropionates via microwave-assisted Mizoroki–Heck reaction. *Beilstein J. Org. Chem.* **2020,** *16,* 15–21.

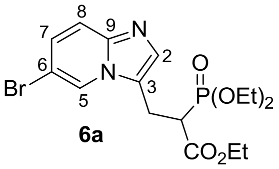

6a

Ethyl 3-(6-bromoimidazo[1,2-*a*]pyridin-3-yl)-2-(diethoxyphosphoryl)propanoate (6a): obtained with the yield 75%, according to the procedure published earlier.[2] Structure was confirmed by the spectral data.

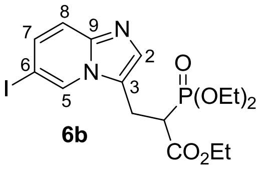

6b

Ethyl 3-(6-iodoimidazo[1,2-*a*]pyridin-3-yl)-2-(diethoxyphosphoryl)propanoate (6b): Scale of compound **5b**: 370 mg. Yield 78%. **^{1}H NMR** (700 MHz, $CDCl_3$) δ 1.20 (t, $^{3}J_{HH}$ = 7.1, $CO_2CH_2C\underline{H}_3$, 3H), 1.35 (t, $^{3}J_{HH}$ = 7.1, $C\underline{H}_3CH_2OP$, 3H), 1.36 (t, $^{3}J_{HH}$ = 7.1, $C\underline{H}_3CH_2OP$, 3H), 3.28-3.36 (m, $C\underline{H}_2C\underline{H}P$, 2H), 3.56 (m, $C\underline{H}_2CHP$, 1H), 4.10-4.25 (m, $C\underline{H}_2OP$, $CO_2C\underline{H}_2$, 6H), 7.32 (dd, $^{3}J_{HH}$ = 9.4, $^{4}J_{HH}$ = 1.6, $CH_{Ar(7)}$, 1H), 7.35-7.39 (m, $CH_{Ar(8)}$, $CH_{Ar(2)}$, 2H), 8.27 (bs, $CH_{Ar(5)}$, 1H). **^{31}P NMR** (283 MHz, $CDCl_3$) δ 20.6. **^{13}C NMR** (176 MHz, $CDCl_3$) δ 14.1 (s, $CO_2CH_2\underline{C}H_3$, 1C), 16.51 and 16.54 (2d, $^{3}J_{PC}$ = 5.9, $\underline{C}H_3CH_2OP$, 2C), 21.7 (d, $^{2}J_{PC}$ = 3.7, $\underline{C}H_2CHP$, 1C), 44.5 (d, $^{1}J_{PC}$ = 129.8, $\underline{C}HP$, 1C) 62.1 (s, $CO_2\underline{C}H_2$, 1C), 63.3 (d, $^{2}J_{PC}$ = 7.0, $\underline{C}H_2OP$, 1C), 63.4 (d, $^{2}J_{PC}$ = 6.6, $\underline{C}H_2OP$, 1C), 75.5 (s, $CH_{Ar(6)}$, 1C), 119.1 (s, $CH_{Ar(8)}$, 1C), 121.3 (d, $^{3}J_{PC}$ = 18.4, $C_{Ar(3)}$, 1C), 128.3 (s, $CH_{Ar(5)}$, 1C), 131.8 (s, $CH_{Ar(7)}$, 1C), 132.2 (s, $CH_{Ar(2)}$, 1C), 144.3 (s, $C_{Ar(9)}$, 1C), 168.2 (d, $^{2}J_{PC}$ = 5.1, $\underline{C}O_2$, 1C).

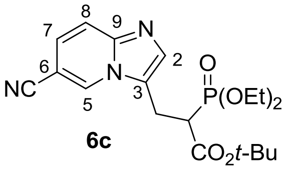

6c

***Tert*-butyl 3-(6-cyanoimidazo[1,2-*a*]pyridin-3-yl)-2-(diethoxyphosphoryl)propanoate (6c):** Scale of reaction: compound **5c** 700 mg. Yield 74%. **^{1}H NMR** (700 MHz, $CDCl_3$) δ 1.34-1.38 (m, $(C\underline{H}_3)_3C$, $C\underline{H}_3CH_2OP$, 15H), 3.22 (ddd, $^{2}J_{PH}$ = 23.1, $^{3}J_{HH}$ = 11.2, $^{3}J_{HH}$ = 3.3, $C\underline{H}P$, 1H), 3.36 (ddd, $^{2}J_{HH}$ = 15.7, $^{3}J_{PH}$ = 10.2, $^{3}J_{HH}$ = 3.3, $C\underline{H}_2CHP$, 1H), 3.55 (ddd, $^{2}J_{HH}$ = 15.7, $^{3}J_{HH}$ = 11.2, $^{3}J_{PH}$ = 7.3, $C\underline{H}_2CHP$, 1H), 4.13 - 4.25 (m, $C\underline{H}_2OP$, 4H), 7.25 (dd, $^{3}J_{HH}$ = 9.4, $^{4}J_{HH}$ = 1.6, $CH_{Ar(7)}$, 1H), 7.57 (s, $CH_{Ar(2)}$, 1H), 7.65 (d, $^{3}J_{HH}$ = 9.4, $^{5}J_{HH}$ = 1.0 $CH_{Ar(8)}$, 1H), 8.55 (bs, $CH_{Ar(5)}$, 1H), **^{31}P NMR** (283 MHz, $CDCl_3$) δ 20.83. **^{13}C NMR** (176 MHz, $CDCl_3$) δ 16.52 (d, $^{3}J_{PC}$ = 6.1, $\underline{C}H_3CH_2OP$, 1C), 16.54 (d, $^{3}J_{PC}$ = 6.0, $\underline{C}H_3CH_2OP$, 1C), 21.4 (d, $^{2}J_{PC}$ = 3.6, $\underline{C}H_2CHP$, 1C), 27.9 (s, $C(\underline{C}H_3)_3$, 3C), 45.6 (d, $^{1}J_{PC}$ = 129.5, $\underline{C}HP$, 1C), 63.2 (d, $^{2}J_{PC}$ = 7.0, $\underline{C}H_2OP$, 1C), 63.3 (d, $^{2}J_{PC}$ = 6.4, $\underline{C}H_2OP$, 1C), 83.1 (s, $\underline{C}(CH_3)_3$, 1C), 98.8 (s, $\underline{C}N$, 1C), 116.7 (s, $CH_{Ar(6)}$, 1C), 119.0 (s, $CH_{Ar(8)}$, 1C), 123.4 (d, $^{3}J_{PC}$ = 18.5, $C_{Ar(3)}$, 1C), 123.5 (s, $CH_{Ar(7)}$, 1C), 130.0 (s, $CH_{Ar(5)}$, 1C), 134.3 (s, $CH_{Ar(2)}$, 1C), 144.8 (s, $C_{Ar(9)}$, 1C), 167.3 (d, $^{2}J_{PC}$ = 5,1 , $\underline{C}O_2$, 1C).

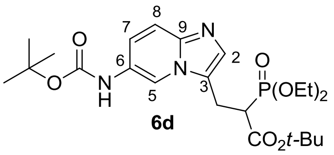

6d

***Tert*-butyl 3-(6-((*tert*-butoxycarbonyl)amino)imidazo[1,2-*a*]pyridin-3-yl)-2-(diethoxyphosphoryl)propanoate (6d):** obtained in three-step synthesis. The first step involved the reduction of nitro group. This reaction was carried out in a single-neck flask equipped with two-way stopcock, which enabled degassing the system (vacuum– hydrogen – vacuum, three times). In a single-neck flask compound **5d** (520 mg, 1.23 mmol) and 10% Pd/C (60 mg) was placed in AcOEt (60 mL). The system was degassed using a two-way stopcock. This suspension was stirred for 48 h at room temperature. The catalyst was then filtered off through a thin layer of Celite 500, and the filtrate was evaporated to dryness. Thus obtained amine derivative was used in the second step which involved introduction of *tert*-butyloxycarbonyl protecting group (Boc). Amine derivative, obtained in the reduction step, was dissolved in DCM (30 mL). Then, Boc_2O (2 equiv., 2.82 mmol, 616 mg) was added and the mixture was stirred for 24 h at room temperature. Next, the solvent was evaporated and obtained oil subjected to the third step, reduction of carbon double bond *HC=CH*CO_2*t*Bu using the procedure with $NaBH_4$ previously described for compound **6a**. Compound **6d** was purified by flash chromatography using Gilson PLC 2250 purification system. Eluents A: DCM and B: Acetone, to both of them TEA was added, 1mL/(1L of eluent) (gradient 0→30 min. 0→70%B, retention time 24 min.). Yield: 48% (350 mg). **^{1}H NMR** (700 MHz, $CDCl_3$) δ 1.38 (t, $^3J_{HH}$ = 7.2, C$\underline{H}_3$$CH_2$OP, 6H), 1.39, (s, (C$\underline{H}_3$)$_3$OCC, 9H), 1.53 (s, (C$\underline{H}_3$)$_3$COC(O)N, 18H), 3.37 – 3.19 (m, C$\underline{H}_2$C$\underline{H}$P, 2H), 3.63 – 3.46 (m, C$\underline{H}_2$C$\underline{H}$P, 1H), 4.29 – 4.14 (m, C$\underline{H}_2$OP, 4H), 6.96 (dd, $^3J_{HH}$ = 9,5, $^4J_{HH}$ = 2.0, C$\underline{H}_{Ar}$, 1H), 7.41 (s, C$\underline{H}_{Ar(2)}$, 1H), 7.54 (d, $^3J_{HH}$ = 9.2, C$\underline{H}_{Ar}$, 1H), 8.52 (bs, C$\underline{H}_{Ar(5)}$, 1H). **^{31}P NMR** (101 MHz, $CDCl_3$) δ 21.84.

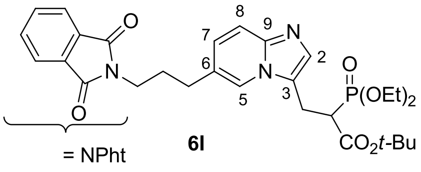

6l

***Ter*t-butyl 2-(diethoxyphosphoryl)-3-(6-(3-(1,3-dioxoisoindolin-2-yl)propyl)imidazo[1,2-*a*]pyridin-3-yl)propanoate (6l):** obtained according to the two-step reduction procedure: The first stage involved the reduction of double bond *HC=CH*CH_2NPht. This reaction was carried out in a single-neck flask equipped with two-way stopcock, which enabled degassing the system (vacuum– hydrogen –vacuum, three times). In a single-neck flask compound **5l** (200 mg) and 10% Pd/C (10 mg) was placed in EtOH (10 mL). The system was degassed using a two-way stopcock. This suspension was stirred overnigth at room temperature. The catalyst was then filtered off through a thin layer of Celite 500, and the filtrate was evaporated to dryness. Thus obtained compound was used in the second step which involved reduction of doube bond *HC=CH*CO_2*t*Bu using the general procedure for reduction. Yield (two-step reduction): 83% (167 mg). **^{1}H NMR** (700 MHz, $CDCl_3$) δ 7.74 (s, C$\underline{H}_{Ar(5)}$, 1H), 7.68 – 7.65 (m, C$\underline{H}_{Pht}$, 2H), 7.57 – 7.54 (m, C$\underline{H}_{Pht}$, 2H), 7.34 (d, $^3J_{HH}$ = 9.2, C$\underline{H}_{Ar(8)}$, 1H), 7.26 (s, C$\underline{H}_{Ar(2)}$, 1H), 6.94 (dd, $^3J_{HH}$ =

9.3, $^4J_{HH}$=1.5, C$\underline{H}_{Ar(7)}$, 1H), 4.16 – 4.11 (m, C$\underline{H}_2$OP, 4H), 3.66 (t, $^3J_{HH}$ = 6.89, C$\underline{H}_2$NPht, 2H), 3.45 (ddd, $^2J_{HH}$ = 15.5, $^3J_{HH}$ = 11.9, $^3J_{PH}$ = 6.7, C$\underline{H}_2$C(H)P, 1H), 3.25 (ddd, $^2J_{PH}$ = 23.5, $^3J_{HH}$ = 12.0, $^3J_{HH}$ = 3.1, CH_2C($\underline{H}$)P, 1H), 3.22 (ddd, $^2J_{HH}$ = 15.3, $^3J_{PH}$ = 9.9, $^3J_{HH}$ = 3.0, C$\underline{H}_2$C(H)P, 1H), 2.63 – 2.57 (m, C$\underline{H}_2CH_2CH_2$NPht, 2H), 2.01 – 1.95 (m, C$\underline{H}_2CH_2$NPht, 2H), 1.29 (s, C(C$\underline{H}_3)_3$, 9H), 1.28 (t, $^3J_{HH}$ = 7.0, C$\underline{H}_3CH_2$OP, 6H). **^{13}C NMR** (176 MHz, $CDCl_3$) δ 168.1 (s, $\underline{C}$ON, 2C), 167.1 (d, $^2J_{PC}$ = 5.1, $\underline{C}O_2$, 1C), 144.4 (s, $\underline{C}_{Ar(9)}$, 1C), 133.7 (s, $\underline{C}H_{Pht}$, 2C), 131.6 (s, $\underline{C}CH_{Pht}$, 2C), 131.2 (s, $\underline{C}H_{Ar(2)}$, 1C), 125.2 (s, $\underline{CH}_{Ar(7)}$, 1C), 124.8 (s, $\underline{C}_{Ar(6)}$, 1C), 122.8 (s, $\underline{C}H_{Pht}$, 2C), 120.9 (d, $^3J_{PC}$ = 19.4, $\underline{C}_{Ar(3)}$, 1C), 120.8 (s, $\underline{C}H_{Ar(5)}$, 1C), 117.3 (s, $\underline{C}H_{Ar(8)}$, 1C), 82.2 (s, $CO_2\underline{C}Me_3$, 1C), 62.9 – 62.6 (m, $\underline{C}H_2$OP, 2C), 44.8 (d, $^2J_{PC}$ = 128.9, $CH_2\underline{C}$(H)P, 1C), 37.1 (s, $\underline{C}H_2$NPht, 1C), 29.9 (s, $\underline{C}H_2CH_2CH_2$NPht, 1C), 28.8 (s, $\underline{C}H_2CH_2$NPht, 1C), 27.6 (s, C($\underline{C}H_3)_3$, 3C), 21.4 (d, $^3J_{PC}$ = 2.7, $\underline{C}H_2$C(H)P, 1C), 16.2 (d, $^3J_{PC}$ = 5.9, $\underline{C}H_3CH_2$OP, 1C), 16.2 (d, $^3J_{PC}$ = 6.1, $\underline{C}H_3CH_2$OP, 1C). **^{31}P NMR** (101 MHz, $CDCl_3$) δ 22.10.

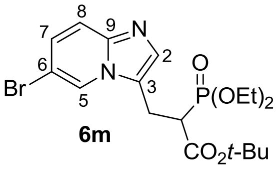

6m

***Tert*-butyl 3-(6-bromoimidazo[1,2-*a*]pyridin-3-yl)-2-(diethoxyphosphoryl)propanoate (6m)**: obtained with the yield 87%, according to the procedure published earlier.[3] Structure was confirmed by the spectral data.

2.3. General procedure for Suzuki cross-coupling - compounds 6e-h

compound **6a** or **6m** (0.23 mmol, 1 equiv.), arylboronic acid (0.45 mmol, 1.5 equiv.), Na_2CO_3 (27 mg, 0.25 mmol, 1.1 equiv.) and solvents - toluene:EtOH: H_2O (v/v 2:1:2) (3 mL) were placed in pressure vial equipped with a magnetic stirring bar. The mixture was stirred for 5 minutes with a continuous flow of argon. Then, $Pd(PPh_3)_4$ (7 mg, 2.5 %mol) was added and the mixture was stirred for 5 minutes in argon atmosphere. Then, MW irradiation (with initial 150 W power) was applied. The following conditions were used: 25 min, 80 °C. The reaction mixture was poured into water (5 ml) and the product was extracted with chloroform (5x5 ml, pH 9.) The combined organic phase was dried over $MgSO_4$. The appropriate product was purified by flash chromatography using dichloromethane: acetone (100: 0 => 40:60).

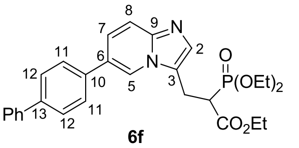

6f

Ethyl 3-(6-([1,1'-biphenyl]-4-yl)imidazo[1,2-*a*]pyridin-3-yl)-2 (diethoxyphosphoryl)propanoate (6f): Scale of reaction: compound **6m** 100 mg. Yield 83%. **^{1}H NMR** (700 MHz, $CDCl_3$) δ 1.19 (t, $^3J_{HH}$ = 7.10, C$\underline{H}_3CH_2$OC, 3H), 1.34 (bt, $^3J_{HH}$ = 7.10, C$\underline{H}_3CH_2$OP, 6H), 3.35-3.48 (m, CH_2C$\underline{H}$P, C$\underline{H}_2$CHP, 2H), 3.67 (ddd, $^2J_{HH}$ = 16.3, $^3J_{HH}$ = 11.5, $^3J_{PH}$ = 7.2, C$\underline{H}_2$CHP, 1H), 4.09-4.25 (m, C$\underline{H}_2$OC, C$\underline{H}_2$OP, 6H), 7.33-7.38 (m, CH_{Ar}, 1H), 7.42-7.46 (m, $CH_{Ar(2)}$, CH_{Ar}, 3H), 7.47 (dd, $^3J_{HH}$ = 9.3, $^4J_{HH}$ = 1.8, $CH_{Ar(7)}$, 1H), 7.60-7.65 (m, CH_{Ar}, 2H), 7.66 (dd, $^3J_{HH}$ = 9.3, $^5J_{HH}$ = 1.0, $CH_{Ar(8)}$, 1H), 7.65-7.72 (m, CH_{Ar}, 4H), 8.25 (dd, $^4J_{HH}$ = 1.8, $^5J_{HH}$ = 1.0, $CH_{Ar(5)}$, 1H), **^{31}P NMR** (700 MHz, $CDCl_3$) δ 20.95, **^{13}C NMR** (700 MHz, $CDCl_3$) δ 14.1 (s, $\underline{C}H_3CH_2$OC, 1C), 16.4 (d,$^3J_{PC}$ = 6.0, $\underline{C}H_3CH_2$OP, 1C), 16.5 (d, $^3J_{PC}$ = 5.9,

$\underline{C}H_3CH_2OP$, 1C), 21.8 (d, $^2J_{PC}$ = 3.3, $\underline{C}H_2CHP$, 1C), 44.5 (d, $^1J_{PC}$ = 129.6, $\underline{C}HP$, 1C), 62.0 (s, $\underline{C}H_2OC$, 1C), 63.2 (d, $^2J_{PC}$ = 7.0, $\underline{C}H_2OP$, 1C), 63.3 (d, $^2J_{PC}$ = 6.4, $\underline{C}H_2OP$, 1C), 117.9 (s, $CH_{Ar(8)}$, 1C), 120.4 (s, $\underline{C}H_{Ar(5)}$, 1C), 121.7 (d, $^3J_{PC}$ = 18.1, $\underline{C}_{Ar(3)}$, 1C), 124.4 (s, $\underline{C}H_{Ar(7)}$, 1C), 126.4 (s, $\underline{C}_{Ar(6)}$, 1C), 127.1, (s, $\underline{C}H_{Ar}$, 2C), 127.4 (s, $CH_{Ar,}$ 2C), 127.6 (s, CH_{Ar}, 1C), 127.9 (s, CH_{Ar}, 2C), 128.8 (s, CH_{Ar}, 2C), 132.3 (s, $CH_{Ar(2)}$, 1C), 136.3 (s, C_{Ar}, 1C), 140.4 (s, C_{Ar}, 1C), 140.8 (s, C_{Ar}, 1C), 144.1 (s, $C_{Ar(9)}$, 1C),168.4 (d, $^2J_{PC}$ = 4.9, $\underline{C}O_2$, 1C).

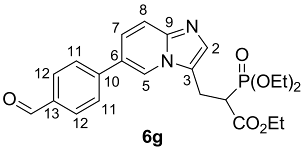

6g

Ethyl 2-(diethoxyphosphoryl)-3-(6-(4-formylphenyl)imidazo[1,2-*a*]pyridin-3-yl)propanoate (6g): Scale of reaction: compound **6m** 400 mg. Yield 77%. **HRMS m/z**: calculated 477.1949 ($C_{23}H_{27}N_2O_6P + CH_3OH)^+$, found 477.1597. **^{1}H NMR** (700 MHz, $CDCl_3$) δ 1.19 (t, $^3J_{HH}$ = 7.10, $C\underline{H_3}CH_2OC$, 3H), 1.34 (t, $^3J_{HH}$ = 7.1, $C\underline{H_3}CH_2OP$, 3H), 1.35 (t, $^3J_{HH}$ = 7.10, $C\underline{H_3}CH_2OP$, 3H), 3.38 (ddd, $^2J_{PH}$ = 23.0, $^3J_{HH}$ = 11.5, $^3J_{HH}$ = 3.3, $C\underline{H}P$, 1H), 3.44 (ddd, $^2J_{HH}$ = 15.8, $^3J_{PH}$ = 10.6, $^3J_{HH}$ = 3.3, $C\underline{H_2}CHP$, 1H), 3.67 (ddd, $^2J_{HH}$ = 15.8, $^3J_{HH}$ = 11.5, $^3J_{PH}$ = 7.1, $C\underline{H_2}CHP$, 1H), 4.10 - 4.23 (m, $C\underline{H_2}OC$, $C\underline{H_2}OP$, 6H), 7.47 (dd, $^3J_{HH}$ = 9.3, $^4J_{HH}$ = 1.8, $CH_{Ar(7)}$, 1H), 7.48 (s, $CH_{Ar(2)}$, 1H), 7.70 (dd, $^3J_{HH}$ = 9.3, $^5J_{HH}$ = 0.8, $CH_{Ar(8)}$, 1H), 7.76-7.79 (m, $C\underline{H}_{Ar}$, 2H), 7.97-8.00 (m, $C\underline{H}_{Ar}$, 2H), 8.33 (dd, $^4J_{HH}$ = 1.8, $^5J_{HH}$ = 0.8, $CH_{Ar(5)}$, 1H), 10.06 (s, $C\underline{H}O$, 1H). **^{31}P NMR** (283 MHz, $CDCl_3$) δ 20.85, **^{13}C NMR** (176 MHz, $CDCl_3$) δ 14.1 (s, $\underline{C}H_3CH_2OC$, 1C), 16.47 (d, $^3J_{PC}$ = 8.3 $\underline{C}H_3CH_2OP$, 1C), 16.50 (d, $^3J_{PC}$ = 8.6 $\underline{C}H_3CH_2OP$, 1C), 21.7 (d, $^2J_{PC}$ = 3.6, $\underline{C}H_2CHP$, 1C), 44.6 (d, $^1J_{PC}$ = 129.7, $\underline{C}HP$, 1C), 62.1 (s, $\underline{C}H_2OC$, 1C), 63.3 (d, $^2J_{PC}$ = 7.5, $\underline{C}H_2OP$, 1C), 63.4 (d, $^2J_{PC}$ = 6.5, $\underline{C}H_2OP$, 1C), 118.3 (s, $CH_{Ar(8)}$, 1C), 121.5 (s, $CH_{Ar(5)}$, 1C), 122.1 (d, $^3J_{PC}$ = 18.1, $C_{Ar(3)}$, 1C), 124.1 (s, $CH_{Ar(7)}$, 1C), 125.6 (s, $C_{Ar(6)}$, 1C), 127.6 (s, $CH_{Ar(11)}$, 2C), 130.6 (s, $CH_{Ar(12)}$, 2C), 132.6 (s, $CH_{Ar(2)}$, 1C), 135.8 (s, $C_{Ar(13)}$, 1C), 143.5 (s, $CH_{Ar(10)}$, 1C), 145.1 (s, $C_{Ar(9)}$, 1C), 168.4 (d, $^2J_{PC}$ = 5.1, $\underline{C}O_2$, 1C), 191.7 (s, $\underline{C}HO$, 1C).

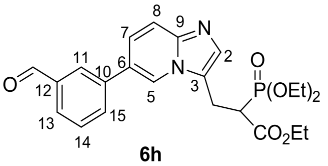

6h

Ethyl 2-(diethoxyphosphoryl)-3-(6-(3-formylphenyl)imidazo[1,2-*a*]pyridin-3-yl)propanoate (6h): Scale of reaction: compound **6m** 100 mg. Yield 76%. **^{1}H NMR** (700 MHz, $CDCl_3$) δ 1.19 (t, $^3J_{HH}$ = 7.10, $C\underline{H_3}CH_2OC$, 3H), 1.34 (t, $^3J_{HH}$ = 7.10, $C\underline{H_3}CH_2OP$, 6H), 3.39 (ddd, $^2J_{PH}$ = 23.0, $^3J_{HH}$ = 11.1, $^3J_{HH}$ = 3.3, $CH_2C\underline{H}P$, 1H), 3.44 (ddd, $^2J_{HH}$ = 15.6, $^3J_{PH}$ = 10.1, $^3J_{HH}$ = 3.3, $C\underline{H_2}CHP$, 1H), 3.66 (ddd, $^2J_{HH}$ = 15.6, $^3J_{HH}$ = 11.1, $^3J_{PH}$ = 7.1, $C\underline{H_2}CHP$, 1H), 4.10-4.23 (m, $C\underline{H_2}OC$, $C\underline{H_2}OP$, 6H), 7.46 (dd, $^3J_{HH}$ = 9.3, $^4J_{HH}$ = 1.8, $CH_{Ar(7)}$, 1H), 7.47 (s, $CH_{Ar(2)}$, 1H), 7.66 (t, $^3J_{HH}$ = 7.6, $CH_{Ar(14)}$, 1H), 7.68 (dd, $^3J_{HH}$ = 9.3, $^5J_{HH}$ = 1.0, $CH_{Ar(8)}$, 1H), 7.87 (ddd, $^3J_{HH}$ = 7.6, $^4J_{HH}$ = 2.0, $^4J_{HH}$ = 1.1, $CH_{Ar(15)}$, 1H), 7.90 (ddd, $^3J_{HH}$ = 7.6, $^4J_{HH}$ = 1.1, $CH_{Ar(13)}$, 1H), 8.09-8.13 (m, $CH_{Ar(11)}$, 1H), 8.28 (dd, $^4J_{HH}$ = 1.8, $^5J_{HH}$ = 1.0, $CH_{Ar(5)}$, 1H), 10.10 (s, $C\underline{H}O$, 1H), **^{31}P NMR** (283 MHz, $CDCl_3$) δ 20.87. **^{13}C NMR** (176 MHz, $CDCl_3$) δ 14.1 (s, $\underline{C}H_3CH_2OC$, 1C), 16.5 (d, $^3J_{PC}$ = 6.3, $\underline{C}H_3CH_2OP$, 1C), 16.5 (d, $^3J_{PC}$ = 6.0, $\underline{C}H_3CH_2OP$, 1C), 21.8 (d, $^2J_{PC}$ = 3.6, $\underline{C}H_2CHP$, 1C), 44.4 (d, $^1J_{PC}$ = 129.7, $\underline{C}HP$, 1C), 62.1 (s, $\underline{C}H_2OC$, 1C), 63.3 (d, $^2J_{PC}$ = 6.9, $\underline{C}H_2OP$, 1C), 63.4 (d, $^2J_{PC}$ = 6.5, $\underline{C}H_2OP$, 1C), 118.2 (s, $CH_{Ar(8)}$, 1C), 121.0 (s, $CH_{Ar(5)}$, 1C), 122.0 (d, $^3J_{PC}$ = 18.3, $C_{Ar(3)}$, 1C), 124.2 (s, $CH_{Ar(7)}$, 1C), 125.6 (s, $C_{Ar(6)}$, 1C), 127.9 (s, $CH_{Ar(11)}$, 1C), 129.5 (s, $CH_{Ar(13)}$, 1C), 130.0 (s, $CH_{Ar(14)}$, 1C), 132.6 (s, $CH_{Ar(2)}$, 1C), 132.9 (s, $CH_{Ar(15)}$, 1C), 137.3 (s, $C_{Ar(12)}$, 1C), 138.6 (s, $C_{Ar(10)}$, 145.1 (s, $C_{Ar(9)}$, 1C), 168.4 (d, $^2J_{PC}$ = 5.1, $\underline{C}O_2Et$, 1C), 192.0 (s, $\underline{C}HO$, 1C).

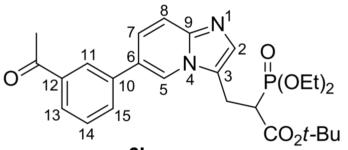

6i

***Tert*-butyl 3-(6-(3-acetylphenyl)imidazo[1,2-*a*]pyridin-3-yl)-2-(diethoxyphosphoryl)propanoate (6i):** Scale of reaction: compound **6m** 370 mg. Yield 67%. **^{1}H NMR** (700 MHz, $CDCl_3$) δ 1.36 (t, $^{3}J_{HH}$ = 7.10, $C\underline{H_3}CH_2OP$, 6H), 1.38 (s, $(C\underline{H_3})_3C$, 9H), 2.68 (s, $C(O)C\underline{H_3}$, 3H), 3.29 (ddd, $^{2}J_{PH}$ = 22.8, $^{3}J_{HH}$ = 11.3, $^{3}J_{HH}$ = 3.0, $C\underline{H}P$, 1H), 3.40 (ddd, $^{2}J_{HH}$ = 15.7, $^{3}J_{PH}$ = 10.3, $^{3}J_{HH}$ = 3.0, $C\underline{H_2}CHP$, 1H), 3.62 (ddd, $^{2}J_{HH}$ = 15.5, $^{3}J_{HH}$ = 11.3, $^{3}J_{PH}$ = 6.8, $C\underline{H_2}CHP$, 1H), 4.12-4.25 (m, $C\underline{H_2}OP$, 4H), 7.45 (dd, $^{3}J_{HH}$ = 9.3, $^{4}J_{HH}$ = 1.8, $CH_{Ar(7)}$, 1H), 7.49 (s, $CH_{Ar(2)}$, 1H), 7.59 (t, $^{3}J_{HH}$ = 7.7, $CH_{Ar(14)}$, 1H), 7.68 (dd, $^{3}J_{HH}$ = 9.3, $^{5}J_{HH}$ = 1.0, $CH_{Ar(8)}$, 1H), 7.81 (ddd, $^{3}J_{HH}$ = 7.7, $^{4}J_{HH}$ = 2.0, $^{4}J_{HH}$ = 1.1, $CH_{Ar(15)}$, 1H), 7.97 (dt, $^{3}J_{HH}$ = 7.8, $^{4}J_{HH}$ = 1.4, $CH_{Ar(13)}$, 1H), 8.20 (bt, $^{4}J_{HH}$ = 1.8, $CH_{Ar(11)}$, 1H), 8.28 (bs, $CH_{Ar(5)}$, 1H). **^{31}P NMR** (283 MHz, $CDCl_3$) δ 21.58. **^{13}C NMR** (176 MHz, $CDCl_3$) δ 16.55 & 16.57 (2d, $^{3}J_{PC}$ = 5.9, $\underline{C}H_3CH_2OP$, 2C), 21.8 (d, $^{2}J_{PC}$ = 3.4, $\underline{C}H_2CHP$, 1C), 26.9 (s, $C(O)\underline{C}H_3$, 1C), 27.9 (s, $(\underline{C}H_3)_3C$, 3C), 45.5 (d, $^{1}J_{PC}$ = 129.3, $\underline{C}HP$, 1C), 63.1 (d, $^{2}J_{PC}$ = 7.0, $\underline{C}H_2OP$, 1C), 63.2 (d, $^{2}J_{PC}$ = 6.4, $\underline{C}H_2OP$, 1C), 82.8 (s, $(CH_3)_3\underline{C}$, 1C), 118.2 (s, $CH_{Ar(8)}$, 1C), 121.1 (s, $CH_{Ar(5)}$, 1C), 122.2 (d, $^{3}J_{PC}$ = 19.1, $C_{Ar(3)}$, 1C), 124.3 (s, $CH_{Ar(7)}$, 1C), 125.9 (s, $C_{Ar(6)}$, 1C), 126.9 (s, $CH_{Ar(11)}$, 1C), 127.9 (s, $CH_{Ar(13)}$, 1C), 129.6 (s, $CH_{Ar(14)}$, 1C), 131.7 (s, $CH_{Ar(15)}$, 1C), 132.5 (s, $CH_{Ar(2)}$, 1C), 138.1 (s, $C_{Ar(12)}$, 1C), 138.3 (s, $C_{Ar(10)}$, 1C), 145.1 (s, $C_{Ar(9)}$, 1C), 167.5 (d, $^{2}J_{PC}$ = 5.1, $\underline{C}O_2$, 1C), 197.9 (s, $\underline{C}(O)CH_3$, 1C).

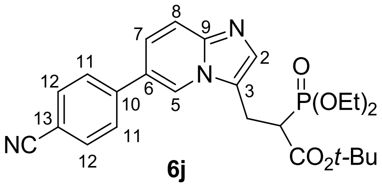

6j

***Tert*-butyl 3-(6-(4-cyanophenyl)imidazo[1,2-*a*]pyridin-3-yl)-2-(diethoxyphosphoryl)propanoate (6j):** Scale of reaction: compound **6m** 100 mg. Yield 64%. **^{1}H NMR** (700 MHz, $CDCl_3$) δ 1.34 (t, $^{3}J_{HH}$ = 6.5, $C\underline{H_3}CH_2OP$, 6H), 1.35 (t, $^{3}J_{HH}$ = 6.8, $C\underline{H_3}CH_2OP$, 6H), 1.36 (s, $(C\underline{H_3})_3C$, 9H), 3.26 (ddd, $^{2}J_{PH}$ = 22.8, $^{3}J_{HH}$ = 11.3, $^{3}J_{HH}$ = 3.1, $CH_2C\underline{H}P$, 1H), 3.39 (ddd, $^{2}J_{HH}$ = 15.8, $^{3}J_{PH}$ = 10.4, $^{3}J_{HH}$ = 3.1, $C\underline{H_2}CHP$, 1H), 3.61 (ddd, $^{2}J_{HH}$ = 15.9, $^{3}J_{HH}$ = 11.3, $^{3}J_{PH}$ = 6.9, $C\underline{H_2}CHP$, 1H), 4.16-4.23 (m, $C\underline{H_2}OP$, 4H), 7.40 (d, $^{3}J_{HH}$ = 9.3, J_{HH} = 1.8, CH_{Ar}, 1H), 7.49 (s, CH_{Ar}, 1H), 7.67 (ddd, $^{3}J_{HH}$ = 9.3, J_{HH} = 1.0, CH_{Ar}, 1H), 7.73 – 7.70 (m, CH_{Ar}, 2H), 7.77 – 7.74 (m, CH_{Ar}, 2H), 8.31 (dd, J_{HH} = 1.8, J_{HH} = 1.0, CH_{Ar}, 1H). **^{31}P NMR** (283 MHz, $CDCl_3$) δ 21.54.

2.4. General procedure for Heck reaction - compounds 6k, 5l [3]

$Pd(OAc)_2$ (2.45 mg, 0.0108 mmol, 0.05 equiv) and tri(*o*-tolyl)phosphine (2.98 mg, 0.0098 mmol, 0.045 equiv) were added to a solution of compound **6m** or **5m** (0.2177 mmol, 1.0 equiv), DIPEA (0.057 mL, 0.327 mmol, 1.5 equiv), benzyl acrylate (0.036 mL, 0.24 mmol, 1.1 equiv) in PCN (2 mL) and placed in pressure vial equipped with a magnetic stirring bar. The mixture was stirred for 1 min and purged with argon via a syringe. Then, MW irradiation (with initial 150 W power) was applied. The following conditions were used: 30 min, 110 °C. Then, the reaction mixture was diluted with DCM (10 mL), and adsorbed on silica gel (≈ 3 g). The solvent was evaporated, and the residue was subjected to column chromatography using gradient DCM:acetone (gradient 100:0 => 40:60) as eluent to give the product as orange oil.

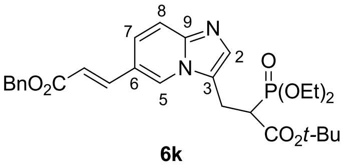

6k

(*E*)-benzyl 3-(3-(3-(tert-butoxy)-2-(diethoxyphosphoryl)-3-oxopropyl)imidazo[1,2-*a*]pyridin-6-yl)acrylate (6k): obtained using benzyl acrylate. Scale of reaction: compound **6m** 460 mg. Yield 63%, **^{1}H NMR** (700 MHz, $CDCl_3$) δ 1.31-1.37 (m, $POCH_2C\underline{H}_3$, $C(C\underline{H}_3)_3$, 15H), 3.23 (ddd, $^2J_{PH}$ = 22.7, $^3J_{HH}$ = 11.5, $^3J_{HH}$ = 3.1, $CH_2C\underline{H}P$, 1H), 3.31 (ddd, $^2J_{HH}$ = 15.5, $^3J_{PH}$ = 10.0, $^3J_{HH}$ = 3.1, $C\underline{H}_2CHP$, 1H), 3.66 (ddd, $^2J_{HH}$ = 15.5, $^3J_{HH}$ = 11.5, $^3J_{PH}$ = 7.0, $C\underline{H}_2CHP$, 1H), 4.07 – 4.26 (m, $POC\underline{H}_2$, 4H), 5.23 (s, $PhC\underline{H}_2$, 2H), 6.46 (d, $^3J_{HH}$ = 16.0, $C\underline{H}CO_2$, 1H), 7.29-7.46 (m, $CH_2C_6\underline{H}_5$, $C\underline{H}_{Ar(2,7)}$, 7H), 7.55 (dd, $^3J_{HH}$ = 9.5, $^4J_{HH}$ = 0.9, $C\underline{H}_{Ar\ 8)}$, 1H), 7.69 (d, $^3J_{HH}$ = 16.0, $C\underline{H}$=$CHCO_2$, 1H), 8.13 (bs, $C\underline{H}_{Ar(5)}$, 1H), **^{31}P NMR** (283 MHz, $CDCl_3$) δ 21.65. **^{13}C NMR** (176 MHz, $CDCl_3$) δ 16.5 (d, $^3J_{PC}$ = 4.2, $POCH_2\underline{C}H_3$, 1C), 16.5 (d, $^3J_{PC}$ =4.1, $POCH_2\underline{C}H_3$, 1C), 21.5 (d, $^2J_{PC}$ = 3.5, $\underline{C}H_2CHP$, 1C), 27.8 (s, $C(\underline{C}H_3)_3$, 3C), 45.3 (d, $^1J_{PC}$ = 129.4, $\underline{C}HP$, 1C), 63.0 (d, $^2J_{PC}$ = 7.0, $PO\underline{C}H_2$, 1C), 63.2 (d, $^2J_{PC}$ = 6.4, $PO\underline{C}H_2$, 1C), 66.6 (s, $\underline{C}H_2Ph$, 1C), 82.8 (s, $\underline{C}(CH_3)_3$, 1C), 118.3 (s, $\underline{C}H_{(8)}$, 1C), 118.4 (s, $\underline{C}HCO_2$, 1C), 120.7 (s, $\underline{C}_{Ar(6)}$, 1C), 121.1 (s, $\underline{C}H_{Ar(7)}$, 1C), 122.6 (d, $^3J_{PC}$ = 19.0, $\underline{C}_{Ar(3)}$, 1C), 125.6 (s, $\underline{C}H_{Ar(5)}$, 1C), 128.4 (s, $\underline{C}H_{Bn}$, 3C), 128.7 (s, $\underline{C}H_{Bn}$, 2C), 132.7 (s, $\underline{C}H_{Ar(2)}$, 1C), 136.0 (s, $\underline{C}_{Bn}$, 1C), 141.2 (s, $\underline{C}H$=$CHCO_2$, 1C), 145.4 (s, $C_{Ar(9)}$, 1C), 166.3 (s, $\underline{C}O_2Bn$, 1C), 167.3 (d, $^2J_{PC}$ = 5.2, $\underline{C}O_2C(CH_3)_3$, 1C).

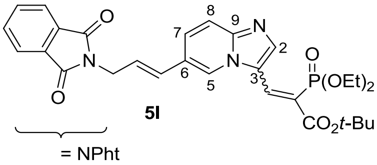

5l

***Tert*-butyl 2-(diethoxyphosphoryl)-3-(6-((*E*)-3-(1,3-dioxoisoindolin-2-yl)prop-1-en-1-yl)imidazo[1,2-*a*]pyridin-3-yl)acrylate (5l):** obtained using 2-allylisoindoline-1,3-dione. Scale of reaction: compound **5m** 300 mg . Yield: 65% (84% purity according to ^{31}P NMR spectrum). **^{1}H NMR** (700 MHz, $CDCl_3$) δ 8.40 (s, $C\underline{H}_{Ar(2)}$, 1H), 8.18 (s, $C\underline{H}_{Ar(5)}$, 1H), 7.88 – 7.85 (m, $C\underline{H}_{Pht}$, 2H), 7.76 (d, $^3J_{PH}$ = 23.9, $PCC\underline{H}$, 1H), 7.74 – 7.71 (m, $C\underline{H}_{Pht}$, 2H), 7.59 (dd, $^3J_{HH}$ = 9.3, $C\underline{H}_{Ar(7)}$, 1H), 7.46 (dd, $^3J_{HH}$ = 9.4, $^4J_{HH}$=1.5, $C\underline{H}_{Ar(8)}$, 1H), 6.55 (d, $^3J_{HH}$ = 15.9, $C\underline{H}CHCH_2NPht$, 1H), 6.31 (dt, $^3J_{HH}$ = 15.8, $^3J_{HH}$ = 5.9, $C\underline{H}CH_2NPht$, 1H), 4.47 (dd, $^3J_{HH}$ = 5.9, $^4J_{HH}$ = 1.3, $C\underline{H}_2NPht$, 2H), 4.17 – 4.09 (m, $C\underline{H}_2OP$, 4H), 1.54 (s, $C(C\underline{H}_3)_3$, 9H), 1.34 (t, $^3J_{HH}$ = 7.1, $C\underline{H}_3CH_2OP$, 6H). **^{13}C NMR** (176 MHz, $CDCl_3$) δ 167.94 (s, $\underline{C}ON$, 2C), 165.09 (d, $^2J_{PC}$ = 11.1, $\underline{C}O_2$, 1C), 147.1 (s, $\underline{C}_{Ar(9)}$, 1C), 140.8 (s, $\underline{C}H_{Ar(2)}$, 1C), 134.2 (s, $\underline{C}H_{Pht}$, 2C), 132.2 (s, $\underline{C}CH_{Pht}$, 2C), 132.1 (d, $^2J_{PC}$ = 10.1, $PC\underline{C}H$, 1C), 128.1 (s, $C_{Ar(6)}\underline{C}H$, 1C), 125.3 (s, $\underline{C}HCH_2NPht$, 1C), 124.8 (s, $\underline{C}H_{Ar(8)}$, 1C), 124.2 (s, $\underline{C}_{Ar(6)}$, 1C), 123.6 (s, $\underline{C}H_{Pht}$, 2C), 122.1 (s, $\underline{C}H_{Ar(5)}$, 1C), 120.7 (d,

$^3J_{PC}$ = 24.8, $\underline{C}_{Ar(3)}$, 1C), 118.4 (d, $^1J_{PC}$ = 182.0, P$\underline{C}$, 1C), 118.2 (s, $\underline{C}H_{Ar(7)}$, 1C), 82.7 (s, $CO_2\underline{C}Me_3$, 1C), 62.5 (d, $^2J_{PC}$ = 4.9, $\underline{C}H_2OP$, 2C), 39.3 (s, $\underline{C}H_2NPht$, 1C), 28.1 (s, $C(\underline{C}H_3)_3$, 3C), 16.4 (d, $^3J_{PC}$ = 6.9, $\underline{C}H_3CH_2OP$, 2C). **^{31}P NMR** (101 MHz, $CDCl_3$) δ 16.69. **HRMS** ($C_{29}H_{32}N_3O_7P + H^+$) m/z: calculated 566.2051, found 566.2061.

3.5. General procedure for the fluorination - compounds 7a-l

Compound **6a-l** (0.84 mmol, 1 equiv.) was added under argon atmosphere to a cooled (-30°C) suspension of NaH (50 mg, 1.25 mmol, 1.5 equiv., 60% suspension in oil) in THF (6 mL, 1.5 mL/100 mg of substrate). It was stirred for 50 min at -10 to +20 °C and then cooled to -60 °C followed by the addition of NFSI (315 mg, 1 mmol, 1.2 equiv.) in THF (4 mL). It was stirred for 20 min at -60 °C and for 20 h at 25-30 °C temperature. Depending on the analog, a precipitate should form after a couple of hours. The next stage is the addition of a small amount of chloroform (1 mL) and filtration of the resulting precipitate under reduced pressure. The sediment should also be flushed with chloroform. Next 10 mL of water was added. Solution was made basic with saturated Na_2CO_3 solution to pH>9. The water layer was extracted with chloroform (4 x 10 mL). Organic layer was dried over anhydrous $MgSO_4$ solvent was evaporated and residue was subjected to column chromatography using DCM: acetone (100:20 => 10:90) system as eluent to give product as orange oil.

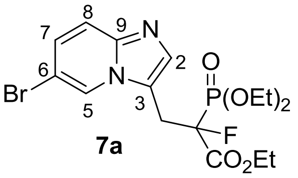

7a

Ethyl 3-(6-Bromoimidazo[1,2-*a*]pyridin-3-yl)-2-(diethoxyphosphoryl)-2-fluoropropanoate (7a): obtained with the yield 44%, according to the procedure published earlier.[2] Structure was confirmed by the spectral data.

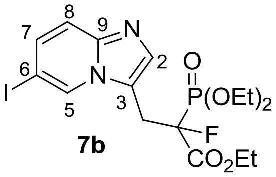

7b

Ethyl 3-(6-iodoimidazo[1,2-*a*]pyridin-3-yl)-2-(diethoxyphosphoryl)-2-fluoropropanoate (7b): Scale of reaction: compound **6b** 290 mg. Yield 67%. **^{1}H NMR** (700 MHz, $CDCl_3$) δ 1.21 (t, $^3J_{HH}$ = 7.1, $CO_2CH_2C\underline{H_3}$, 3H), 1.359 (t, $^3J_{HH}$ = 7.1, $C\underline{H_3}CH_2OP$, 3H), 1.361 (t, $^3J_{HH}$ = 7.1, $C\underline{H_3}CH_2OP$, 3H), 3.69 (ddd, $^2J_{HH}$ = 16.1, $^3J_{FH}$ = 12.5, $^3J_{PH}$ = 6.7, $C\underline{H_2}CFP$, 1H), 3.84 (ddd, $^3J_{FH}$ = 37.0, $^2J_{HH}$ = 16.2, $^3J_{PH}$ = 6.0, $C\underline{H_2}CHP$, 1H), 4.19-4.31 (m, $C\underline{H_2}OP$, $CO_2C\underline{H_2}$, 6H), 7.33 (dd, $^3J_{HH}$ = 9.4, $^4J_{HH}$ = 1.6, $CH_{Ar(7)}$, 1H), 7.37 (dd, $^3J_{HH}$ = 9.4, $^5J_{HH}$ = 0.9, $CH_{Ar(8)}$, 1H), 7.43 (bs, $CH_{Ar(2)}$, 1H), 8.31 (bq, $^4J_{HH}$ = 1.4, $CH_{Ar(5)}$, 1H). **^{31}P NMR** (283 MHz, $CDCl_3$) δ 11.26 (d, $^2J_{PF}$ = 81.9). **^{13}C NMR** (176 MHz, $CDCl_3$) δ 14.1 (s, $CO_2CH_2\underline{C}H_3$, 1C), 16.4-16.5 (m, $\underline{C}H_3CH_2OP$, 2C), 28.5 (dd, $^2J_{FC}$ = 20.8, $^2J_{PC}$ = 2.3, $\underline{C}H_2CFP$, 1C), 63.0 (s, $CO_2\underline{C}H_2$, 1C), 64.7 (d, $^2J_{PC}$ = 7.0, $\underline{C}H_2OP$, 1C), 65.0 (d, $^2J_{PC}$ = 6.8, $\underline{C}H_2OP$, 1C), 75.6 (s, $C_{Ar(6)}$, 1C), 96.0 (dd, $^1J_{FC}$ = 199.6, $^1J_{PC}$ = 160.2, $\underline{C}FP$, 1C), 116.9 (d, $^3J_{PC}$ = 14.1, $CH_{Ar(3)}$, 1C), 118.8 (s, $CH_{Ar(8)}$, 1C), 129.0 (d, $^5J_{FC}$ = 5.0, $CH_{Ar(5)}$, 1C), 132.4 (s, $CH_{Ar(7)}$, 1C), 134.4 (s, $CH_{Ar(2)}$, 1C), 144.6 (s, $C_{Ar(9)}$, 1C), 166.2 (dd, $^2J_{FC}$ = 22.5, $^2J_{PC}$ = 4.1, $\underline{C}O_2$, 1C).

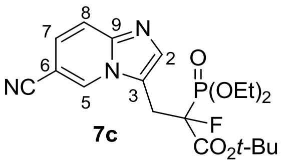

7c

***Tert*-butyl 3-(6-cyanoimidazo[1,2-*a*]pyridin-3-yl)-2-(diethoxyphosphoryl)-2-fluoropropanoate (7c):** Scale of reaction: compound **6c** 340 mg. Yield 90%. **^{1}H NMR** (700 MHz, $CDCl_3$) δ 1.34-1.39 (m, $(C\underline{H}_3)_3C$, $C\underline{H}_3CH_2OP$, 15H), 3.70 (ddd, $^{2}J_{HH}$ = 16.2, $^{3}J_{FH}$ = 12.2, $^{3}J_{PH}$ = 6.7, $C\underline{H}_2CFP$, 1H), 3.88 (ddd, $^{3}J_{FH}$ = 36.9, $^{2}J_{HH}$ = 16.2, $^{3}J_{PH}$ = 6.0, $C\underline{H}_2CFP$, 1H), 4.11 - 4.40 (m, $C\underline{H}_2OP$, 4H), 7.26 (dd, $^{3}J_{HH}$ = 9.3, $^{4}J_{HH}$ = 1.6, $CH_{Ar(7)}$, 1H), 7.63 (s, $CH_{Ar(2)}$, 1H), 7.64 (d, $^{3}J_{HH}$ = 9.3, $^{5}J_{HH}$ = 1.0 $CH_{Ar(8)}$, 1H), 8.59 (bs, $CH_{Ar(5)}$, 1H), **^{31}P NMR** (283 MHz, $CDCl_3$) δ 11.61 (d, $^{2}J_{PF}$ = 82.0), **^{13}C NMR** (176 MHz, $CDCl_3$) δ 16.52 (d, $^{3}J_{PC}$ = 5.6, $\underline{C}H_3CH_2OP$, 1C), 16.54 (d, $^{3}J_{PC}$ = 5.7, $\underline{C}H_3CH_2OP$, 1C), 27.9 (s, $C(\underline{C}H_3)_3$, 3C), 28.3 (d, $^{2}J_{FC}$ = 20.9, $^{2}J_{PC}$ = 2.8, $\underline{C}H_2CFP$, 1C), 64.6 (d, $^{2}J_{PC}$ = 7.0, $\underline{C}H_2OP$, 1C), 64.9 (d, $^{2}J_{PC}$ = 6.6, $\underline{C}H_2OP$, 1C), 85.0 (s, $\underline{C}(CH_3)_3$, 1C), 95.6 (dd, $^{1}J_{FC}$ = 199.6, $^{1}J_{PC}$ = 160.1, $CH_2\underline{C}FP$, 1C), 98.8 (s, $\underline{C}N$, 1C), 116.7 (s, $C_{Ar(6)}$, 1C), 118.8 (d, $^{3}J_{PC}$ = 14.6, $C_{Ar(3)}$, 1C), 118.9 (s, $CH_{Ar(8)}$, 1C), 124.0 (s, $CH_{Ar(7)}$, 1C), 130.7 (d, $^{5}J_{FC}$ = 5.8, $CH_{Ar(5)}$, 1C), 136.6 (s, $CH_{Ar(2)}$, 1C), 145.3 (s, $C_{Ar(9)}$, 1C), 164.9 (dd, $^{2}J_{FC}$ = 21.9, $^{2}J_{PC}$ = 3.6, $\underline{C}O_2$, 1C), **^{19}F NMR** (659 MHz, $CDCl_3$) δ -173.73 (ddd, $^{2}J_{PF}$ = 82.0, $^{3}J_{FH}$ = 36.9, $^{3}J_{FH}$ = 12.2, $CH_2C\underline{F}P$, 1F).

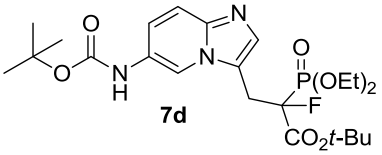

7d

***Tert*-butyl 3-(6-((*tert*-butoxycarbonyl)amino)imidazo[1,2-*a*]pyridin-3-yl)-2-(diethoxyphosphoryl)-2-fluoropropanoate (7d):** Scale of reaction: compound **6d** 190 mg. Yield 66%. **^{1}H NMR** (700 MHz, $CDCl_3$) δ 8.60 (bs, $C\underline{H}_{Ar}$, 1H), 7.53 (d, $^{3}J_{HH}$ = 10.4, $C\underline{H}_{Ar}$, 1H), 7.51 (s, $C\underline{H}_{Ar}$, 1H), 6.98 (dd, $^{3}J_{HH}$ = 9.6, $^{5}J_{HH}$ = 2.0, $C\underline{H}_{Ar}$, 1H), 6.40 (bs, $N\underline{H}$, 1H), 4.39 – 4.21 (m, $C\underline{H}_2OP$, 4H), 3.87 (ddd, $^{3}J_{FH}$ = 37.5, $^{2}J_{HH}$ = 16.1, $^{3}J_{PH}$= 5.1, $C\underline{H}_2CFP$, 1H), 3.67 (ddd, $^{2}J_{HH}$ = 16.0, $^{3}J_{FH}$ = 11.4, $^{3}J_{PH}$= 7.0, $C\underline{H}_2CFP$, 1H), 1.52 (s, $(C\underline{H}_3)_3COC(O)N$, 9H), 1.41 (s, $CCO_2(C\underline{H}_3)_3$, 9H), 1.40 (t, $^{3}J_{HH}$ = 6.8, $C\underline{H}_3CH_2OP$, 6H). **^{31}P NMR** (700 MHz, $CDCl_3$) δ 12.32 (d, $^{2}J_{PF}$ = 83.5).

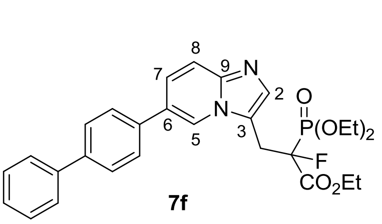

7f

Ethyl 3-(6-([1,1'-biphenyl]-4-yl)imidazo[1,2-*a*]pyridin-3-yl)-2-(diethoxyphosphoryl)-2-fluoropropanoate (7f): Scale of reaction: compound **6f** 83 mg. Yield 55%. **^{1}H NMR** (700 MHz, $CDCl_3$) δ 1.21 (t, $^{3}J_{HH}$ = 7.1, $CO_2CH_2C\underline{H}_3$, 3H), 1.34-1.41 (m, $POCH_2C\underline{H}_3$, 6H), 3.81 (ddd, $^{2}J_{HH}$ = 16.2, $^{3}J_{FH}$ = 12.6, $^{3}J_{PH}$ = 6.7, $C\underline{H}_2CFP$, 1H), 3.95 (ddd, $^{3}J_{FH}$ = 37.0, $^{2}J_{HH}$ = 16.2, $^{3}J_{PH}$ = 6.1, $C\underline{H}_2CFP$, 1H), 4.23 (q, $^{3}J_{HH}$ = 7.1, $CO_2C\underline{H}_2$, 2H), 4.26-4.31 (m, $POC\underline{H}_2$, 4H), 7.35-7.40 (m, CH_{Ar}, 1H), 7.44-7.51 (m, CH_{Ar}, 3H), 7.54 (s, $CH_{Ar(2)}$, 1H), 7.62-7.69 (m, CH_{Ar}, 5H), 7.69-7.73 (m, CH_{Ar}, 2H), 8.34 (bs, $CH_{Ar(5)}$, 1H). **^{31}P NMR** (284 MHz, $CDCl_3$) δ 11.51 (d, $^{2}J_{PF}$ = 81.3). **^{13}C NMR** (176 MHz, $CDCl_3$) δ 14.1 (s, $CO_2CH_2\underline{C}H_3$, 1C), 16.5 (d, $^{3}J_{PC}$ = 5.6, $\underline{C}H_3CH_2OP$, 2C), 28.7 (d, $^{2}J_{FC}$ = 21.0, $\underline{C}H_2CPF$, 1C), 63.0 (s, $CO_2\underline{C}H_2$, 1C), 64.6 (d, $^{2}J_{PC}$ = 7.0,

$\underline{C}H_2OP$, 1C), 64.9 (d, $^2J_{PC}$ = 6.7, $\underline{C}H_2OP$, 1C), 96.3 (dd, $^1J_{FC}$ = 200.2, $^1J_{PC}$ = 160.2, $\underline{C}PF$, 1C), 117.2 (d, $^3J_{PC}$ = 13.7, $C_{Ar(3)}$, 1C), 117.8 (s, $CH_{Ar(8)}$, 1C), 121.3 (d, $^5J_{FC}$ = 4.9, $CH_{Ar(5)}$, 1C), 124.9 (s, $CH_{Ar(7)}$, 1C), 126.4 (s, $C_{Ar(6)}$, 1C), 127.2 (s, $CH_{Ar,}$ 2C), 127.5 (s, $CH_{Ar,}$ 2C), 127.7 (s, $CH_{Ar,}$ 1C), 127.9 (s, $CH_{Ar,}$ 2C), 129.0 (s, $CH_{Ar,}$ 2C), 134.9 (s, $CH_{Ar(2)}$, 1C), 136.5 (s, $CH_{Ar,}$ 1C), 140.5 (s, $CH_{Ar,}$ 1C), 140.9 (s, $CH_{Ar,}$ 1C), 145.6 (s, C_{Ar}, 1C), 166.5 (dd, $^2J_{FC}$ = 22.3, $^2J_{PC}$ = 3.9, $\underline{C}O_2Et$, 1C),

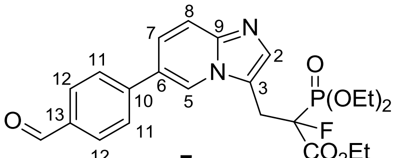

7g

Ethyl 2-(diethoxyphosphoryl)-2-fluoro-3-(6-(4-formylphenyl)imidazo[1,2-*a*]pyridin-3-yl)propanoate (7g): Scale of reaction: compound **6g** 50 mg. Yield 67%. **HRMS** $(C_{23}H_{26}FN_2O_6P+H)^+$ m/z: calculated 477.1586, found 477.1597. **^{1}H NMR** (700 MHz, $CDCl_3$) δ 1.20 (t, $^3J_{HH}$ = 7.1, $CO_2CH_2C\underline{H_3}$, 3H), 1.34 (t, $^3J_{HH}$ = 7.1, $POCH_2C\underline{H_3}$, 3H), 1.36 (t, $^3J_{HH}$ = 7.1, $POCH_2C\underline{H_3}$, 3H), 3.80 (ddd, $^2J_{HH}$ = 16.2, $^3J_{FH}$ = 12.8, $^3J_{PH}$ = 6.6, $C\underline{H_2}CFP$, 1H), 3.95 (ddd, $^3J_{FH}$ = 37.0, $^2J_{HH}$ = 16.2, $^3J_{PH}$ = 6.1, $C\underline{H_2}CFP$, 1H), 4.19-4.31 (m, $CO_2C\underline{H_2}$, $POC\underline{H_2}$, 6H), 7.46 (dd, $^3J_{HH}$ = 9.4, $^4J_{HH}$ = 1.8, $CH_{Ar(7)}$, 1H), 7.55 (s, $CH_{Ar(2)}$, 1H), 7.68 (dd, $^3J_{HH}$ = 9.4, $^5J_{HH}$ = 0.9, $CH_{Ar(8)}$, 1H), 7.73-7.77 (m, $CH_{Ar(11)}$, 2H), 7.95-8.00 (m, $CH_{Ar(12)}$, 2H), 8.37 (bs, $CH_{Ar(5)}$, 1H), 10.06 (s, $C\underline{H}O$, 1H). **^{31}P NMR** (284 MHz, $CDCl_3$) δ 11.38 (d, $^2J_{PF}$ = 81.4). **^{13}C NMR** (176 MHz, $CDCl_3$) δ 14.1 (s, $CO_2CH_2\underline{C}H_3$, 1C), 16.50 (d, $^3J_{PC}$ = 5.4, $POCH_2\underline{C}H_3$, 1C), 16.51 (d, $^3J_{PC}$ = 5.7, $POCH_2\underline{C}H_3$, 1C), 28.7 (bd, $^2J_{FC}$ = 20.6, $\underline{C}H_2CPF$, 1C), 63.0 (s, $CO_2\underline{C}H_2$, 1C), 64.7 (d, $^2J_{PC}$ = 7.0, $PO\underline{C}H_2$, 1C), 65.0 (d, $^2J_{PC}$ = 6.9, $PO\underline{C}H_2$, 1C), 96.3 (dd, $^1J_{FC}$ = 200, $^1J_{PC}$ = 160, $\underline{C}PF$, 1C), 117.6 (d, $^3J_{PC}$ = 13.8, $C_{Ar(3)}$, 1C), 118.2 (s, $CH_{Ar(8)}$, 1C), 122.3 (d, $^5J_{FC}$ = 5.1, $CH_{Ar(5)}$, 1C), 124.4 (s, $CH_{Ar(7)}$, 1C), 125.5 (s, $C_{Ar(6)}$, 1C), 127.7 (s, $CH_{Ar(11)}$, 2C), 130.6 (s, $CH_{Ar(12)}$, 2C), 135.3 (s, $CH_{Ar(2)}$, 1C), 135.8 (s, $C_{Ar(13)}$, 1C), 143.6 (s, $C_{Ar(10)}$, 1C), 145.7 (s, $C_{Ar(9)}$, 1C), 166.4 (dd, $^2J_{FC}$ = 22.5, $^2J_{PC}$ = 4.1, $\underline{C}O_2$, 1C), 191.7 (s, $\underline{C}HO$, 1C).

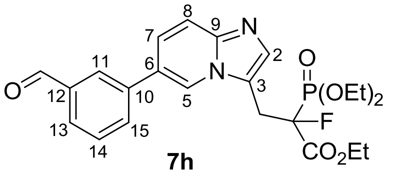

7h

Ethyl 2-(diethoxyphosphoryl)-2-fluoro-3-(6-(3-formylphenyl)imidazo[1,2-*a*]pyridin-3-yl)propanoate (7h): Scale of reaction: compound **6h** 100 mg. Yield 20%. **HRMS m/z**: calculated 477.1586 $(C_{23}H_{27}N_2O_6P + CH_3OH)^+$, found 477.1586, **^{1}H NMR** (700 MHz, $CDCl_3$) δ 1.21 (t, $^3J_{HH}$ = 7.1, $C\underline{H_3}CH_2OC$, 3H), 1.36 (t, $^3J_{HH}$ = 7.0, $C\underline{H_3}CH_2OP$, 3H), 1.37 (t, $^3J_{HH}$ = 7.1, $C\underline{H_3}CH_2OP$, 3H), 3.82 (ddd, $^2J_{HH}$ = 16.3, $^3J_{FH}$ = 12.7, $^3J_{PH}$ = 6.7, $C\underline{H_2}CFP$, 1H), 3.96 (ddd, $^3J_{FH}$ = 36.9, $^2J_{HH}$ = 16.3, $^3J_{PH}$ = 6.3, $C\underline{H_2}CFP$, 1H), 4.20-4.31 (m, $CO_2C\underline{H_2}$, $C\underline{H_2}OOP$, 6H), 7.46 (dd, $^3J_{HH}$ = 9.3, $^4J_{HH}$ = 1.8, $CH_{Ar(7)}$, 1H), 7.56 (s, $CH_{Ar(2)}$, 1H), 7.66 (t, $^3J_{HH}$ = 7.6, $CH_{Ar(14)}$, 1H), 7.69 (dd, $^3J_{HH}$ = 9.3, $^5J_{HH}$ = 1.0, $CH_{Ar(8)}$, 1H), 7.86 (ddd, $^3J_{HH}$ = 7.7, $^4J_{HH}$ = 2.0, $^4J_{HH}$ = 1.3, $CH_{Ar(15)}$, 1H), 7.91 (dt, $^3J_{HH}$ = 7.6, $^4J_{HH}$ = 1.3, $CH_{Ar(13)}$, 1H), 8.08-8.10 (m, $CH_{Ar(11)}$, 1H), 8.33-8.37 (m, $CH_{Ar(5)}$, 1H), 10.11 (s, $C\underline{H}O$, 1H), **^{31}P NMR** (283 MHz, $CDCl_3$) δ 11.40 (d, $^2J_{PF}$ = 81.9), **^{13}C NMR** (176 MHz, $CDCl_3$) δ 14.1 (s, $CO_2CH_2\underline{C}H_3$, 1C), 16.54 (bd, $^3J_{PC}$ = 5.6, $POCH_2\underline{C}H_3$, 2C), 28.7 (d, $^2J_{FC}$ = 20.4, $^2J_{PC}$ = 2.1, $\underline{C}H_2CFP$, 1C), 63.0 (s, $CO_2\underline{C}H_2$, 1C), 64.7 (d, $^2J_{PC}$ = 7.1, $PO\underline{C}H_2$, 1C), 65.0 (d, $^2J_{PC}$ = 6.8, $PO\underline{C}H_2$, 1C), 96.3 (dd, $^1J_{FC}$ = 199.8, $^1J_{PC}$ = 159.9, $\underline{C}PF$, 1C), 117.5 (d, $^3J_{PC}$ = 13.9, $C_{Ar(3)}$, 1C), 118.2 (s, $CH_{Ar(8)}$, 1C), 121.9 (d, $^5J_{FC}$ = 5.1, $CH_{Ar(5)}$, 1C), 124.6 (s, $CH_{Ar(7)}$, 1C), 125.6 (s, $C_{Ar(6)}$, 1C), 128.0 (s, $CH_{Ar(11)}$, 1C), 129.4 (s, $CH_{Ar(13)}$, 1C), 130.0 (s,

$CH_{Ar(14)}$, 1C), 133.1 (s, $CH_{Ar(15)}$, 1C), 135.2 (s, $CH_{Ar(2)}$, 1C), 137.3 (s, $C_{Ar(12)}$, 1C), 138.8 (s, $C_{Ar(10)}$, 1C), 145.7 (s, $C_{Ar(9)}$, 1C), 166.5 (dd, $^2J_{FC}$ = 22.3, $^2J_{PC}$ = 4.0, $\underline{C}O_2Et$, 1C), 192.0(s, $\underline{C}HO$, 1C).

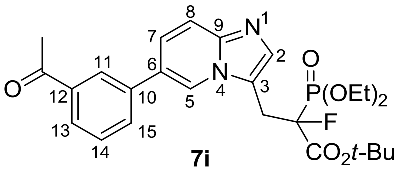

7i

***Tert*-butyl 3-(6-(3-acetylphenyl)imidazo[1,2-*a*]pyridin-3-yl)-2-(diethoxyphosphoryl)-2-fluoropropanoate (7i):** Scale of reaction: compound **6i** 270 mg. Yield 77%. **^{1}H NMR** (700 MHz, $CDCl_3$) δ 1.35 (t, $^3J_{HH}$ = 6.3, $C\underline{H}_3CH_2OP$, 1H), 1.368 (t, $^3J_{HH}$ = 5.5, $C\underline{H}_3CH_2OP$, 1H), 1.38 (s, $(C\underline{H}_3)_3C$, 9H), 2.66 (s, $C(O)C\underline{H}_3$, 3H), 3.75 (ddd, $^2J_{HH}$ = 16.2, $^3J_{FH}$ = 12.3, $^3J_{PH}$ = 6.6, $C\underline{H}_2CFP$, 1H), 3.92 (ddd, $^3J_{FH}$ = 37.1, $^2J_{HH}$ = 16.2, $^3J_{PH}$ = 6.0, $C\underline{H}_2CFP$, 1H) 4.21-4.31 (m, $C\underline{H}_2OP$, 4H), 7.44 (dd, $^3J_{HH}$ = 9.2, $^4J_{HH}$ = 1.8, $CH_{Ar(7)}$, 1H), 7.55-7.60 (m, $CH_{Ar(2)}$, $CH_{Ar(14)}$, 2H), 7.67 (d, $^3J_{HH}$ = 9.3, $CH_{Ar(8)}$, 1H), 7.78 (d, $^3J_{HH}$ = 7.7, $CH_{Ar(15)}$, 1H), 7.96 (bd, $^3J_{HH}$ = 7.9, $CH_{Ar(13)}$, 1H), 8.16 (bm, $CH_{Ar(11)}$, 1H), 8.34 (bs, $CH_{Ar(5)}$, 1H). **^{31}P NMR** (283 MHz, $CDCl_3$) δ 12.11 (d, $^2J_{PF}$ = 84). **^{13}C NMR** (176 MHz, $CDCl_3$) δ 16.5 (t, $^3J_{PC}$ = 5.1, $\underline{C}H_3CH_2OP$, 2C), 26.8 (s, $C(O)\underline{C}H_3$, 1C), 27.9 (s, $(\underline{C}H_3)_3C$, 3C), 28.6 (dd, $^2J_{FC}$ = 20.9, $^2J_{PC}$ = 2.7, $\underline{C}H_2CFP$, 1C), 64.5 (d, $^2J_{PC}$ = 7.0, $\underline{C}H_2OP$, 1C), 64.7 (d, $^2J_{PC}$ = 6.8, $\underline{C}H_2OP$, 1C), 84.7 (s, $(CH_3)_3\underline{C}$, 1C), 95.8 (dd, $^1J_{FC}$ = 199.8, $^1J_{PC}$ = 159.9, $\underline{C}FP$, 1C), 117.6 (d, $^3J_{PC}$ = 14.6, $C_{Ar(3)}$, 1C), 117.9 (s, $CH_{Ar(8)}$, 1C), 121.9 (d, $^5J_{FC}$ = 5.1, $CH_{Ar(5)}$, 1C), 124.7 (s, $CH_{Ar(7)}$, 1C), 125.9 (s, $C_{Ar(6)}$, 1C), 126.9 (s, $CH_{Ar(11)}$, 1C), 127.9 (s, $CH_{Ar(13)}$, 1C), 129.5 (s, $CH_{Ar(14)}$, 1C), 131.7 (s, $CH_{Ar(15)}$, 1C), 135.0 (s, $CH_{Ar(2)}$, 1C), 138.0 (s, $C_{Ar(12)}$, 1C), 138.3 (s, $C_{Ar(10)}$, 1C), 145.5 (s, $C_{Ar(9)}$, 1C), 165.2 (dd, $^2J_{FC}$ = 22.2, $^2J_{PC}$ = 3.8, $\underline{C}O_2{}^tBu$, 1C), 197.8 (s, $\underline{C}(O)CH_3$, 1C).

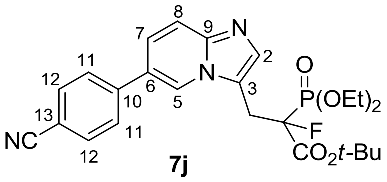

7j

***Tert*-butyl 3-(6-(4-cyanophenyl)imidazo[1,2-a]pyridin-3-yl)-2-(diethoxyphosphoryl)-2-fluoropropanoate (7j):** Scale of reaction: compound **6j** 300 mg. Yield 89%. **^{1}H NMR** (700 MHz, $CDCl_3$) δ 1.34 (t, $^3J_{HH}$ = 7.1, $POCH_2C\underline{H}_3$, 3H), 1.38 (t, $^3J_{HH}$ = 7.1, $POCH_2C\underline{H}_3$, 3H), 1.38 (s, $C(C\underline{H}_3)_3$, 9H), 3.75 (ddd, $^2J_{HH}$ = 16.2, $^3J_{FH}$ = 12.6, $^3J_{PH}$ = 6.5, $C\underline{H}_2CFP$, 1H), 3.92 (ddd, $^3J_{FH}$ = 37.0, $^2J_{HH}$ = 16.2, $^3J_{PH}$ = 6.2, $C\underline{H}_2CFP$, 1H), 4.20-4.31 (m, $POC\underline{H}_2$, 4H), 7.40 (dd, $^3J_{HH}$ = 9.3, $^4J_{HH}$ = 1.8, $CH_{Ar(7)}$, 1H), 7.58 (s, $CH_{Ar(2)}$, 1H), 7.68 (d, $^3J_{HH}$ = 9.3, $CH_{Ar(8)}$, 1H), 7.69 (d, $^3J_{HH}$ = 8.3, $CH_{Ar(11)}$, 2H), 7.76 (d, $^3J_{HH}$ = 8.1, $CH_{Ar(12)}$, 2H), 8.36 (bs, $CH_{Ar(5)}$, 1H). **^{31}P NMR** (284 MHz, $CDCl_3$) δ 12.06 (d, $^2J_{PF}$ = 83.2). **^{13}C NMR** (176 MHz, $CDCl_3$) δ 16.5 (d, $^3J_{PC}$ = 5.7, $POCH_2\underline{C}H_3$, 1C), 16.6 (d, $^3J_{PC}$ = 5.9, $POCH_2\underline{C}H_3$, 1C), 27.9 (s, $(\underline{C}H_3)_3C$, 3C), 28.6 (dd, $^2J_{FC}$ = 20.7, $^2J_{PC}$ = 2.6, $\underline{C}H_2CPF$, 1C), 64.5 (d, $^2J_{PC}$ = 7.0, $PO\underline{C}H_2$, 1C), 64.9 (d, $^2J_{PC}$ = 7.0, $PO\underline{C}H_2$, 1C), 84.8 (s, $(CH_3)_3\underline{C}$, 1C), 95.8 (dd, $^1J_{FC}$ = 199.6, $^1J_{PC}$ = 160.0, $\underline{C}PF$, 1C), 111.7 (s, $\underline{C}N$, 1C), 117.8 (d, $^3J_{PC}$ = 14.1, $C_{Ar(3)}$, 1C), 118.3 (s, $CH_{Ar(8)}$, 1C), 118.7 (s, $C_{Ar(13)}$, 1C), 122.5 (d, $^5J_{FC}$ = 5.6, $CH_{Ar(5)}$, 1C), 124.0 (s, $CH_{Ar(7)}$, 1C), 124.9 (s, $C_{Ar(6)}$, 1C), 127.8 (s, $CH_{Ar(11)}$, 2C), 133.0 (s, $CH_{Ar(12)}$, 2C), 135.5 (s, $CH_{Ar(2)}$, 1C), 142.2 (s, $C_{Ar(10)}$, 1C), 145.6 (s, $C_{Ar(9)}$, 1C), 165.1 (dd, $^2J_{FC}$ = 22.1, $^2J_{PC}$ = 3.7, $\underline{C}O_2$, 1C).

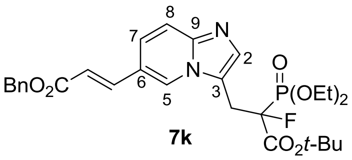

(*E*)-benzyl 3-(3-(3-(tert-butoxy)-2-(diethoxyphosphoryl)-2-fluoro-3-oxopropyl)imidazo[1,2-*a*]pyridin-6-yl)acrylate (7k): Scale of reaction: compound **6k** 500 mg. Yield 52%. **^{1}H NMR** (700 MHz, $CDCl_3$) δ 1.38 (s, $C(C\underline{H_3})_3$, 9H), 1.36-1.39 (m, $\underline{C}H_3CH_2OP$, 6H), 3.69 (ddd, $^{2}J_{HH}$ = 16.1, $^{3}J_{FH}$ = 12.3, $^{3}J_{PH}$ = 6.5, $C\underline{H_2}CFP$, 1H), 3.87 (ddd, $^{3}J_{FH}$ = 37.4, $^{2}J_{HH}$ = 16.1, $^{3}J_{PH}$ = 5.9, $C\underline{H_2}CFP$, 1H), 4.21-4.31 (m, $C\underline{H_2}OP$, 4H), 5.26 (s, $C\underline{H_2}Ph$, 2H), 6,47 (d, $^{3}J_{HH}$ = 15.9, $C\underline{H}CHCO_2Bn$, 1H), 7.31-7.45 (m, $C\underline{H}_{(Bn)}$, $CH_{Ar(7)}$, 6H), 7.53 (s, $CH_{Ar(2)}$, 1H), 7,57 (d, $^{3}J_{HH}$ = 9.4, $CH_{Ar(8)}$, 1H), 7.70(d, $^{3}J_{HH}$ = 15.9, $CH{=}C\underline{H}CO_2Bn$, 1H), 8.22 (bs, $CH_{Ar(5)}$, 1H), **^{31}P NMR** (700 MHz, $CDCl_3$) δ 12.00 (d, $^{2}J_{PF}$ = 83.1), **^{13}C NMR** (700 MHz, $CDCl_3$) δ 16.6 (d, $^{3}J_{PC}$ = 3.1, $\underline{C}H_3CH_2OP$, 1C), 16.5 (d, $^{2}J_{PC}$ = 3.1, $\underline{C}H_3CH_2OP$, 1C), 27.9 (s, $C(\underline{C}H_3)_3$, 3C), 28.4 (d, $^{2}J_{FC}$ = 16.6, $\underline{C}H_2CFP$, 1C), 64.5 (d, $^{2}J_{PC}$ = 7.2, $\underline{C}H_2OP$, 1C), 64.8 (d, $^{2}J_{PC}$ = 6.7, $\underline{C}H_2OP$, 1C), 66.7 (s, $\underline{C}H_2Ph$, 1C), 84.8 (s, $\underline{C}(CH_3)_3$, 1C) 95.8 (dd, $^{1}J_{FC}$ = 199.8, $^{1}J_{PC}$ = 159.0, $\underline{C}FP$, 1C), 118.2 (d, $^{3}J_{PC}$ = 14.5, $\underline{C}_{Ar(3)}$, 1C), 118.3 (s, $\underline{C}H_{Ar(8)}$, 1C), 118.5 (s, $\underline{C}HCHCO_2Bn$, 1C), 120.7 (s, $\underline{C}_{Ar(6)}$, 1C), 121.5 (s, $\underline{C}H_{Ar(7)}$, 1C), 126.2 (s, $\underline{C}H_{Ar(5)}$, 1C), 128.5 (s, $\underline{C}H_{(Bn)}$, 3C), 128.8 (s, $\underline{C}H_{(Bn)}$, 2C), 135.4 (d, $^{4}J_{FC}$ = 7.2, $\underline{C}H_{Ar(2)}$, 1C), 136.0 (s, $\underline{C}_{(Bn)}$, 1C), 141.4 (s, $\underline{C}HCO_2Bn$, 1C), 146.0 (s, $C_{Ar(9)}$, 1C), 165.0 (dd, $^{2}J_{FC}$ = 22.0, $^{2}J_{PC}$ = 3.6, $\underline{C}O_2C(CH_3)_3$, 1C), 166.5 (s, $\underline{C}O_2Bn$, 1C).

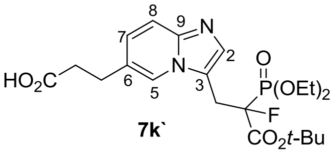

3-(3-(3-(tert-butoxy)-2-(diethoxyphosphoryl)-2-fluoro-3-oxopropyl)imidazo[1,2-*a*]pyridin-6-yl)propanoic acid (7k'): In a single-neck flask compound **7k** (150 mg) and 10% Pd/C (10mg) was placed in MeOH (10 mL). The system was degassed using a two-way stopcock. This suspension was stirred overnight at room temperature. The catalyst was then filtered off through a thin layer of Celite500, and the filtrate was evaporated to dryness, giving pure **5i'** with 85% yield. **HRMS m/z**: calculated 473.1847 $(C_{21}H_{30}FN_2O_7P + H)^{+}$, found 473.1850, **^{1}H NMR** (700 MHz, $CDCl_3$) δ 1.26-1.38 (m, $(C\underline{H_3})_3C$, $C\underline{H_3}CH_2OP$, 15H), 2.64 (t, $^{3}J_{HH}$ = 7.8, $C\underline{H_2}CO_2$, 2H), 2.96 (t, $^{3}J_{HH}$ = 7.8, $C\underline{H_2}CH_2CO_2$, 2H), 3.64 (ddd, $^{2}J_{HH}$ = 16.2, $^{3}J_{FH}$ = 11.9, $^{3}J_{PH}$ = 6.7, $C\underline{H_2}CFP$, 1H), 3.81 (ddd, $^{3}J_{FH}$ = 37.5, $^{2}J_{HH}$ = 16.2, $^{3}J_{PH}$ = 5.6, $C\underline{H_2}CFP$, 1H), 4.16 - 4.29 (m, $C\underline{H_2}OP$, 4H), 7.14 (dd, $^{3}J_{HH}$ = 9.2, $^{4}J_{HH}$ = 1.1, $CH_{Ar(7)}$, 1H), 7.45 (s, $CH_{Ar(2)}$, 1H), 7.58 (d, $^{3}J_{HH}$ = 9.2, $CH_{Ar(8)}$, 1H), 8.00 (bs, $CH_{Ar(5)}$, 1H), 10.87 (bs, $CO_2\underline{H}$, (15), 1H). **^{31}P NMR** (283 MHz, $CDCl_3$) δ 12.0 (d, $^{2}J_{PF}$ = 83.9). **^{13}C NMR** (176 MHz, $CDCl_3$) δ 16.4 (d, $^{3}J_{PC}$ = 5.5, $\underline{C}H_3CH_2OP$, 1C), 16.4 (d, $^{3}J_{PC}$ = 5.6, $\underline{C}H_3CH_2OP$, 1C), 27.7 (s, $(CO_2(\underline{C}H_3)_3$, 3C), 28.1 (d, $^{2}J_{FC}$ = 23.4, $\underline{C}H_2CFP$, 1C), 28.2 (s, $\underline{C}H_2CH_2CO_2$, 1C), 35.7 (s, $\underline{C}H_2CO_2$, 1C), 64.5 (d, $^{2}J_{PC}$ = 7.0, $\underline{C}H_2OP$, 1C), 64.8 (d, $^{2}J_{PC}$ = 6.5, $\underline{C}H_2OP$, 1C), 84.6 (s, $\underline{C}(CH_3)_3$, 1C), 95.5 (dd, $^{1}J_{FC}$ = 199.5, $^{1}J_{PC}$ = 160.2, $\underline{C}FP$, 1C), 116.6 (s, $CH_{Ar(8)}$, 1C), 116.9 (d, $^{3}J_{PC}$ = 15.0, $C_{Ar(3)}$, 1C), 122.1 (d, $^{5}J_{FC}$ = 4.2, $CH_{Ar(5)}$, 1C), 126.2 (s, $CH_{Ar(6)}$, 1C), 127.5 (s, $CH_{Ar(7)}$, 1C), 132.0 (s, $CH_{Ar(2)}$, 1C), 144.4 (s, $C_{Ar(9)}$, 1C), 164.9 (dd, $^{2}J_{FC}$ = 22.2, $^{2}J_{PC}$ = 3.5, $\underline{C}O_2C(CH_3)_3$, 1C), 175.2 (s, $\underline{C}O_2H$, 1C).

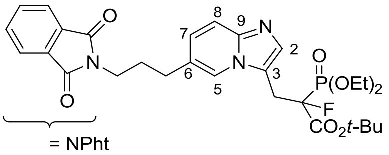

7l

***Tert*-butyl 2-(diethoxyphosphoryl)-3-(6-(3-(1,3-dioxoisoindolin-2-yl)propyl)imidazo[1,2-*a*]pyridin-3-yl)-2-fluoropropanoate (7l):** Scale of reaction: compound **6l** 125 mg. Yield 74%. **^{1}H NMR** (700 MHz, $CDCl_3$) δ 7.92 (s, $C\underline{H}_{Ar(5)}$, 1H), 7.79 – 7.76 (m, $C\underline{H}_{Pht}$, 2H), 7.68 – 7.64 (m, $C\underline{H}_{Pht}$, 2H), 7.48 (d, $^3J_{HH}$ = 9.2, $C\underline{H}_{Ar(8)}$, 1H), 7.45 (s, $C\underline{H}_{Ar(2)}$, 1H), 7.05 (dd, $^3J_{HH}$ = 9.2, $^4J_{HH}$=1.6, $C\underline{H}_{Ar(7)}$, 1H), 4.29 – 4.20 (m, $C\underline{H_2}OP$, 4H), 3.82 (ddd, $^3J_{FH}$ = 37.7, $^2J_{HH}$ = 16.1, $^3J_{PH}$ = 5.8, $C\underline{H_2}C(F)P$, 1H), 3.74 (t, $^3J_{HH}$ = 6.9, $C\underline{H_2}NPht$, 2H), 3.66 (ddd, $^2J_{HH}$ = 16.1, $^3J_{FH}$ = 12.3, $^3J_{PH}$ = 6.5, $C\underline{H_2}C(F)P$, 1H), 2.70 – 2.62 (m, $C\underline{H_2}CH_2CH_2NPht$, 2H), 2.06 – 1.99 (m, $C\underline{H_2}CH_2NPht$, 2H), 1.36 – 1.32 (m, $C\underline{H_3}CH_2OP$, 6H), 1.35 (s, $C(C\underline{H_3})_3$, 9H). **^{13}C NMR** (176 MHz, $CDCl_3$) δ 168.4 (s, $\underline{C}ON$, 2C), 165.0 (dd, $^2J_{FC}$ = 22.1, $^2J_{PC}$ = 3.8, $\underline{C}O_2t$Bu, 1C), 144.9 (s, $\underline{C}_{Ar(9)}$, 1C), 134.0 (s, $\underline{C}H_{Pht}$, 2C), 133.6 (s, $\underline{C}H_{Ar(2)}$, 1C), 132.0 (s, $\underline{C}CH_{Pht}$, 2C), 126.2 (s, $\underline{CH}_{Ar(7)}$, 1C), 125.3 (s, $\underline{C}_{Ar(6)}$, 1C), 123.2 (s, $\underline{C}H_{Pht}$, 2C), 121.8 (d, J_{FC} = 4.7, $\underline{C}H_{Ar(5)}$, 1C), 117.3 (s, $\underline{C}H_{Ar(8)}$, 1C), 116.8 (d, $^3J_{PC}$ = 15.0, $\underline{C}_{Ar(3)}$, 1C), 95.7 (dd, $^1J_{FC}$ = 199.6, $^1J_{PC}$ = 159.6, $\underline{C}(F)P$, 1C), 84.5 (s, $CO_2\underline{C}Me_3$, 1C), 64.6 (d, $^2J_{PC}$ = 6.4, $\underline{C}H_2OP$, 1C), 64.3 (d, $^2J_{PC}$ = 7.0, $\underline{C}H_2OP$, 1C), 37.4 (s, $\underline{C}H_2NPht$, 1C), 30.2 (s, $\underline{C}_{Ar(6)}\underline{C}H_2$, 1C), 29.3 (s, $\underline{C}H_2CH_2NPht$, 1C), 28.3 (d, $^2J_{FC}$ = 21.1, $\underline{C}H_2C(F)P$, 1C), 27.8 (s, $C(\underline{C}H_3)_3$, 3C), 16.4 (d, $^3J_{PC}$ = 5.6, $\underline{C}H_3CH_2OP$, 1C). **^{31}P NMR** (101 MHz, $CDCl_3$) δ 12.58 (d, $^2J_{PF}$ = 83.1).

3.6. Transformation of the nitrile group into amide – synthesis of compounds 7e and 7j':

Compound **7c** (50 mg) was dissolved in the mixture of EtOH:DMSO (*v:v* 4:1, 2 mL). Then, the reaction mixture was cooled to 0 °C and K_2CO_3 (0.15 equiv, 2.4 mg) was added followed by H_2O_2 (30% in water, 5 equiv, 60 μL). The mixture was stirred for 24h at room temperature. Product **7e** was extracted using ethyl acetate (3x 5 mL). Organic phase was dried over $MgSO_4$. After evaporation of solvents, product was obtained as a yellow solid with purity >95%.

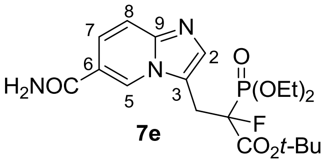

***Tert*-butyl 3-(6-carbamoylimidazo[1,2-*a*]pyridin-3-yl)-2-(diethoxyphosphoryl)-2-fluoropropanoate (7e):** Yield 72%. **^{1}H NMR** (700 MHz, $CDCl_3$) *δ* 1.27-1.38 (m, $(C\underline{H_3})_3C$, $C\underline{H_3}CH_2OP$, 15H), 3.74 (ddd, $^2J_{HH}$ = 16.2, $^3J_{FH}$ = 12.9, $^3J_{PH}$ = 6.9, $C\underline{H_2}CFP$, 1H), 3.89 (ddd, $^3J_{FH}$ = 36.0, $^2J_{HH}$ = 16.2, $^3J_{PH}$ = 6.6, $C\underline{H_2}CFP$, 1H), 4.15 - 4.28 (m, $C\underline{H_2}OP$, 4H), 7.73 (d, $^3J_{HH}$ = 9.4, $CH_{Ar(8)}$, 1H), 7.54 (s, $CH_{Ar(2)}$, 1H), 7.59 (d, $^3J_{HH}$ = 9.4, $^5J_{HH}$ = 1.4, $CH_{Ar(7)}$, 1H), 8.89 (bs, $CH_{Ar(5)}$, 1H), **^{31}P NMR** (283 MHz, $CDCl_3$) *δ* 12.05, (d, $^2J_{PF}$ = 83.4), **^{13}C NMR** (176 MHz, $CDCl_3$) *δ* 16.6 (d, $^3J_{PC}$ = 5.7, $\underline{C}H_3CH_2OP$, 1C), 16.65 (d, $^3J_{PC}$ = 5.7, $\underline{C}H_3CH_2OP$, 1C), 28.0 (s, $C(\underline{C}H_3)_3$, 3C), 28.4 (d, $^2J_{FC}$ = 21.0, $^2J_{PC}$ = 2.8, $\underline{C}H_2CFP$, 1C), 64.9 (d, $^2J_{PC}$ = 7.2, $\underline{C}H_2OP$, 1C), 65.1 (d, $^2J_{PC}$ = 6.7, $\underline{C}H_2OP$, 1C), 85.0 (s, $\underline{C}(CH_3)_3$, 1C), 95.5 (dd, $^1J_{FC}$ = 199.5, $^1J_{PC}$ = 160.2, $CH_2\underline{C}FP$, 1C), 117.26 (s, $CH_{Ar(8)}$, 1C), 119.65 (s, $C_{Ar(6)}$, 1C), 118.4 (d, $^3J_{PC}$ = 14.4, $C_{Ar(3)}$, 1C), 123.15 (s, $CH_{Ar(7)}$, 1C), 126.5 (d, $^5J_{FC}$ = 4.0, $CH_{Ar(5)}$, 1C), 135.6 (s, $CH_{Ar(2)}$, 1C), 146.4 (s, $C_{Ar(9)}$, 1C), 165.2 (dd, $^2J_{FC}$ = 21.9, $^2J_{PC}$ = 3.6, $\underline{C}O_2$, 1C), 167.5 (s, $\underline{C}ONH_2$, 1C).

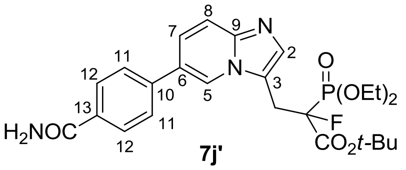

***Tert*-butyl 3-(6-(4-carbamoylphenyl)imidazo[1,2-*a*]pyridin-3-yl)-2-(diethoxyphosphoryl)-2-fluoropropanoate (7j'):** Scale of reaction: compound **7j** 68 mg. Yield 57%. **^{1}H NMR** (700 MHz, $CDCl_3$) δ 1.32 (t, $^{3}J_{HH}$ = 7.1, POCH$_2$C$\underline{H}_3$, 3H), 1.34 (t, $^{3}J_{HH}$ = 7.1, POCH$_2$C$\underline{H}_3$, 3H), 1.34 (s, C(C$\underline{H}_3$)$_3$, 9H), 3.74 (ddd, $^{2}J_{HH}$ = 16.1, $^{3}J_{FH}$ = 12.3, $^{3}J_{PH}$ = 6.6, C$\underline{H}_2$CFP, 1H), 3.91 (ddd, $^{3}J_{FH}$ = 37.3, $^{2}J_{HH}$ = 16.2, $^{3}J_{PH}$ = 5.9, C$\underline{H}_2$CFP, 1H), 4.16-4.29 (m, POC$\underline{H}_2$, 4H), 7.38 (dd, $^{3}J_{HH}$ = 9.3, $^{4}J_{HH}$ = 1.8, CH$_{Ar}$, 1H), 7.53 (s, CH$_{Ar(2)}$, 1H), 7.58 (d, $^{3}J_{HH}$ = 8.4, CH$_{Ar(11)}$, 2H), 7.60 (d, $^{3}J_{HH}$ = 9.6, CH$_{Ar}$, 1H), 7.88 (d, $^{3}J_{HH}$ = 8.4, CH$_{Ar(12)}$, 2H), 8.32 (bs, CH$_{Ar(5)}$, 1H). **^{31}P NMR** (284 MHz, $CDCl_3$) δ 12.02 (d, $^{2}J_{PF}$ = 83.3). **^{13}C NMR** (176 MHz, $CDCl_3$) δ 16.46 (d, $^{3}J_{PC}$ = 5.7, POCH$_2$$\underline{C}H_3$, 1C), 16.49 (d, $^{3}J_{PC}$ = 5.8, POCH$_2$$\underline{C}H_3$, 1C), 27.8 (s, ($\underline{C}H_3$)$_3$C, 3C), 28.4 (d, $^{2}J_{FC}$ = 20.6, $\underline{C}$H$_2$CPF, 1C), 64.5 (d, $^{2}J_{PC}$ = 7.1, PO$\underline{C}$H$_2$, 1C), 64.7 (d, $^{2}J_{PC}$ = 7.1, PO$\underline{C}$H$_2$, 1C), 84.7 (s, (CH$_3$)$_3$$\underline{C}$, 1C), 95.8 (dd, $^{1}J_{FC}$ = 199.6, $^{1}J_{PC}$ = 160.1, $\underline{C}$PF, 1C), 117.6 (d, $^{3}J_{PC}$ = 14.5, C$_{Ar(3)}$, 1C), 117.8 (s, CH$_{Ar}$, 1C), 122.0 (d, $^{5}J_{FC}$ = 5.3, CH$_{Ar(5)}$, 1C), 124.5 (s, CH$_{Ar}$, 1C), 125.6(s, C$_{Ar(6)}$, 1C), 127.1 (s, CH$_{Ar(11)}$, 2C), 128.4 (s, CH$_{Ar(12)}$, 2C), 132.8 (s, C$_{Ar(13)}$, 1C), 134.9 (s, CH$_{Ar(2)}$, 1C), 140.9 (s, C$_{Ar(10)}$, 1C), 145.4 (s, C$_{Ar(9)}$, 1C), 165.1 (dd, $^{2}J_{FC}$ = 21.9, $^{2}J_{PC}$ = 3.8, $\underline{C}$O$_2$, 1C), 169.1 (s, $\underline{C}$ONH$_2$, 1C).

3. Copies of ^{1}H NMR, ^{13}C NMR and ^{31}P NMR spectra

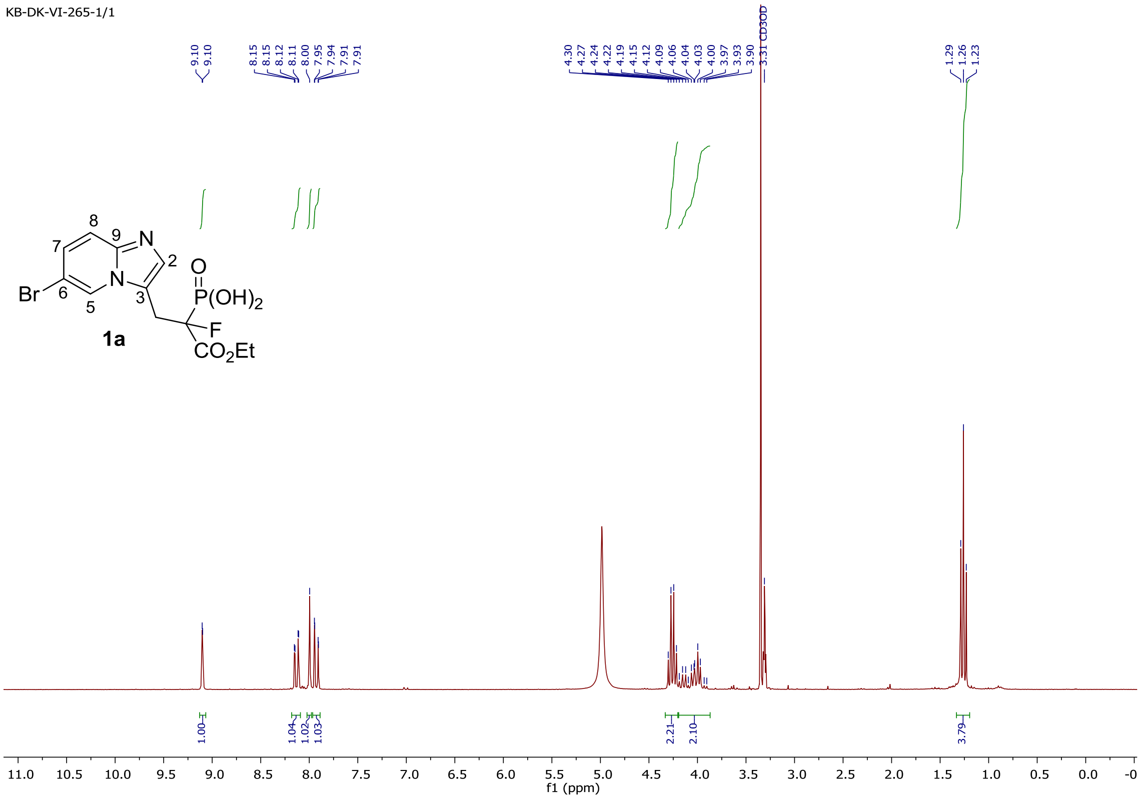

Figure S2. ^{1}H NMR of compound **1a** (700 MHz, CD_3OD).

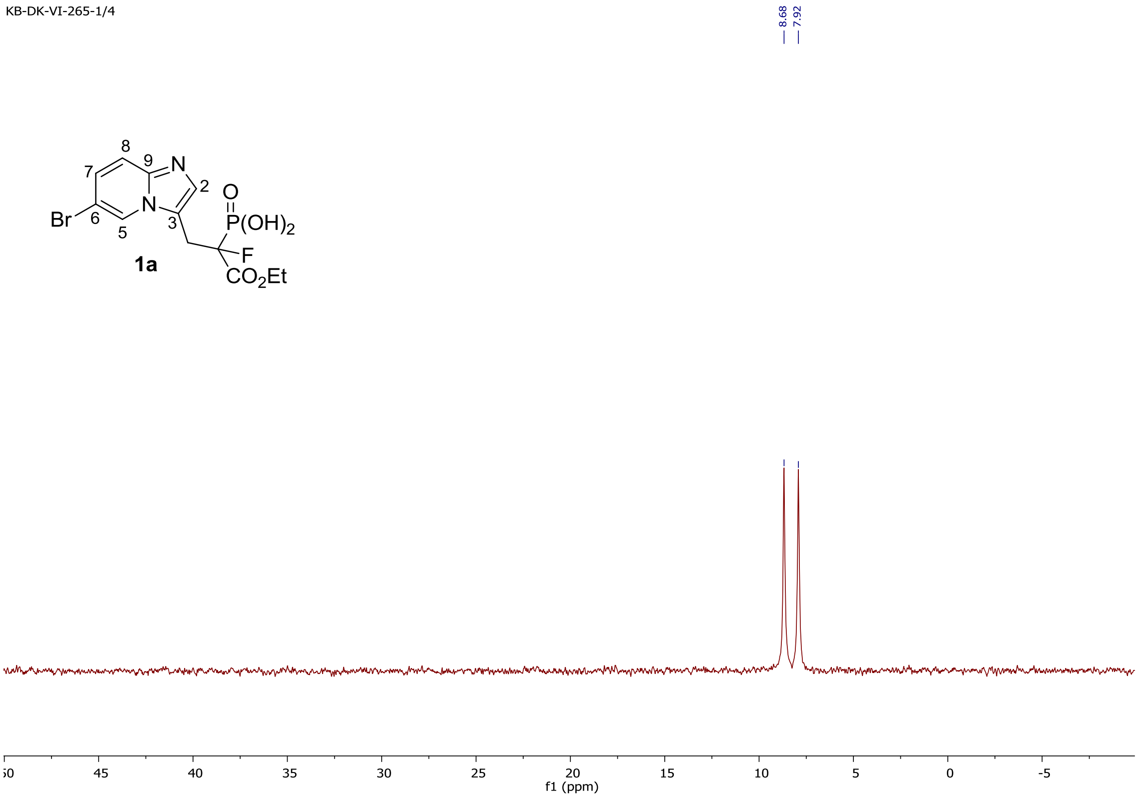

Figure S3. ^{31}P NMR of compound **1a** (284 MHz, CD_3OD).

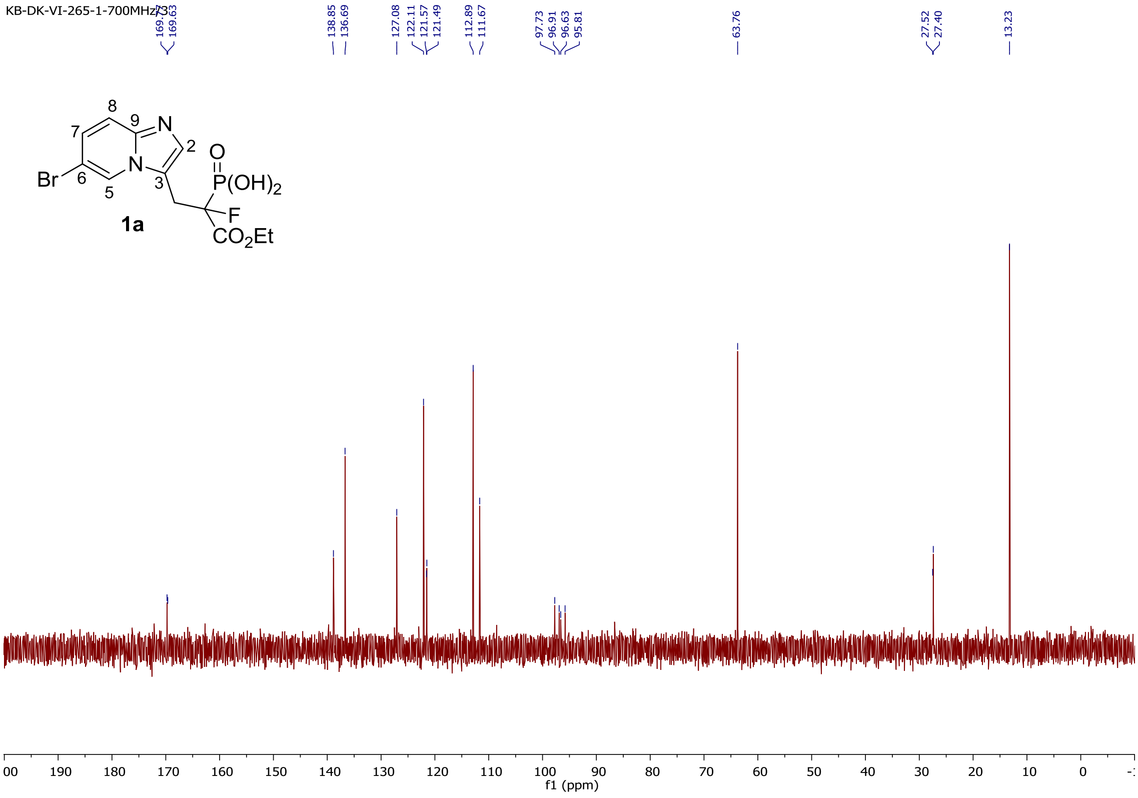

Figure S4. ^{13}C NMR of compound **1a** (176 MHz, CD_3OD).

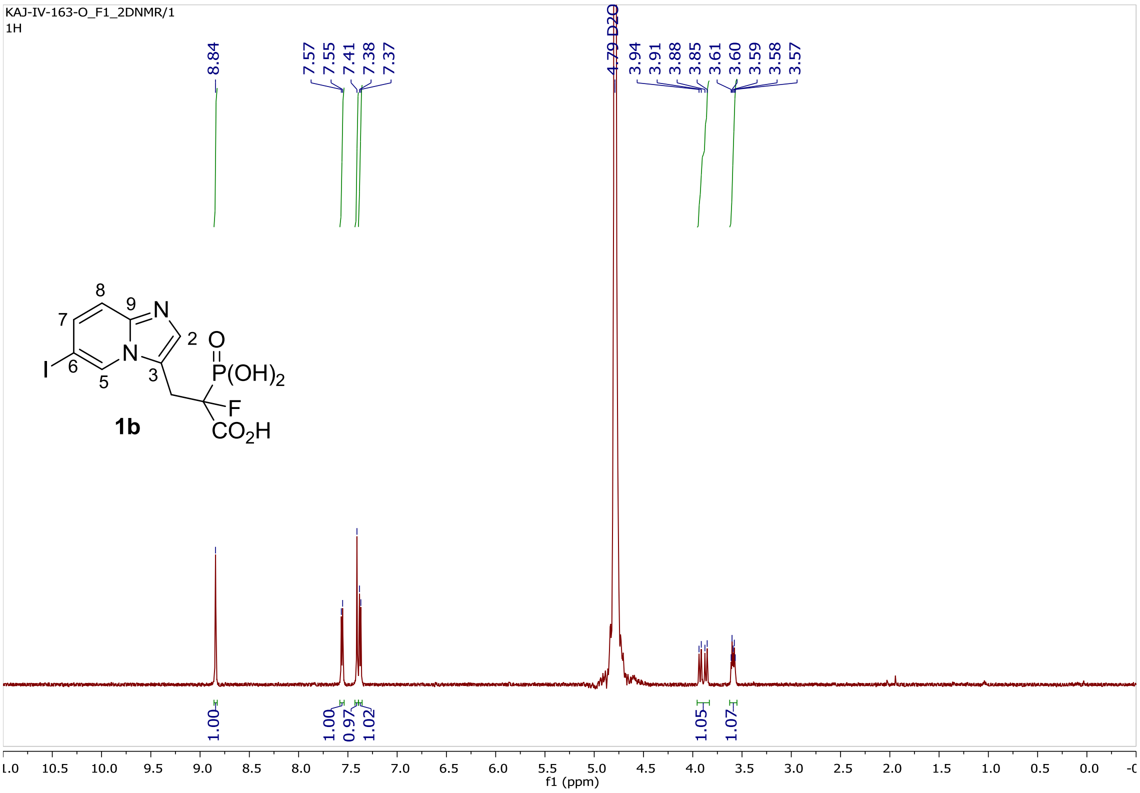

Figure S5. ^{1}H NMR of compound **1b** (700 MHz, D_2O pH 7).

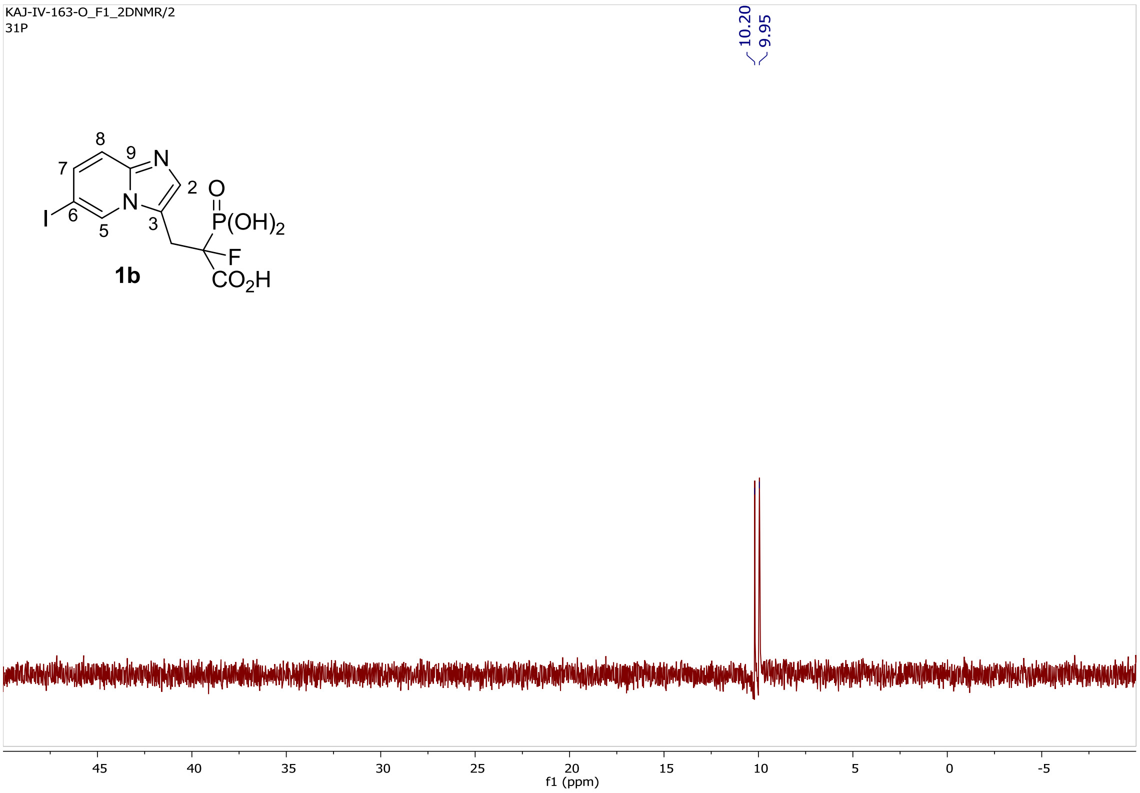

Figure S6. ^{31}P NMR of compound **1b** (286 MHz, D_2O pH 7).

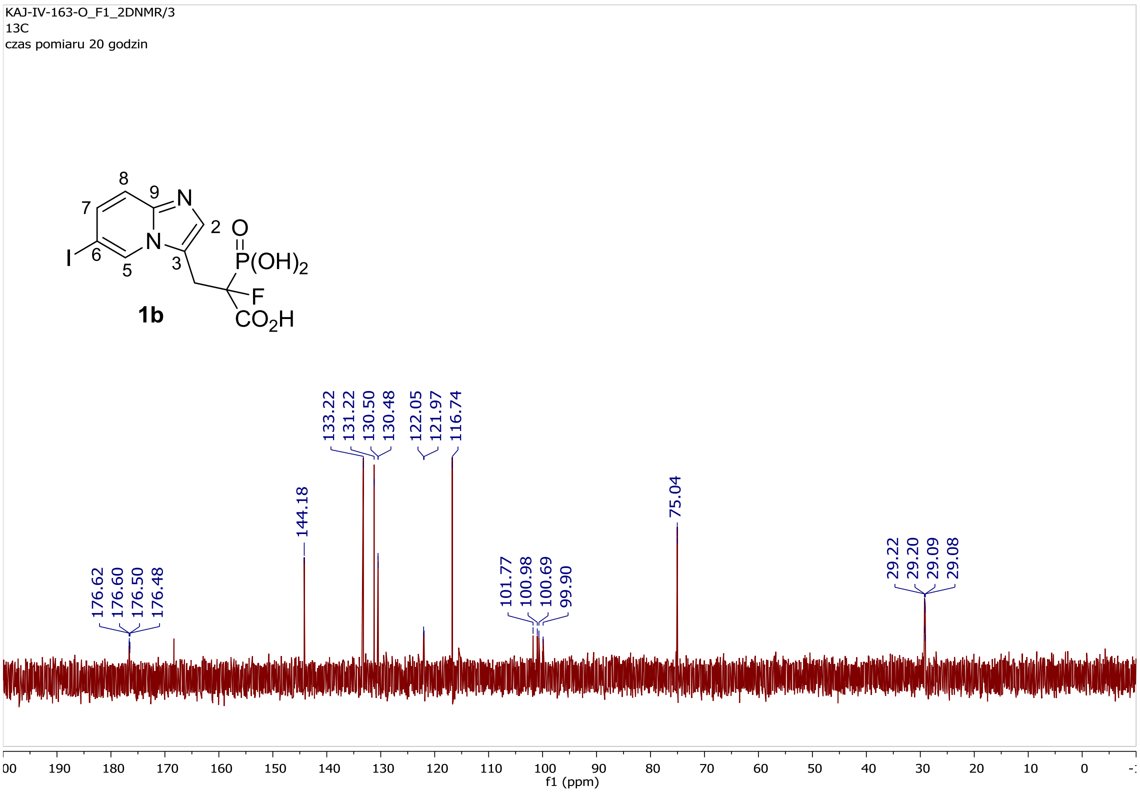

Figure S7. ^{13}C NMR of compound **1b** (176 MHz, D_2O pH 7).

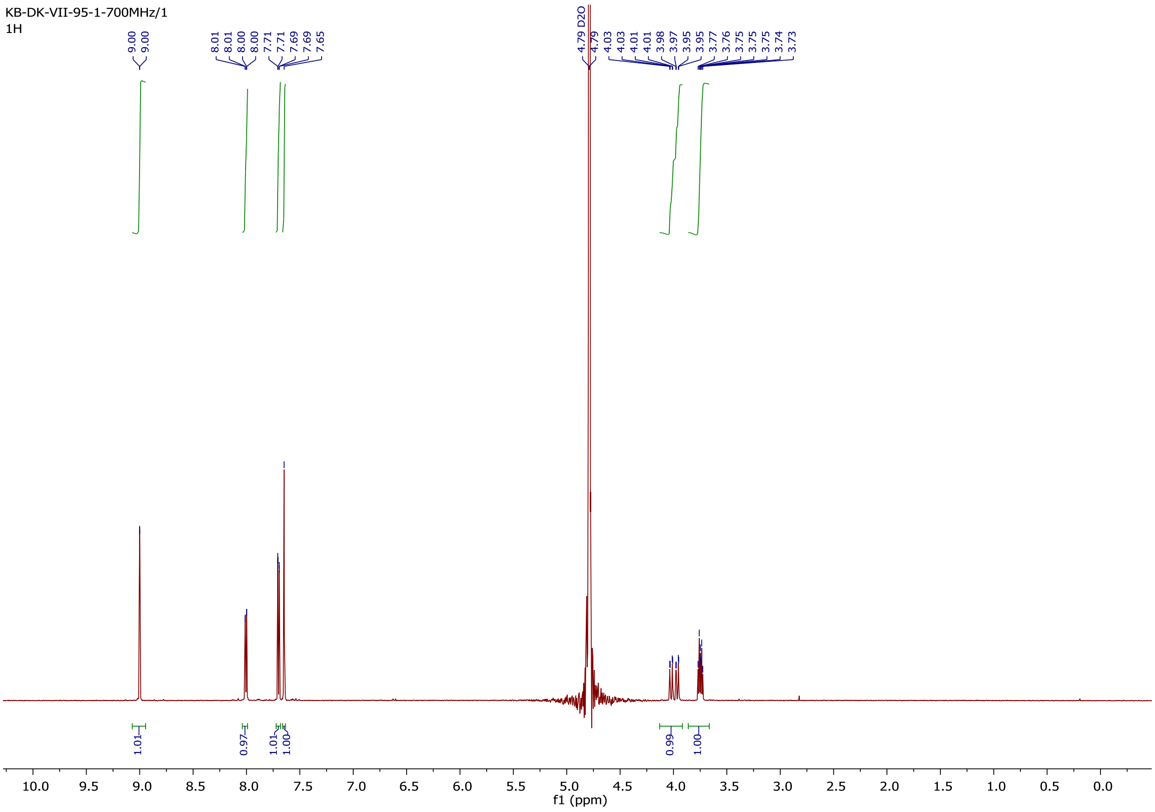

Figure S8. ^{1}H NMR of compound **1c** (700 MHz, D_2O pH 8).

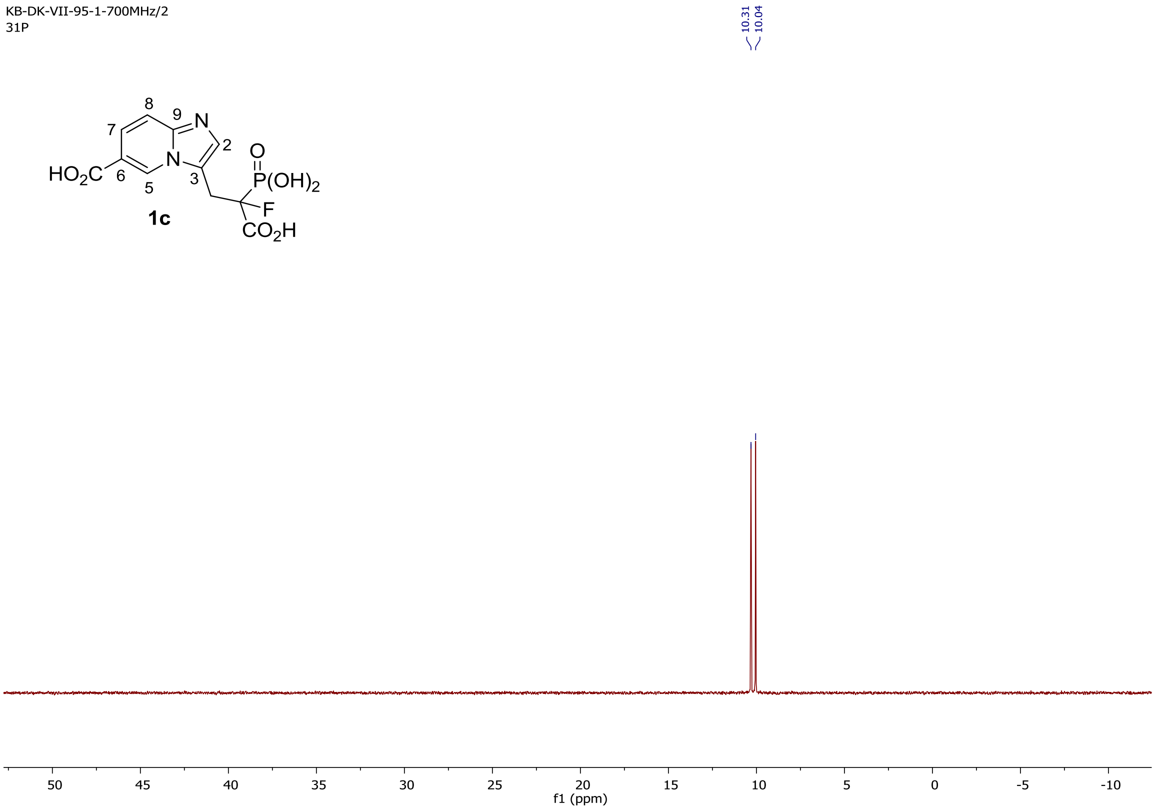

Figure S9. ^{31}P NMR of compound **1c** (284 MHz, D_2O pH 8).

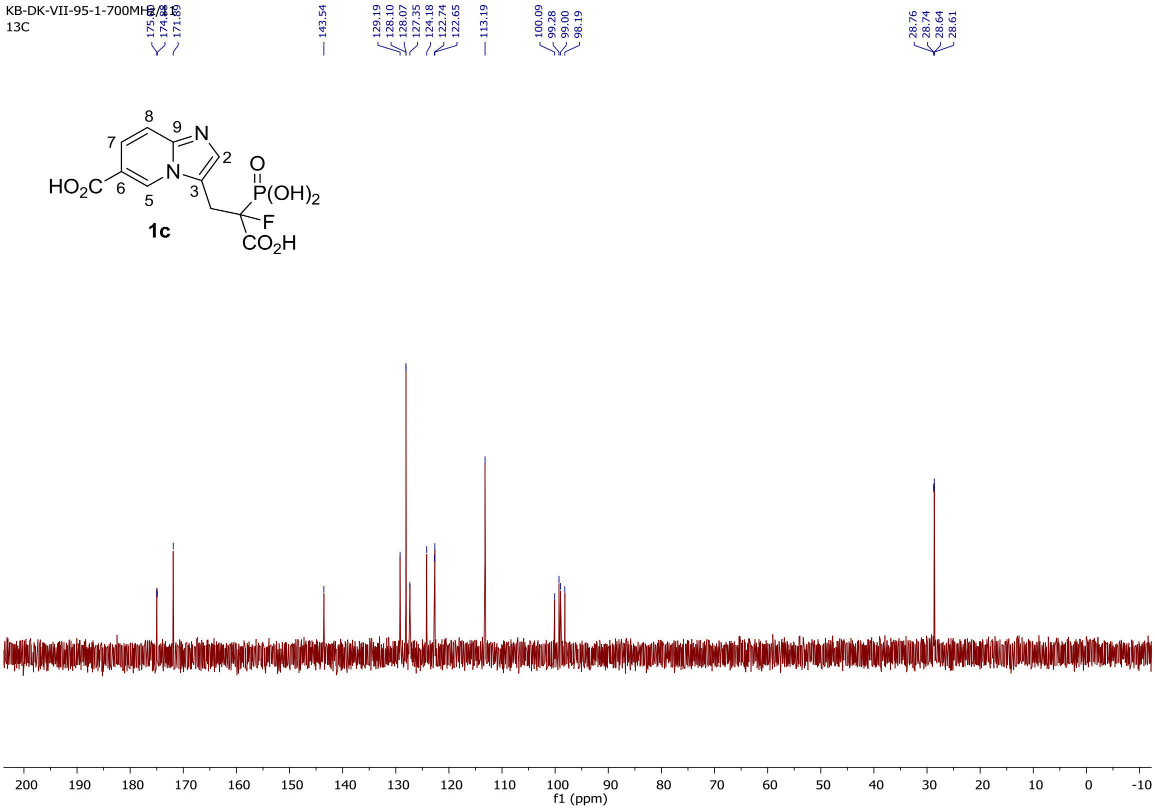

Figure S10. ^{13}C NMR of compound **1c** (176 MHz, D_2O pH 8).

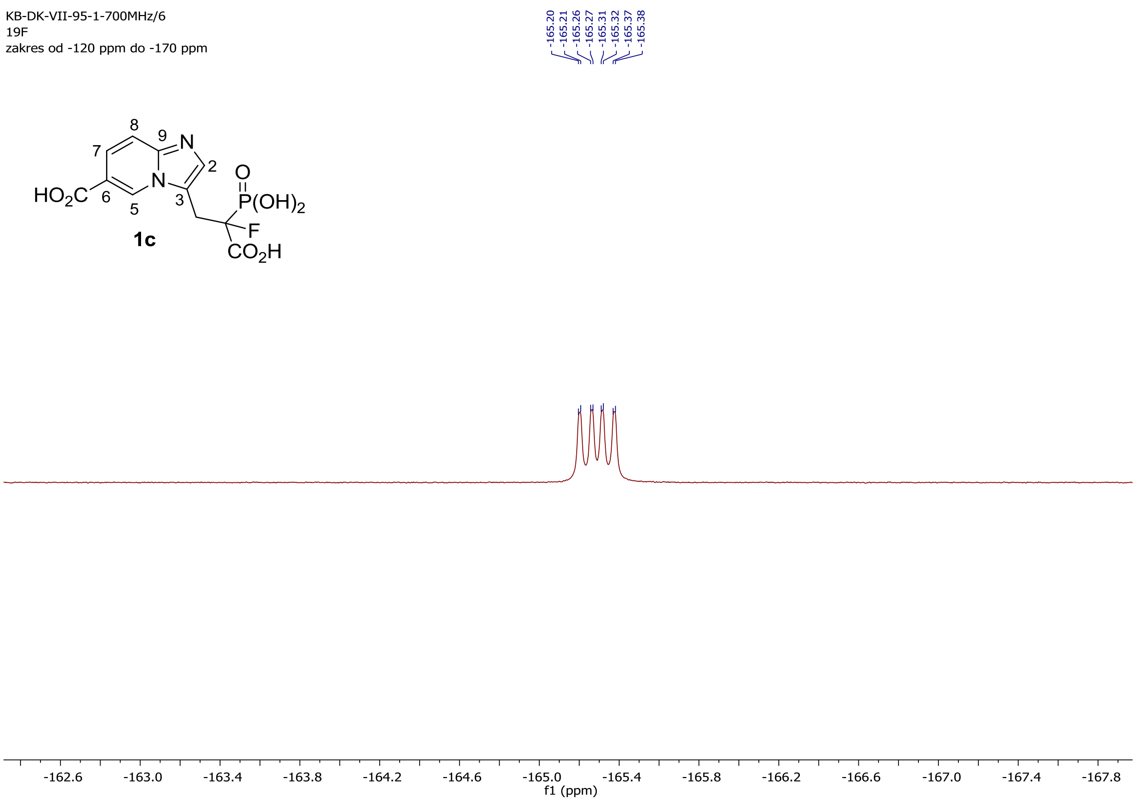

Figure S11. ^{19}F NMR of compound **1c** (659 MHz, D_2O pH 8).

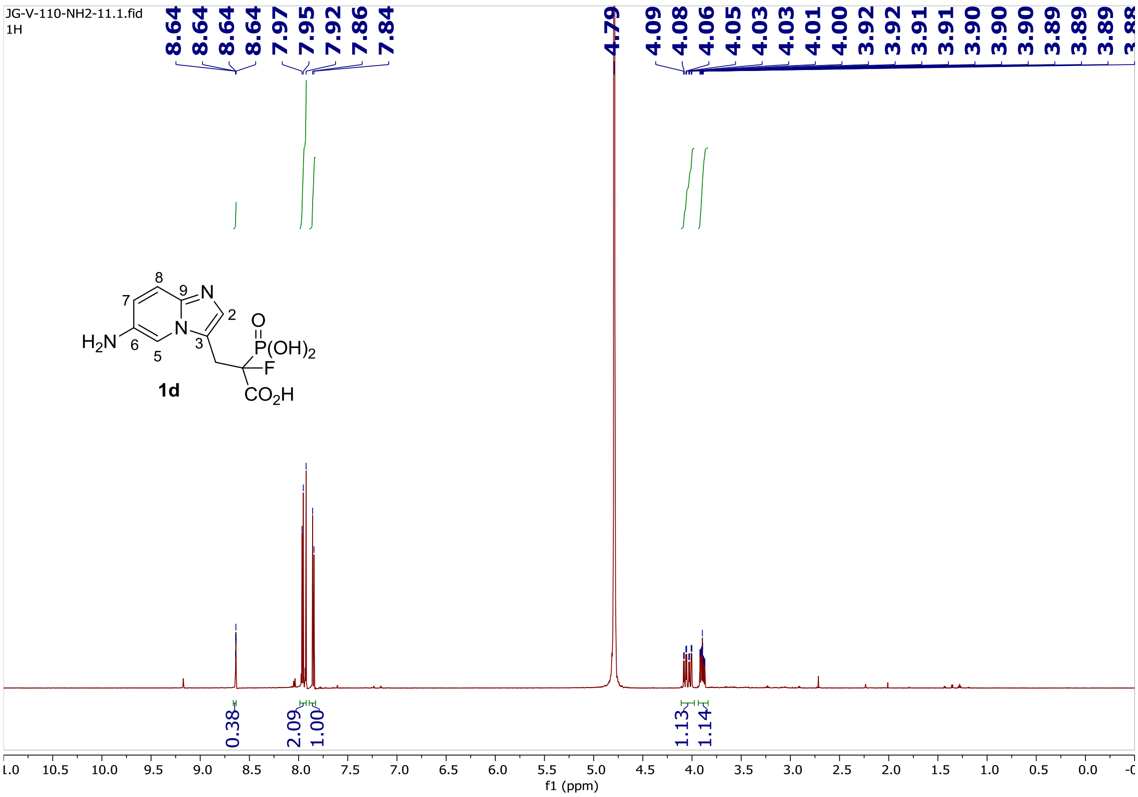

Figure S12.^{1}H NMR of compound **1d** (700 MHz, D_2O pH 2).

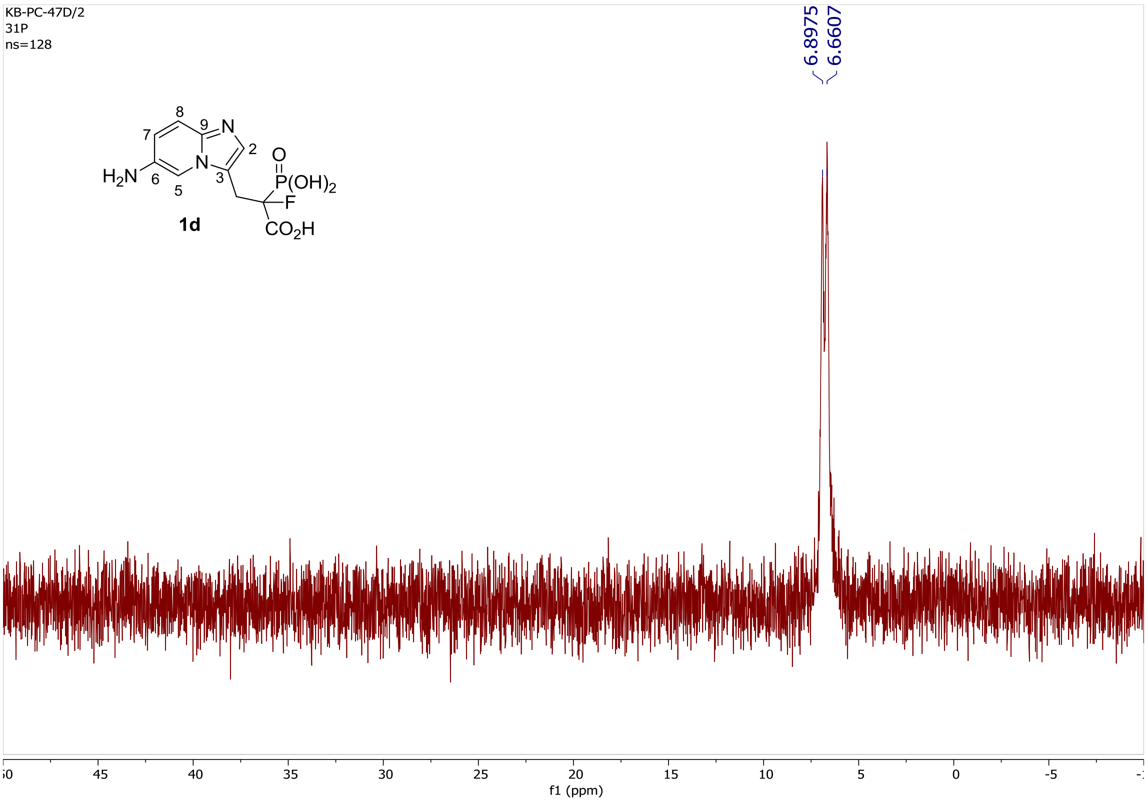

Figure S13.^{31}P NMR of compound **1d** (283 MHz, D_2O pH 2).

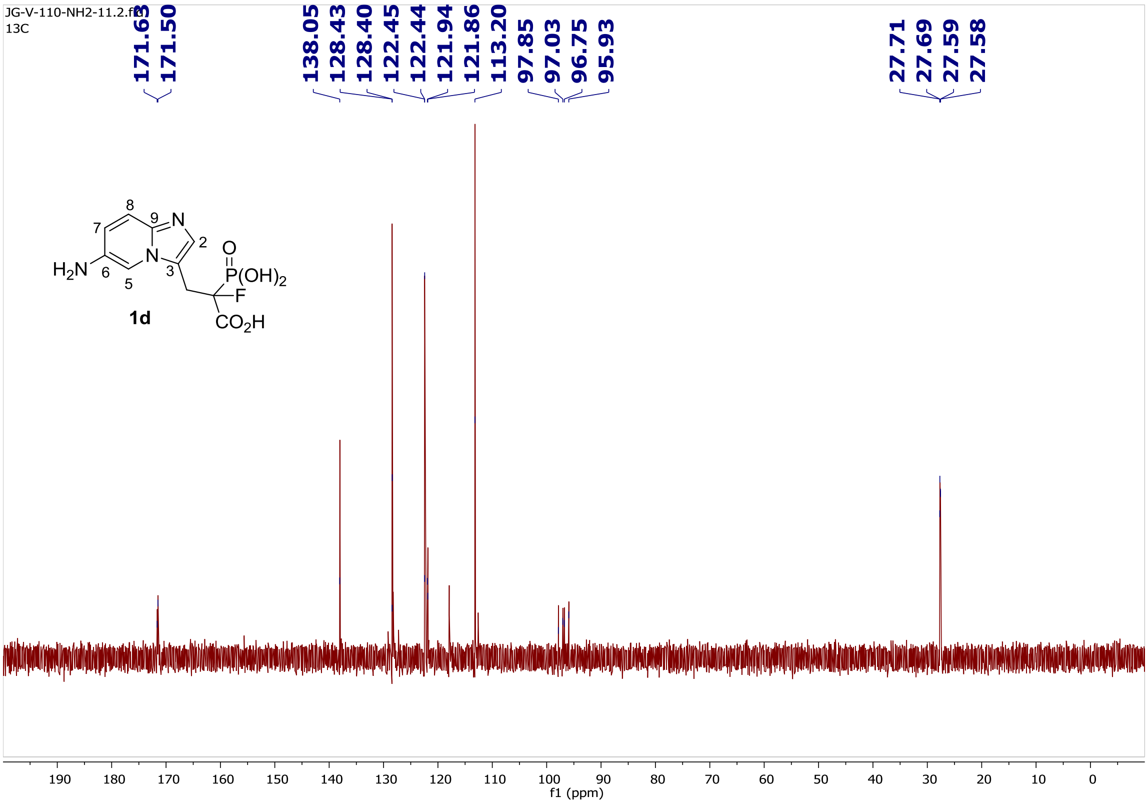

Figure S14.^{13}C NMR of compound **1d** (176 MHz, D_2O pH 2).

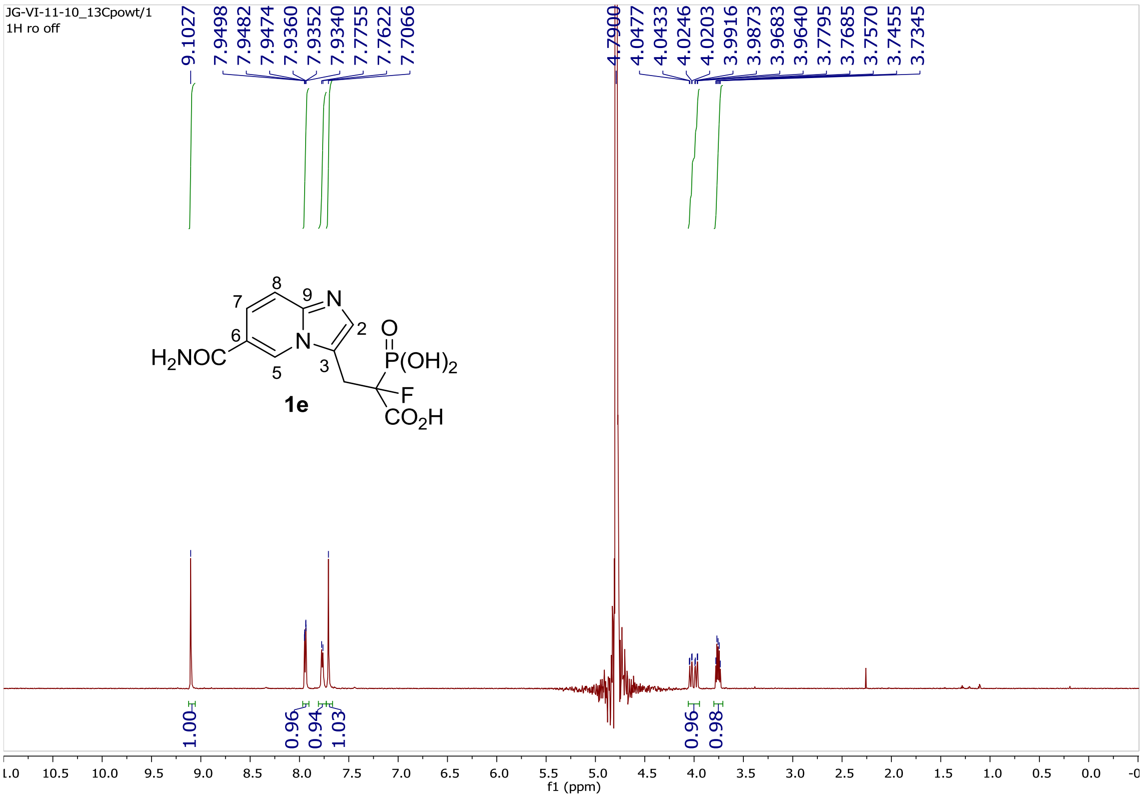

Figure S15.^{1}H NMR of compound **1e** (700 MHz, D_2O pH 7).

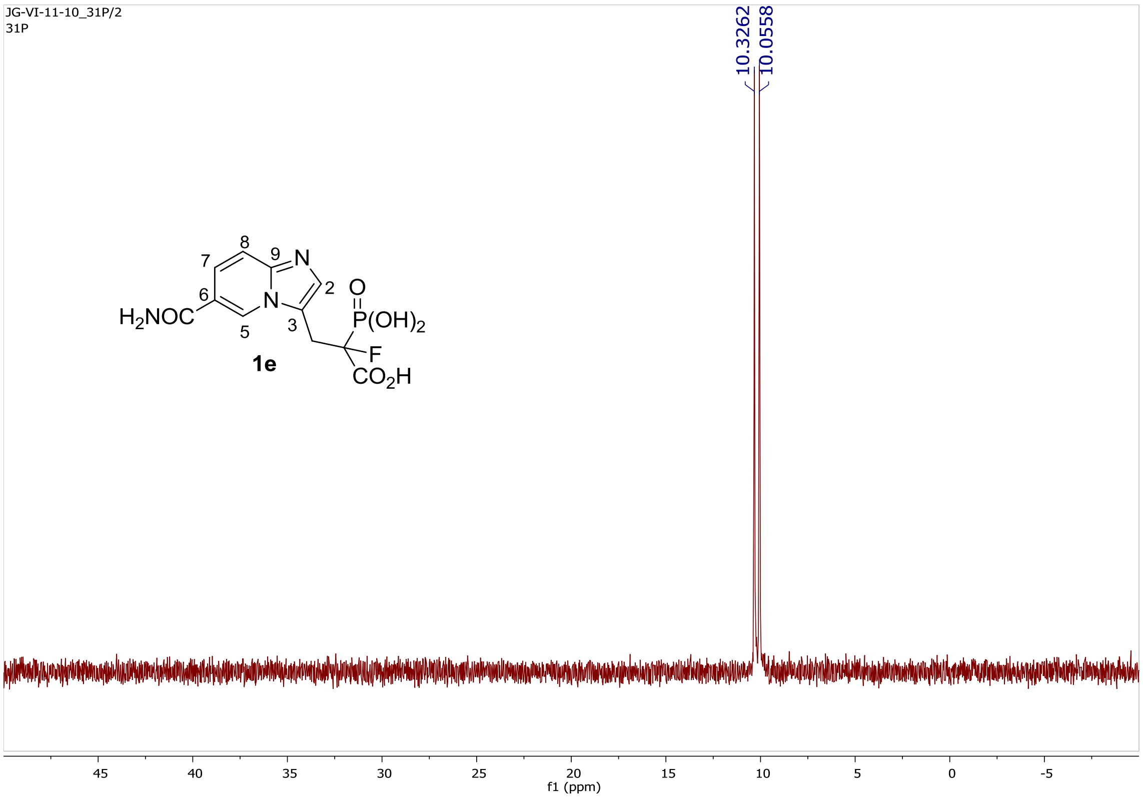

Figure S16.^{31}P NMR of compound **1e** (283 MHz, D_2O pH 7).

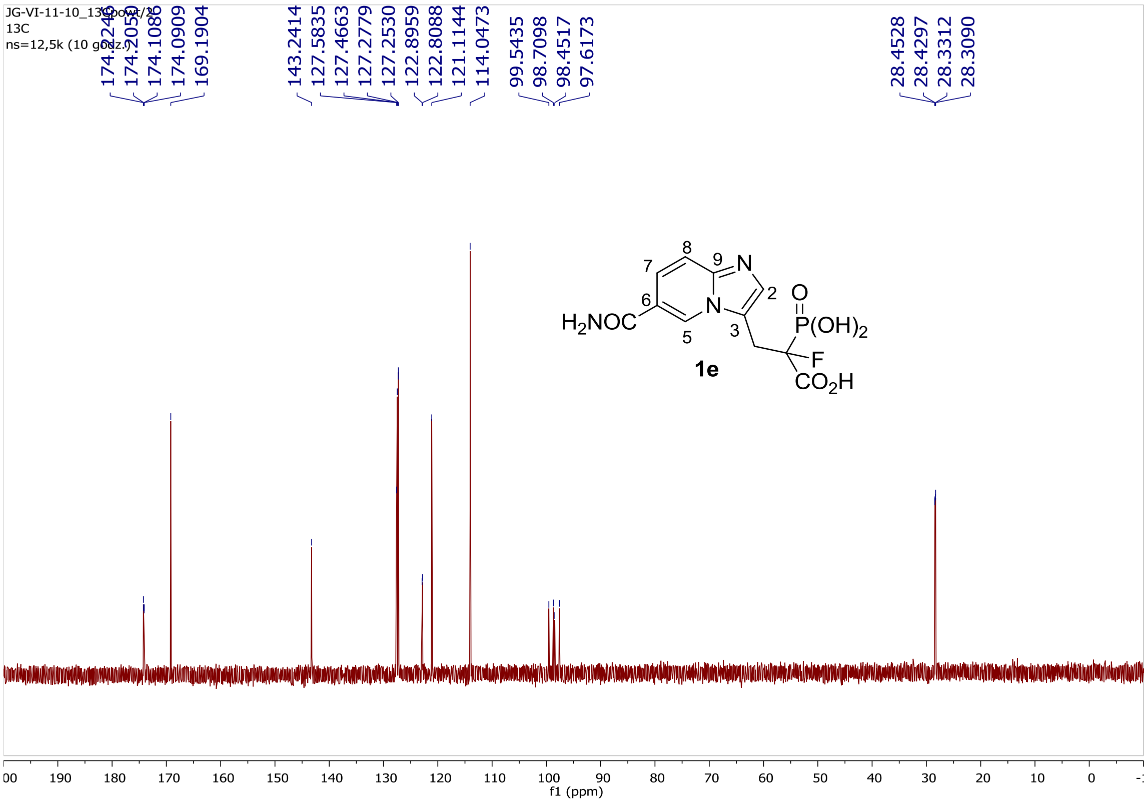

Figure S17. ^{13}C NMR of compound **1e** (176 MHz, D_2O pH 8).

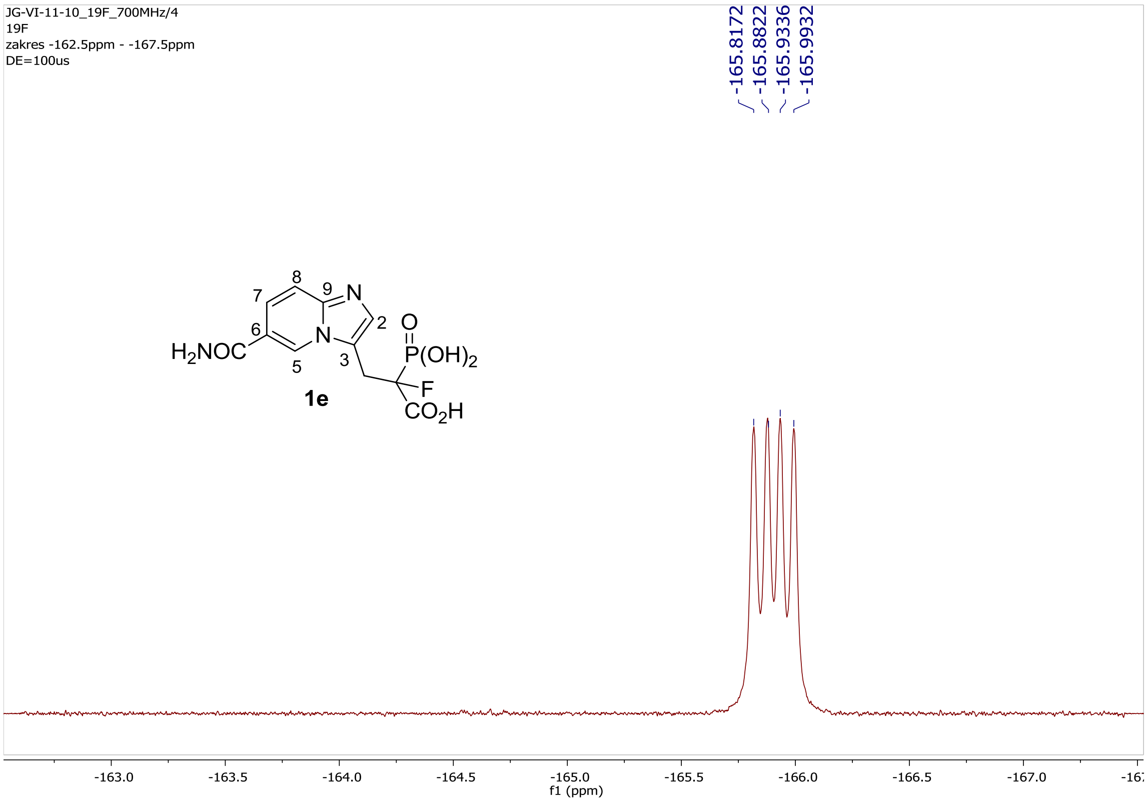

Figure S18. ^{19}F NMR of compound **1e** (659 MHz, D_2O pH 8).

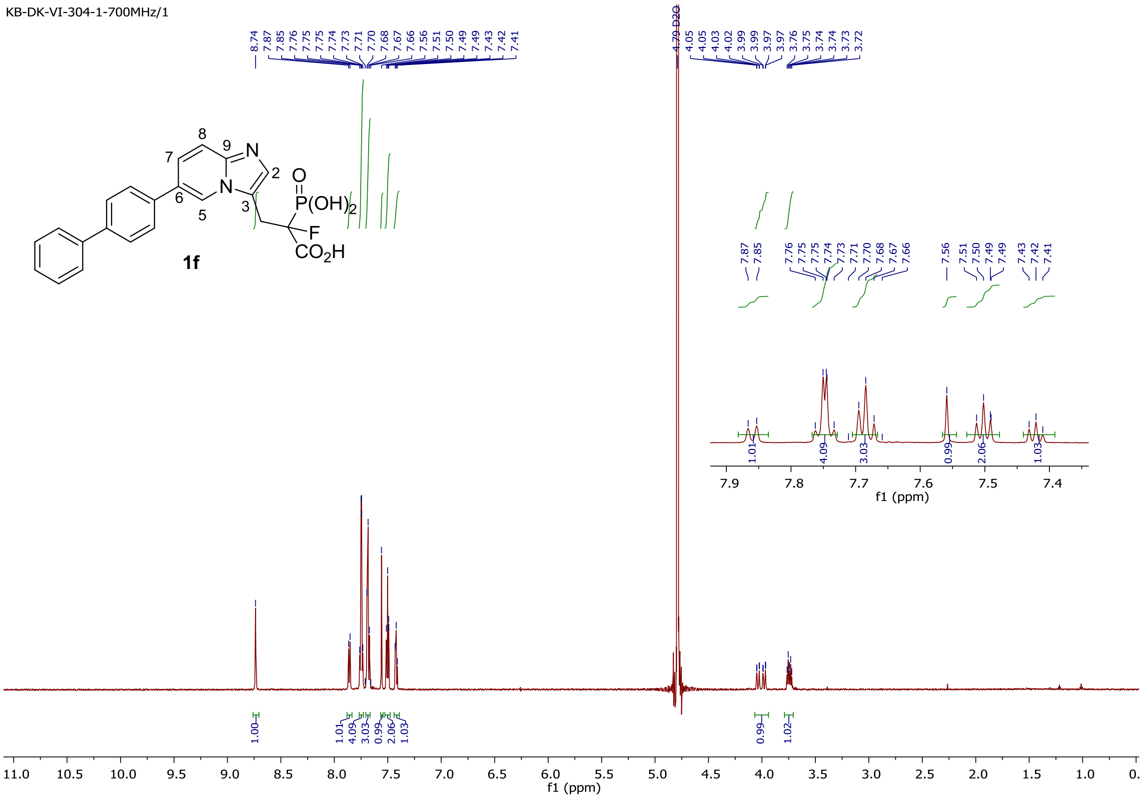

Figure S19. ^{1}H NMR of compound **1f** (700 MHz, D_2O pH 8).

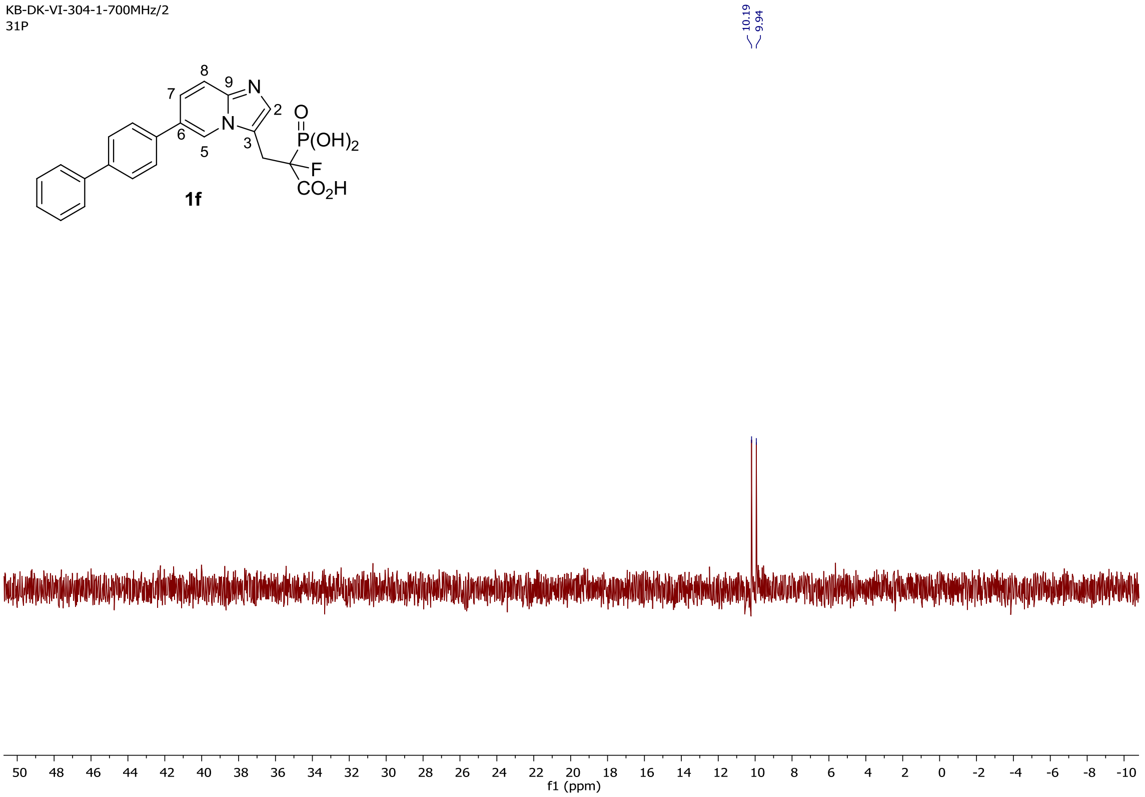

Figure S20. ^{31}P NMR of compound **1f** (284 MHz, D_2O pH 8).

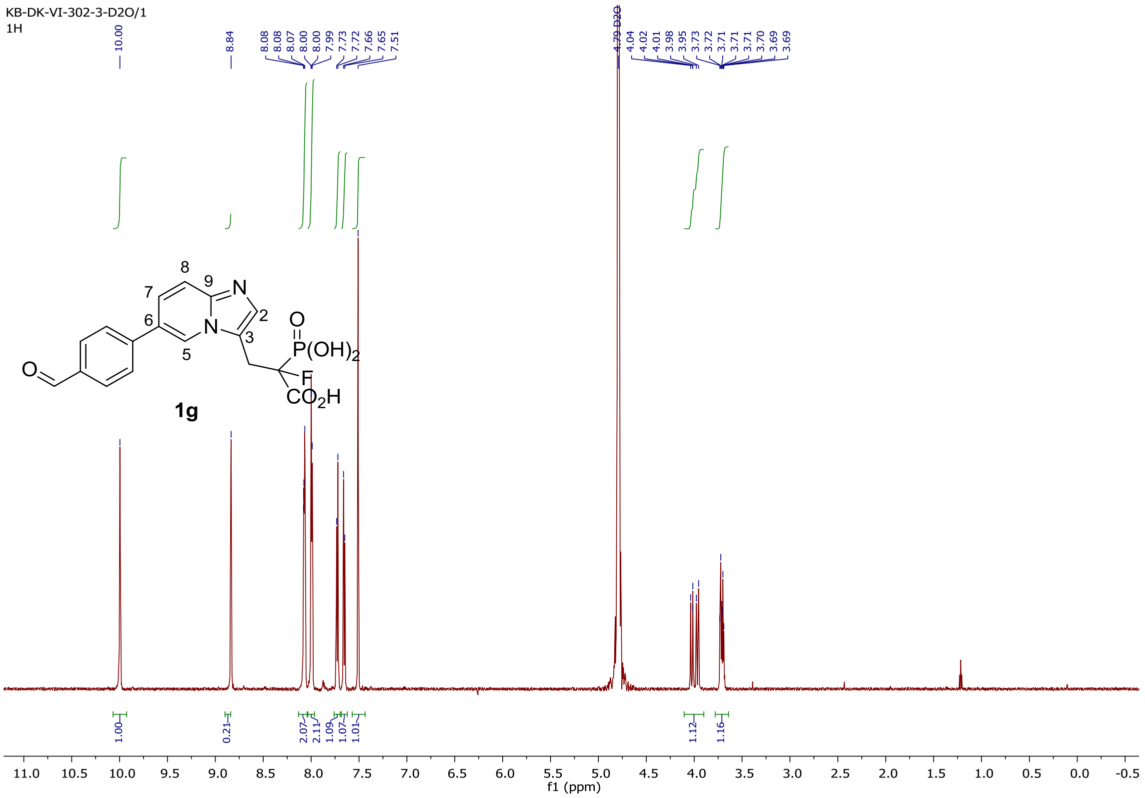

Figure S21. ^{1}H NMR of compound **1g** (700 MHz, D_2O pH 8).

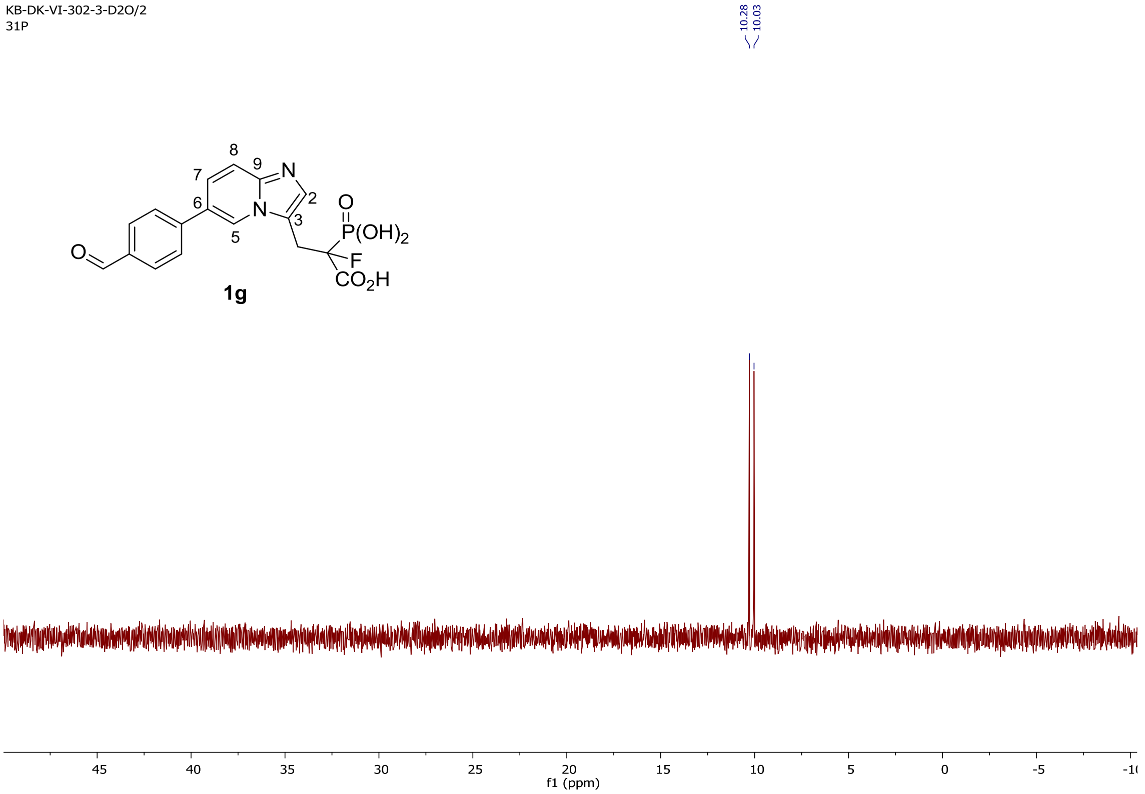

Figure S22. ^{31}P NMR of compound **1g** (284 MHz, D_2O pH 8).

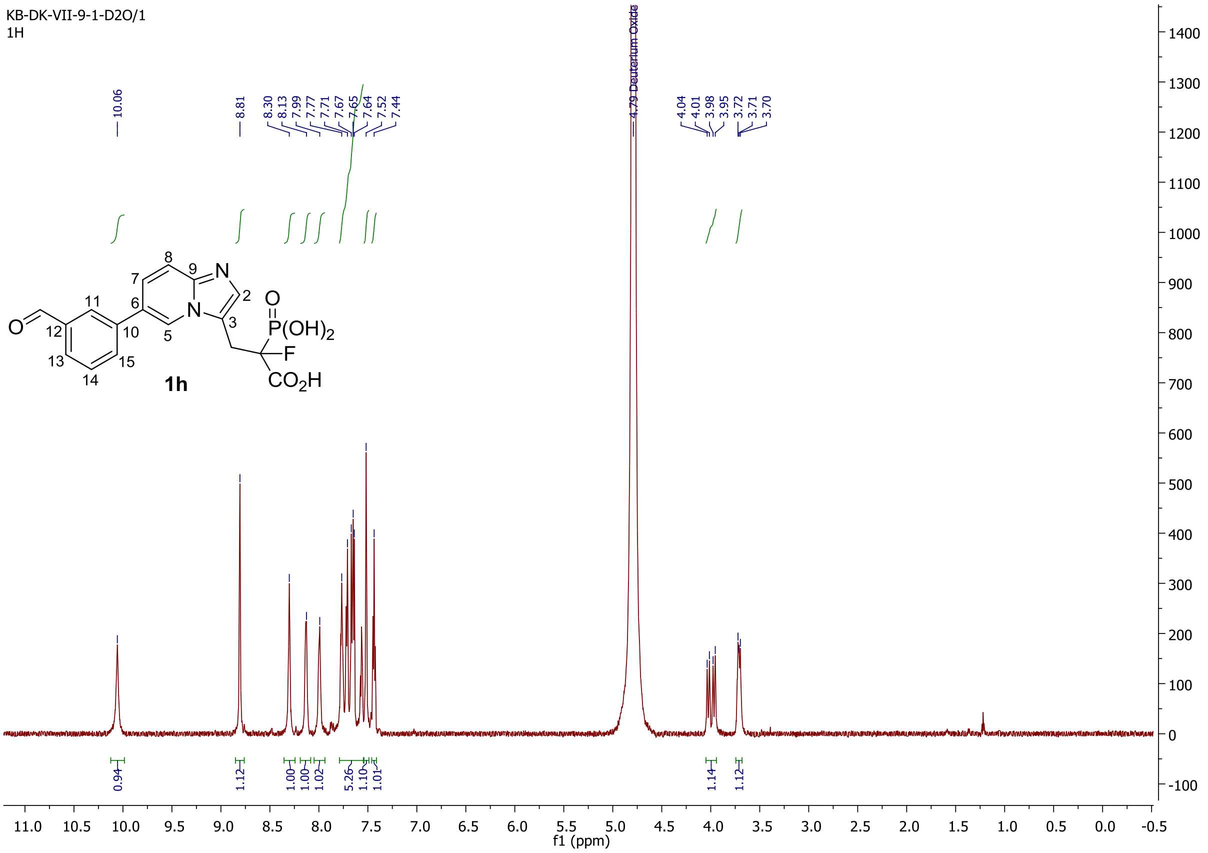

Figure S23. ^{1}H NMR of compound **1h** (700 MHz, D_2O pH 8).

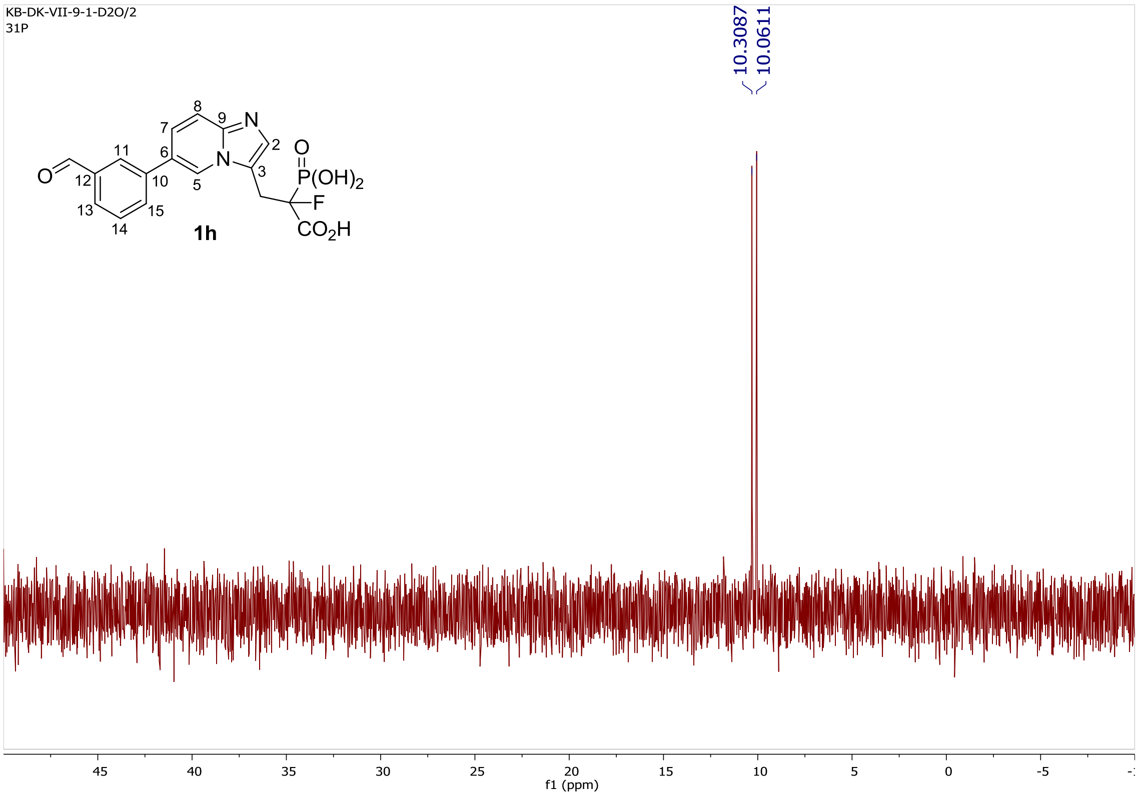

Figure S24. ^{31}P NMR of compound **1h** (283 MHz, D_2O pH 8).

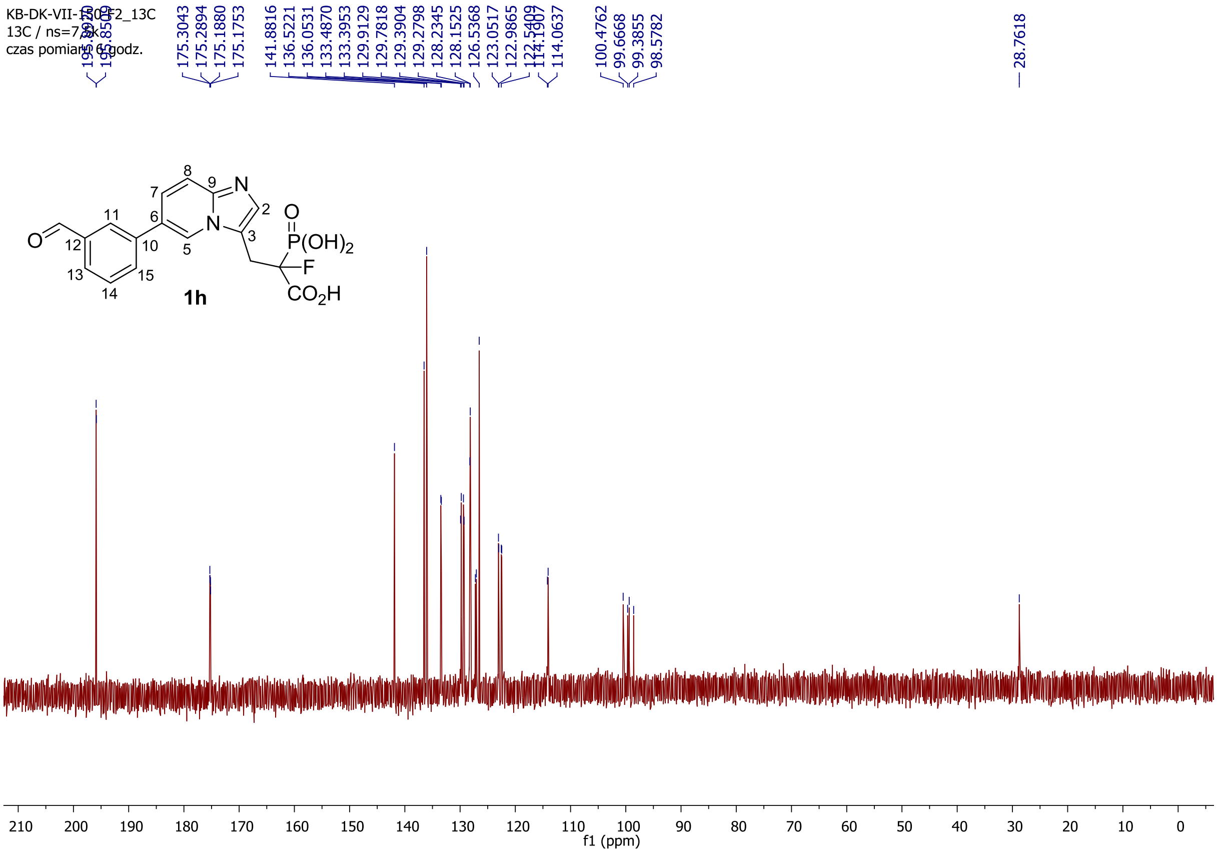

Figure S25. ^{13}C NMR of compound **1h** (176 MHz, D_2O pH 8).

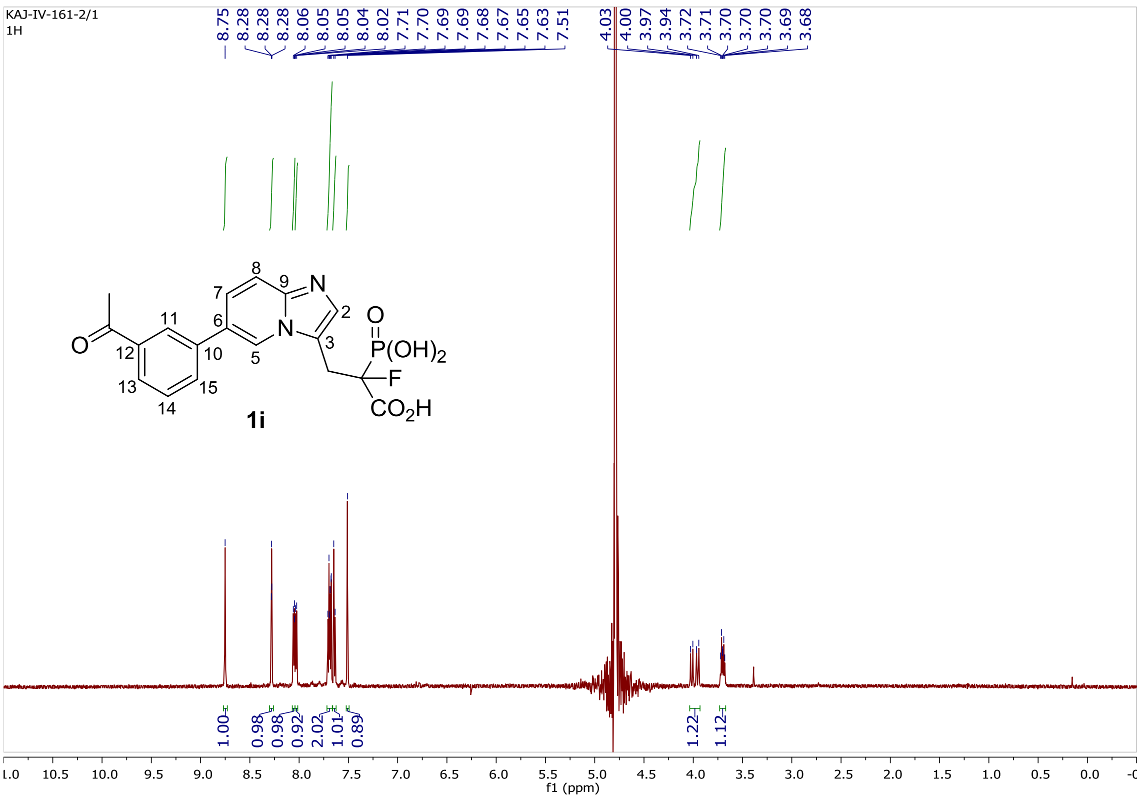

Figure S26. ^{1}H NMR of compound **1i** (700 MHz, D_2O pH 8).

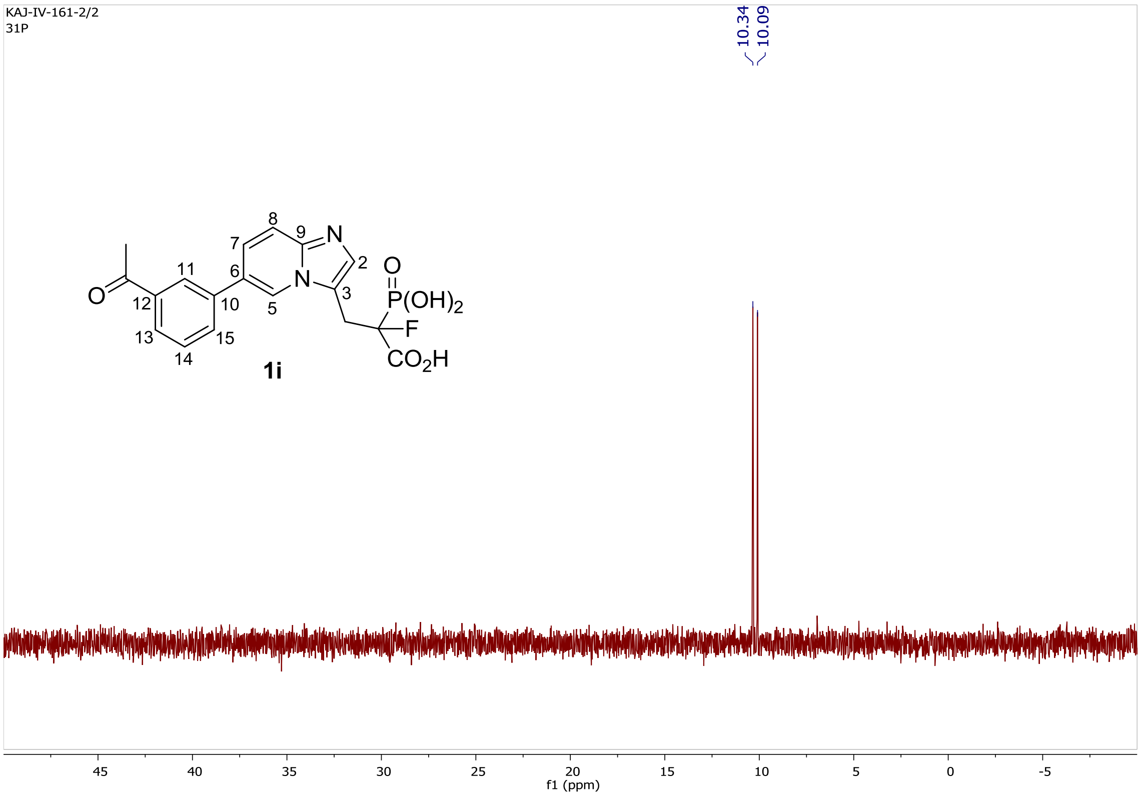

Figure S27. ^{31}P NMR of compound **1i** (284 MHz, D_2O pH 8).

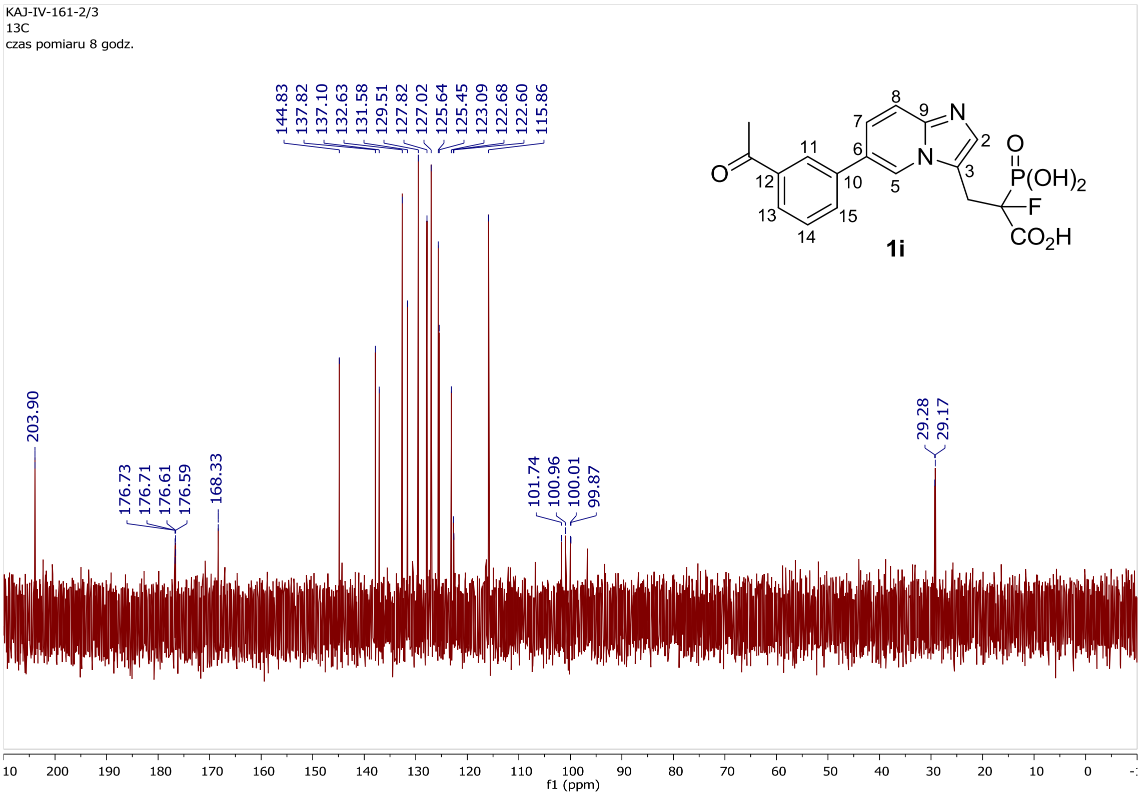

Figure S28. ^{13}C NMR of compound **1i** (176 MHz, D_2O pH 8).

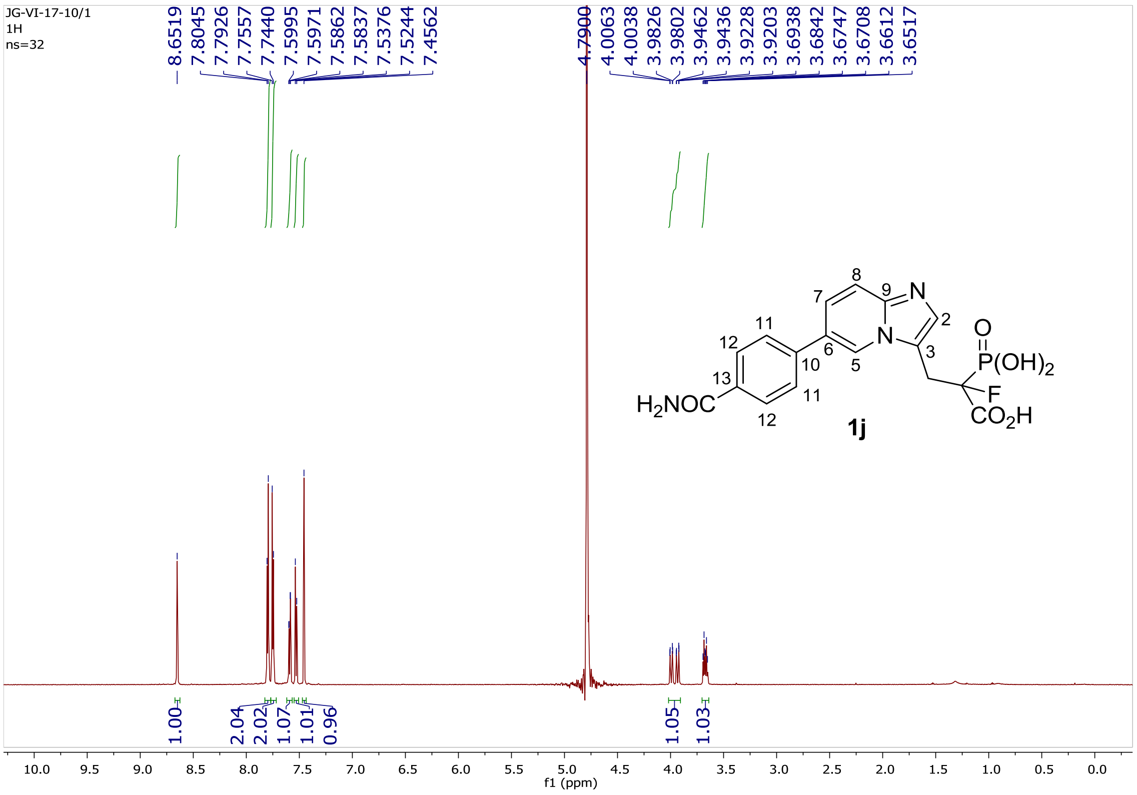

Figure S29. ^{1}H NMR of compound **1j** (700 MHz, D_2O pH 7).

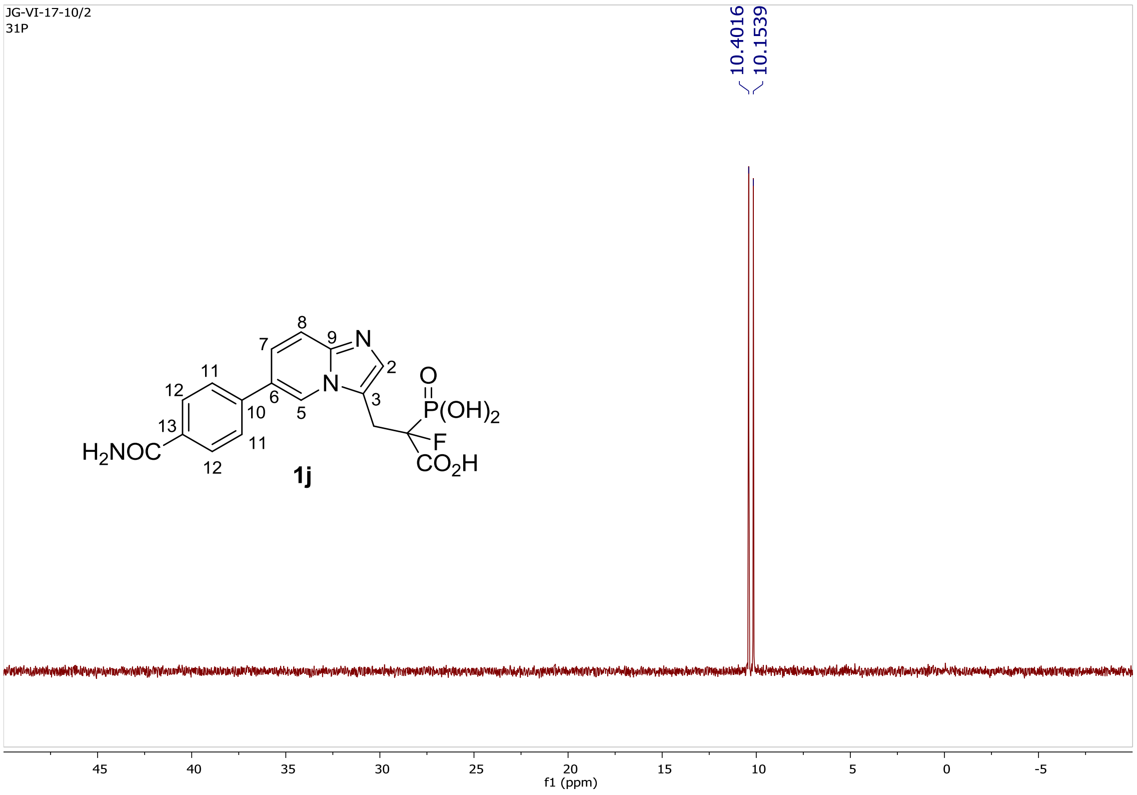

Figure S30. ^{31}P NMR of compound **1j** (284 MHz, D_2O pH 7).

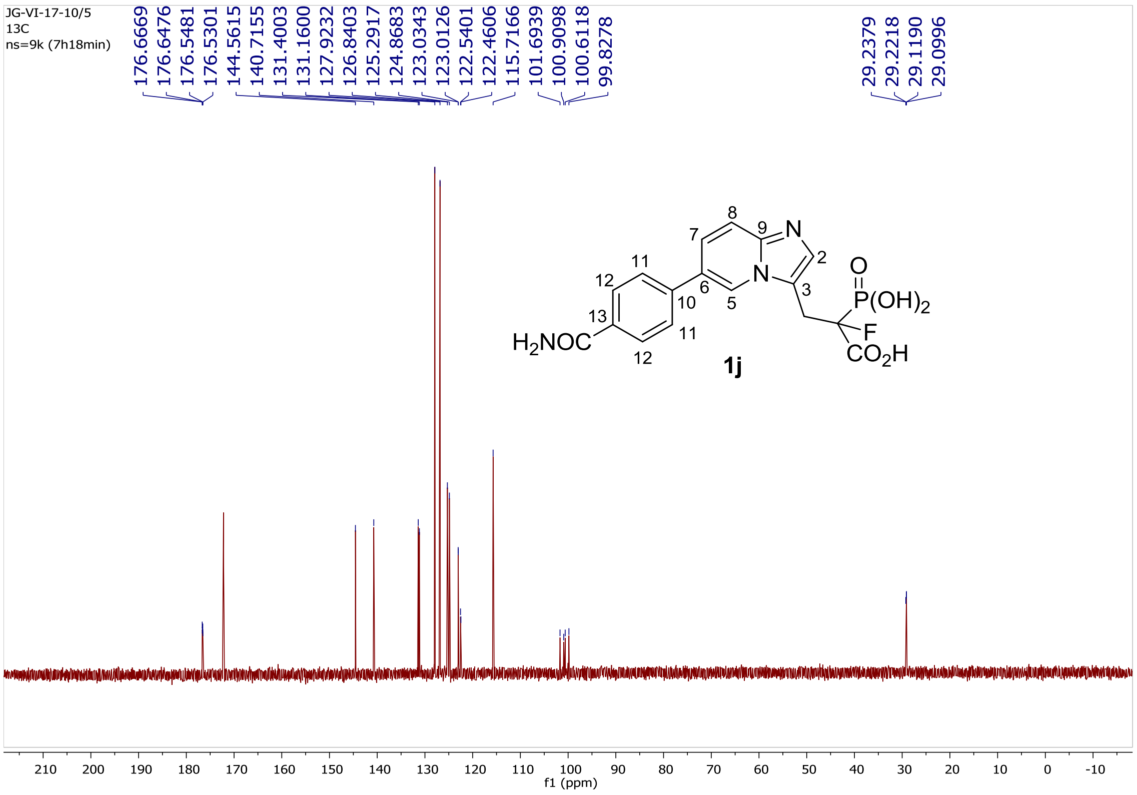

Figure S31. ^{13}C NMR of compound **1j** (176 MHz, D_2O pH 7).

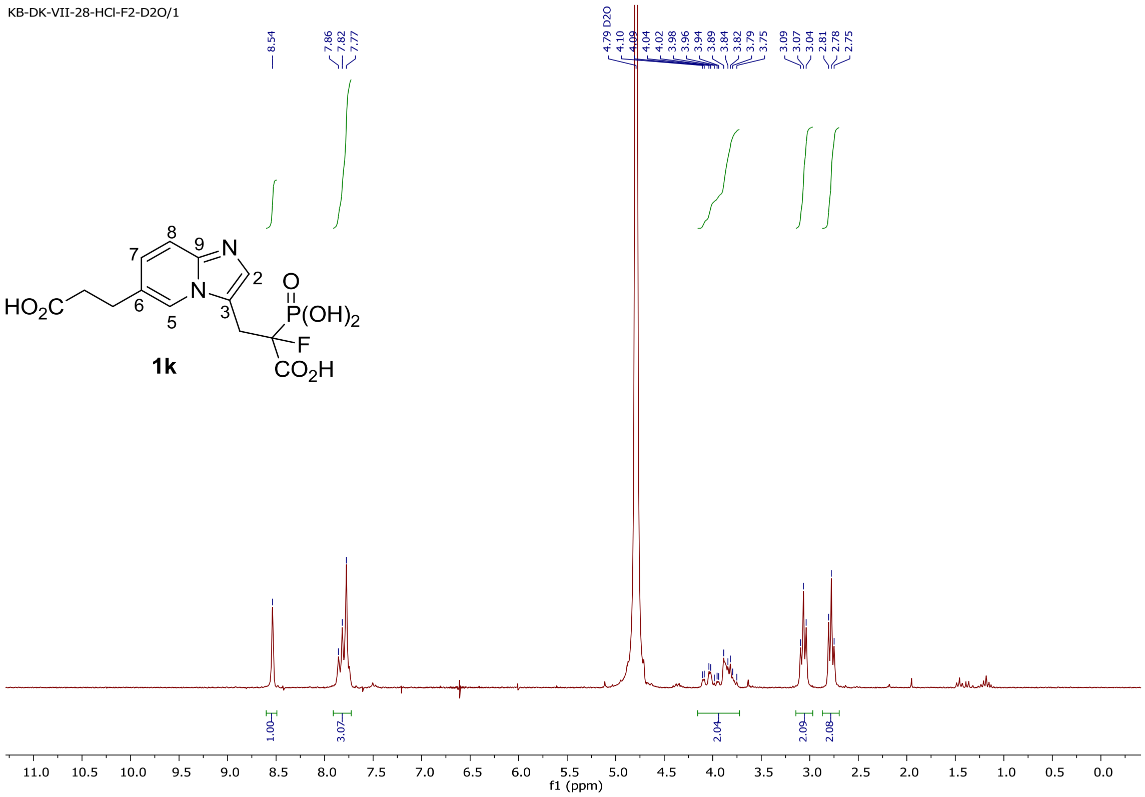

Figure S32. ^{1}H NMR of compound **1k** (700 MHz, D_2O pH 8).

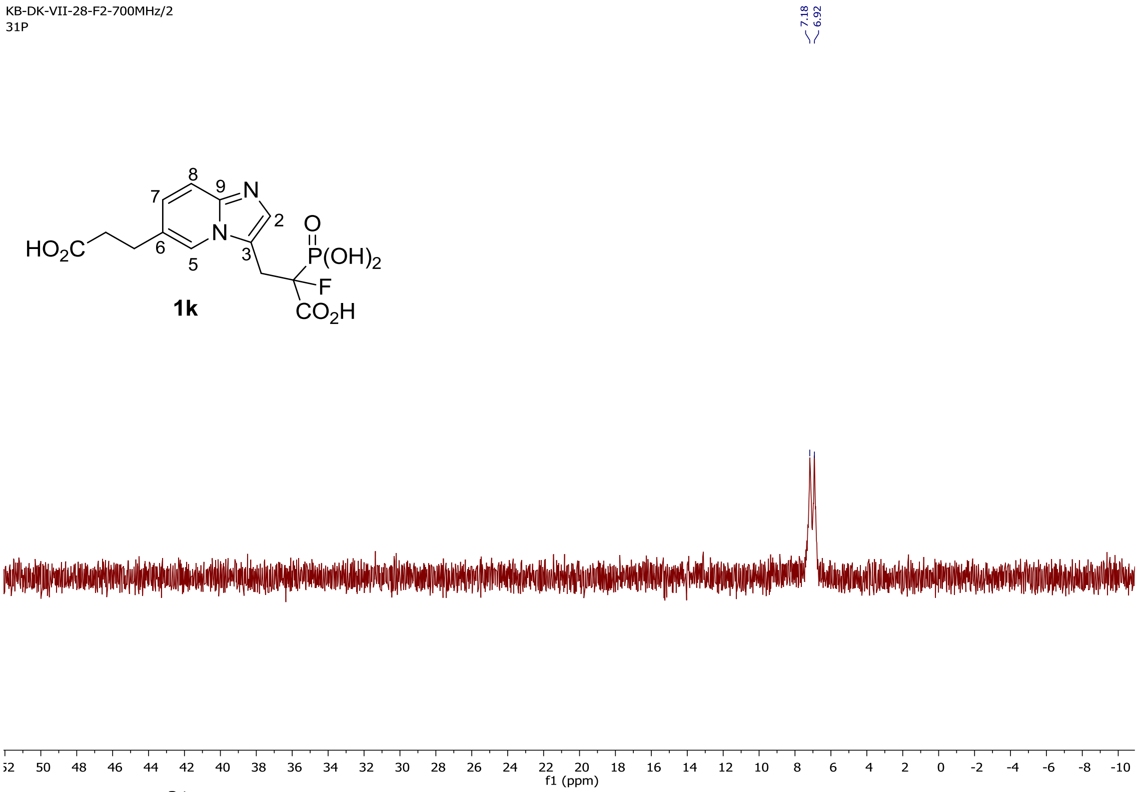

Figure S33. ^{31}P NMR of compound **1k** (284 MHz, D_2O pH 8).

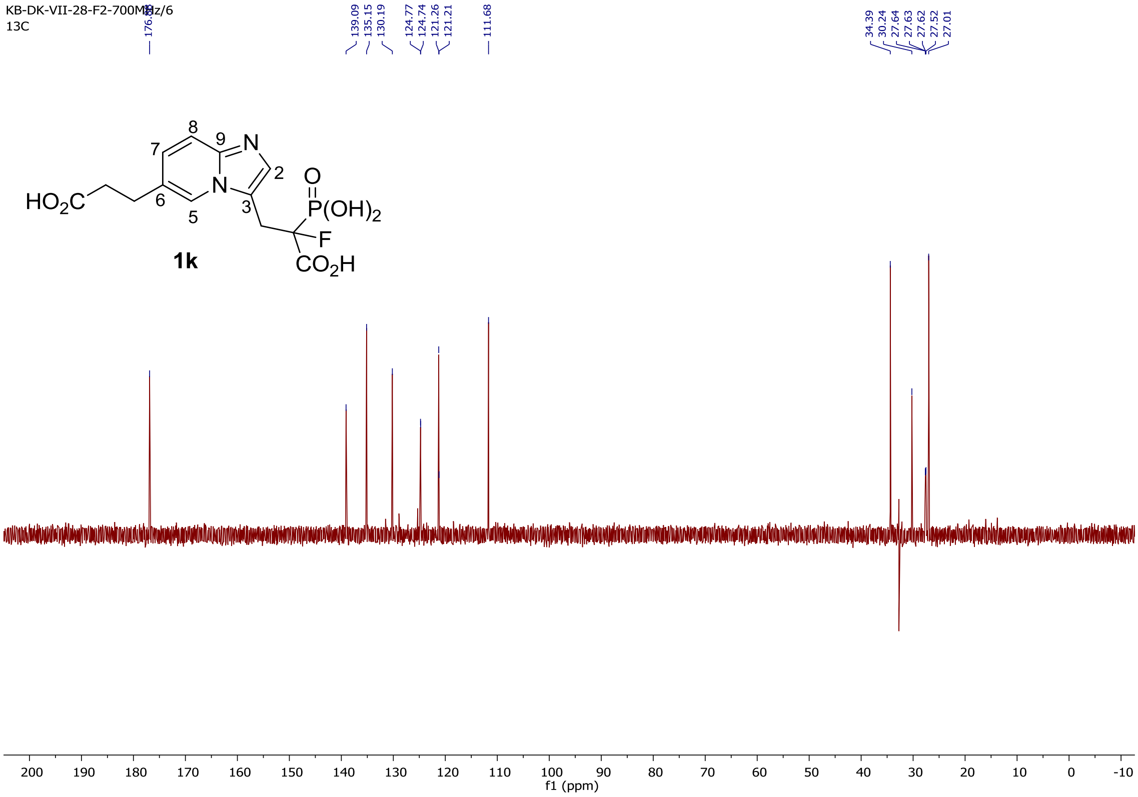

Figure S34. ^{13}C NMR of compound **1k** (176 MHz, D_2O pH 8).

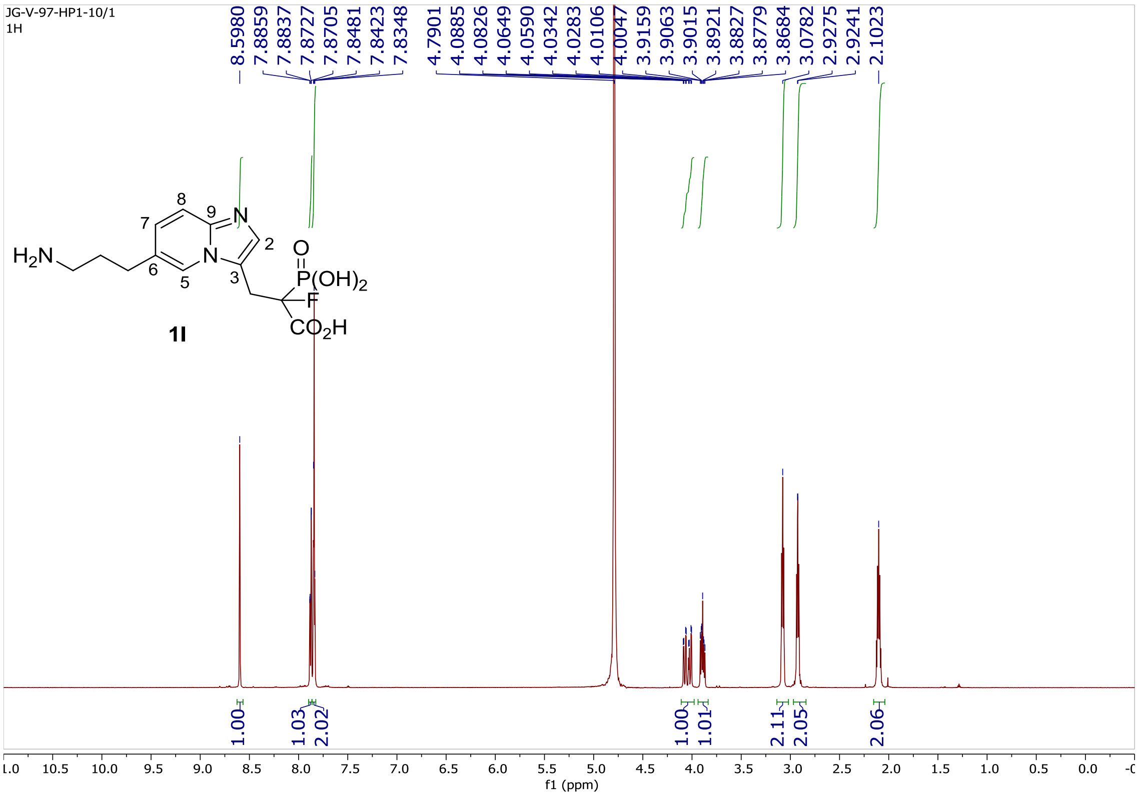

Figure S35.^{1}H NMR of compound **1l** (700 MHz, D_2O pH 2).

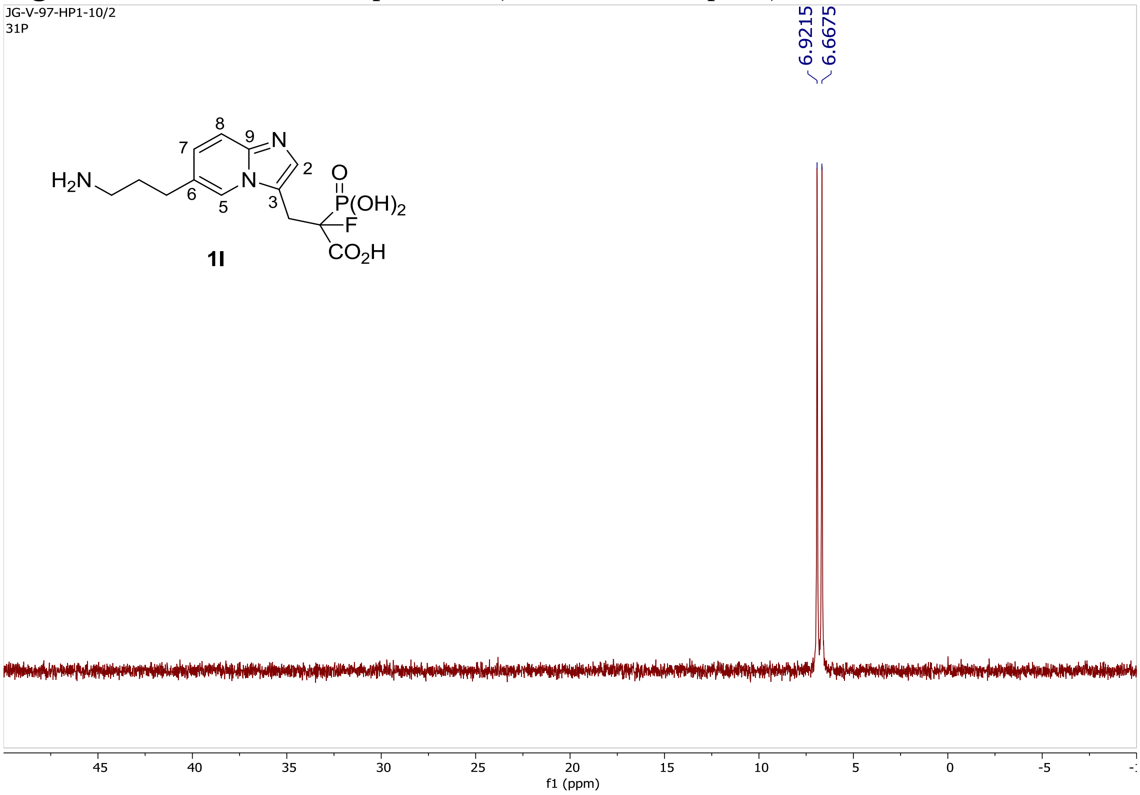

Figure S36.^{31}P NMR of compound **1l** (283 MHz, D_2O pH 2).

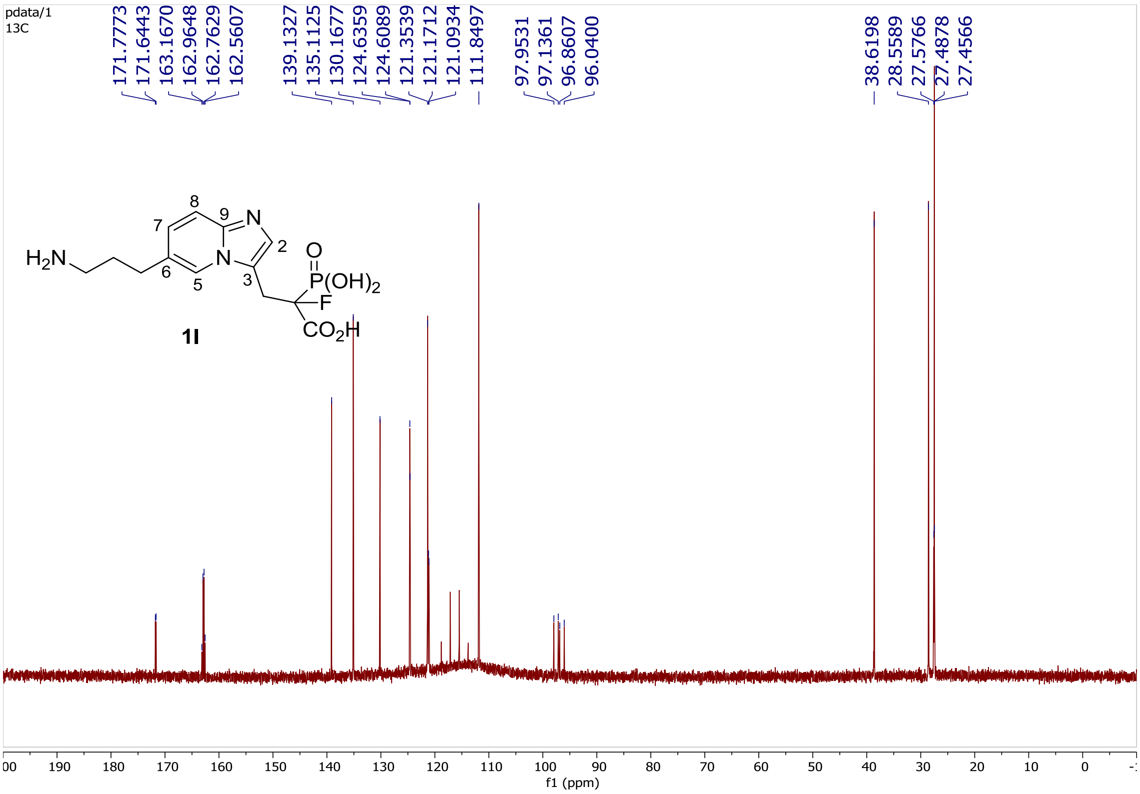

Figure S37.^{13}C NMR of compound **1l** (176 MHz, D_2O pH 2).

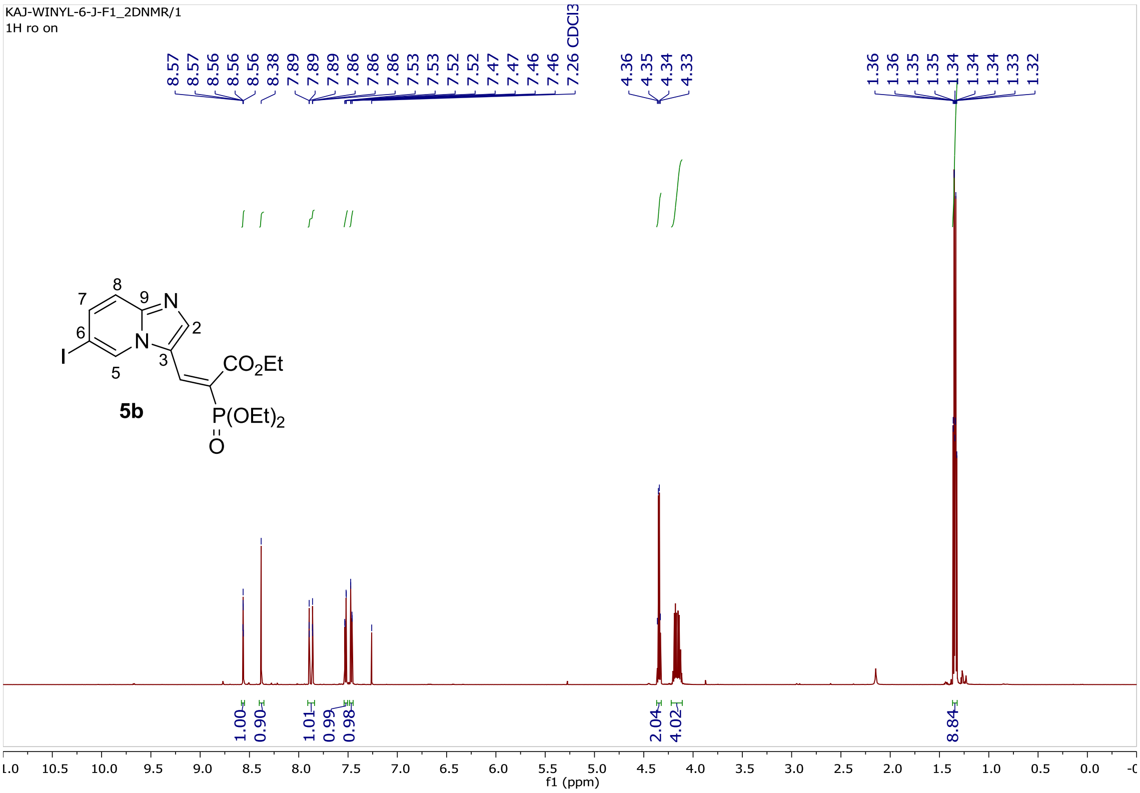

Figure S38. ^{1}H NMR of compound **5b** (700 MHz, $CDCl_3$).

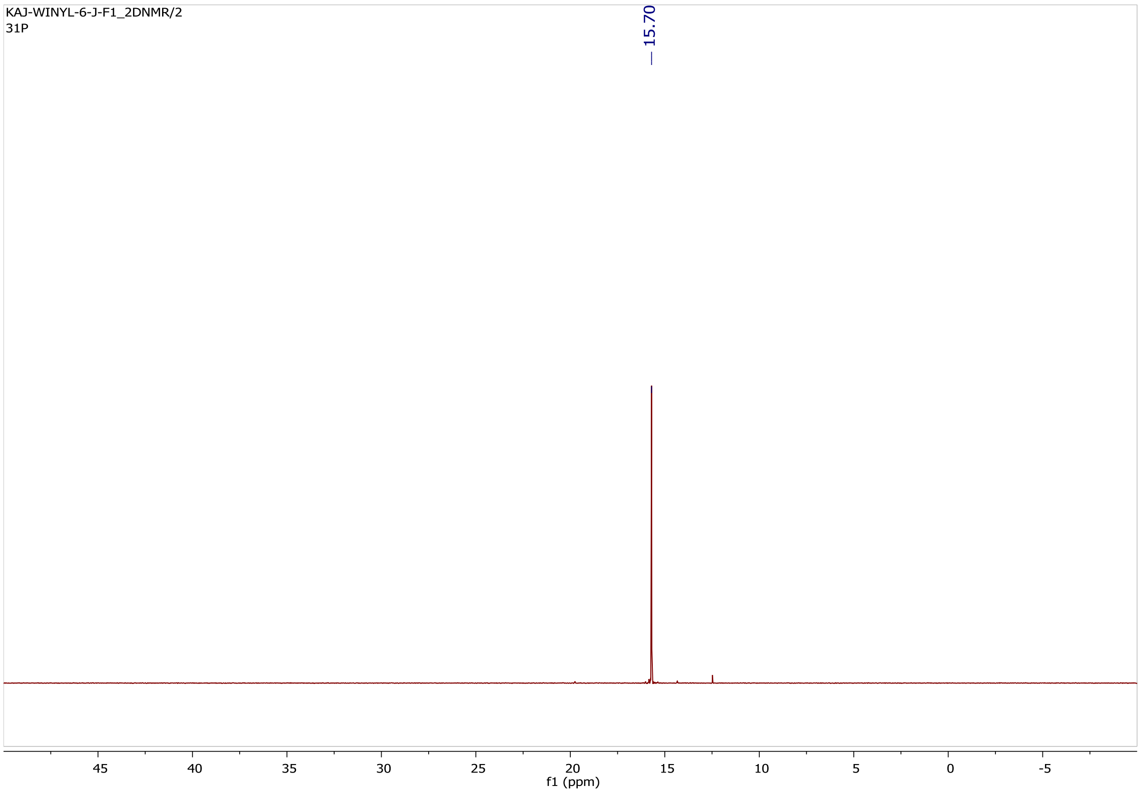

Figure S39. ^{31}P NMR of compound **5b** (283 MHz, $CDCl_3$).

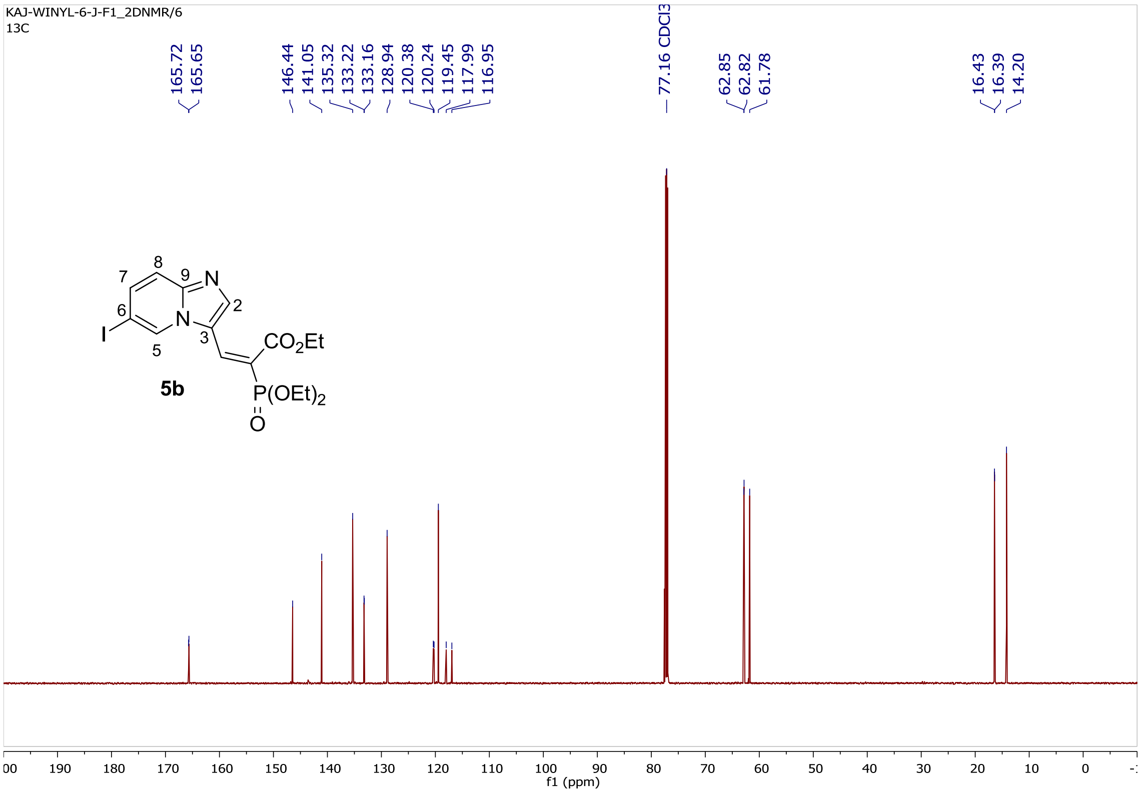

Figure S40. ^{13}C NMR of compound **5b** (176 MHz, $CDCl_3$).

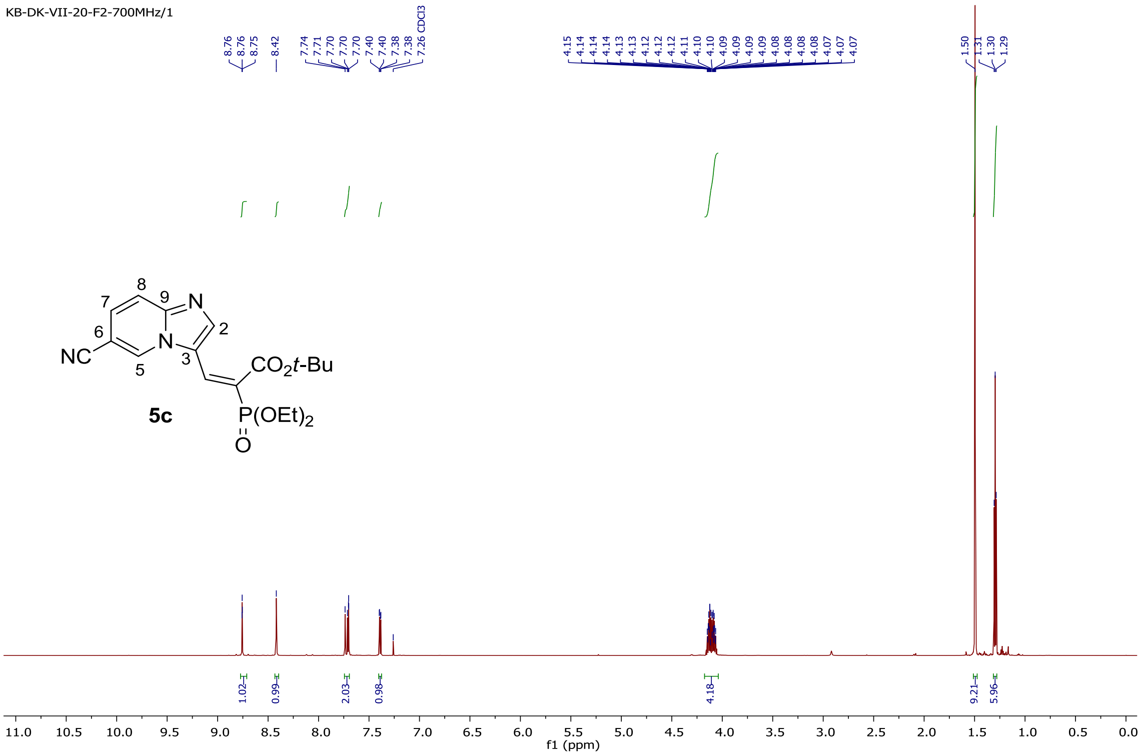

Figure S41. ^{1}H NMR of compound **5c** (700 MHz, $CDCl_3$).

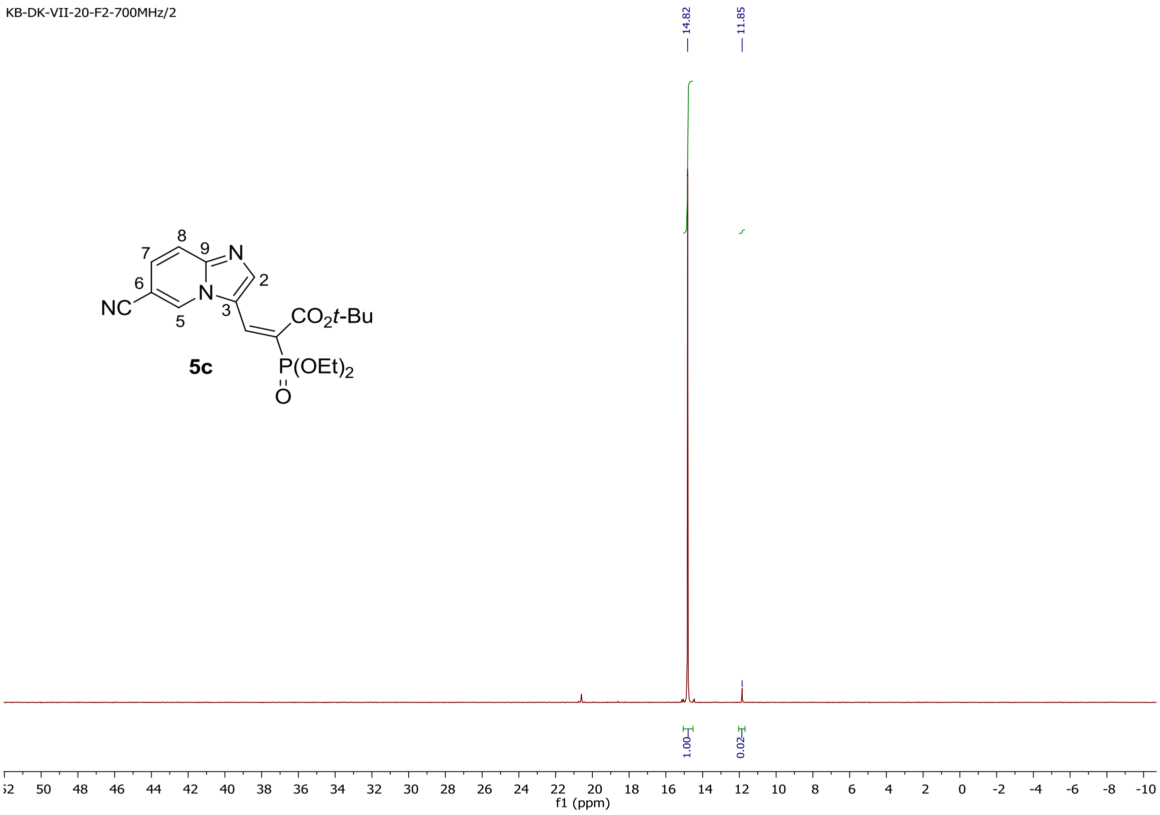

Figure S42. ^{31}P NMR of compound **5c** (283 MHz, $CDCl_3$).

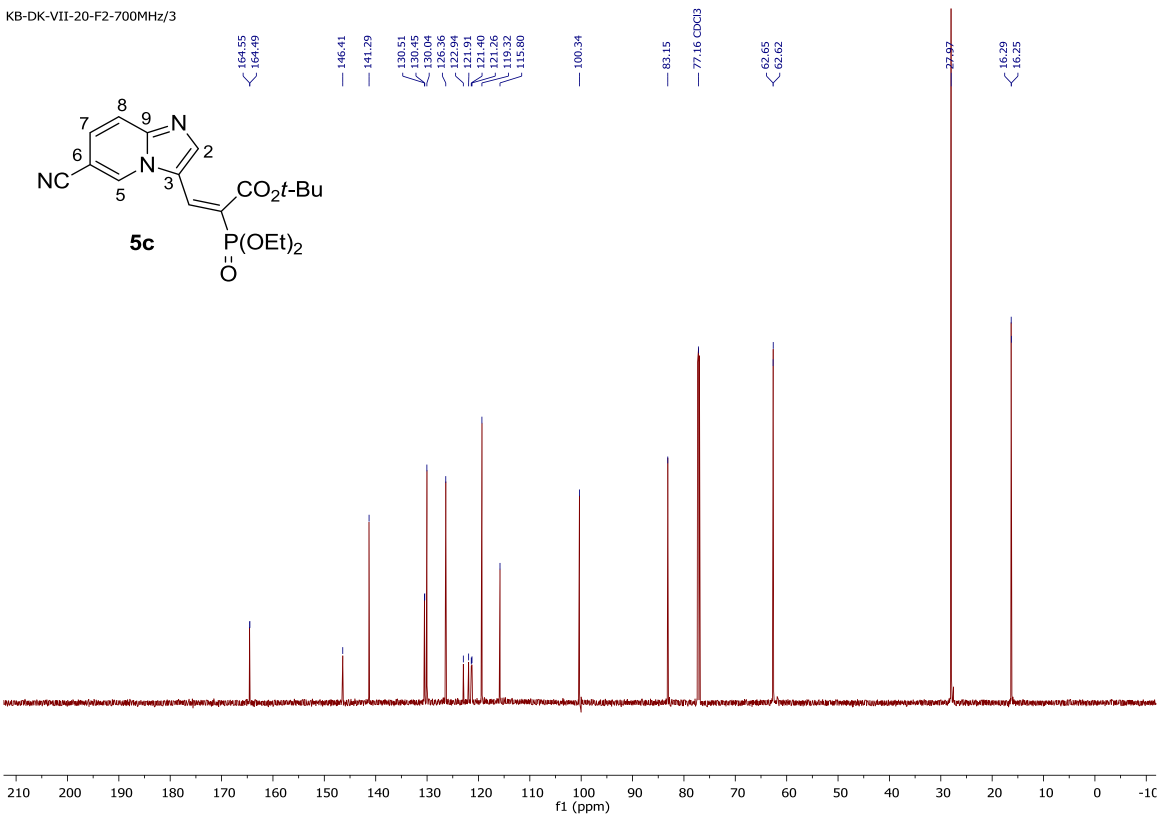

Figure S43. ^{13}C NMR of compound **5c** (176 MHz, $CDCl_3$).

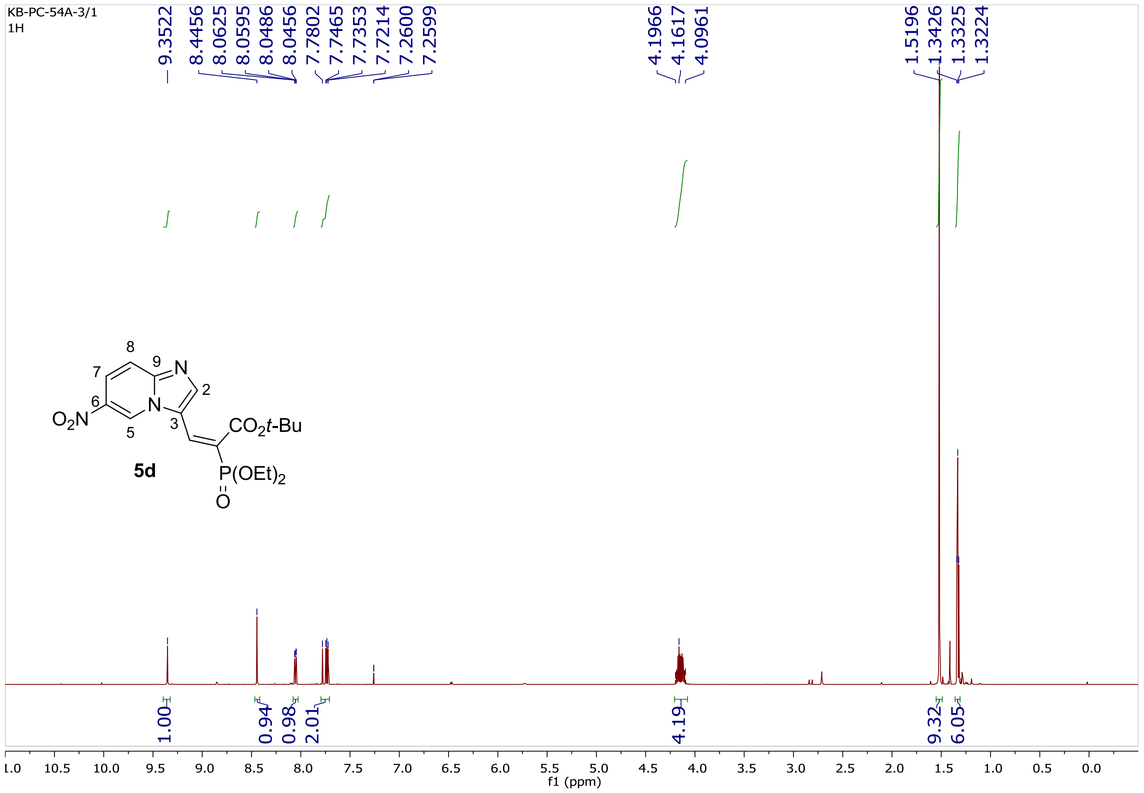

Figure S44. ^{1}H NMR of compound **5d** (700 MHz, $CDCl_3$).

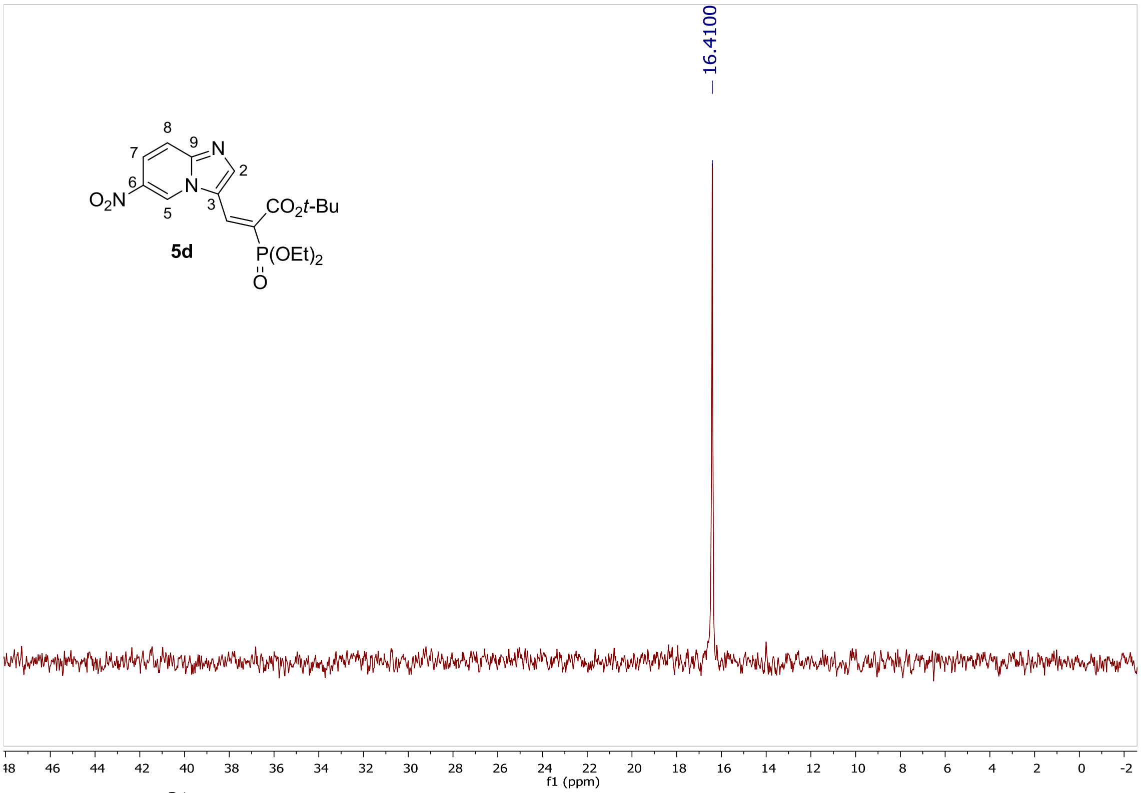

Figure S45. ^{31}P NMR of compound **5d** (101 MHz, $CDCl_3$).

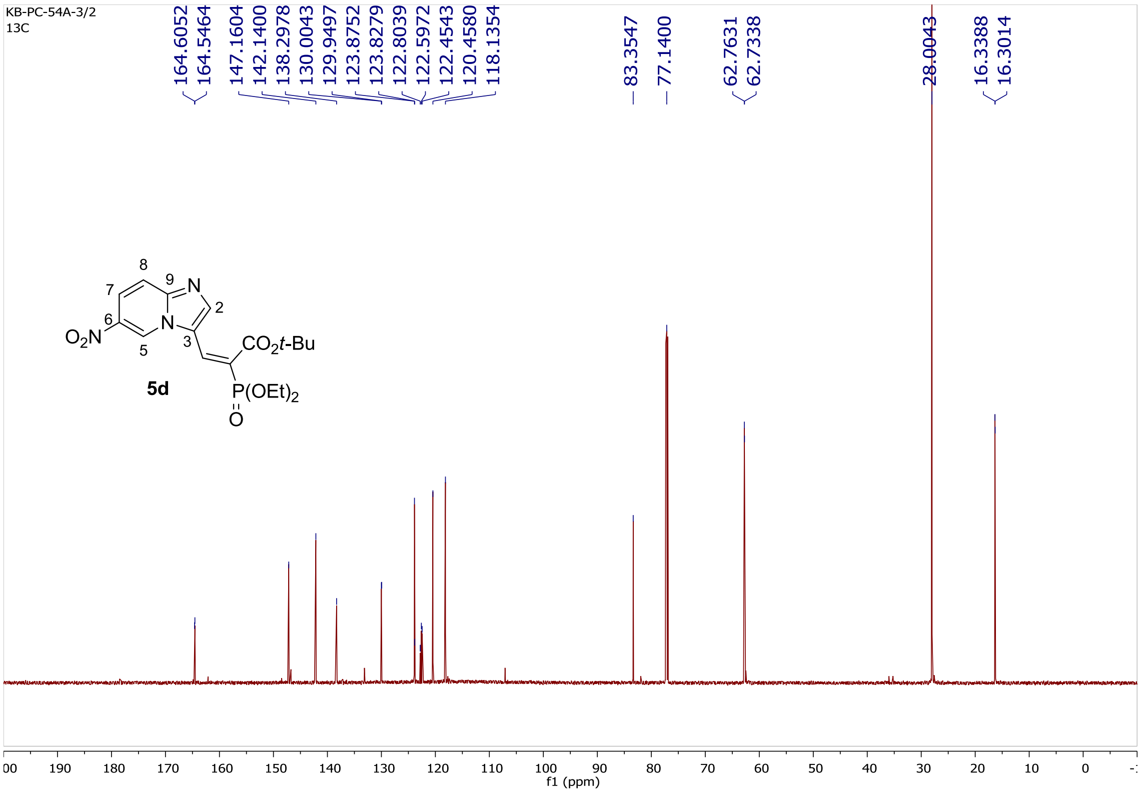

Figure S46. ^{13}C NMR of compound **5d** (176 MHz, $CDCl_3$).

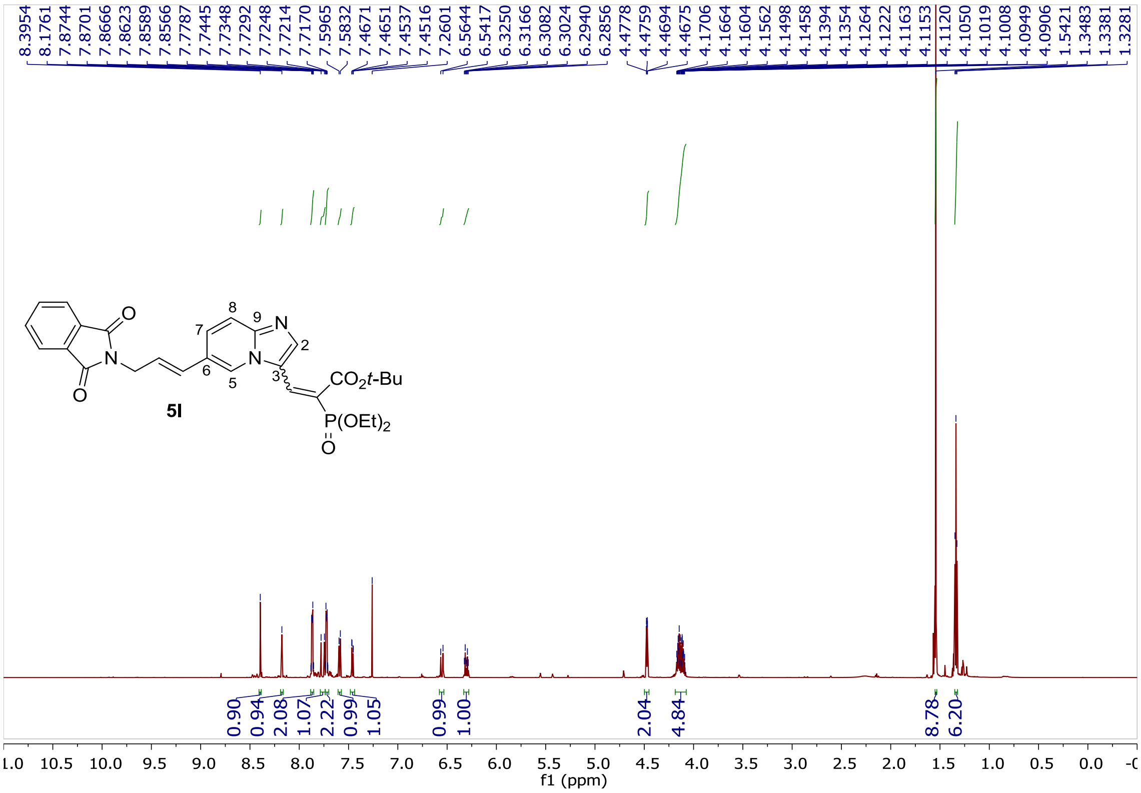

Figure S47. ^{1}H NMR of compound **5l** (700 MHz, $CDCl_3$).

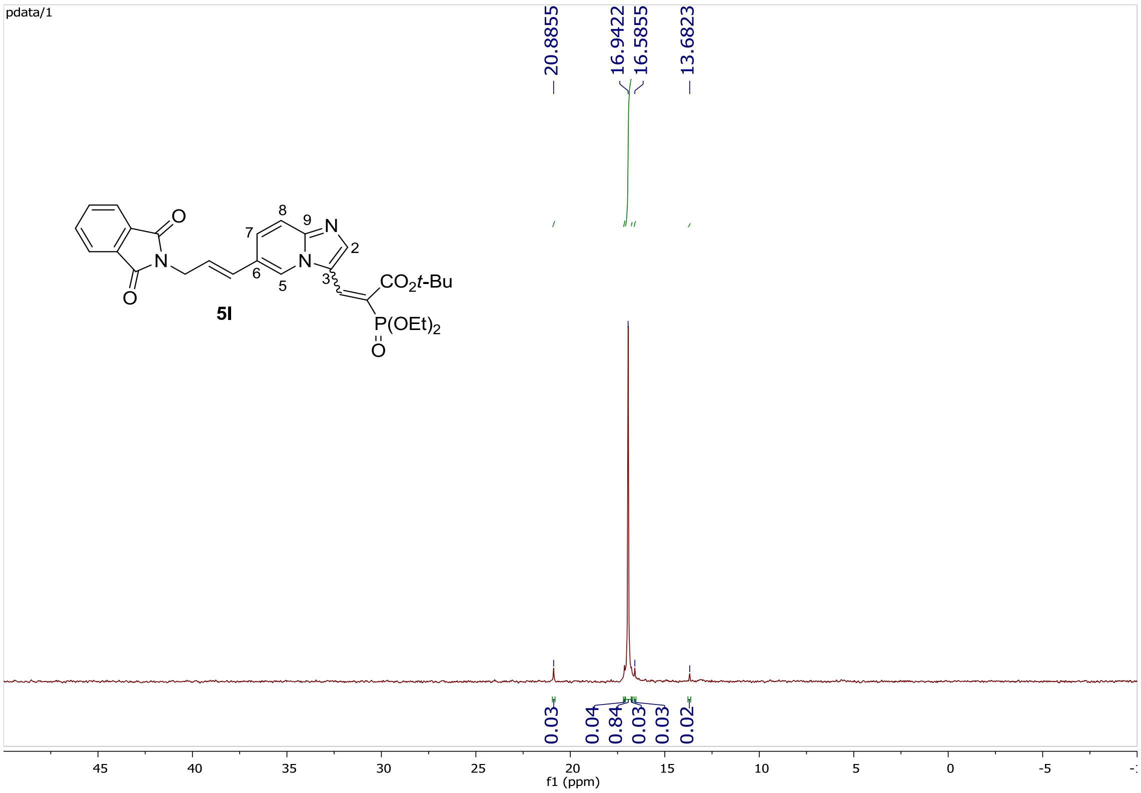

Figure S48. ^{31}P NMR of compound **5l** (283 MHz, $CDCl_3$).

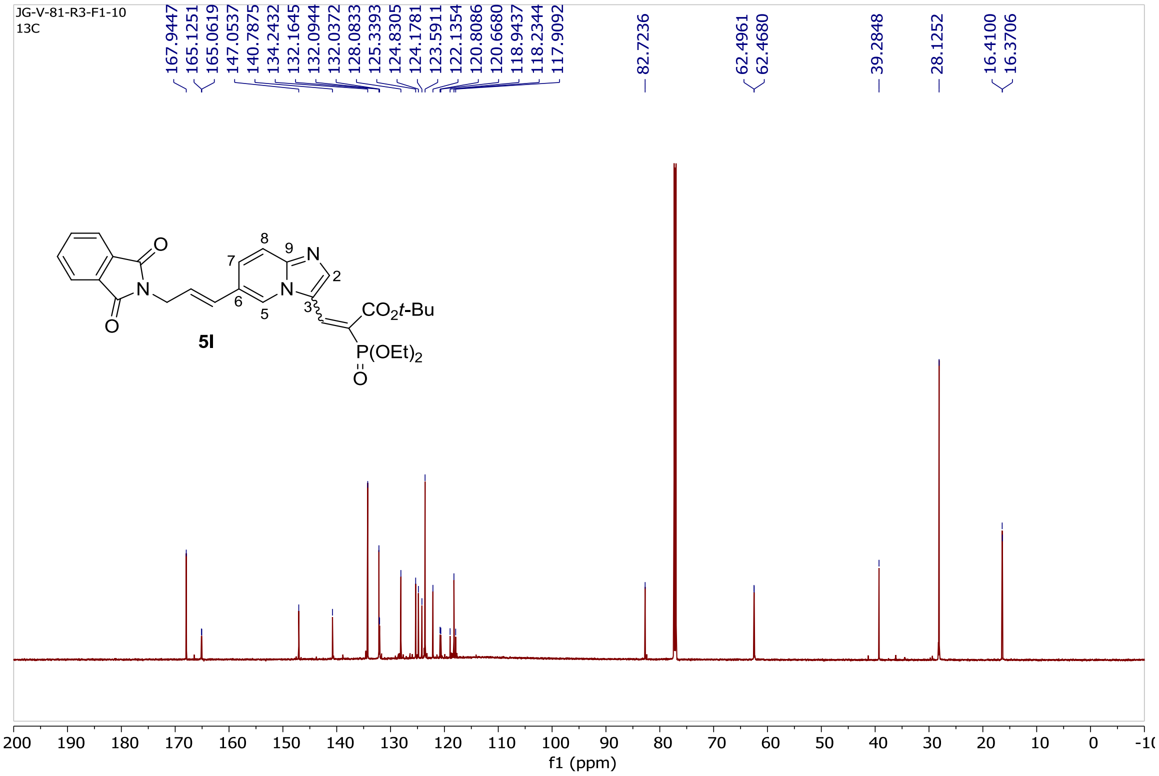

Figure S49. ^{13}C NMR of compound **5l** (176 MHz, $CDCl_3$).

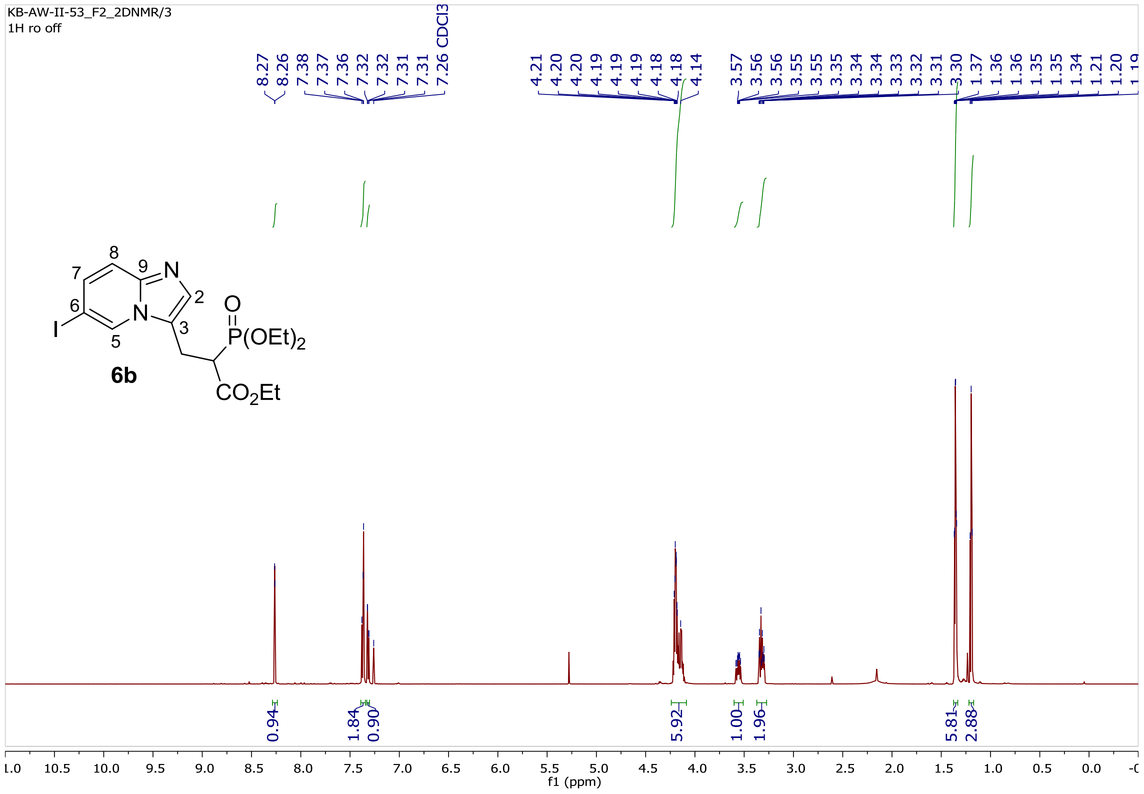

Figure S50. ^{1}H NMR of compound **6b** (700 MHz, $CDCl_3$).

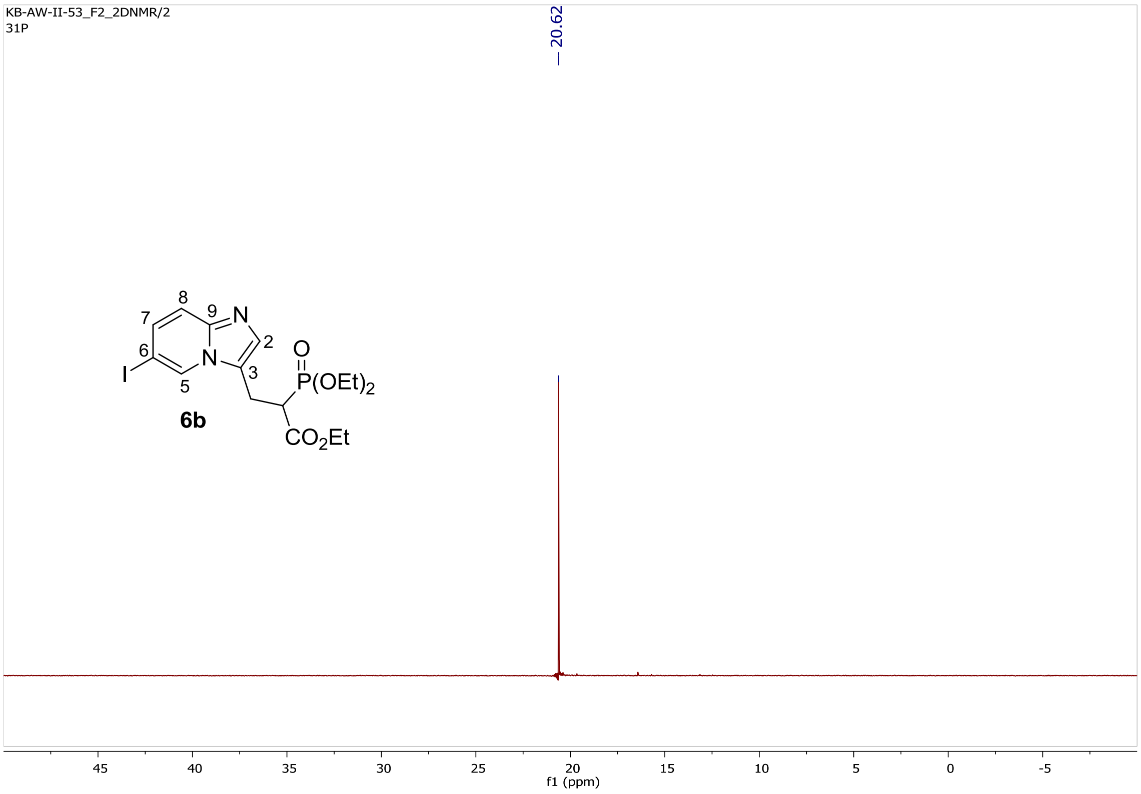

Figure S51. ^{31}P NMR of compound **6b** (283 MHz, $CDCl_3$).

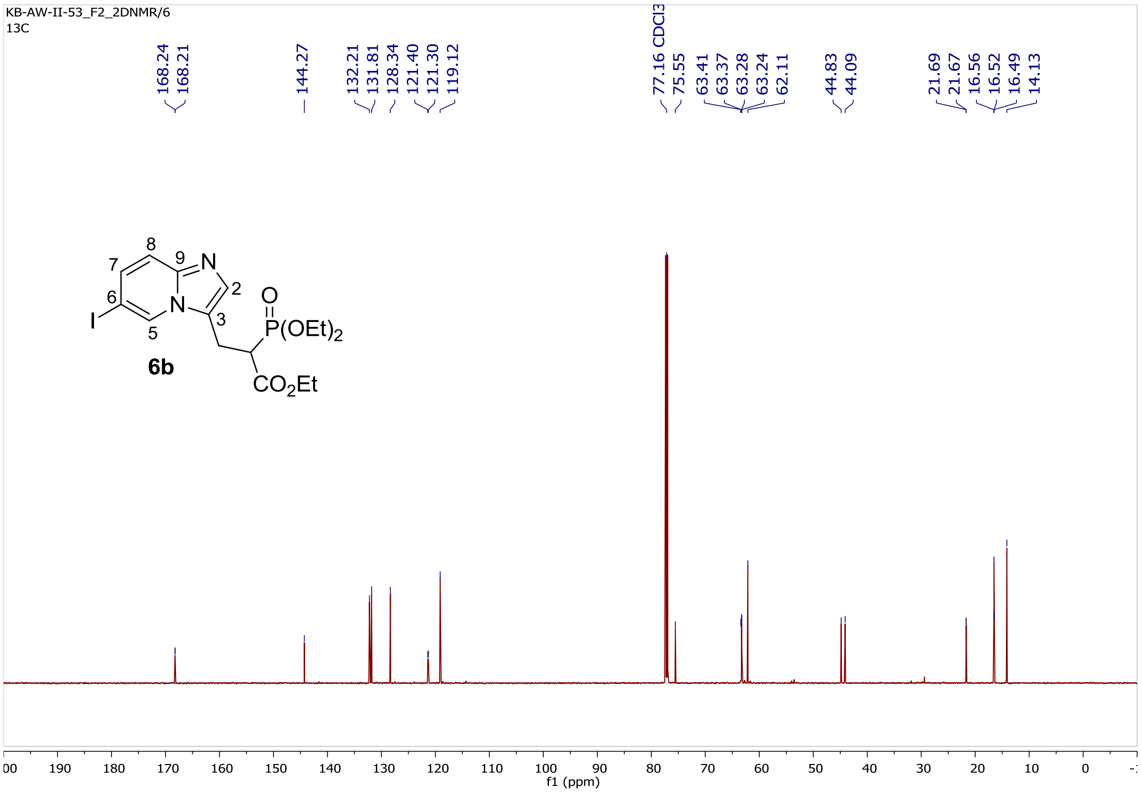

Figure S52. ^{13}C NMR of compound **6b** (176 MHz, $CDCl_3$).

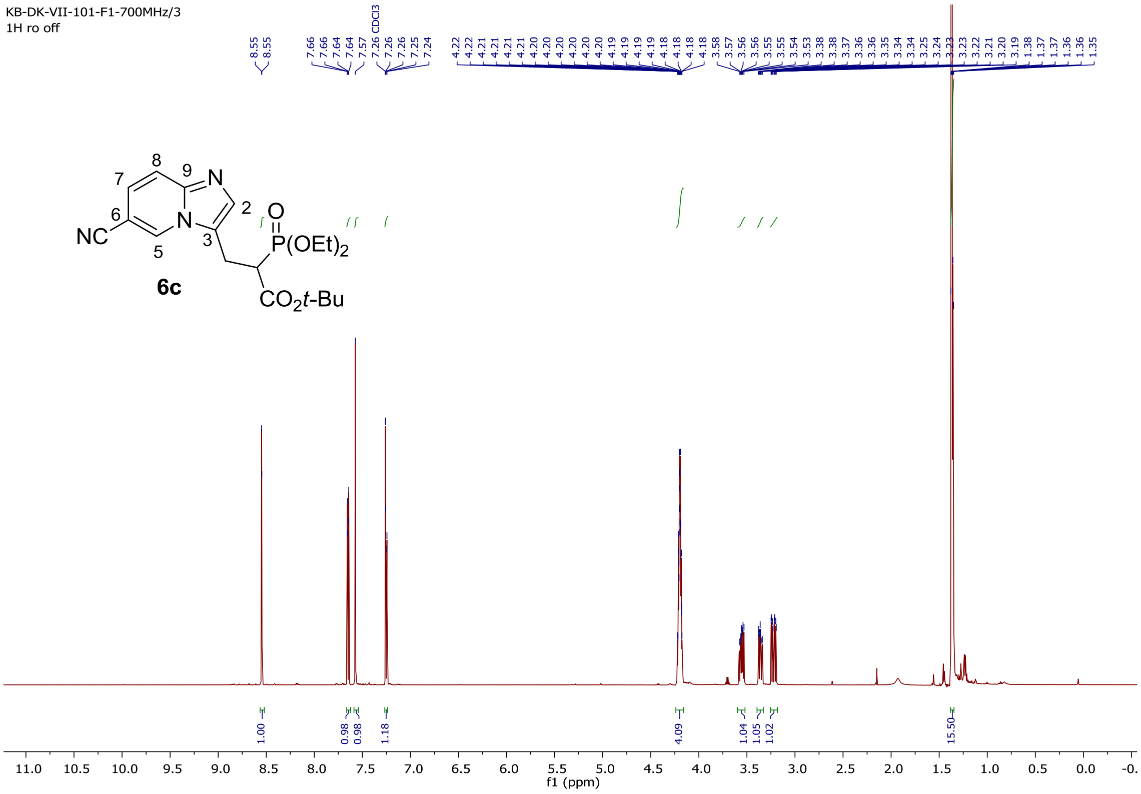

Figure S53. ^{1}H NMR of compound **6c** (700 MHz, $CDCl_3$).

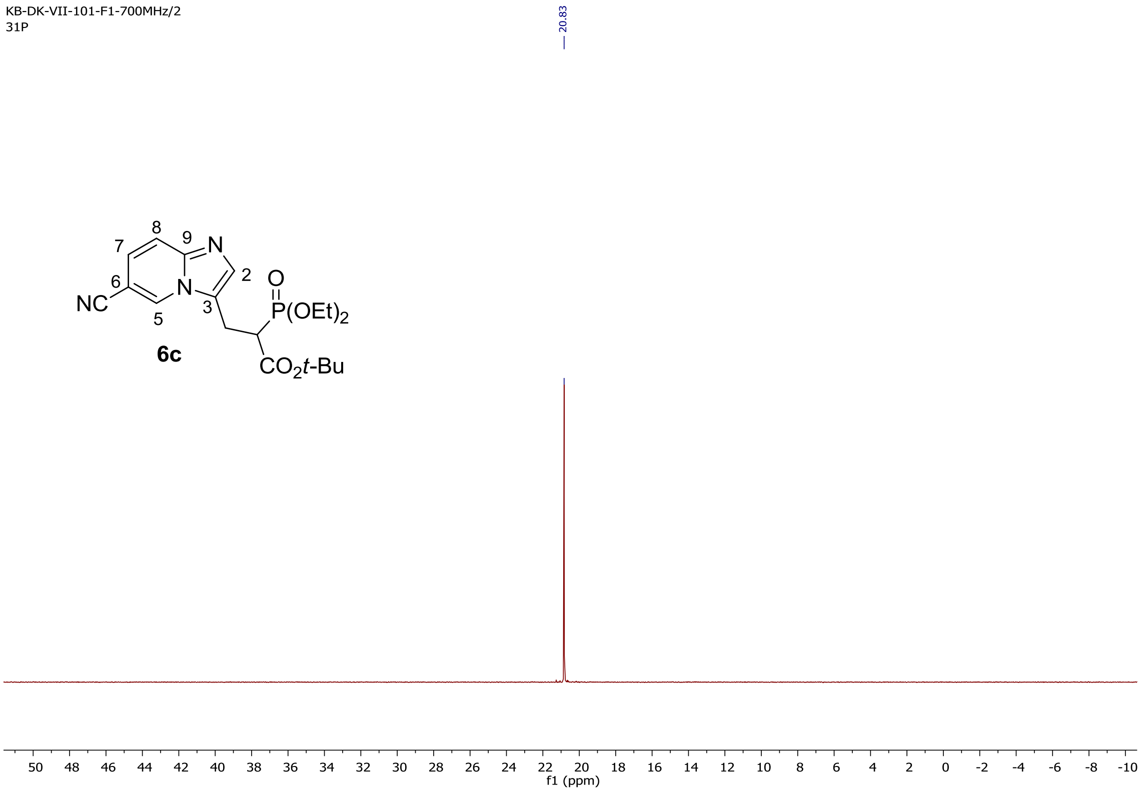

Figure S54. ^{31}P NMR of compound **6c** (283 MHz, $CDCl_3$).

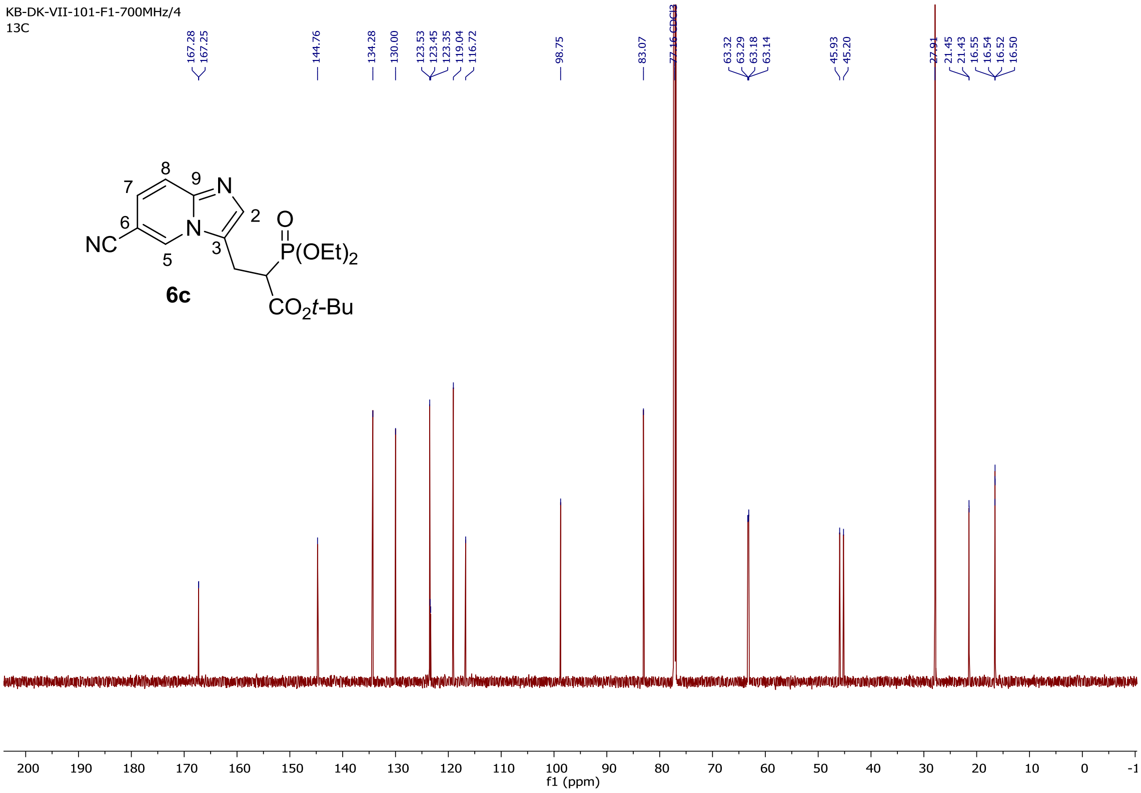

Figure S55. ^{13}C NMR of compound **6c** (176 MHz, $CDCl_3$).

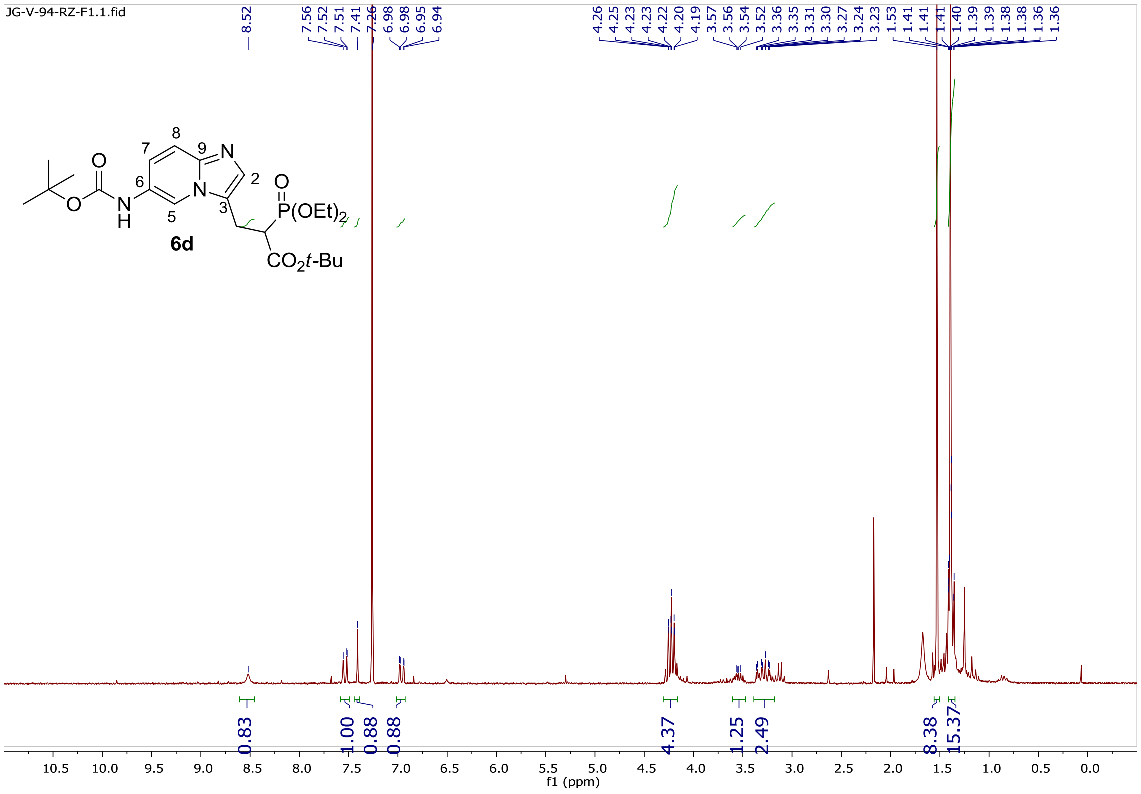

Figure S56. ^{1}H NMR of compound **6d** (250 MHz, $CDCl_3$).

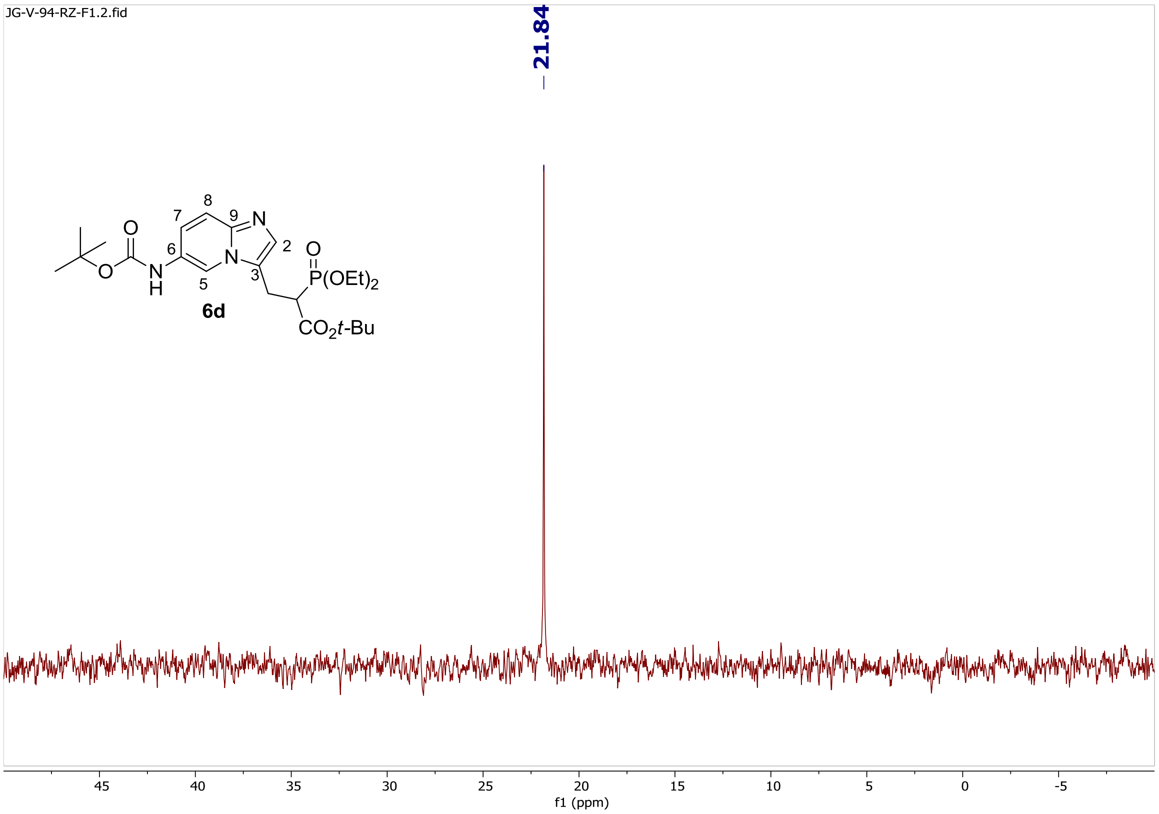

Figure S57. ^{31}P NMR of compound **6d** (101 MHz, $CDCl_3$).

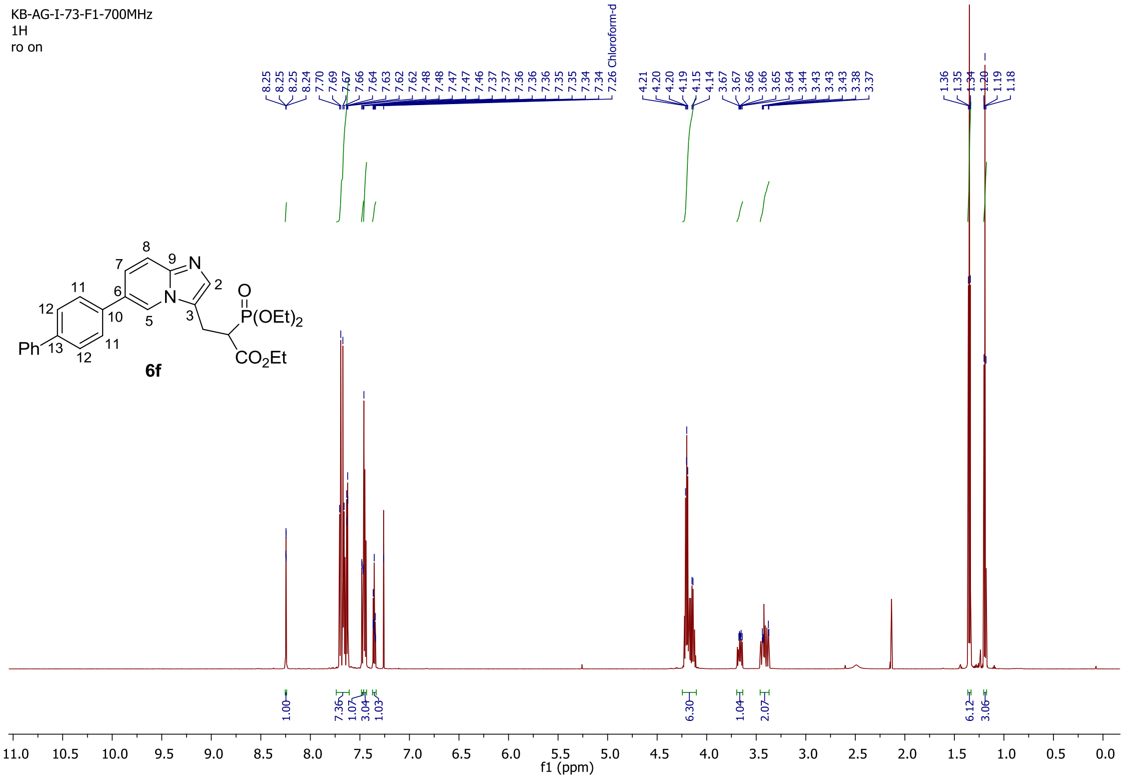

Figure S58. ^{1}H NMR of compound **6f** (700 MHz, $CDCl_3$).

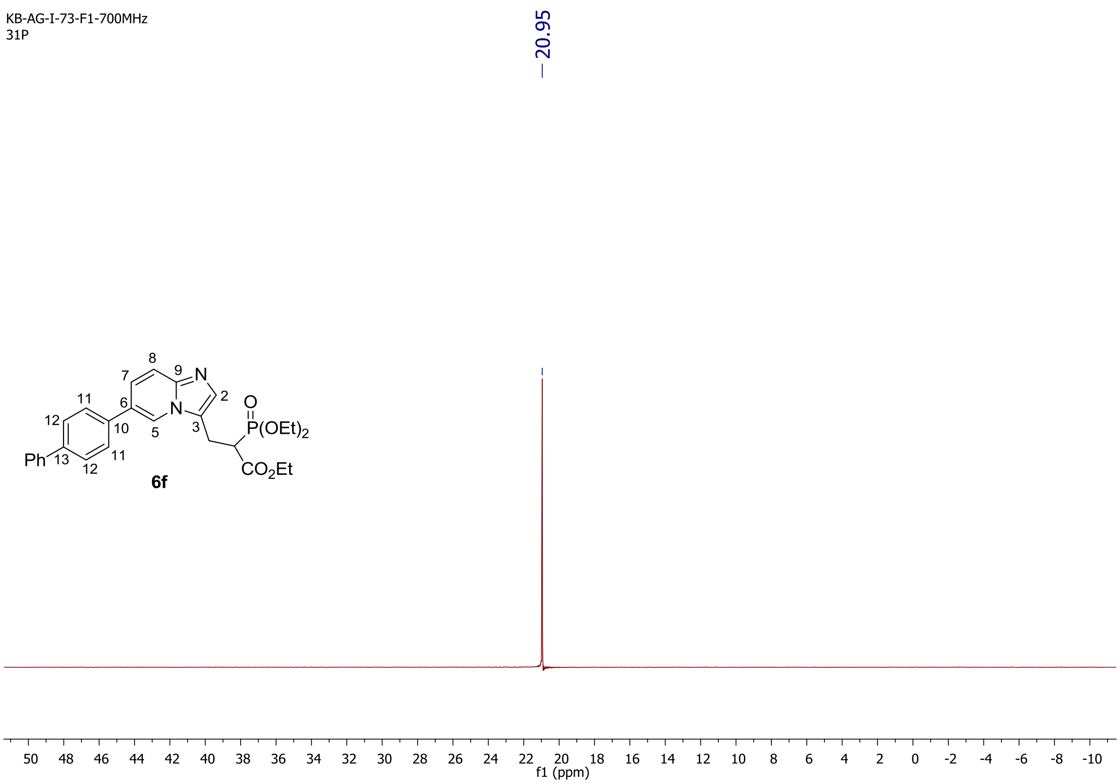

Figure S59. ^{31}P NMR of compound **6f** (101 MHz, $CDCl_3$).

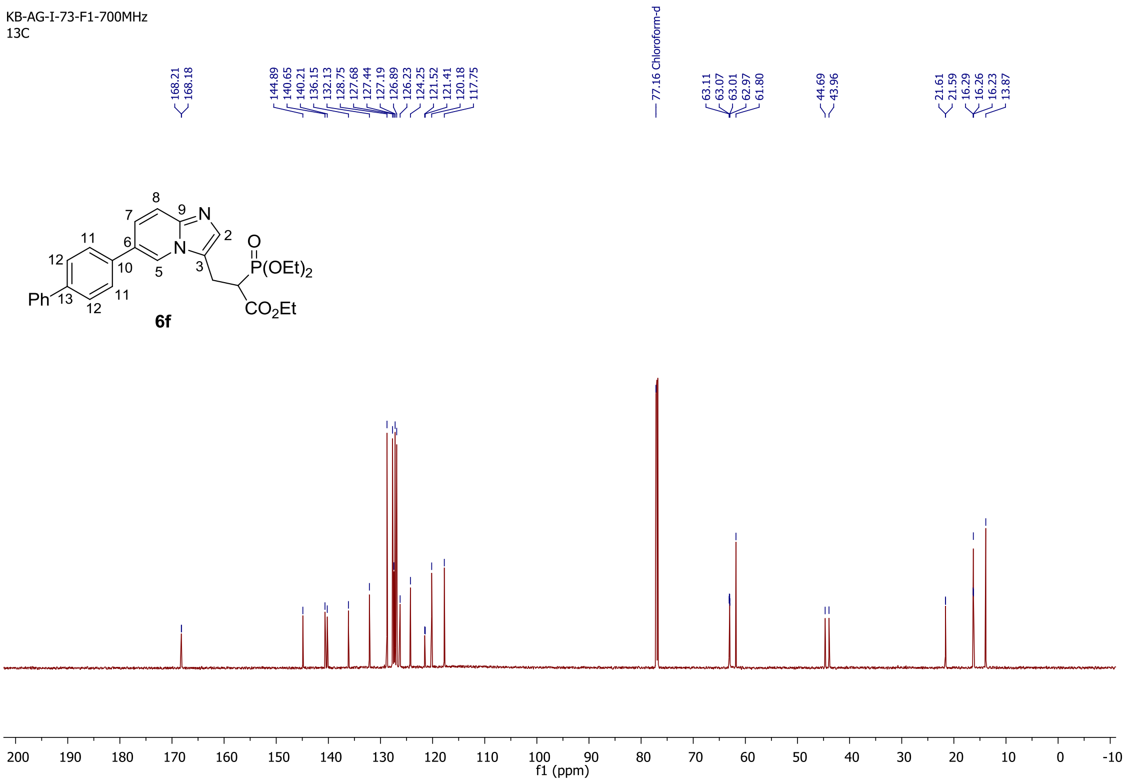

Figure S60. ^{13}C NMR of compound **6f** (176 MHz, $CDCl_3$).

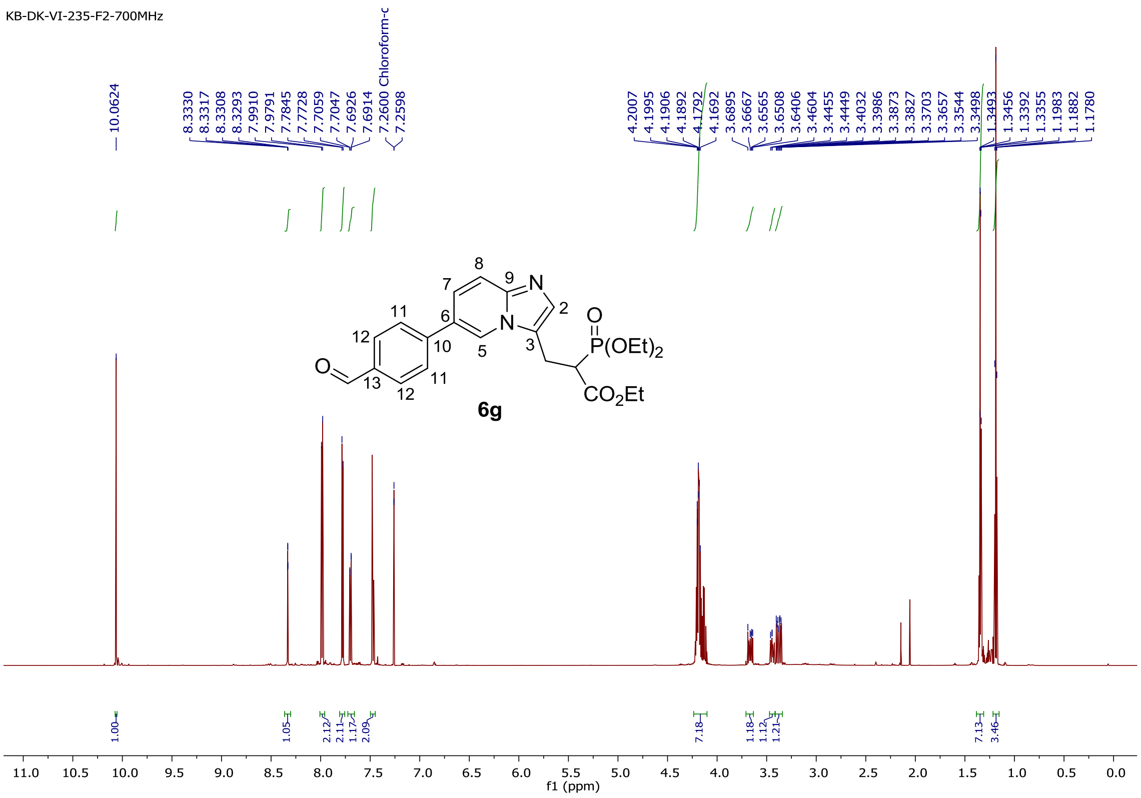

Figure S61. ^{1}H NMR of compound **6g** (700 MHz, $CDCl_3$).

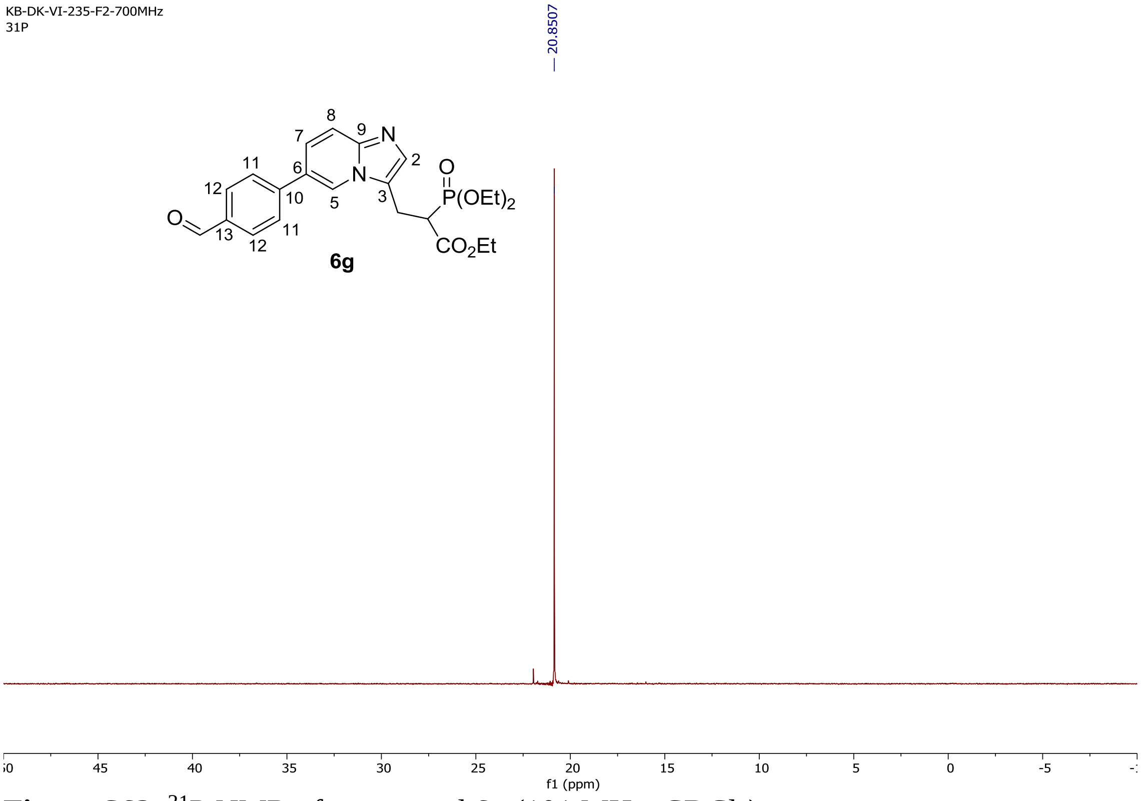

Figure S62. ^{31}P NMR of compound **6g** (101 MHz, $CDCl_3$).

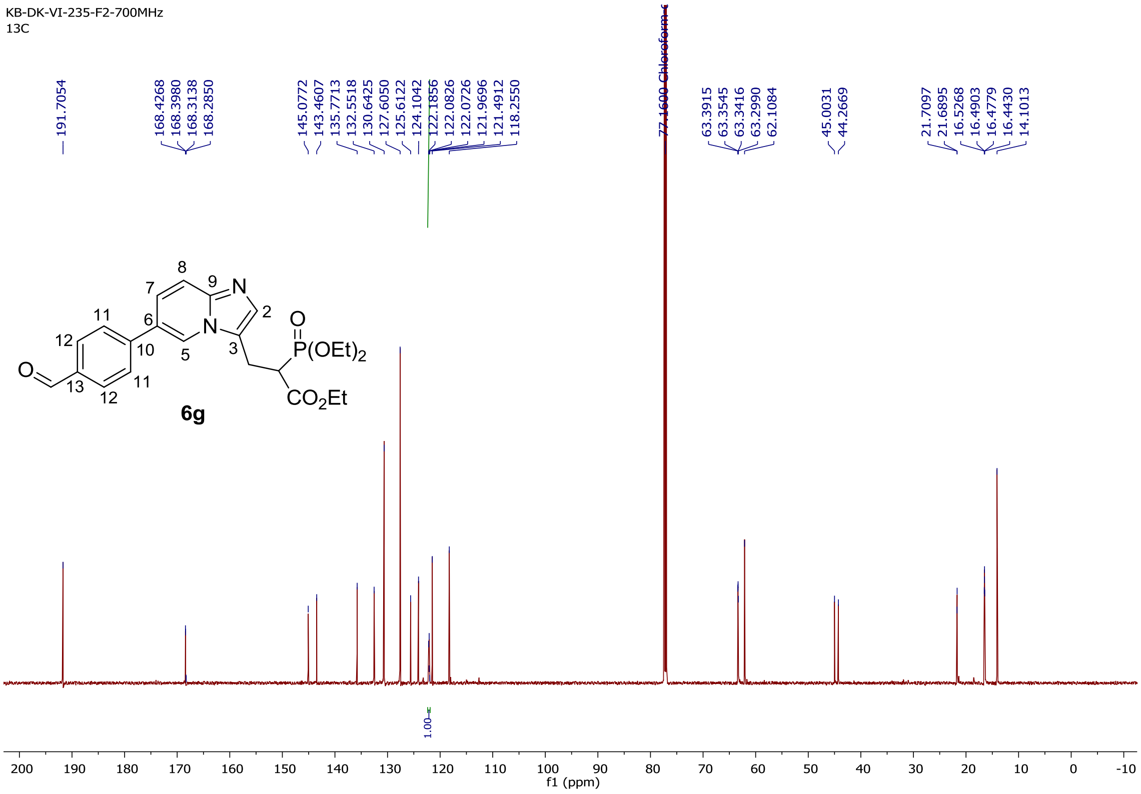

Figure S63. ^{13}C NMR of compound **6g** (176 MHz, $CDCl_3$).

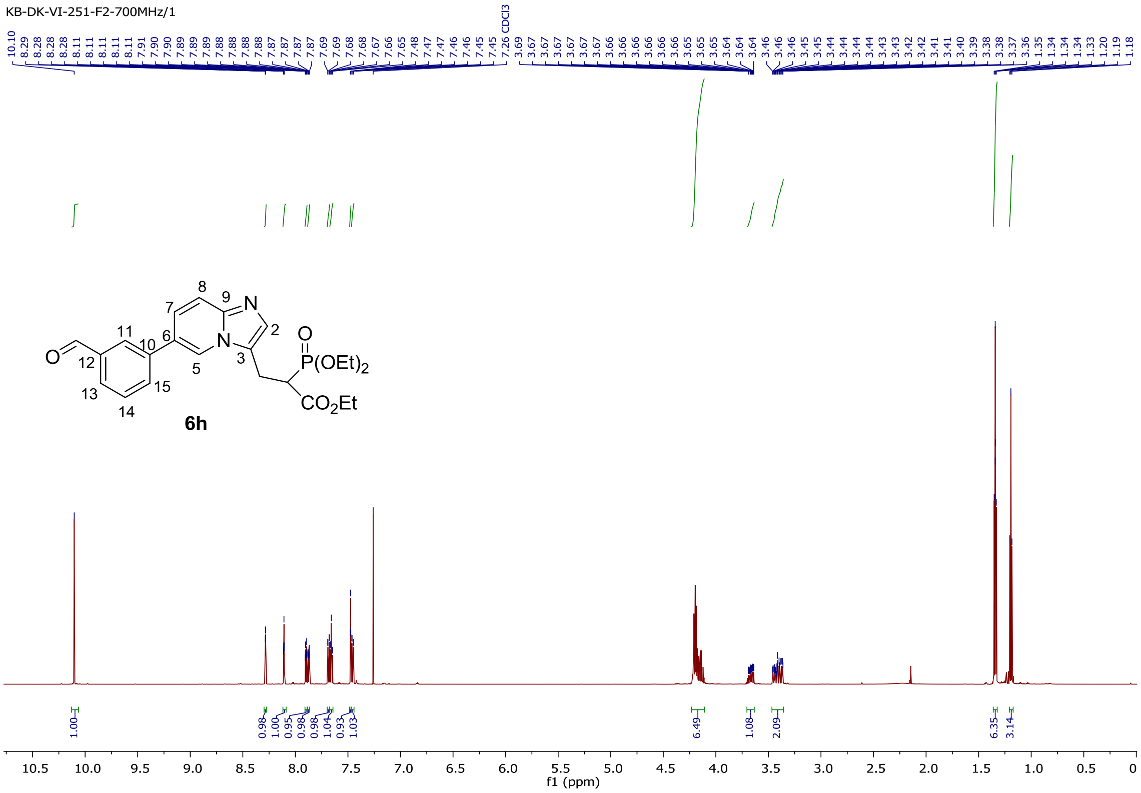

Figure S64. ^{1}H NMR of compound **6h** (700 MHz, $CDCl_3$).

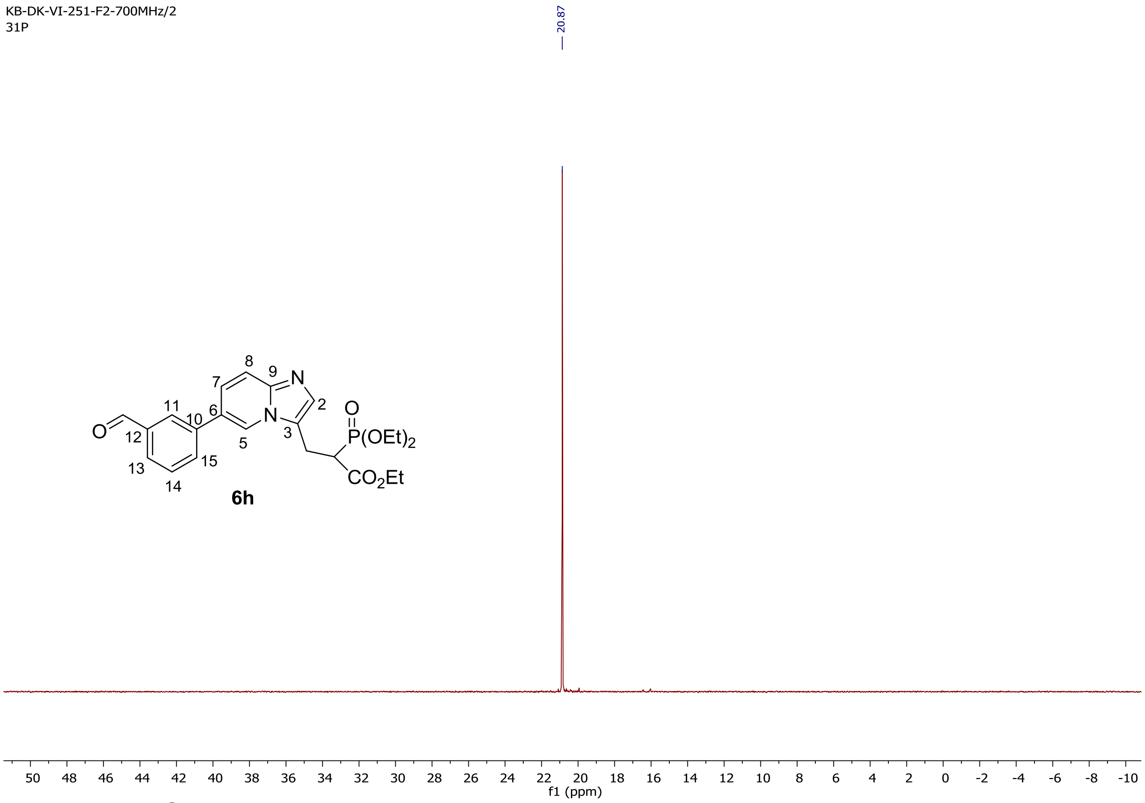

Figure S65. ^{31}P NMR of compound **6h** (283 MHz, $CDCl_3$).

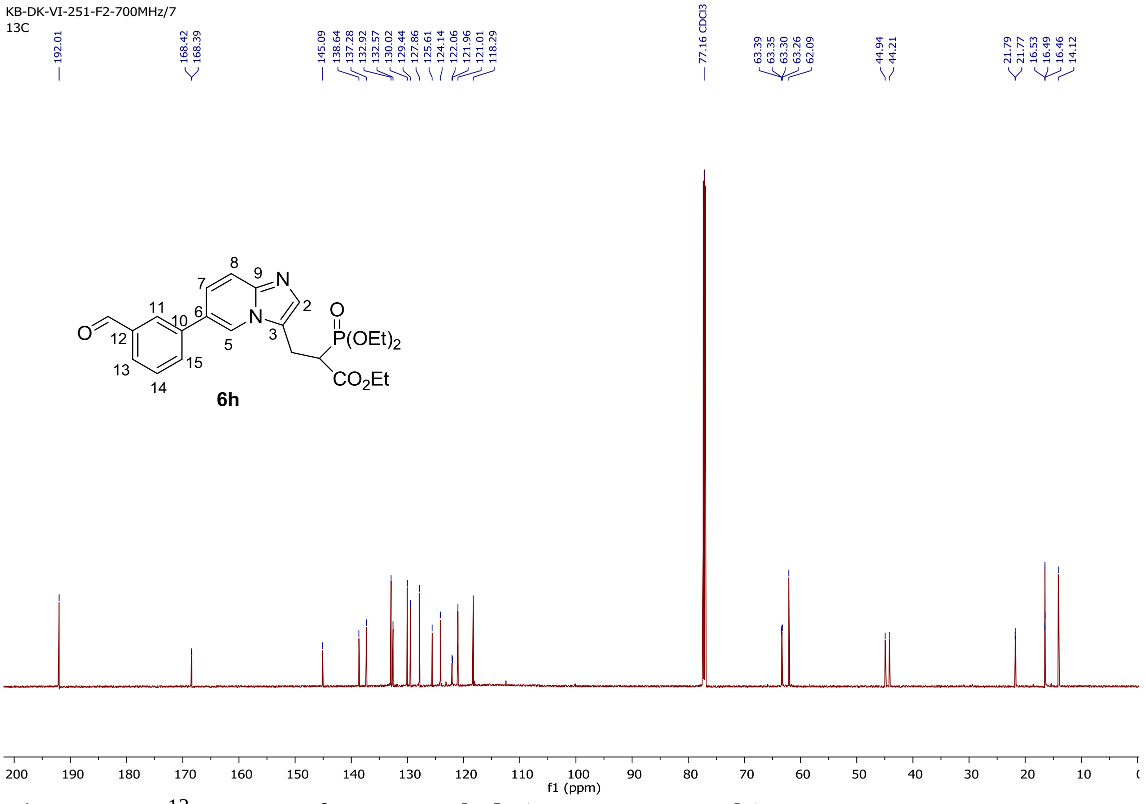

Figure S66. ^{13}C NMR of compound **6h** (176 MHz, $CDCl_3$).

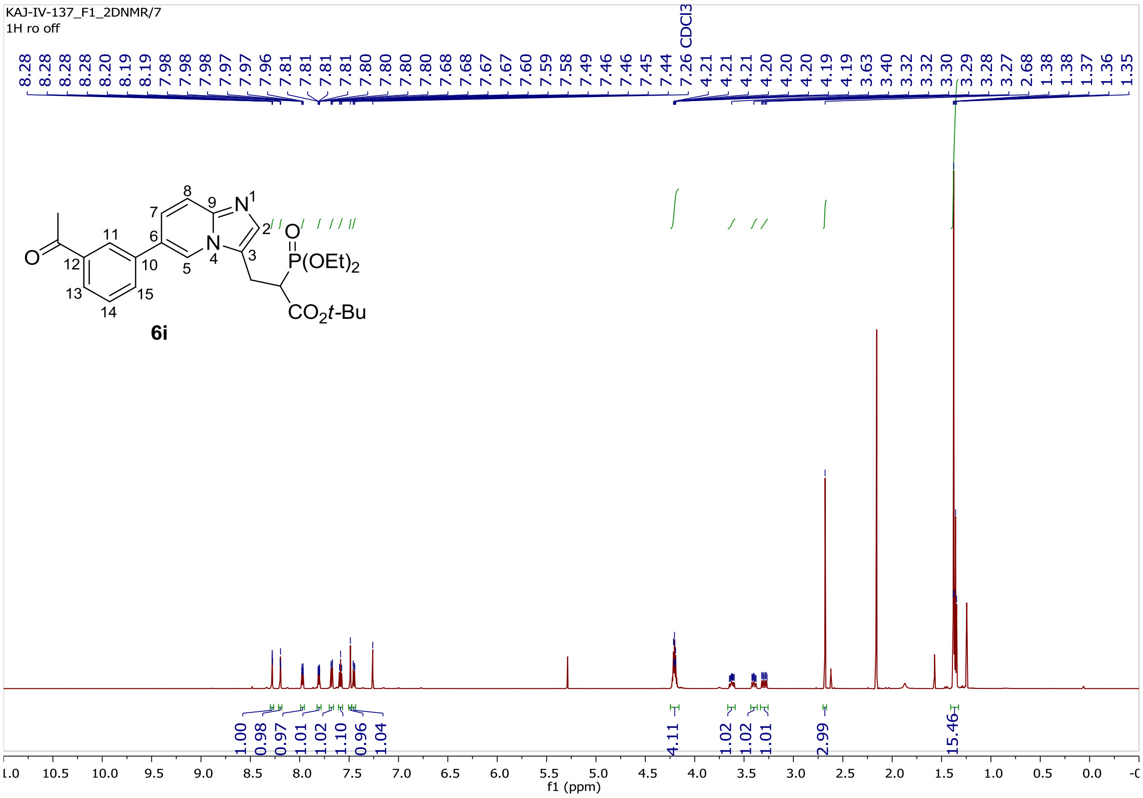

Figure S67. ^{1}H NMR of compound **6i** (700 MHz, $CDCl_3$).

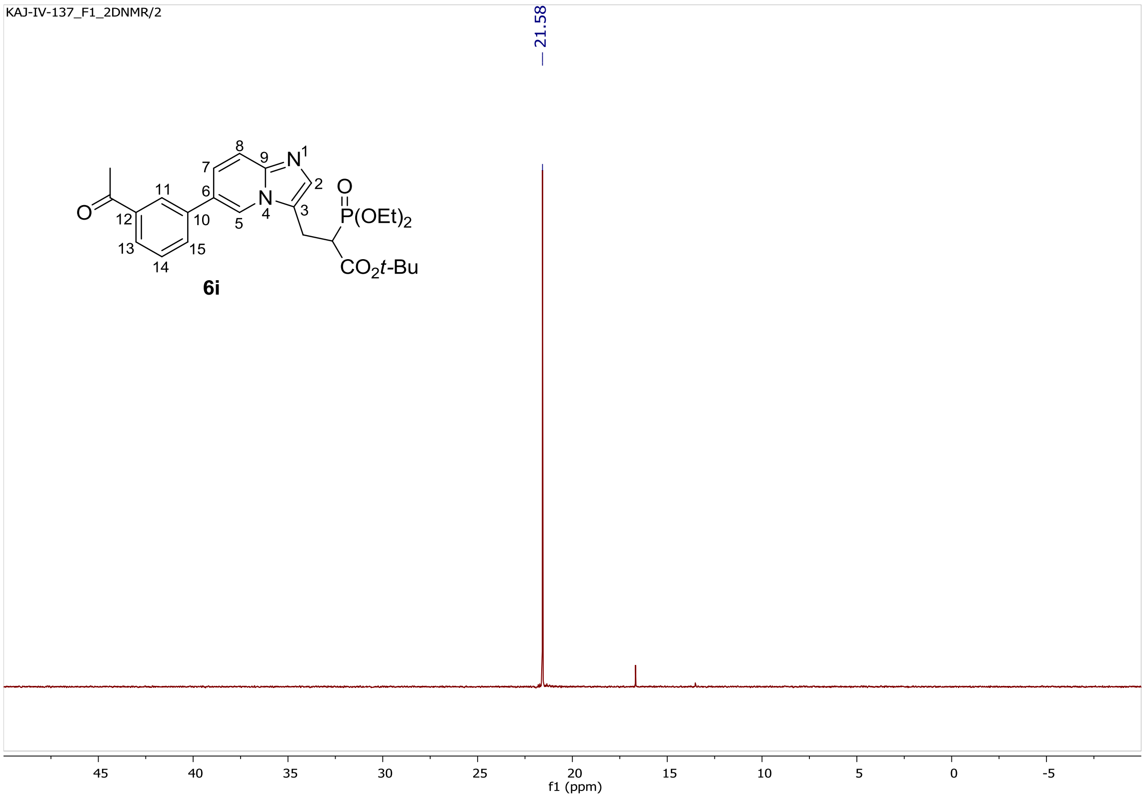

Figure S68. ^{31}P NMR of compound **6i** (283 MHz, $CDCl_3$).

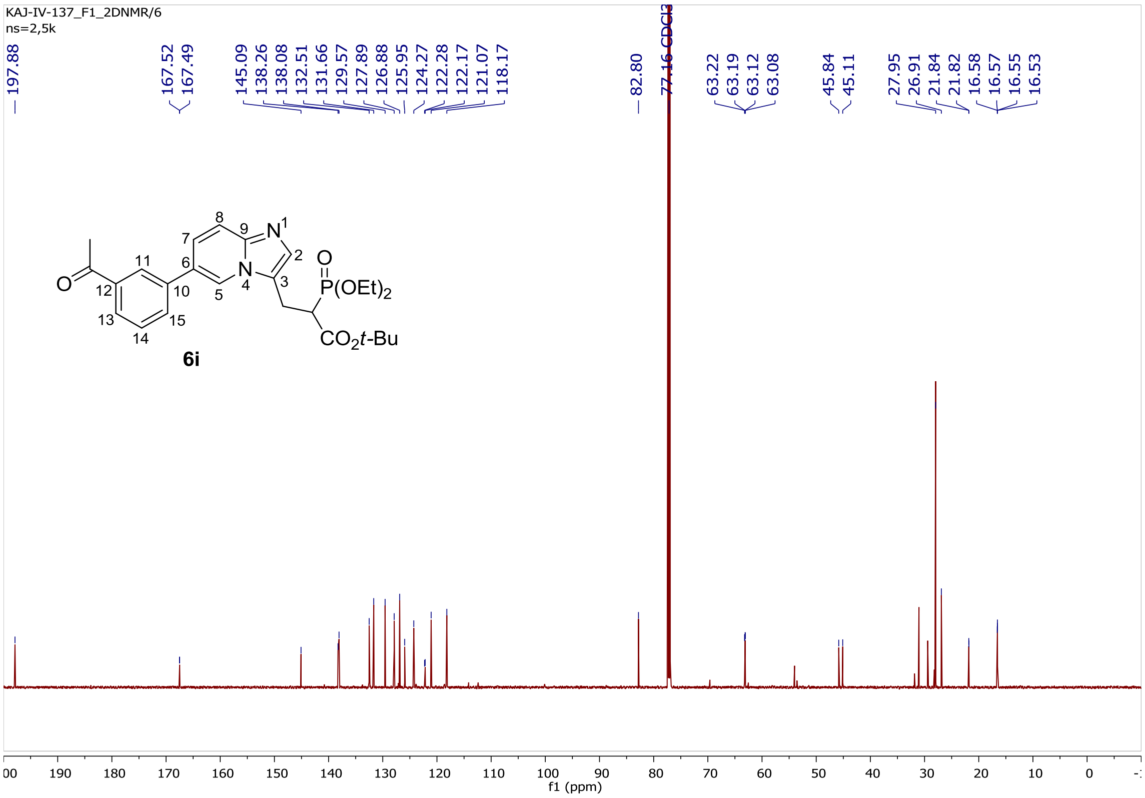

Figure S69. ^{13}C NMR of compound **6i** (176 MHz, $CDCl_3$).

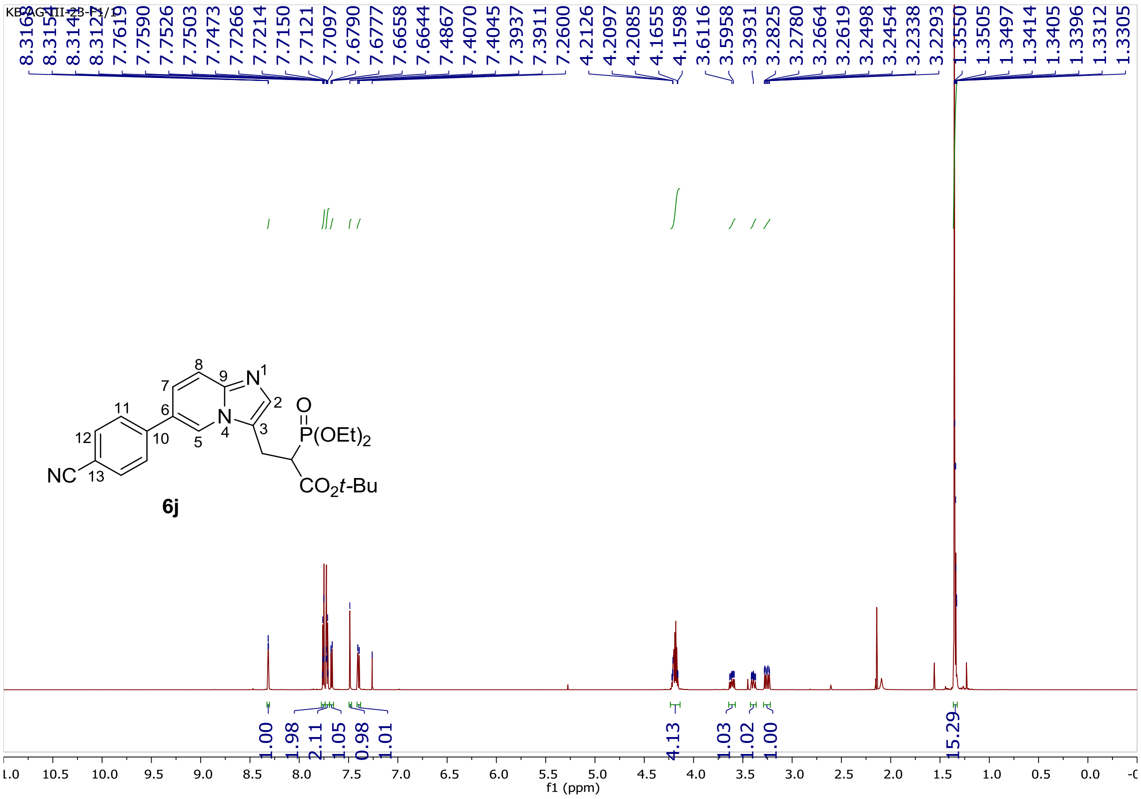

Figure S70. ^{1}H NMR of compound **6j** (700 MHz, $CDCl_3$).

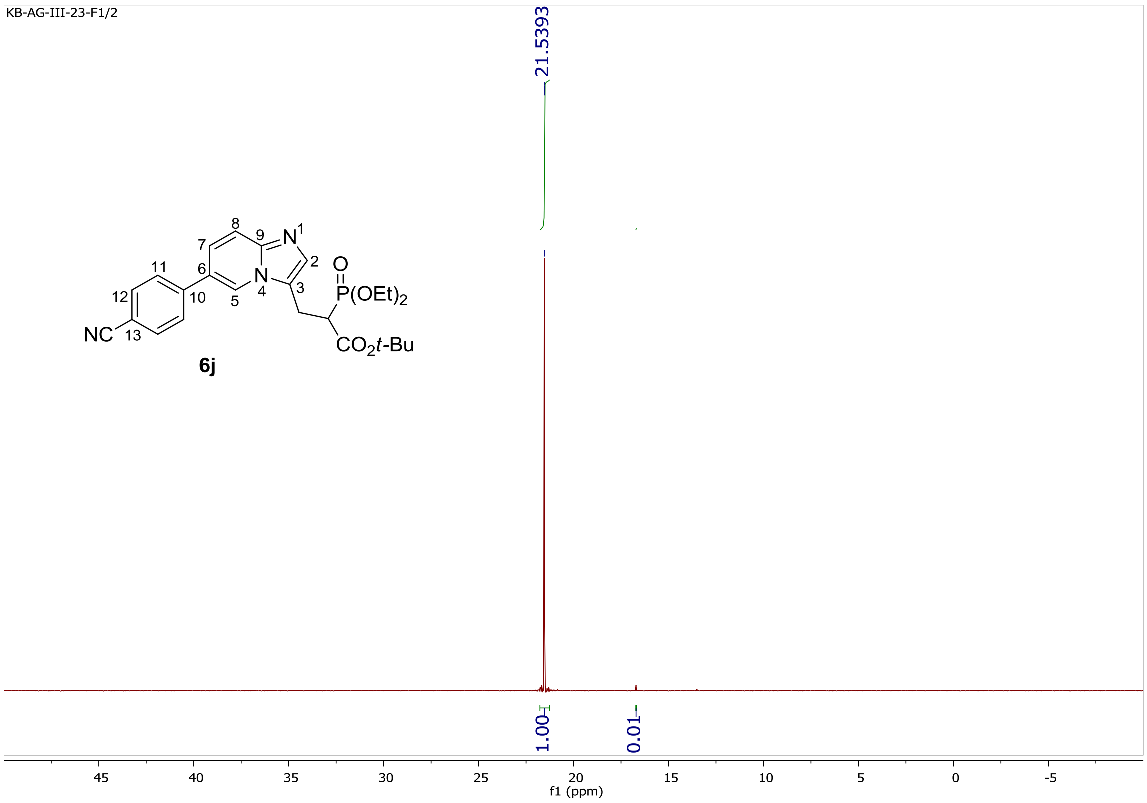

Figure S71. ^{31}P NMR of compound **6j** (283 MHz, $CDCl_3$).

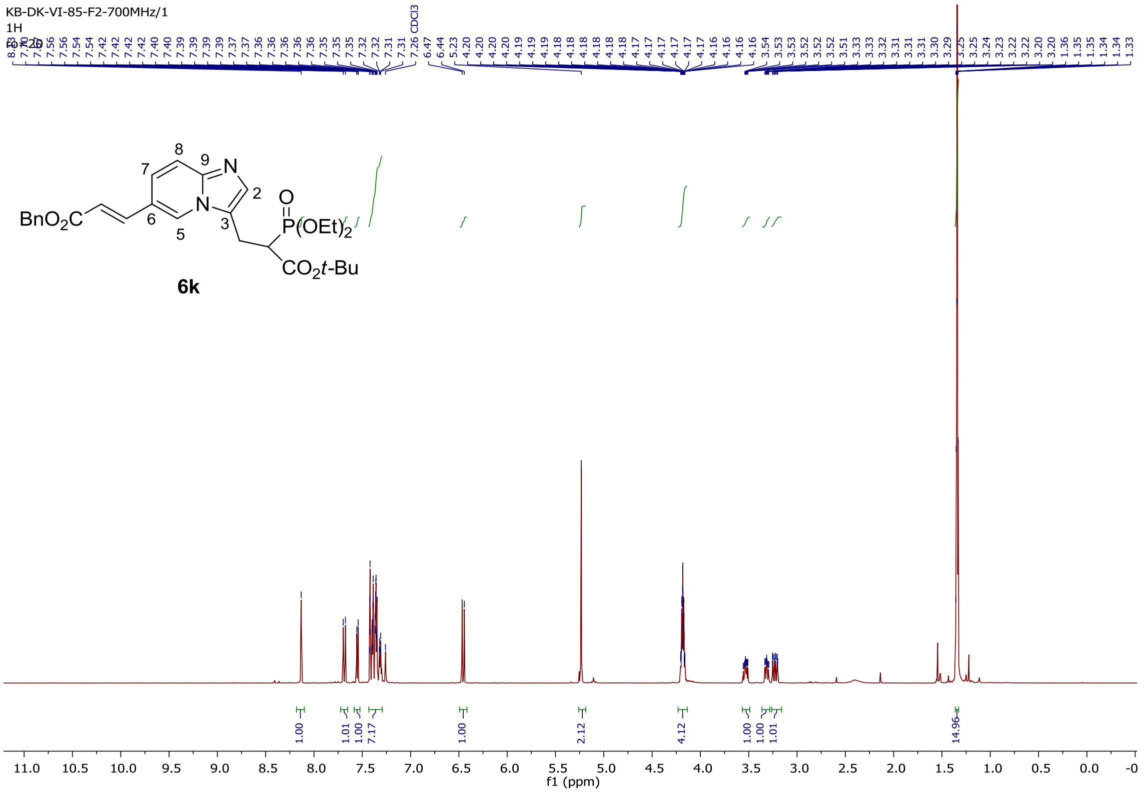

Figure S72. ^{1}H NMR of compound **6k** (700 MHz, $CDCl_3$).

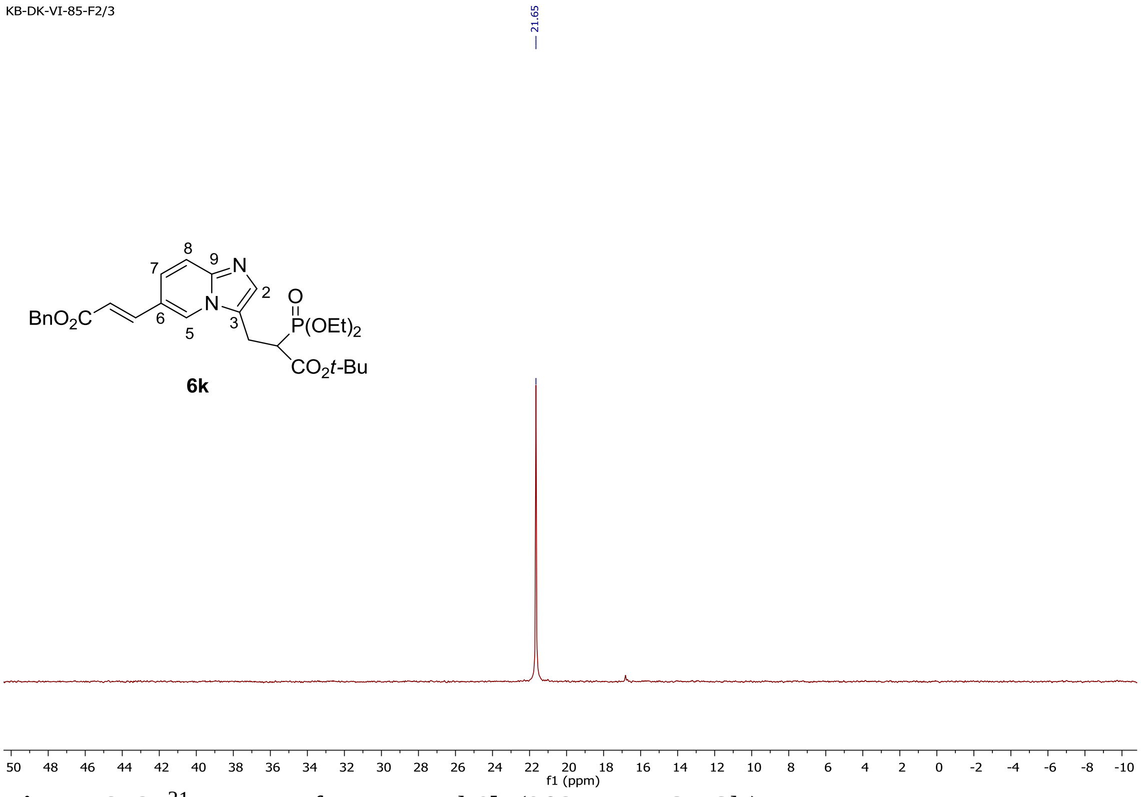

Figure S73. ^{31}P NMR of compound **6k** (283 MHz, $CDCl_3$).

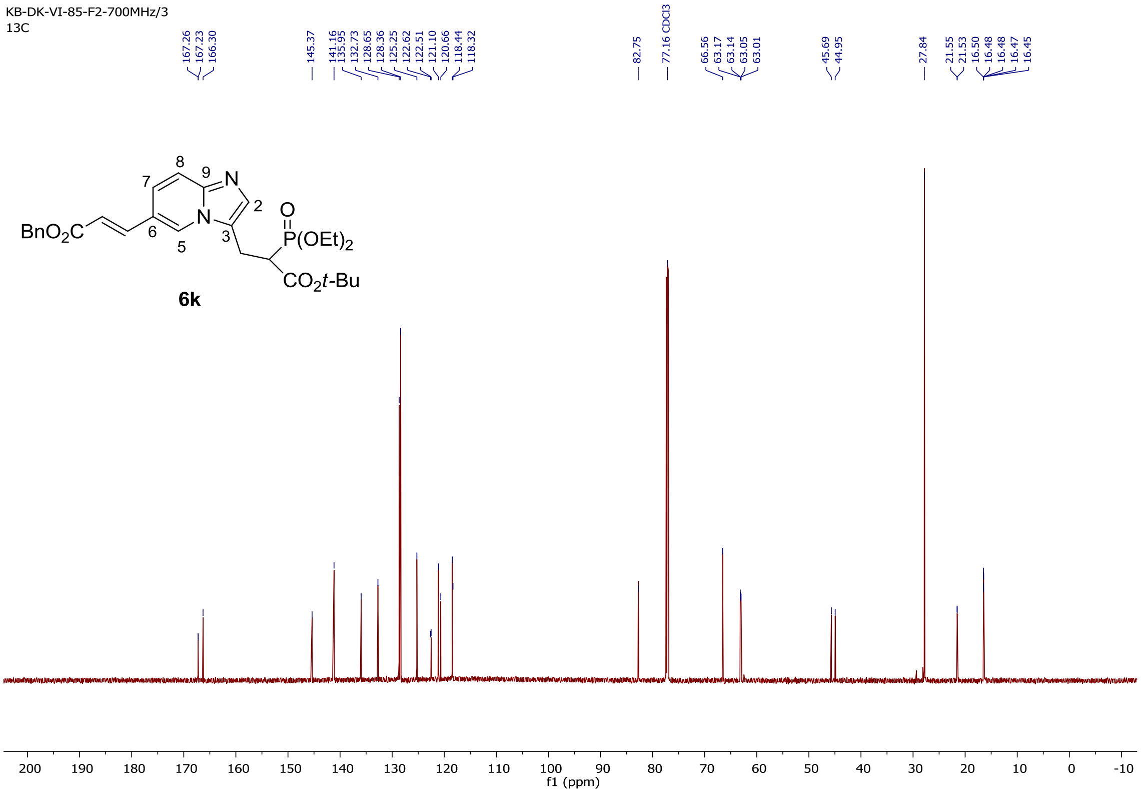

Figure S74. ^{13}C NMR of compound **6k** (176 MHz, $CDCl_3$).

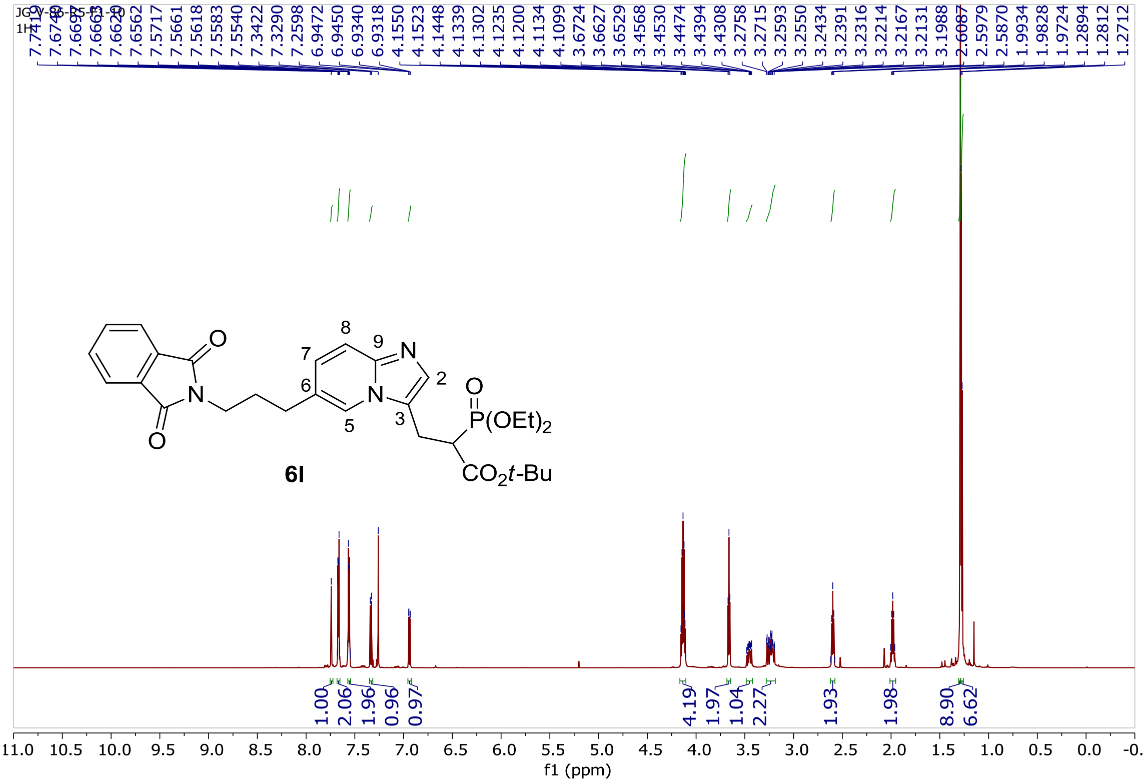

Figure S75. ^{1}H NMR of compound **6l** (700 MHz, $CDCl_3$).

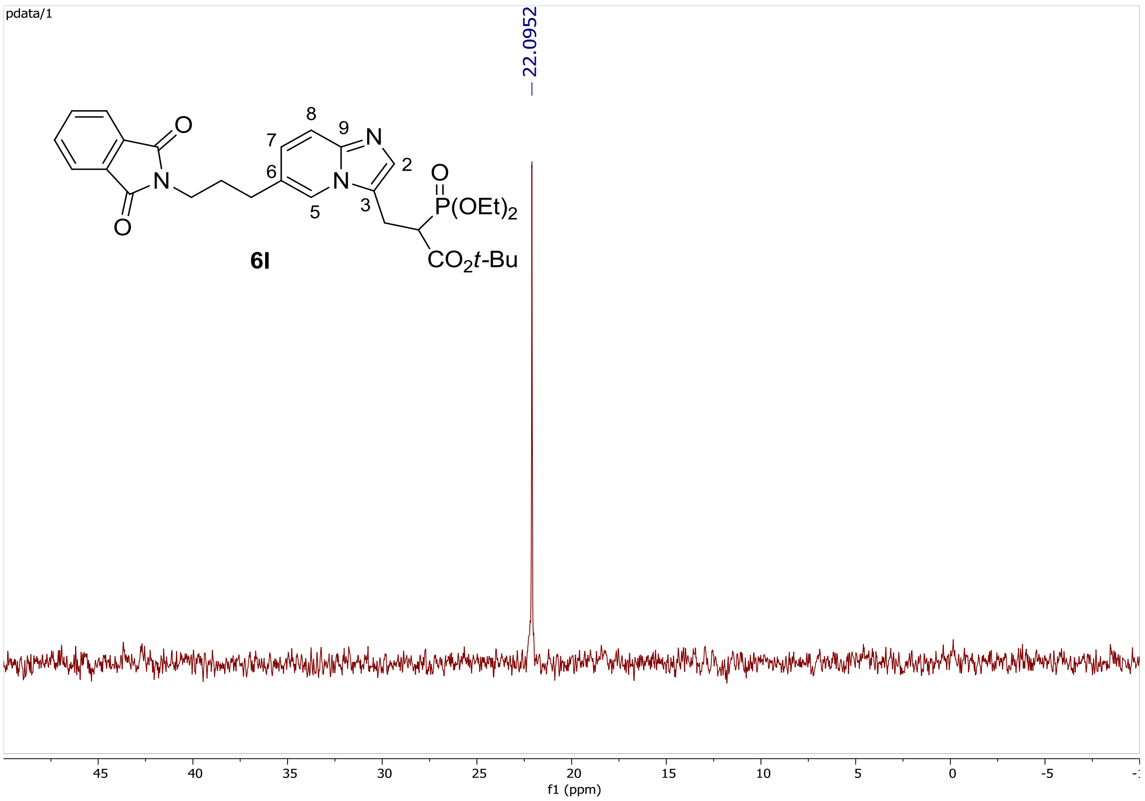

Figure S76. ^{31}P NMR of compound **6l** (101 MHz, $CDCl_3$).

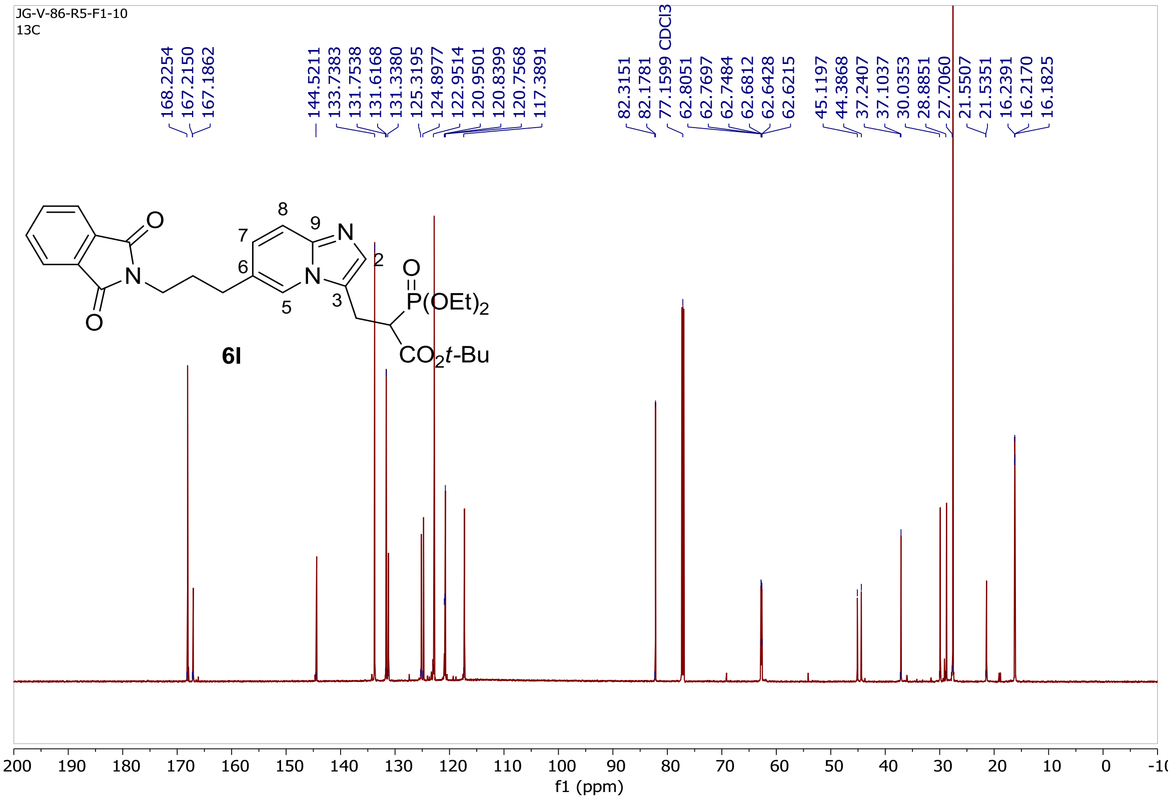

Figure S77. ^{13}C NMR of compound **6l** (176 MHz, $CDCl_3$).

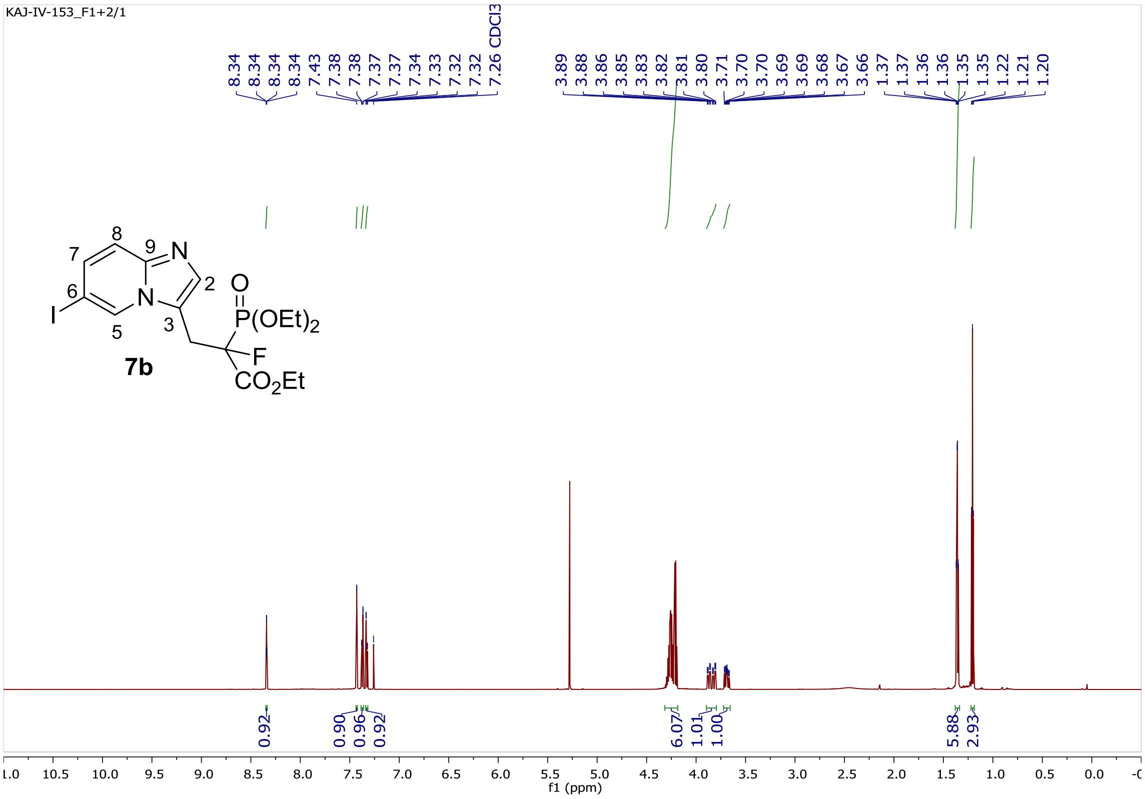

Figure S78. ^{1}H NMR of compound **7b** (700 MHz, $CDCl_3$).

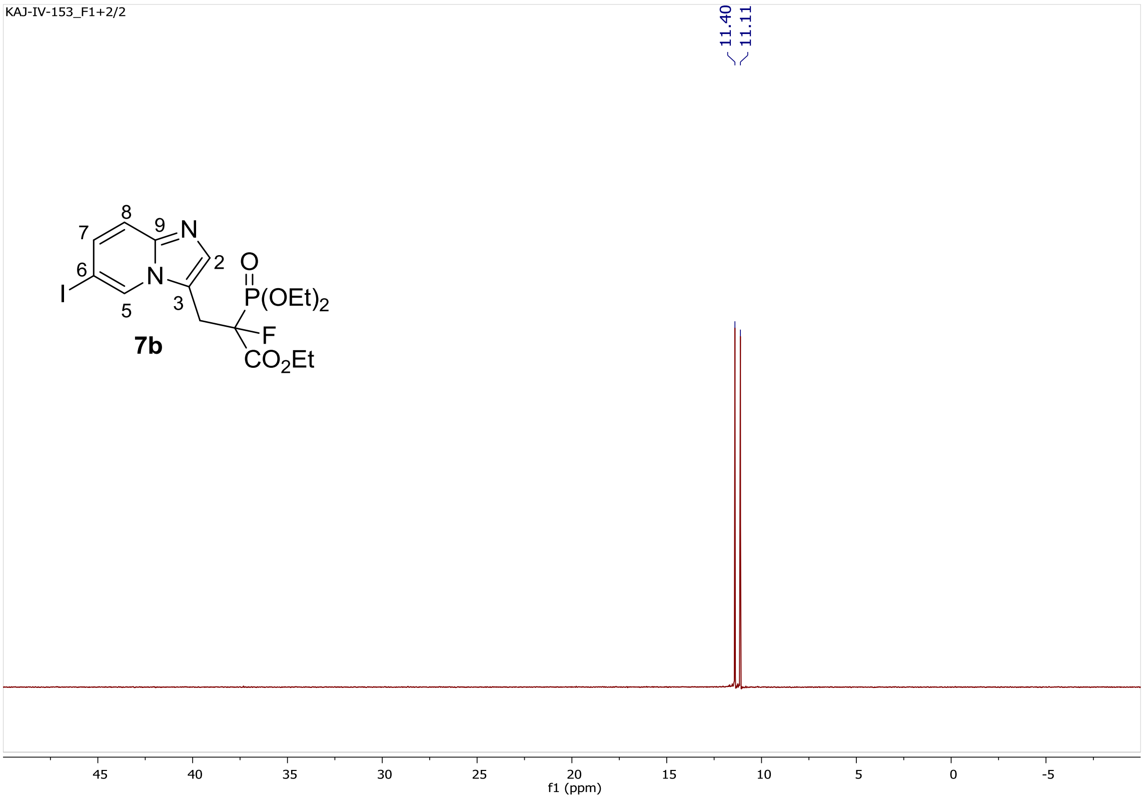

Figure S79. ^{31}P NMR of compound **7b** (283 MHz, $CDCl_3$).

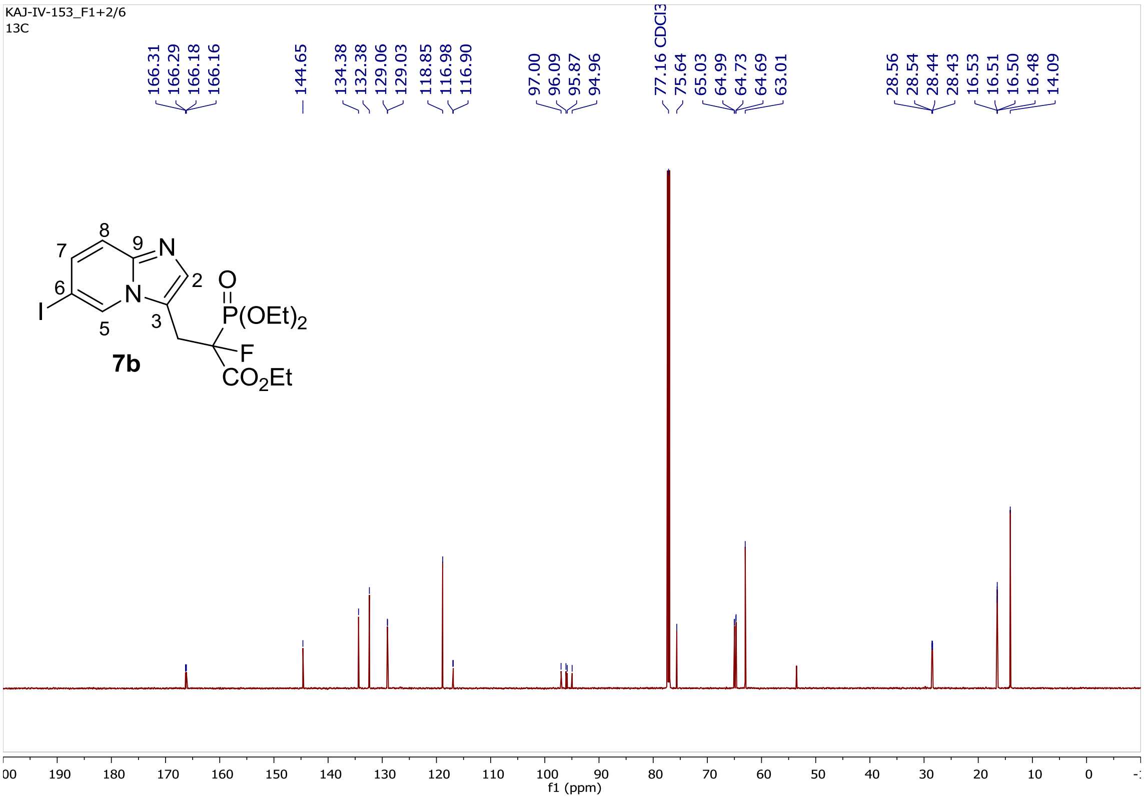

Figure S80. ^{13}C NMR of compound **7b** (176 MHz, $CDCl_3$).

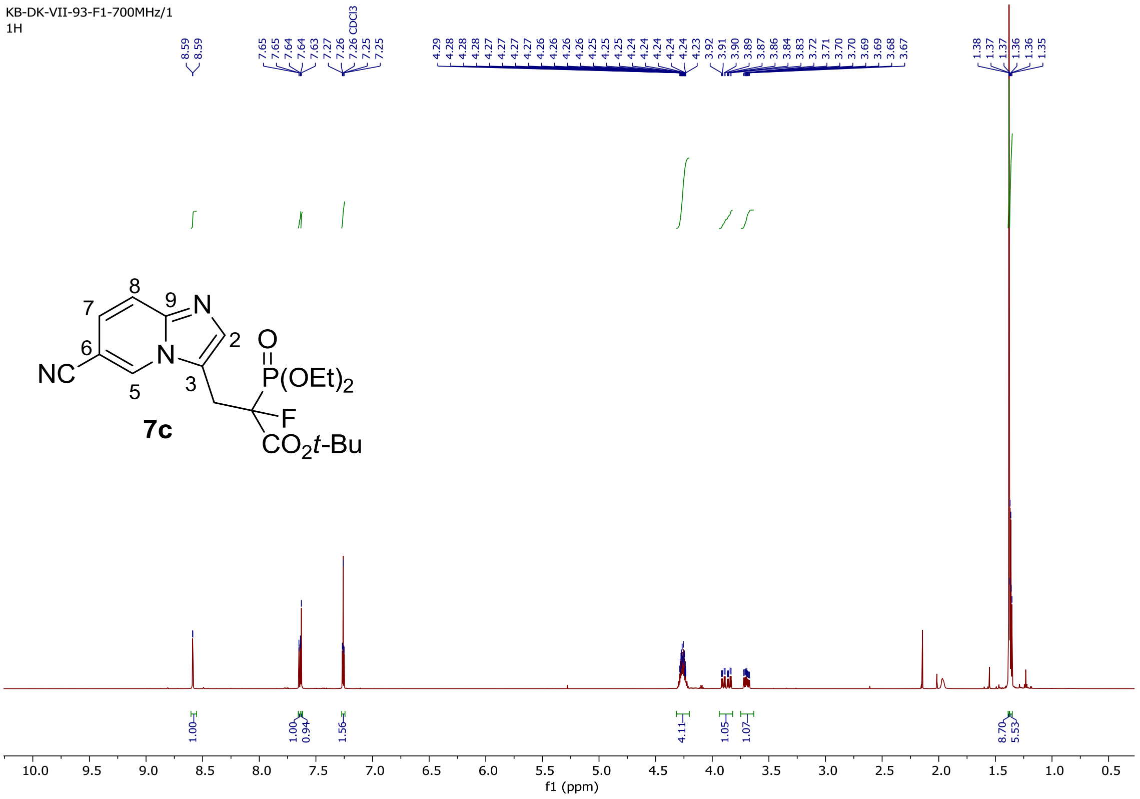

Figure S81. ^{1}H NMR of compound **7c** (700 MHz, $CDCl_3$).

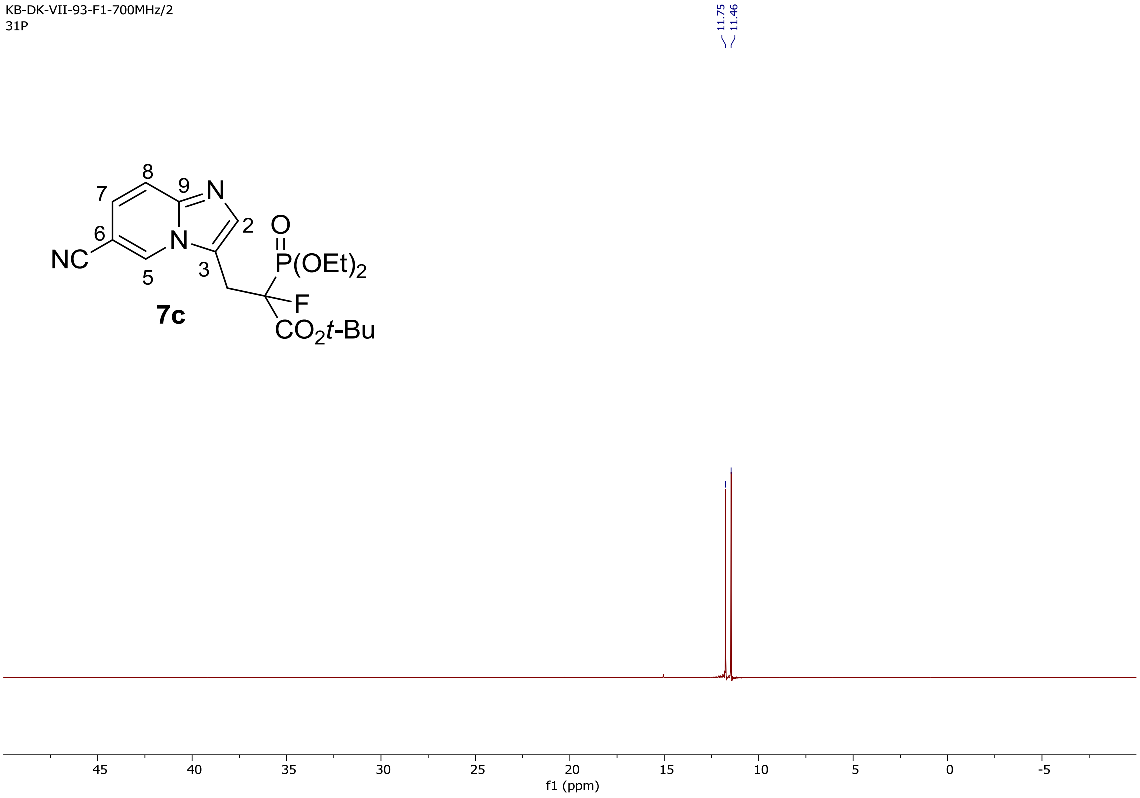

Figure S82. ^{31}P NMR of compound **7c** (283 MHz, $CDCl_3$).

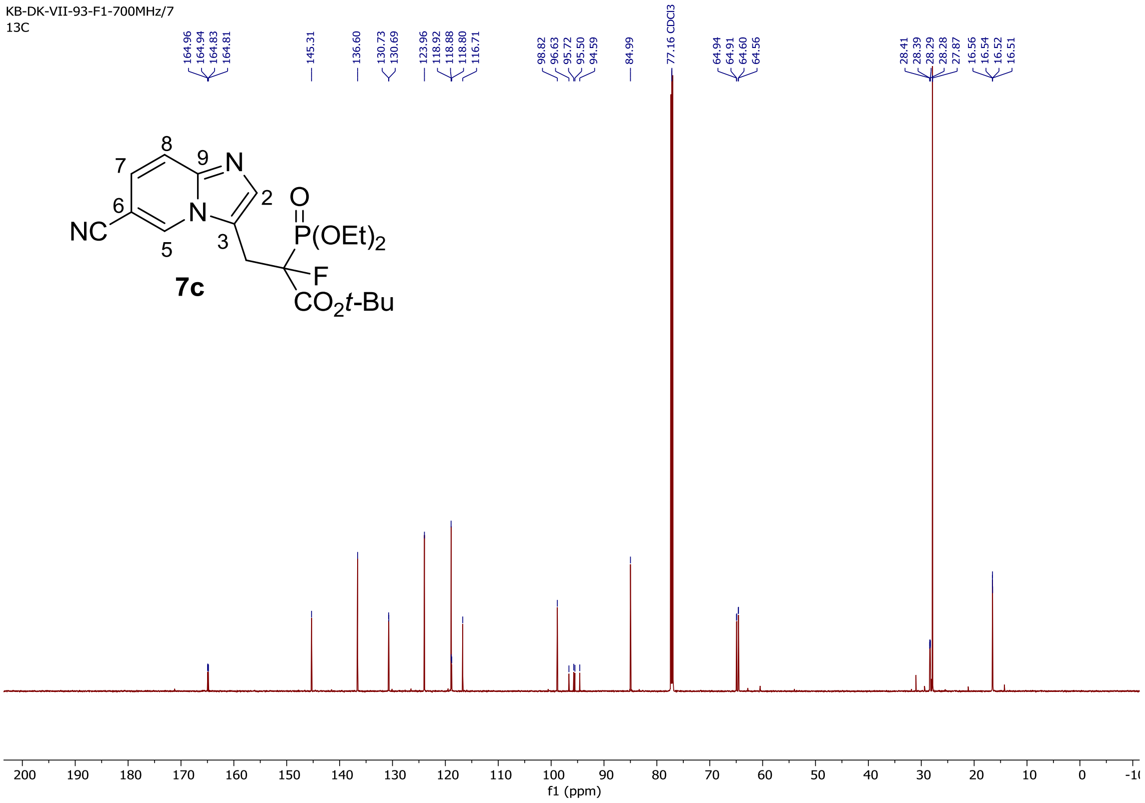

Figure S83. ^{13}C NMR of compound **7c** (176 MHz, $CDCl_3$).

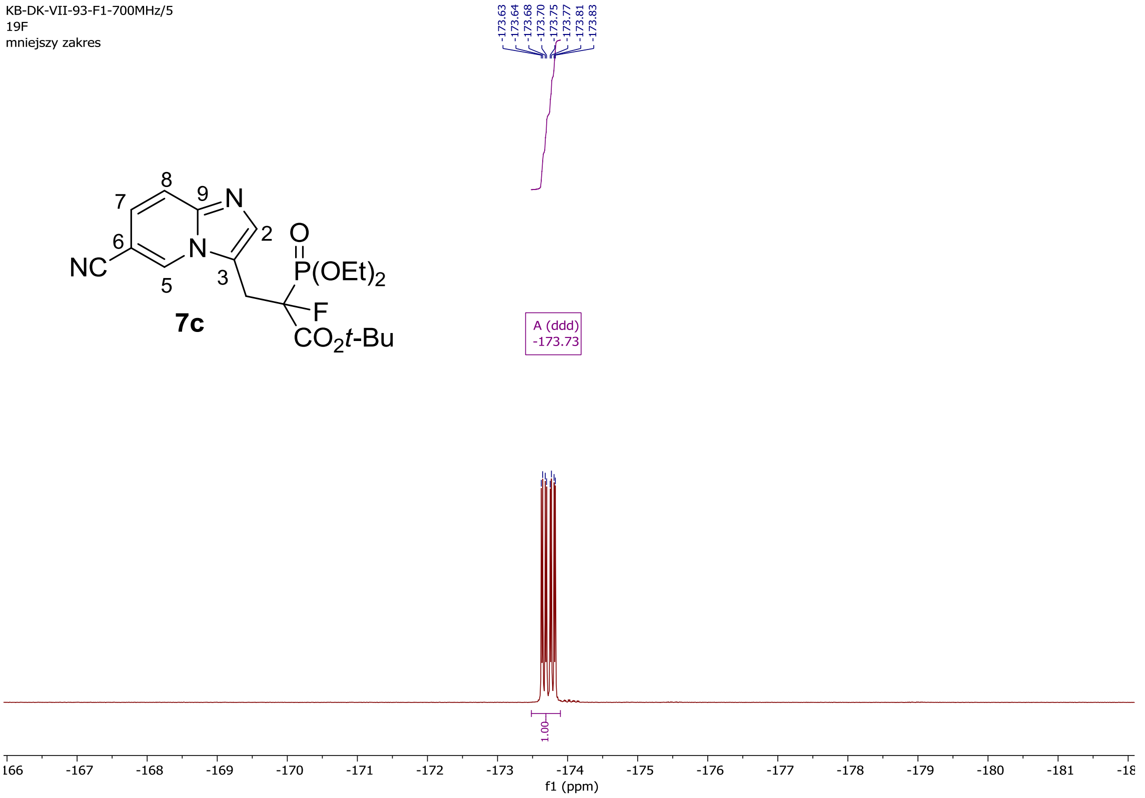

Figure S84. ^{19}F NMR of compound **7c** (659 MHz, $CDCl_3$).

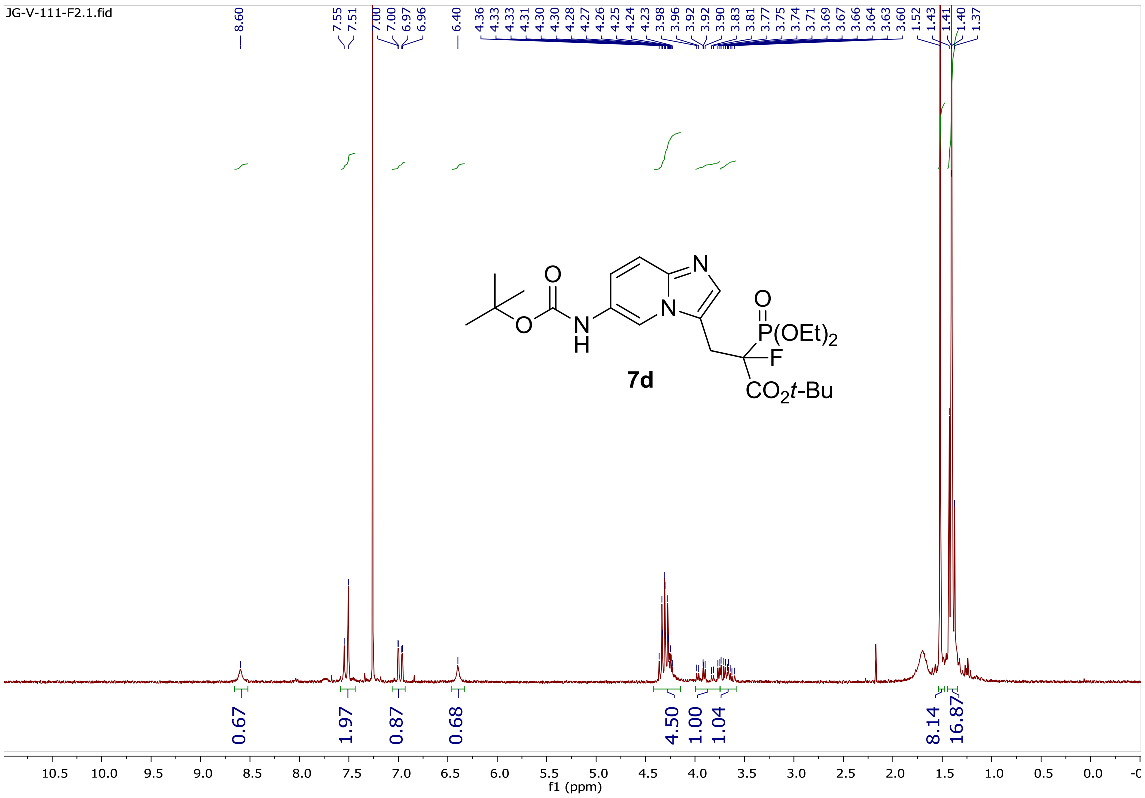

Figure S85. ^{1}H NMR of compound **7d** (250 MHz, $CDCl_3$).

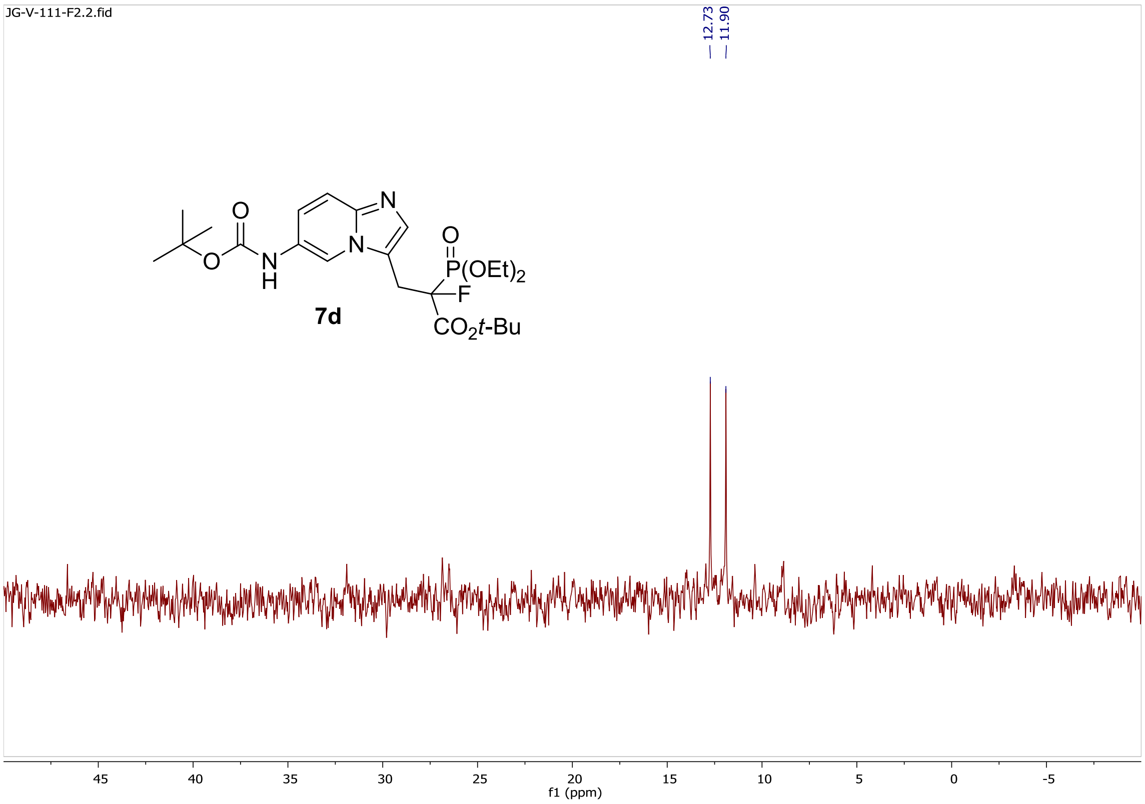

Figure S86. ^{31}P NMR of compound **7d** (101 MHz, $CDCl_3$).

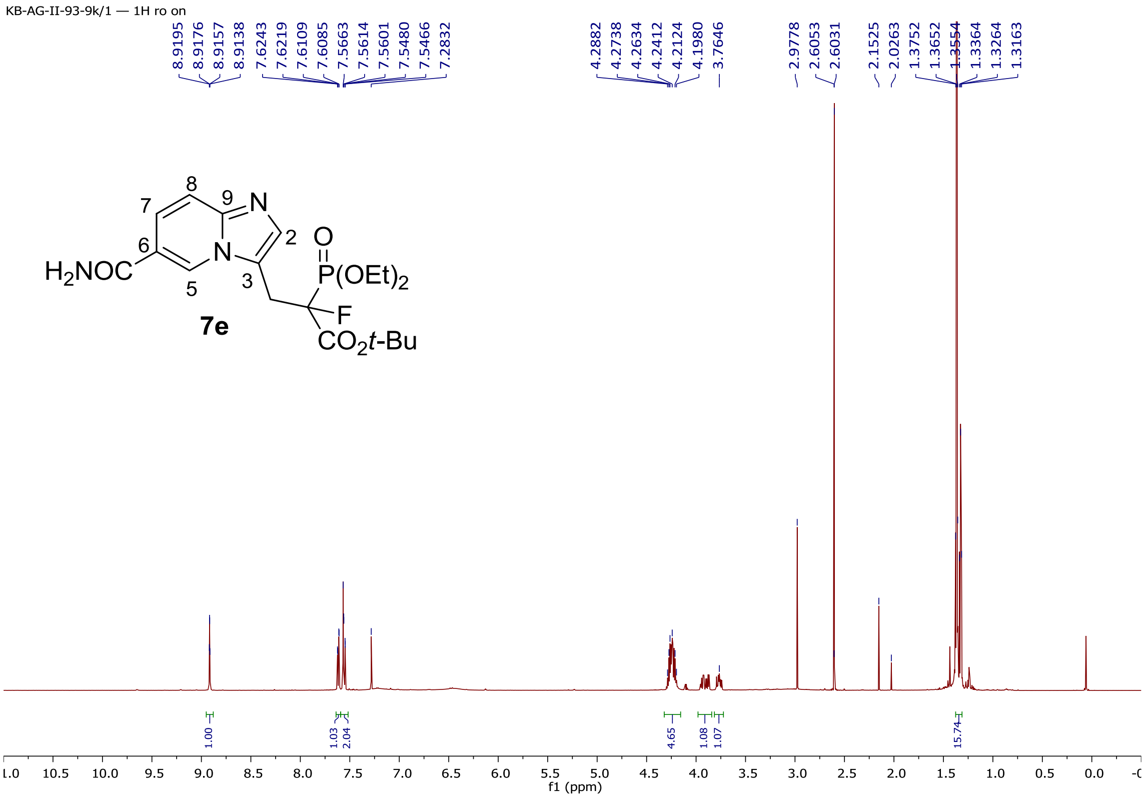

Figure S87. ^{1}H NMR of compound **7e** (700 MHz, $CDCl_3$).

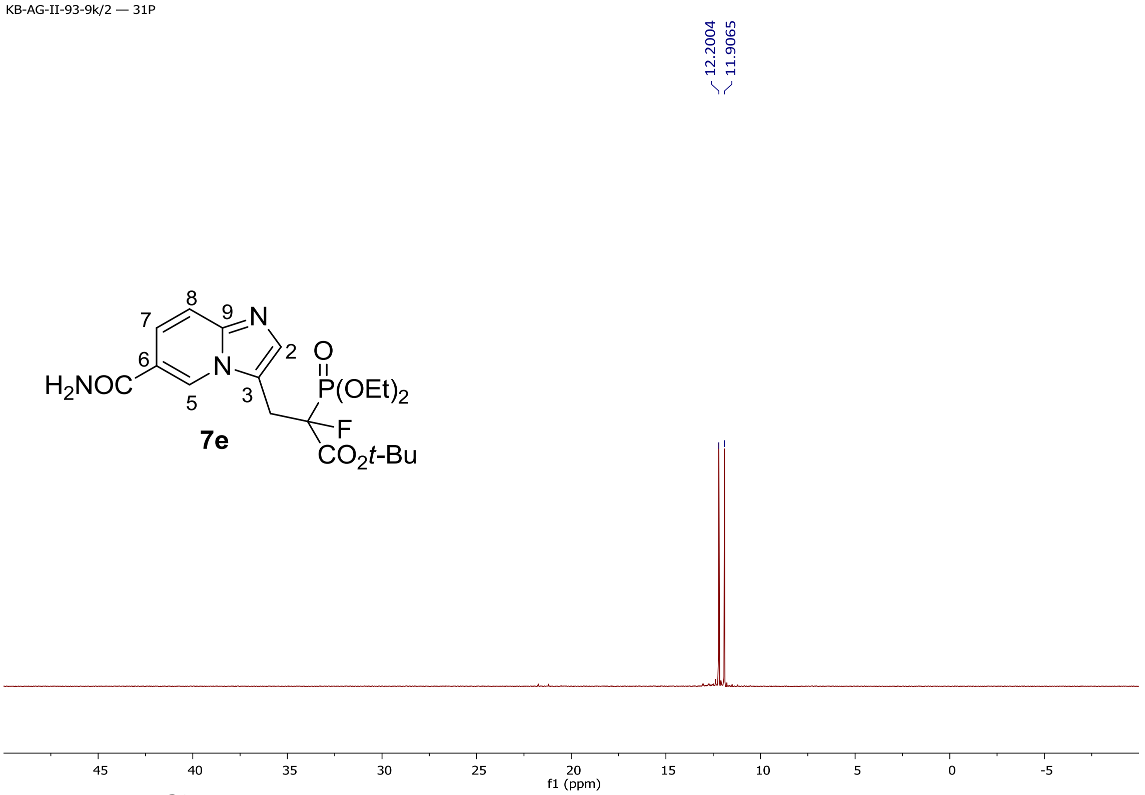

Figure S88. ^{31}P NMR of compound **7e** (284 MHz, $CDCl_3$).

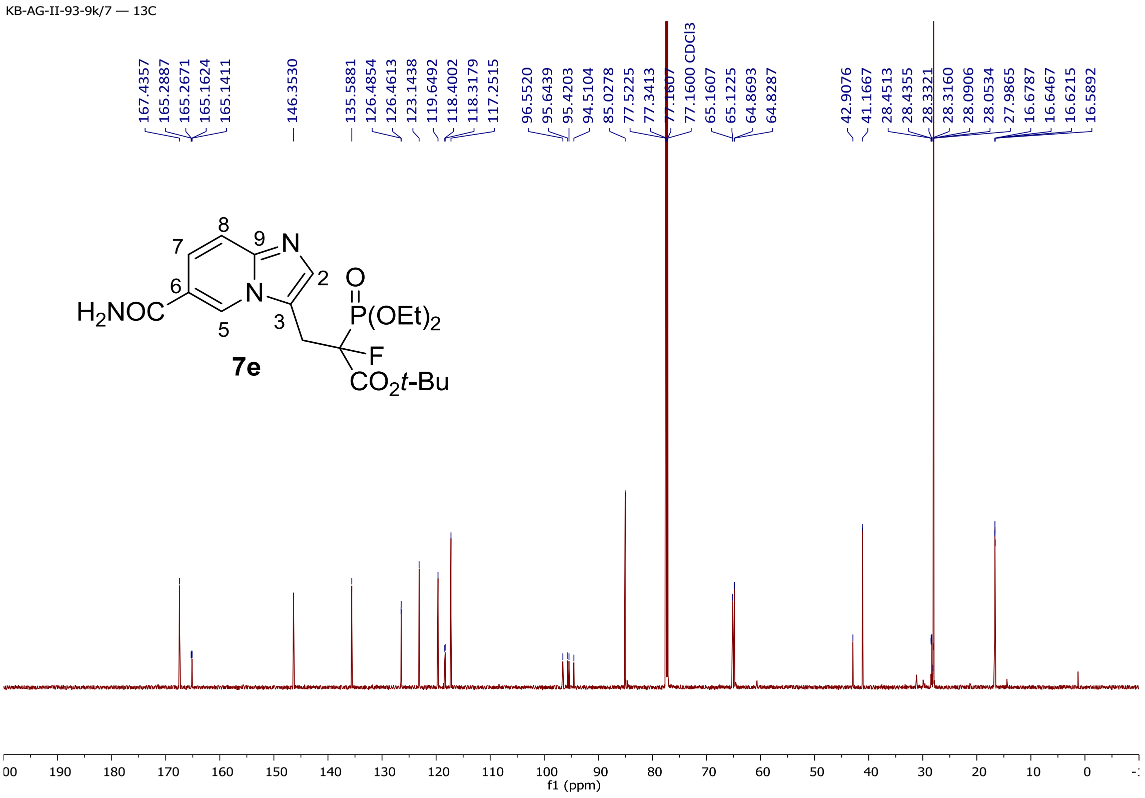

Figure S89. ^{13}C NMR of compound **7e** (176 MHz, $CDCl_3$).

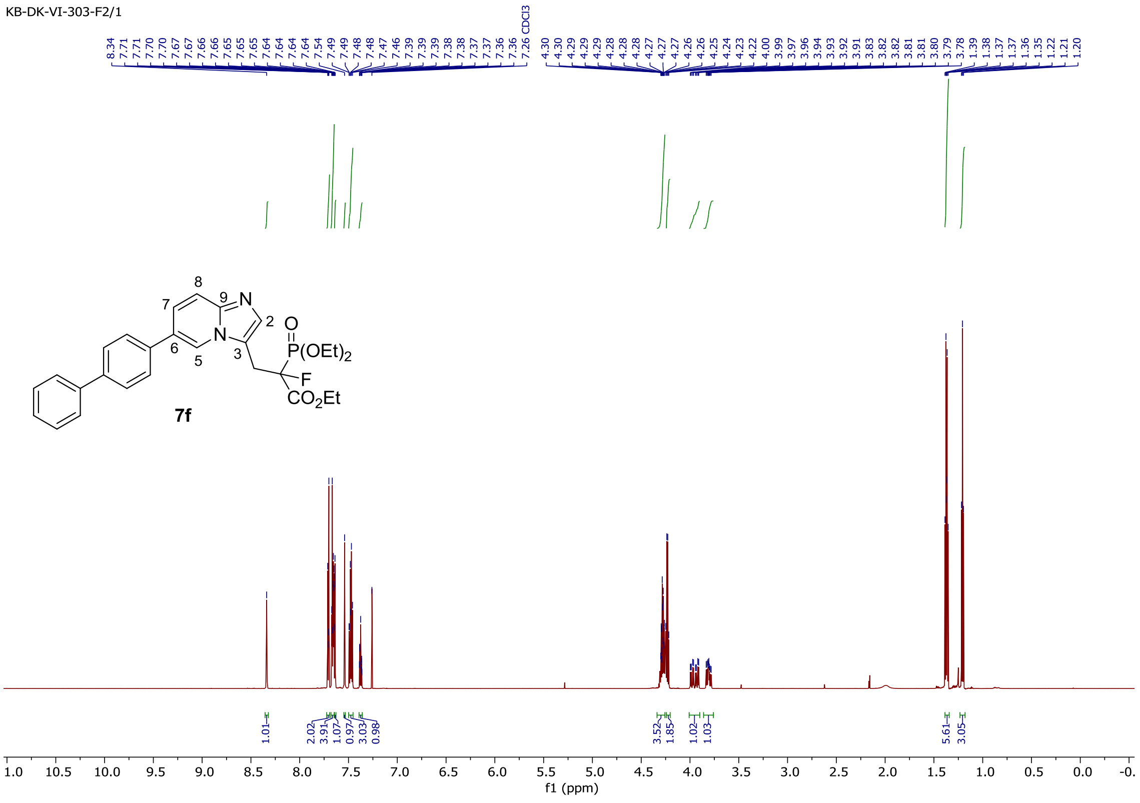

Figure S90. ^{1}H NMR of compound **7f** (700 MHz, $CDCl_3$).

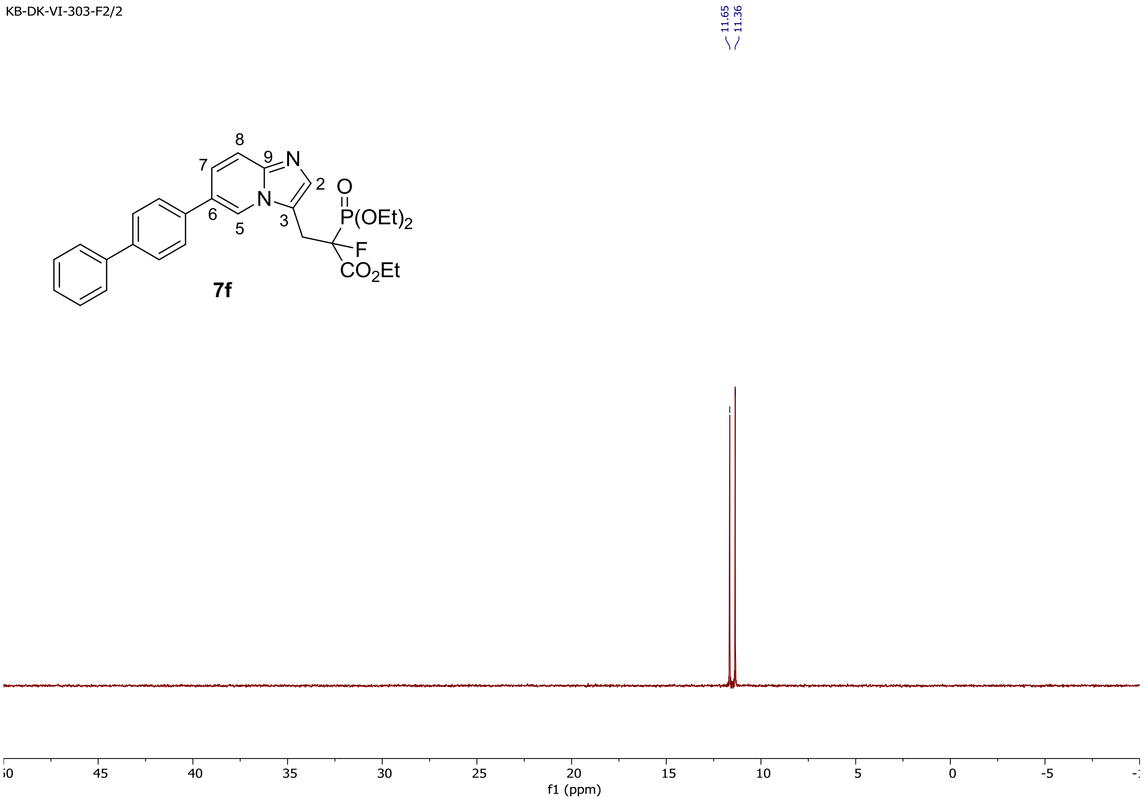

Figure S91. ^{31}P NMR of compound **7f** (284 MHz, $CDCl_3$).

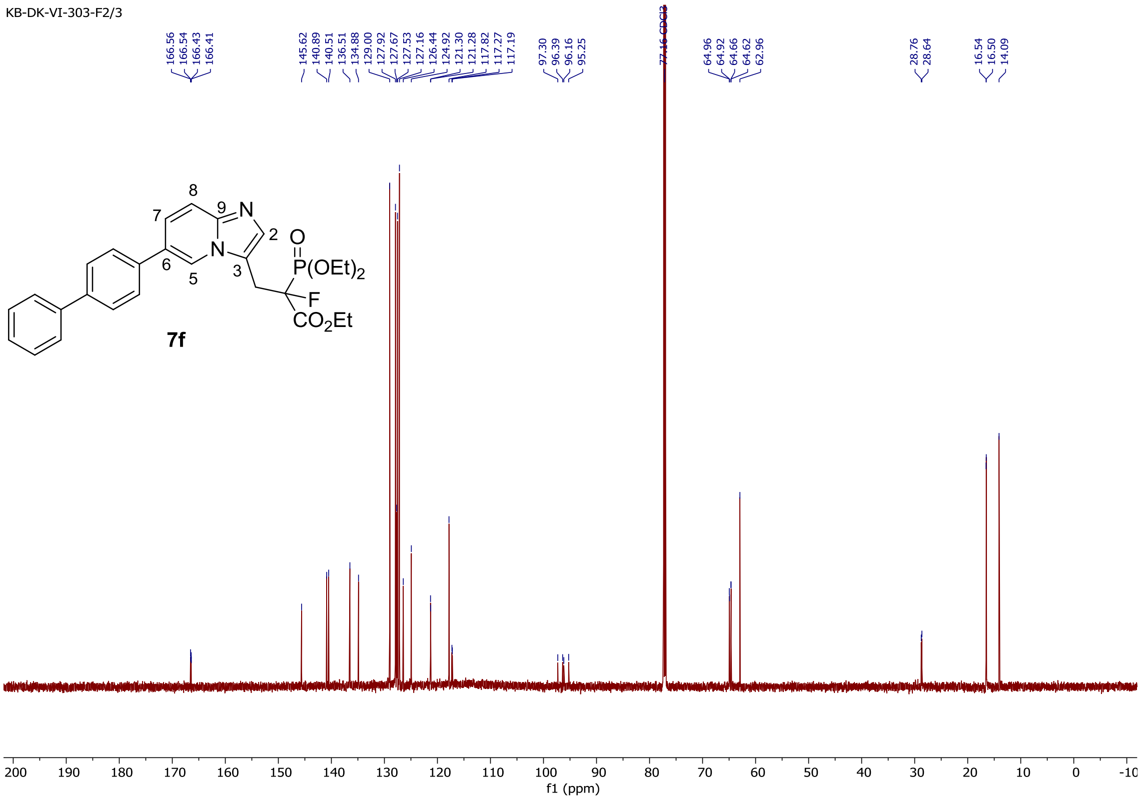

Figure S92. ^{13}C NMR of compound **7f** (176 MHz, $CDCl_3$).

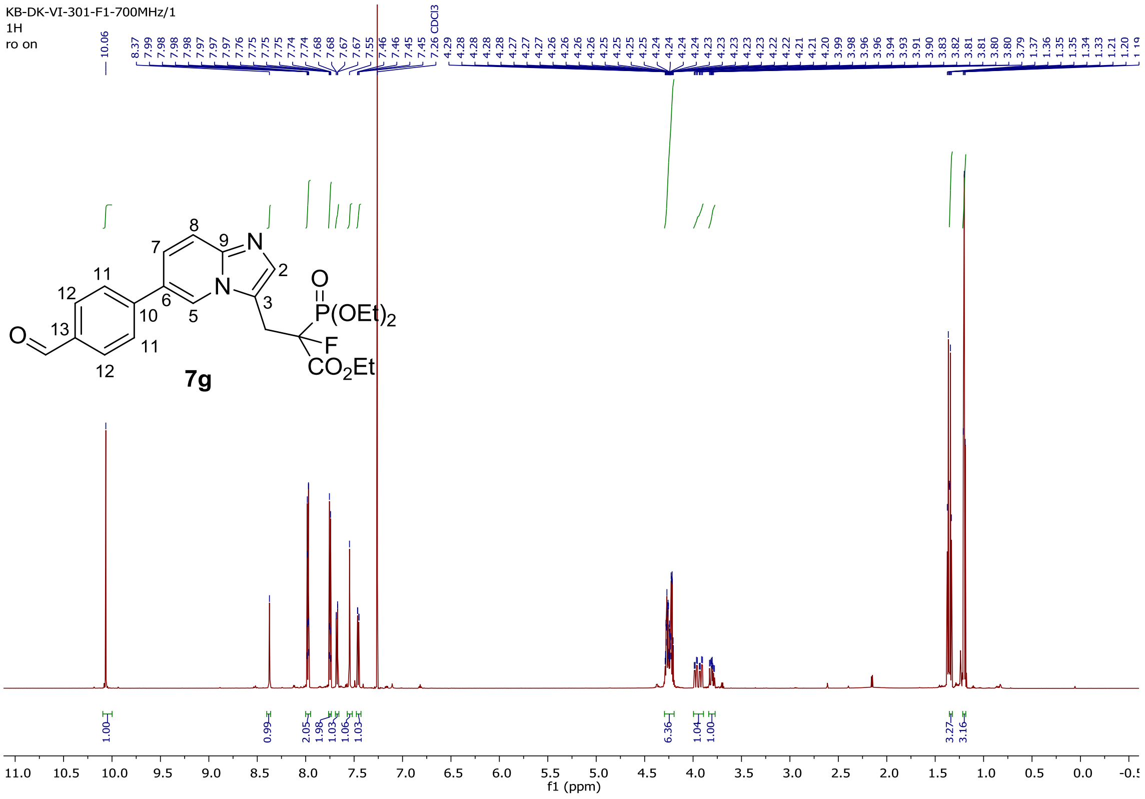

Figure S93. ^{1}H NMR of compound **7g** (700 MHz, $CDCl_3$).

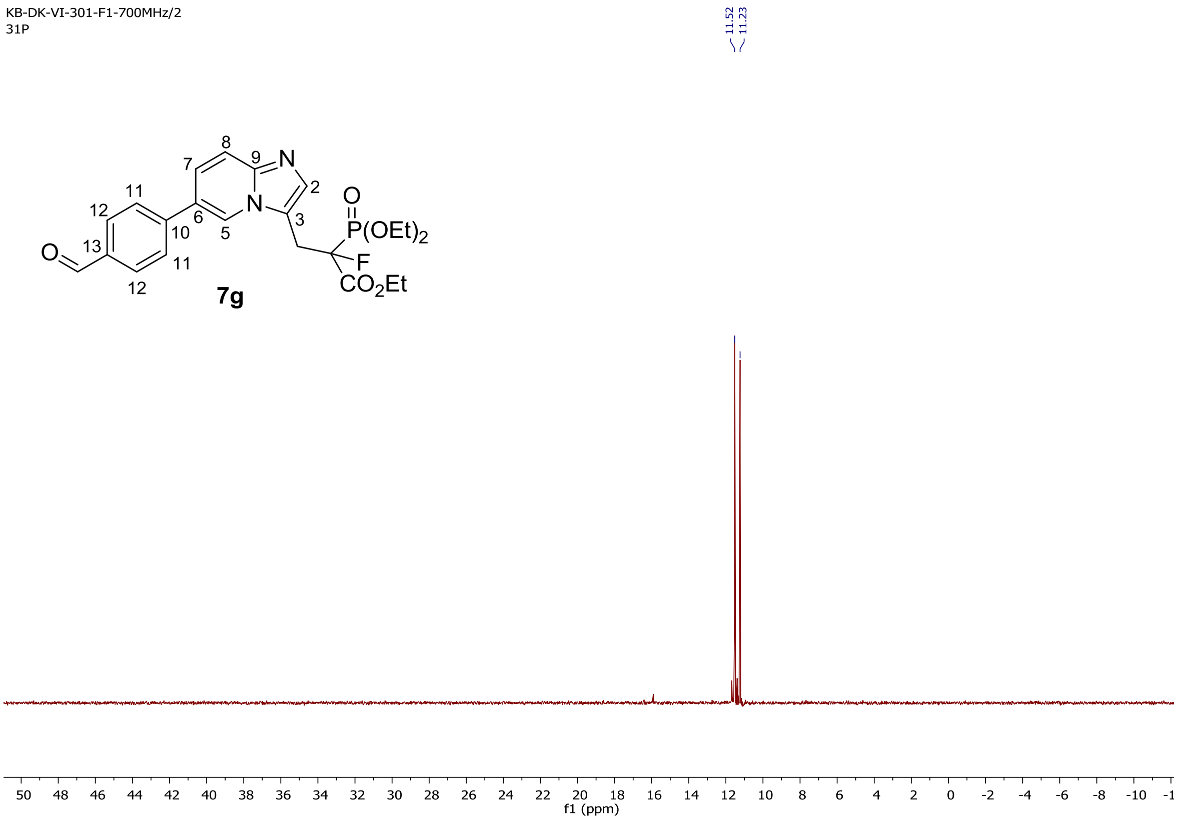

Figure S94. ^{31}P NMR of compound **7g** (284 MHz, $CDCl_3$).

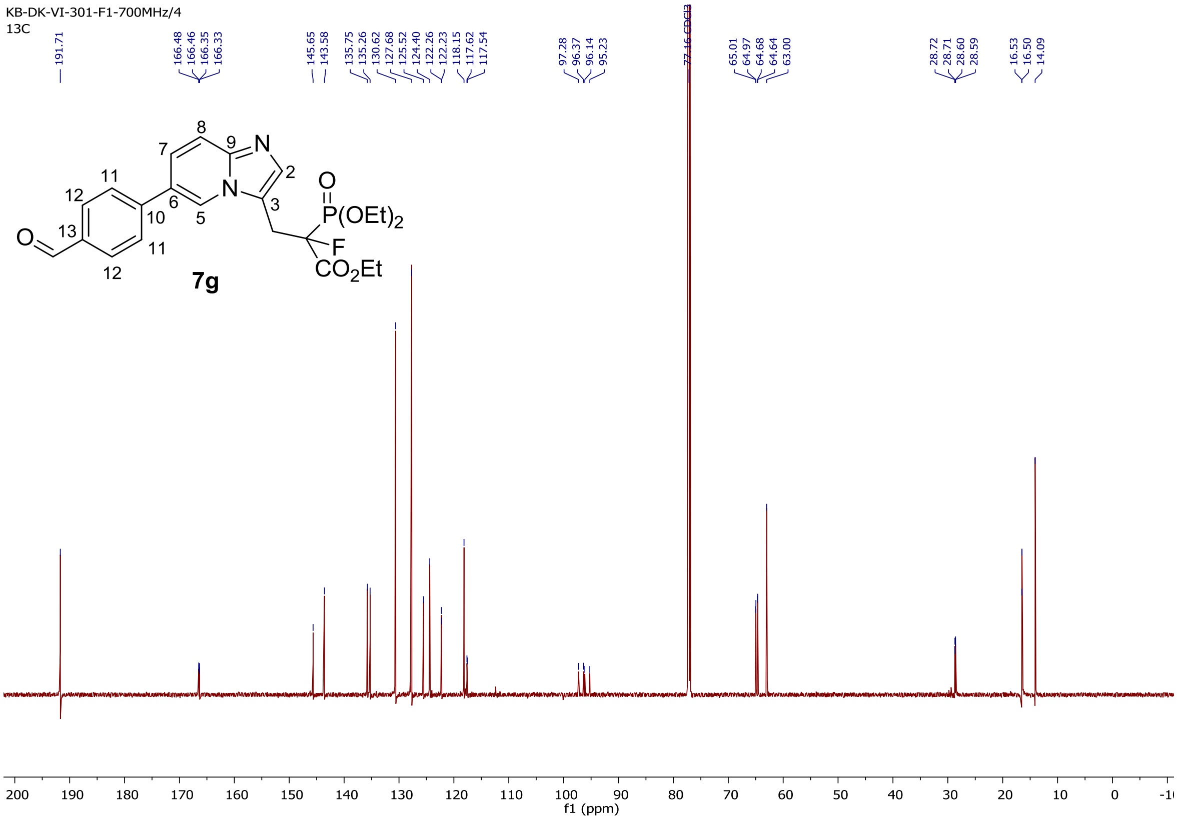

Figure S95. ^{13}C NMR of compound **7g** (176 MHz, $CDCl_3$).

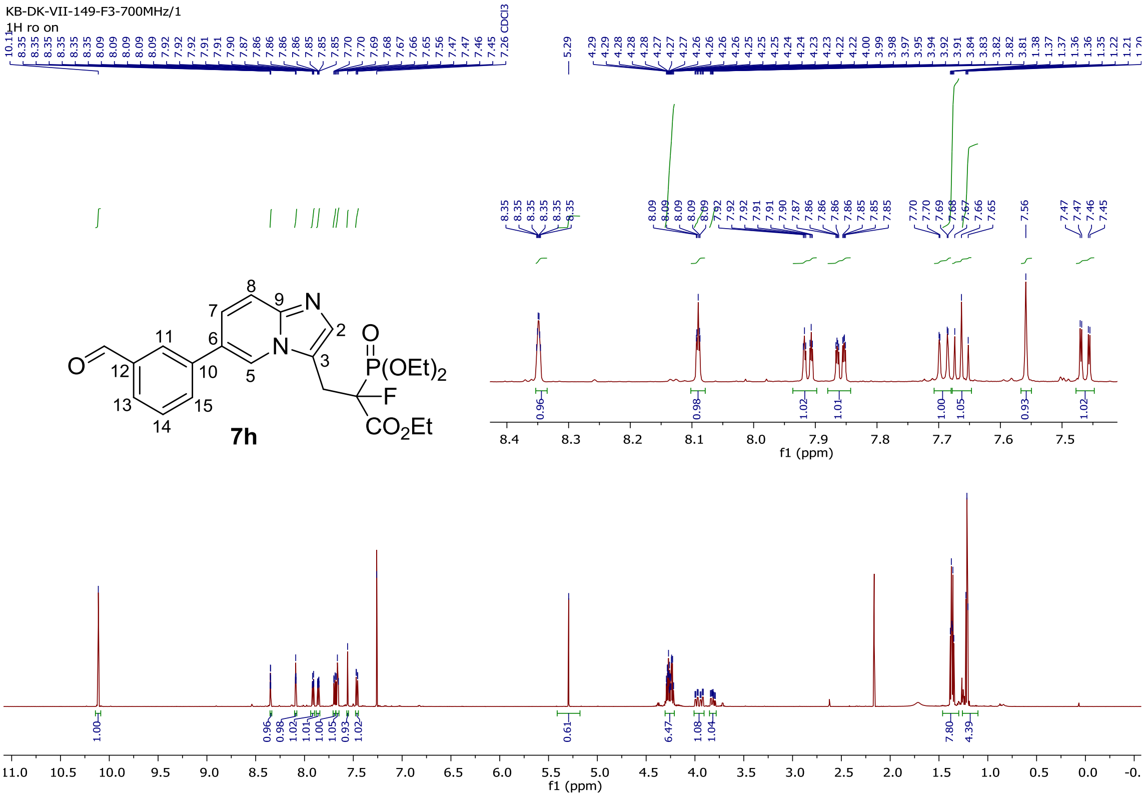

Figure S96. ^{1}H NMR of compound **7h** (700 MHz, $CDCl_3$).

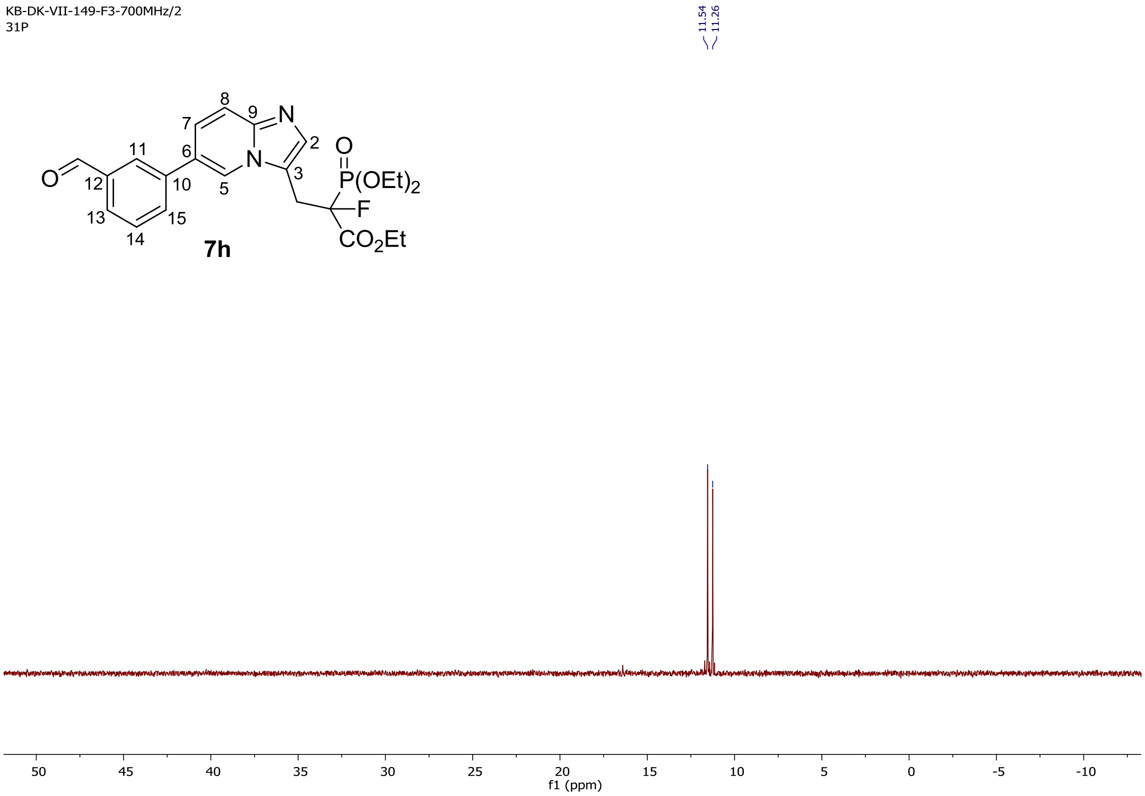

Figure S97. ^{31}P NMR of compound **7h** (283 MHz, $CDCl_3$).

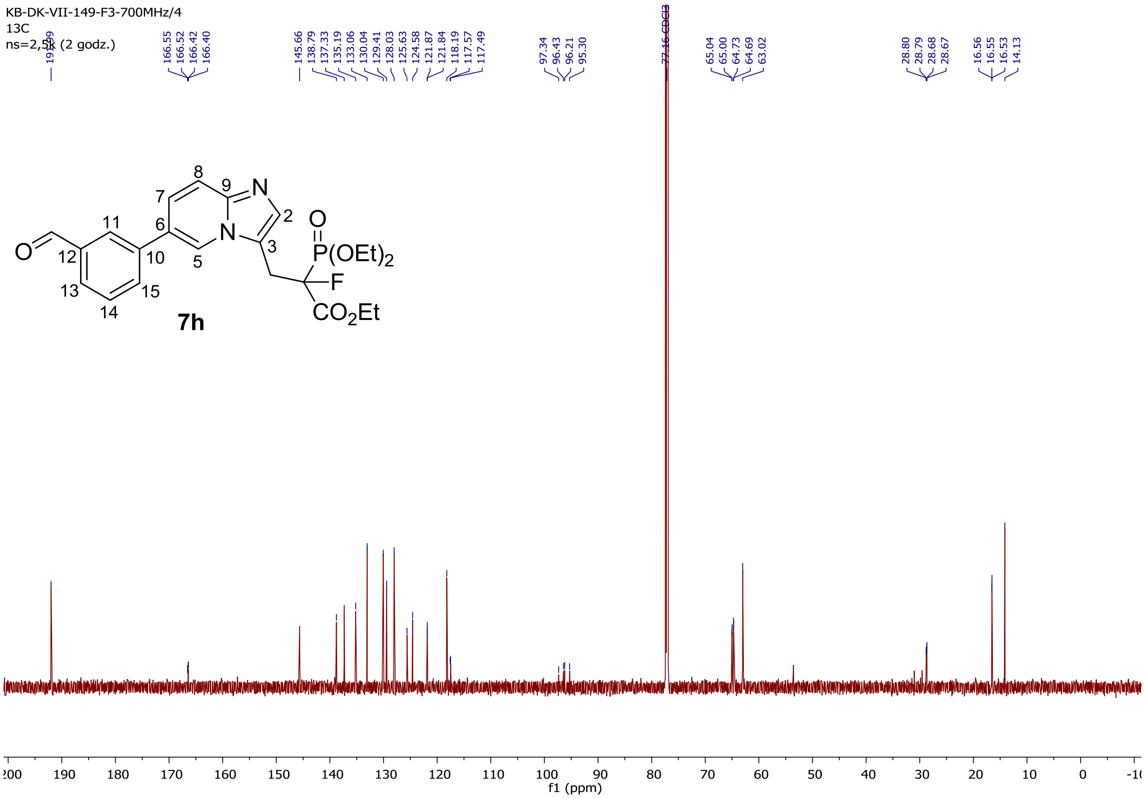

Figure S98. ^{13}C NMR of compound **7h** (176 MHz, $CDCl_3$).

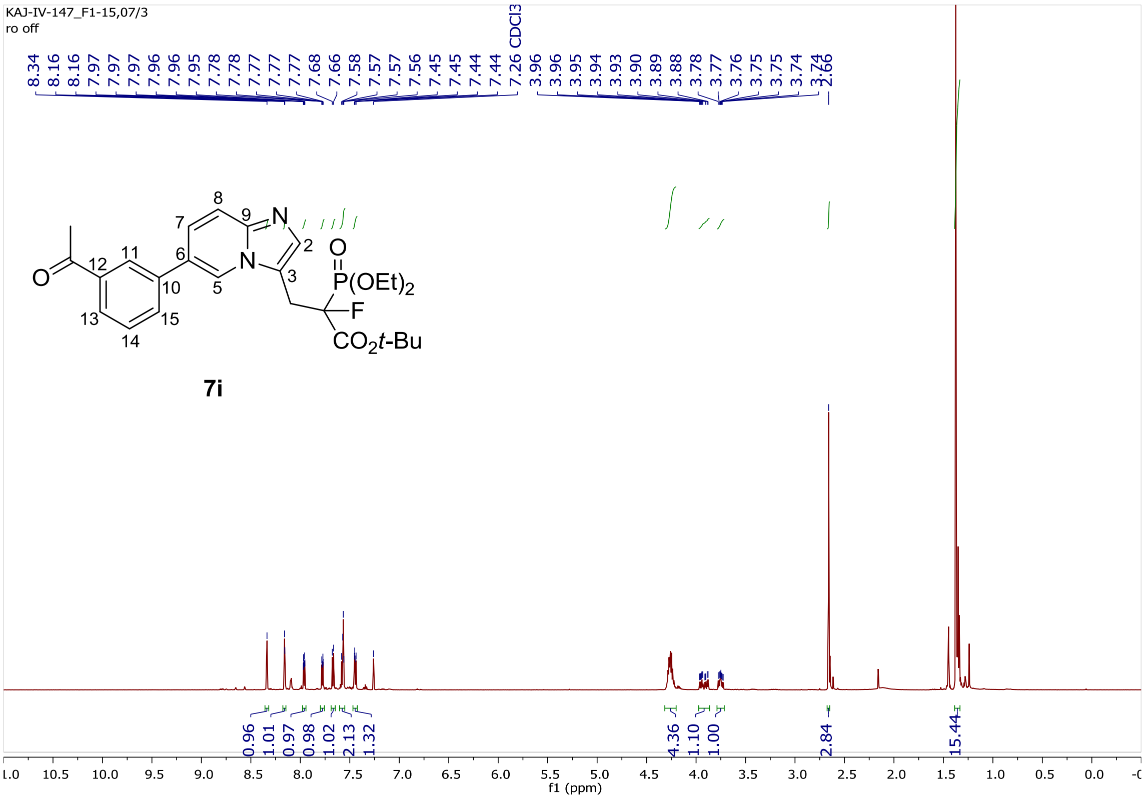

Figure S99. ^{1}H NMR of compound **7i** (700 MHz, $CDCl_3$).

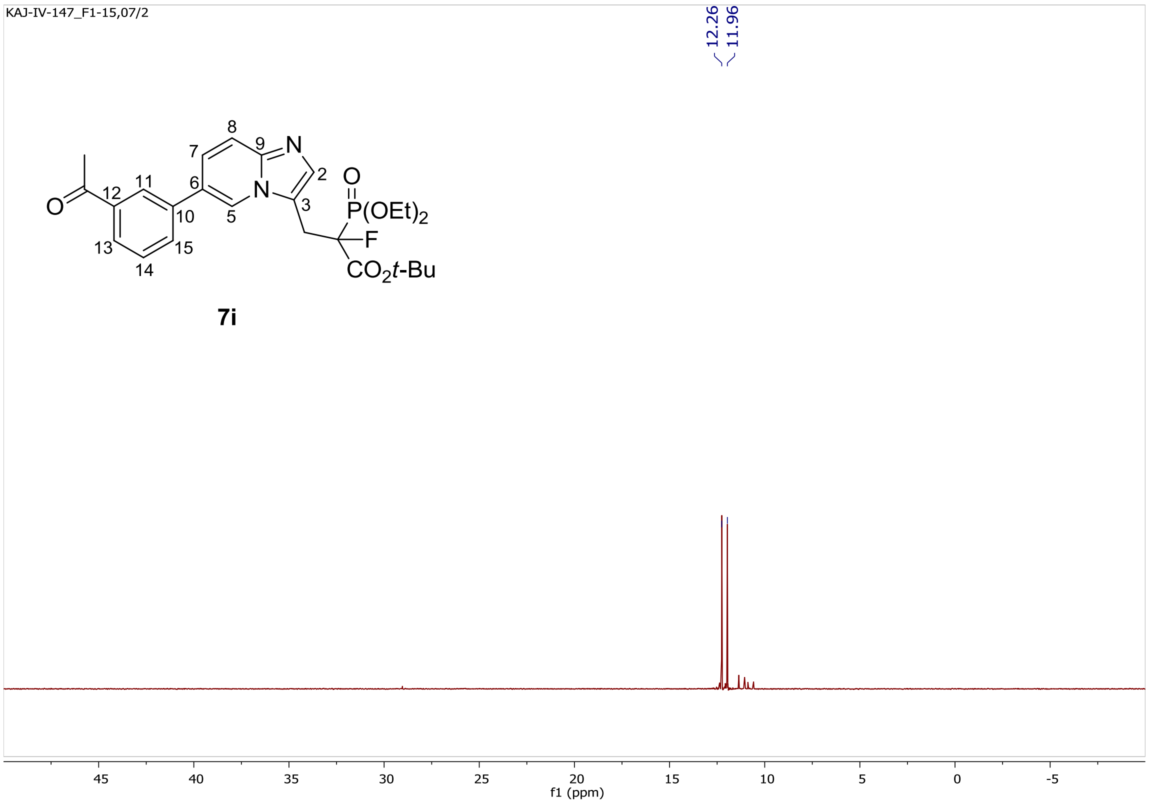

Figure S100. ^{31}P NMR of compound **7i** (283 MHz, $CDCl_3$).

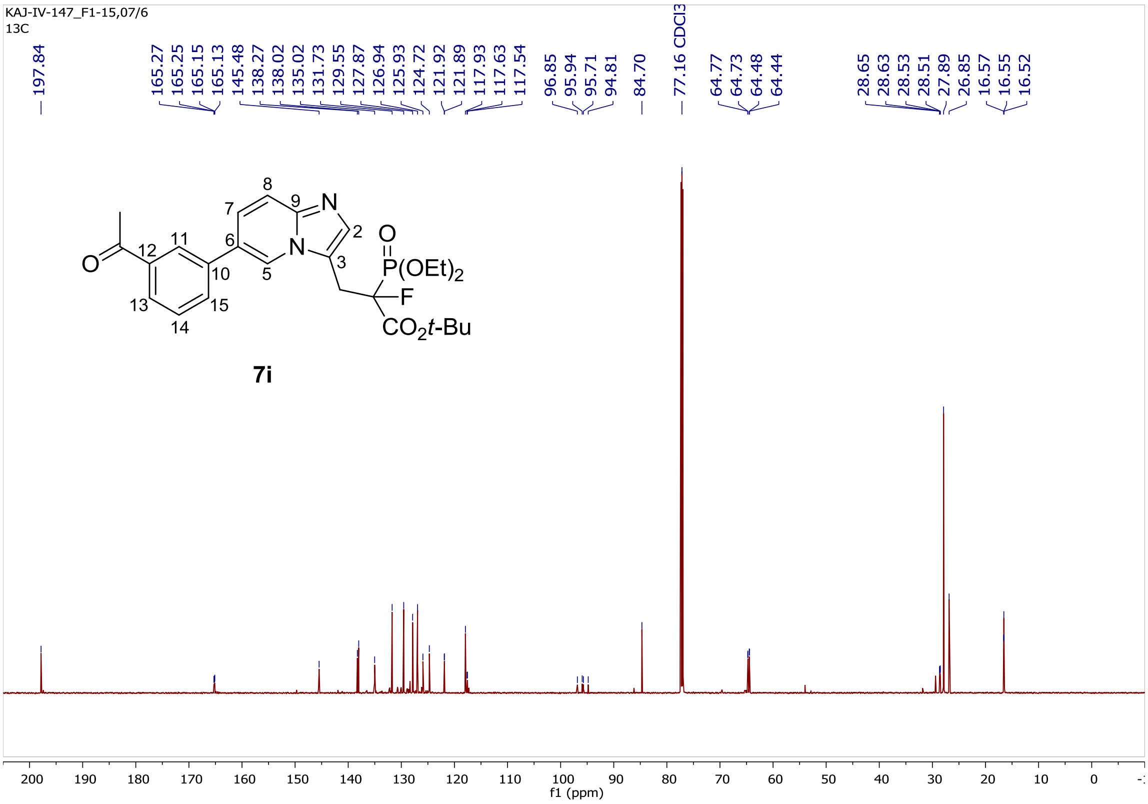

Figure S101. ^{13}C NMR of compound **7i** (176 MHz, $CDCl_3$).

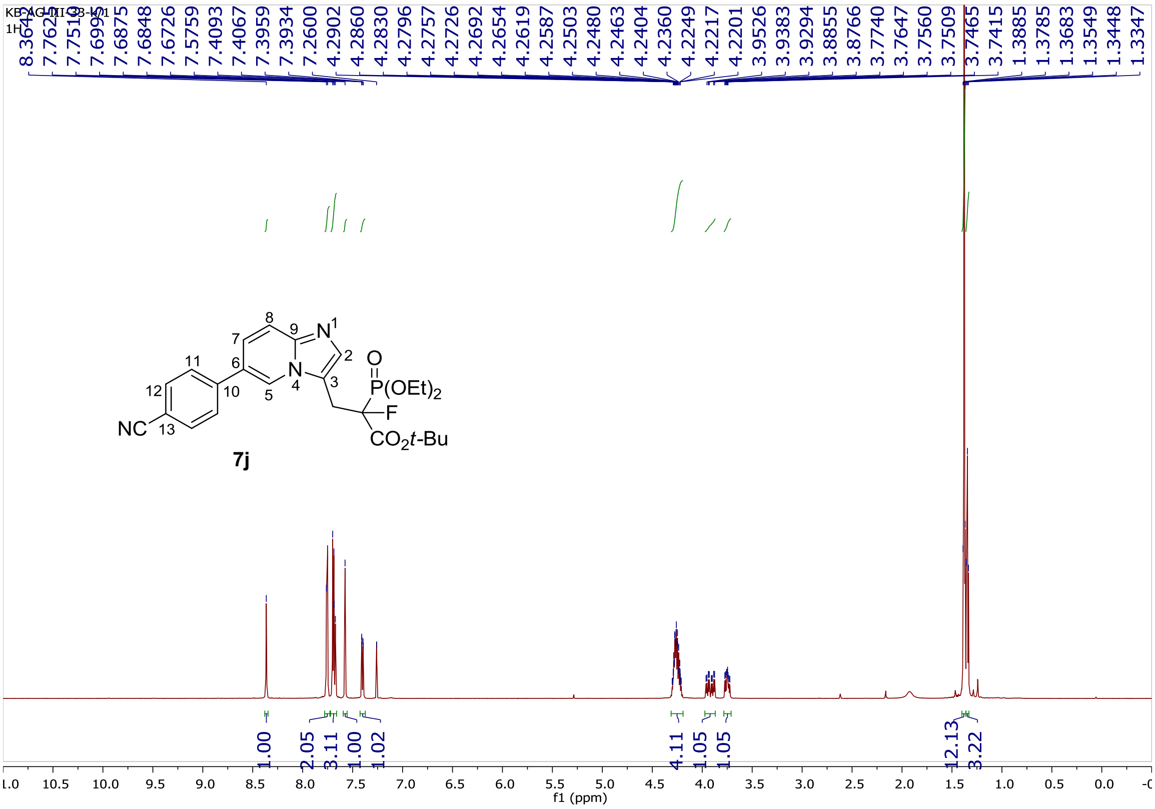

Figure S102. ^{1}H NMR of compound **7j** (700 MHz, $CDCl_3$).

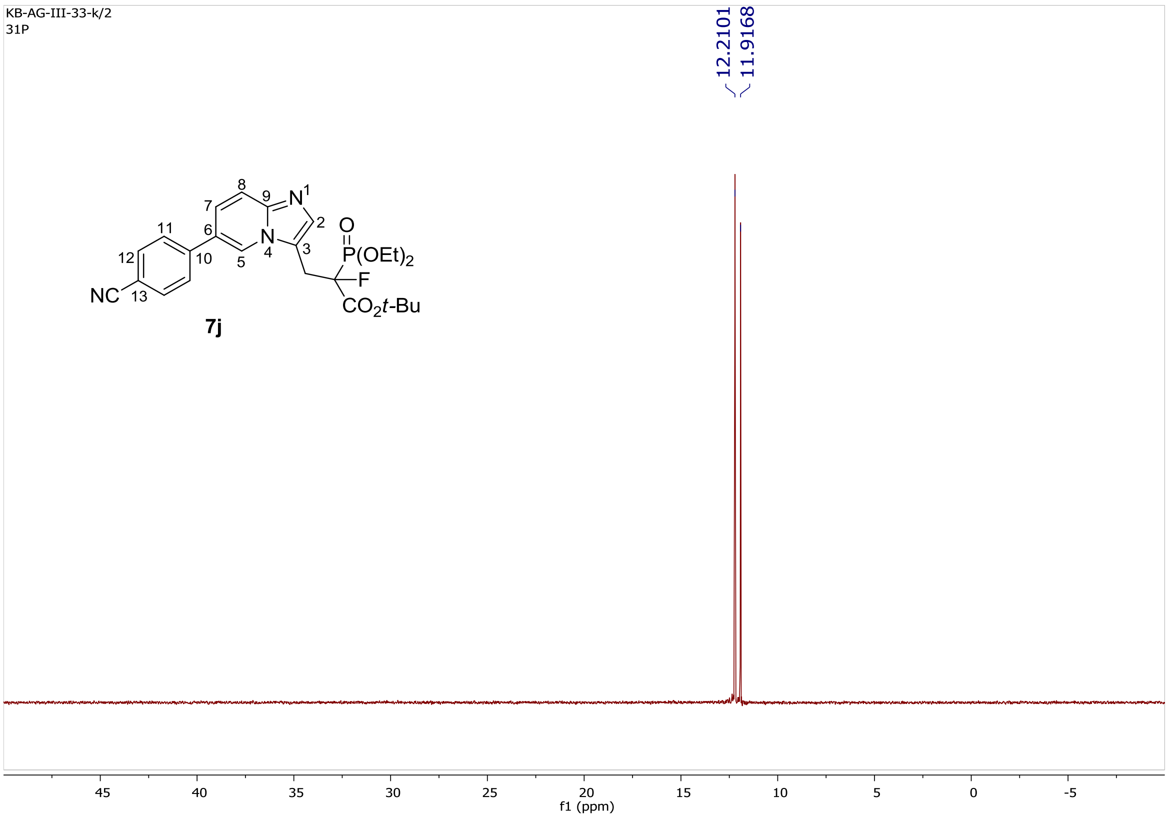

Figure S103. ^{31}P NMR of compound **7j** (283 MHz, $CDCl_3$).

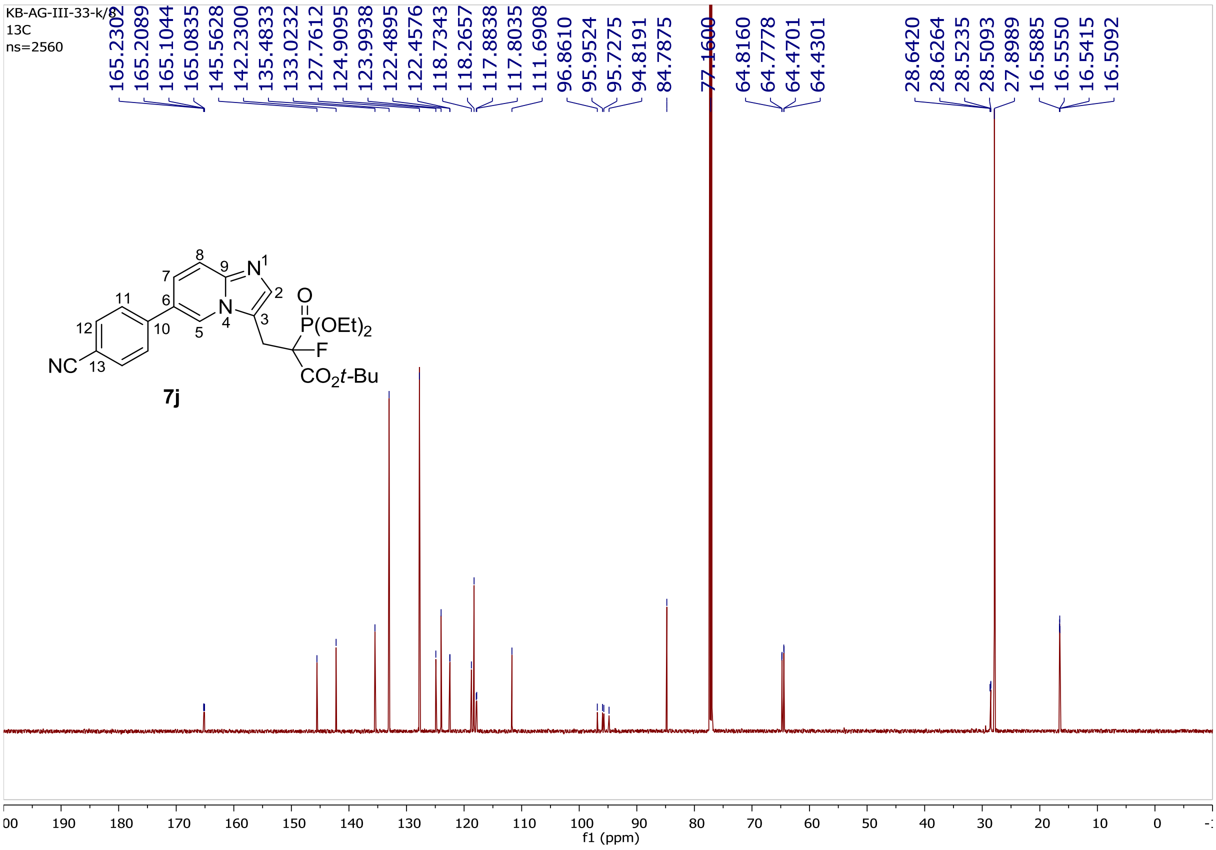

Figure S104. ^{13}C NMR of compound **7j** (176 MHz, $CDCl_3$).

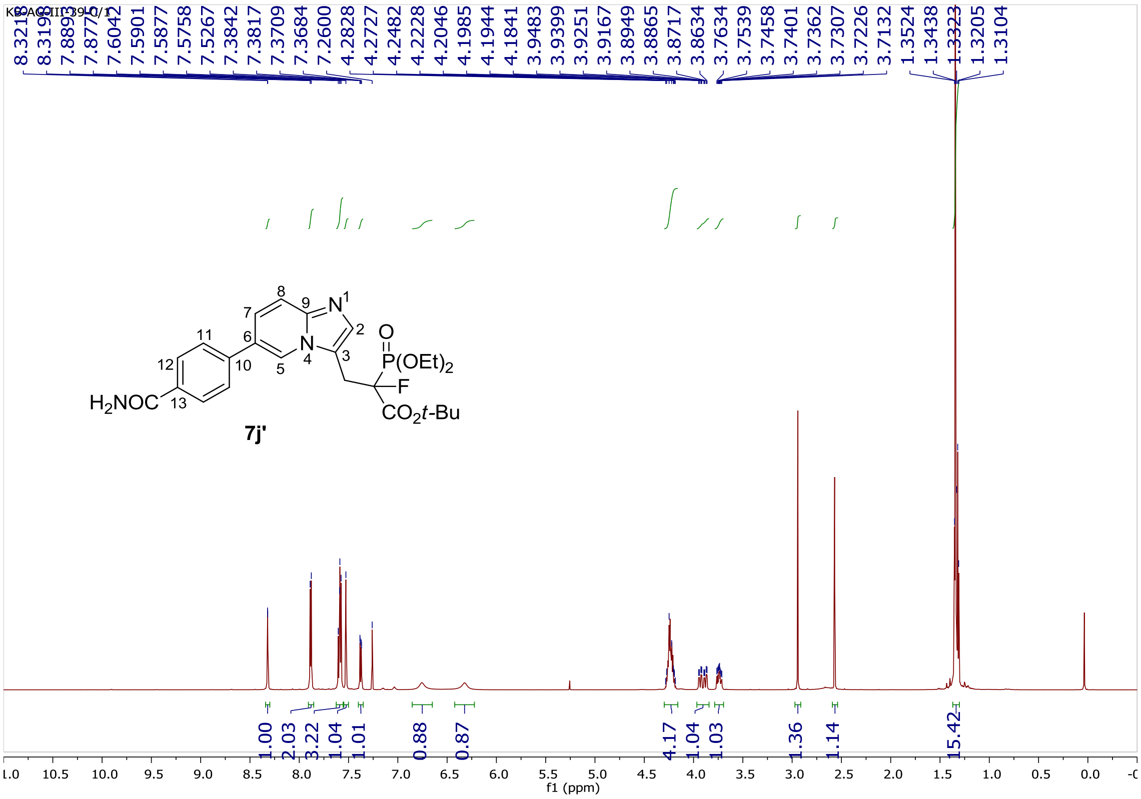

Figure S105. ^{1}H NMR of compound **7j'** (700 MHz, $CDCl_3$).

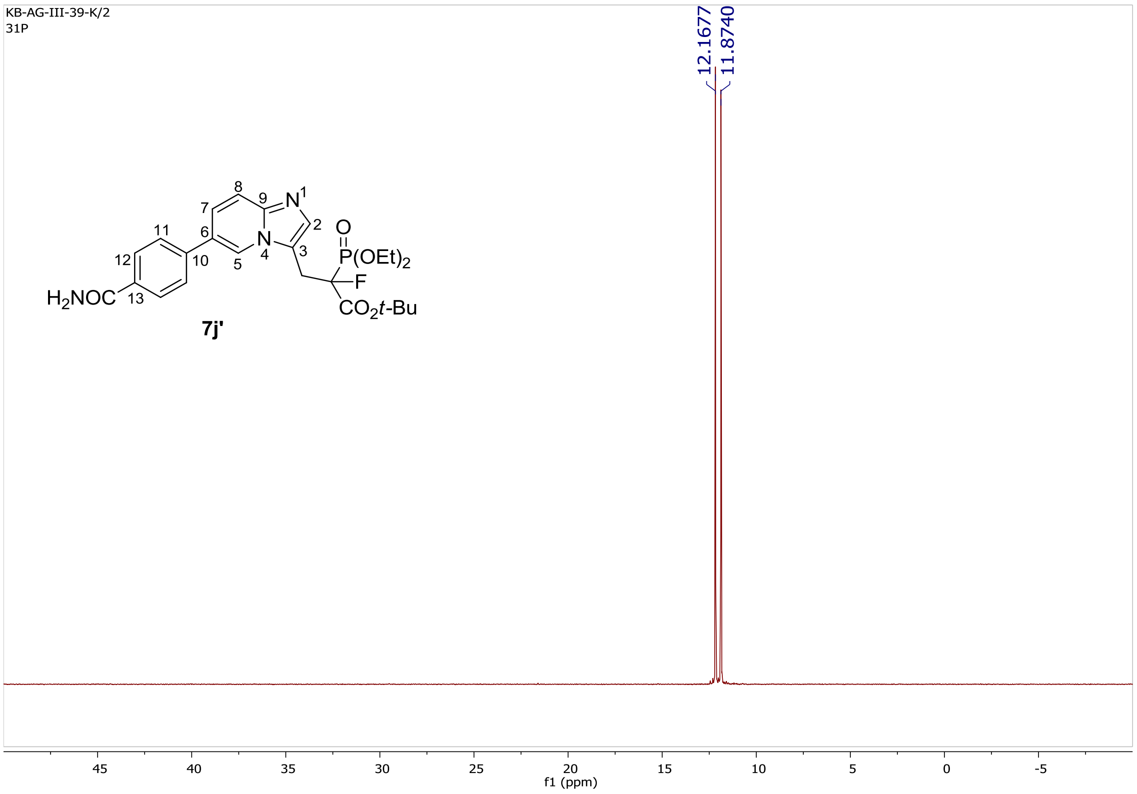

Figure S106. ^{31}P NMR of compound **7j'** (283 MHz, $CDCl_3$).

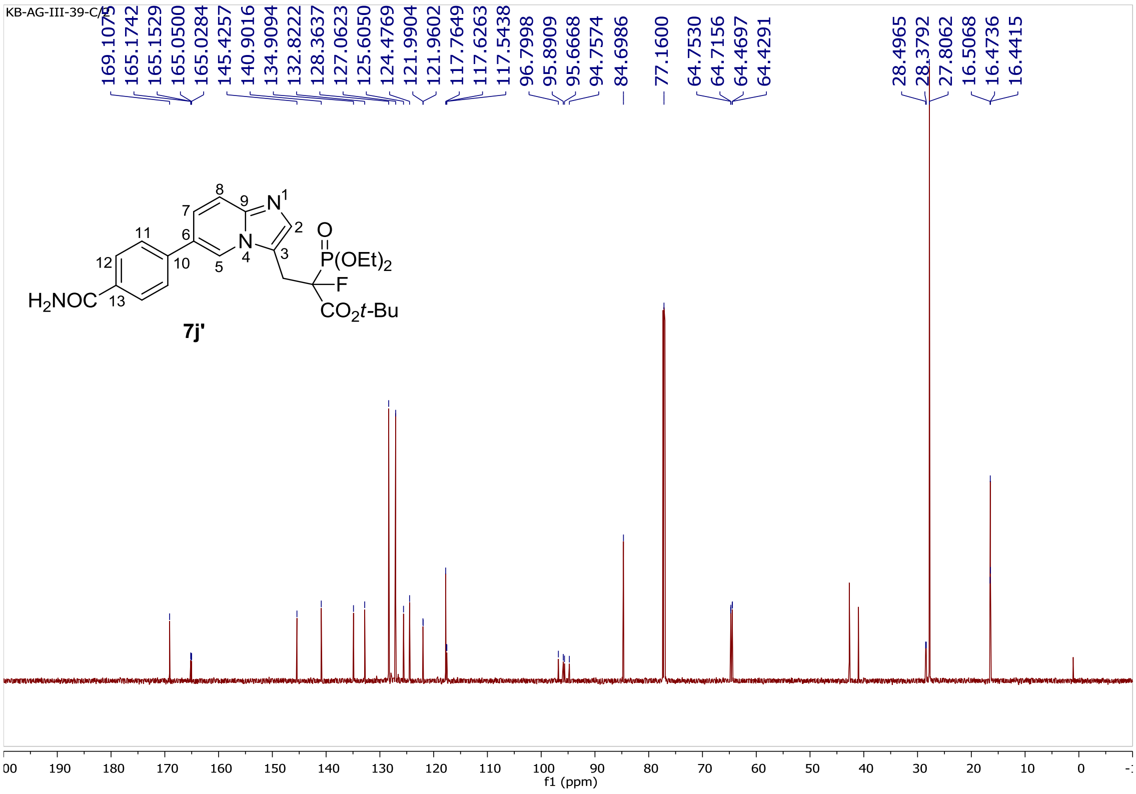

Figure S107. ^{13}C NMR of compound **7j'** (176 MHz, $CDCl_3$).

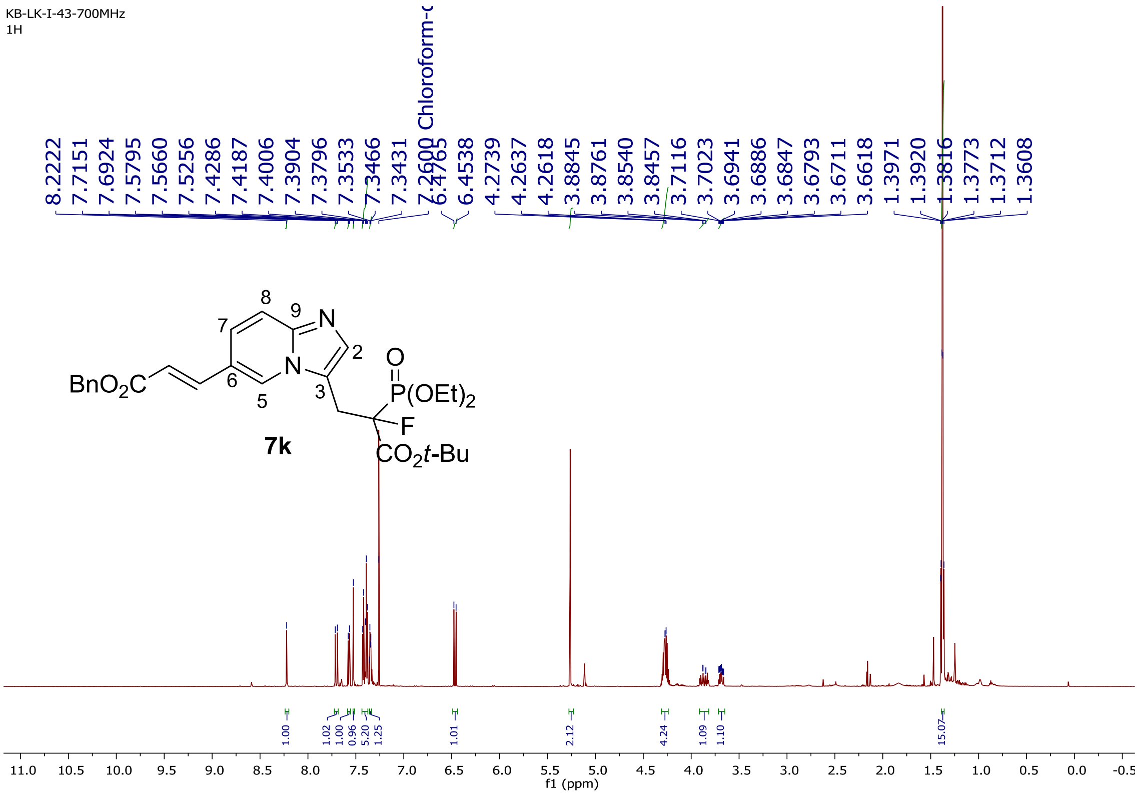

Figure S108. ^{1}H NMR of compound **7k** (700 MHz, $CDCl_3$).

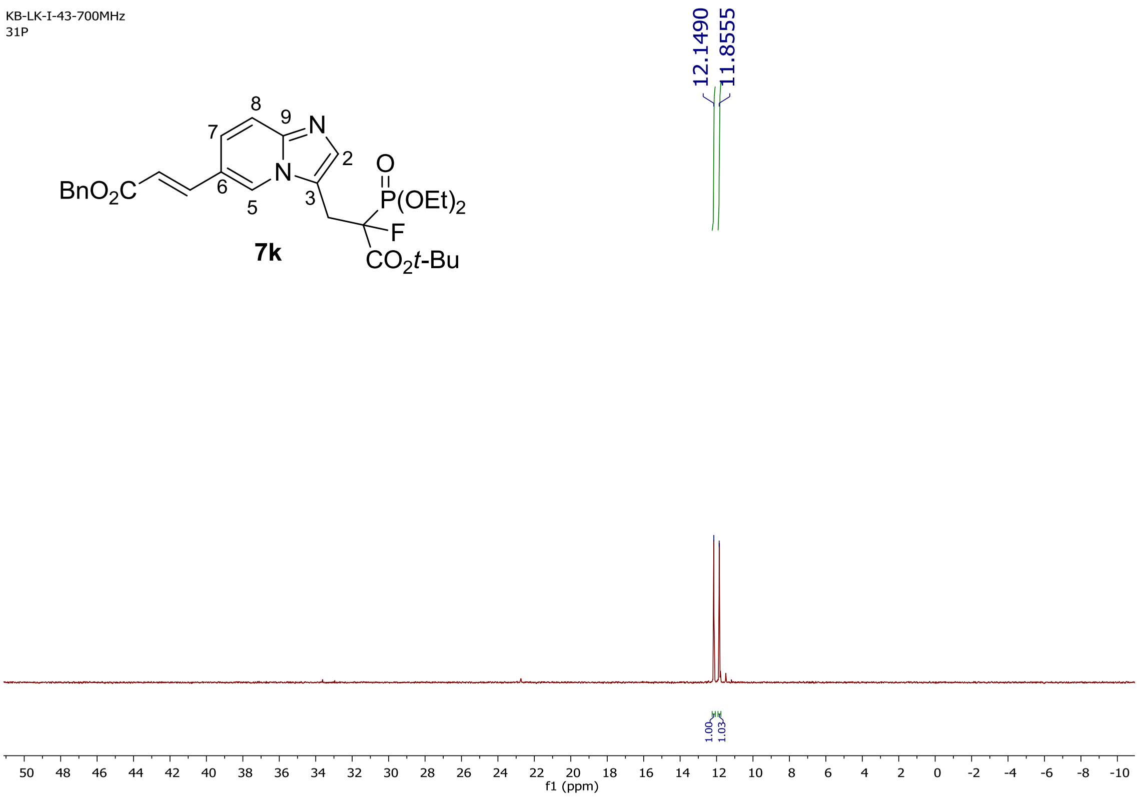

Figure S109. ^{31}P NMR of compound **7k** (283 MHz, $CDCl_3$).

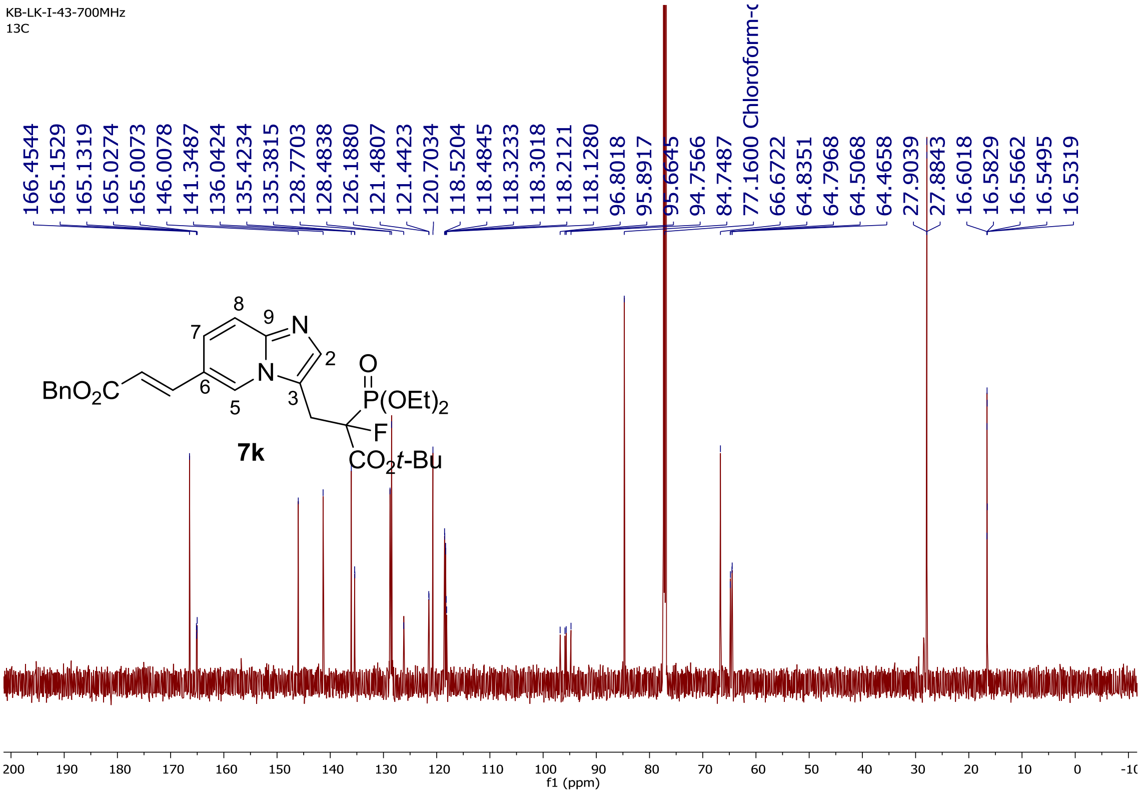

Figure S110. ^{13}C NMR of compound **7k** (176 MHz, $CDCl_3$).

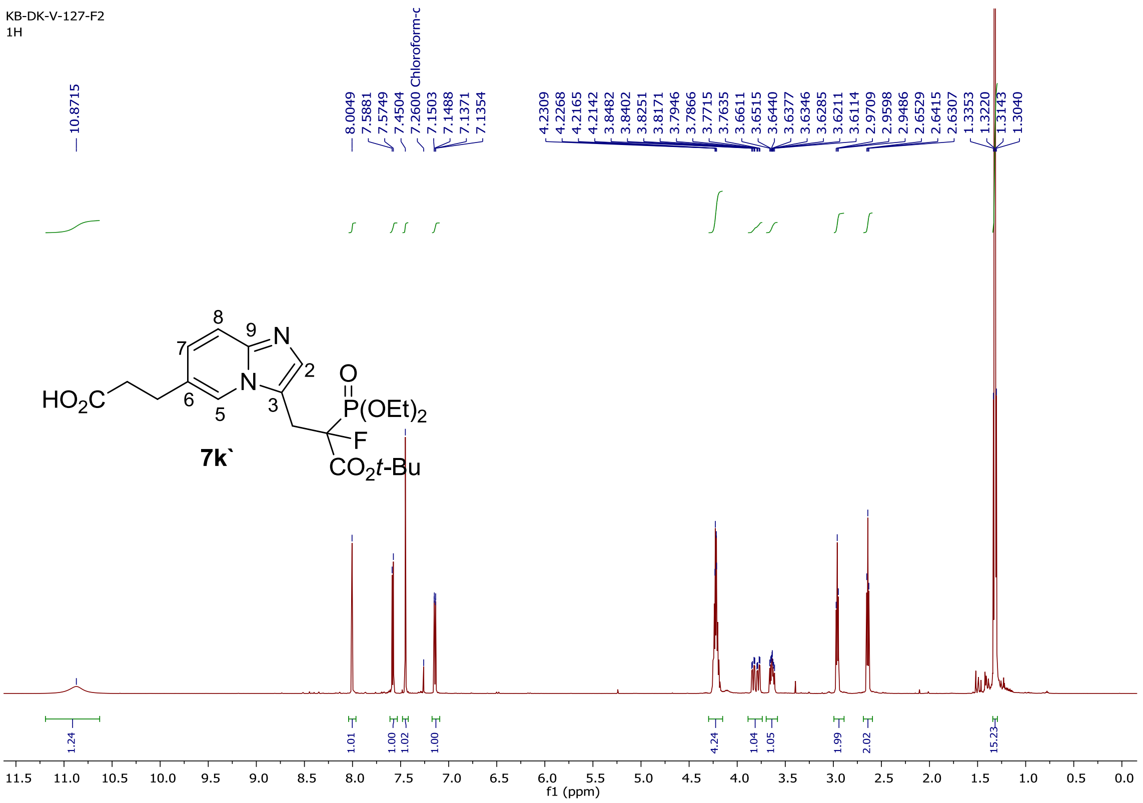

Figure S111. ^{1}H NMR of compound **7k`** (700 MHz, $CDCl_3$).

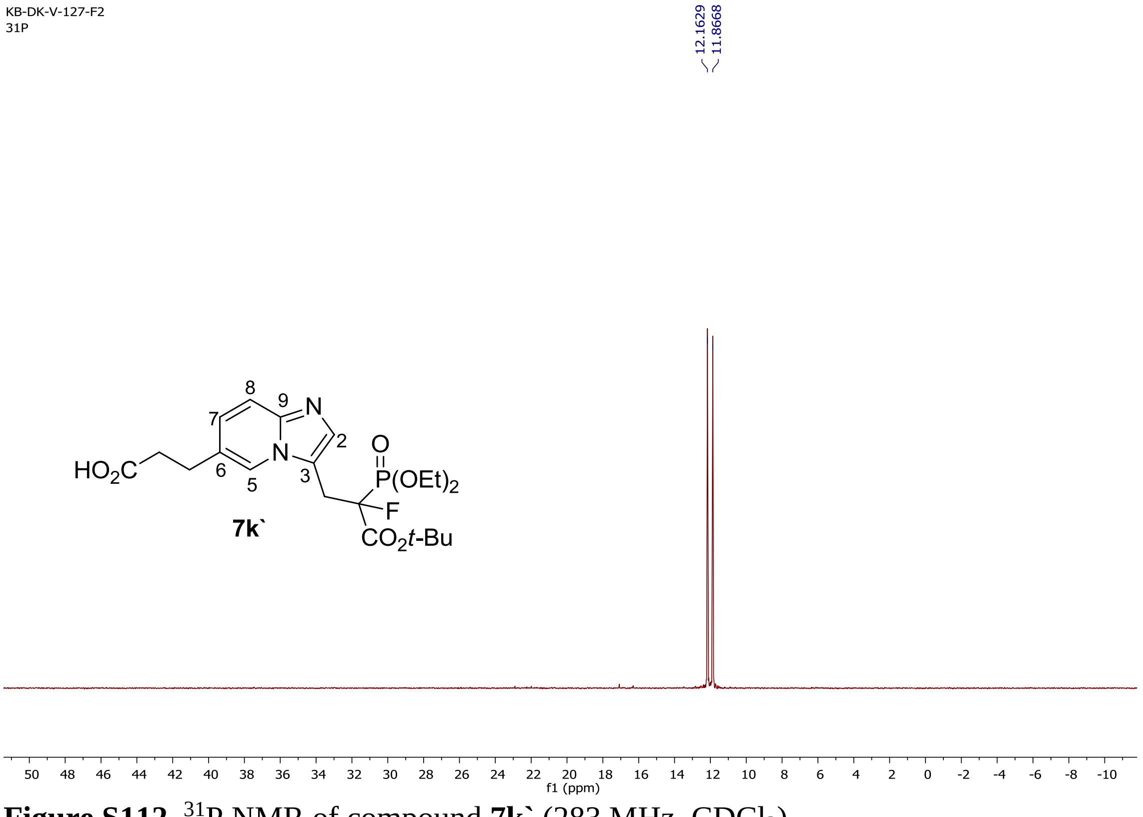

Figure S112. ^{31}P NMR of compound **7k`** (283 MHz, $CDCl_3$).

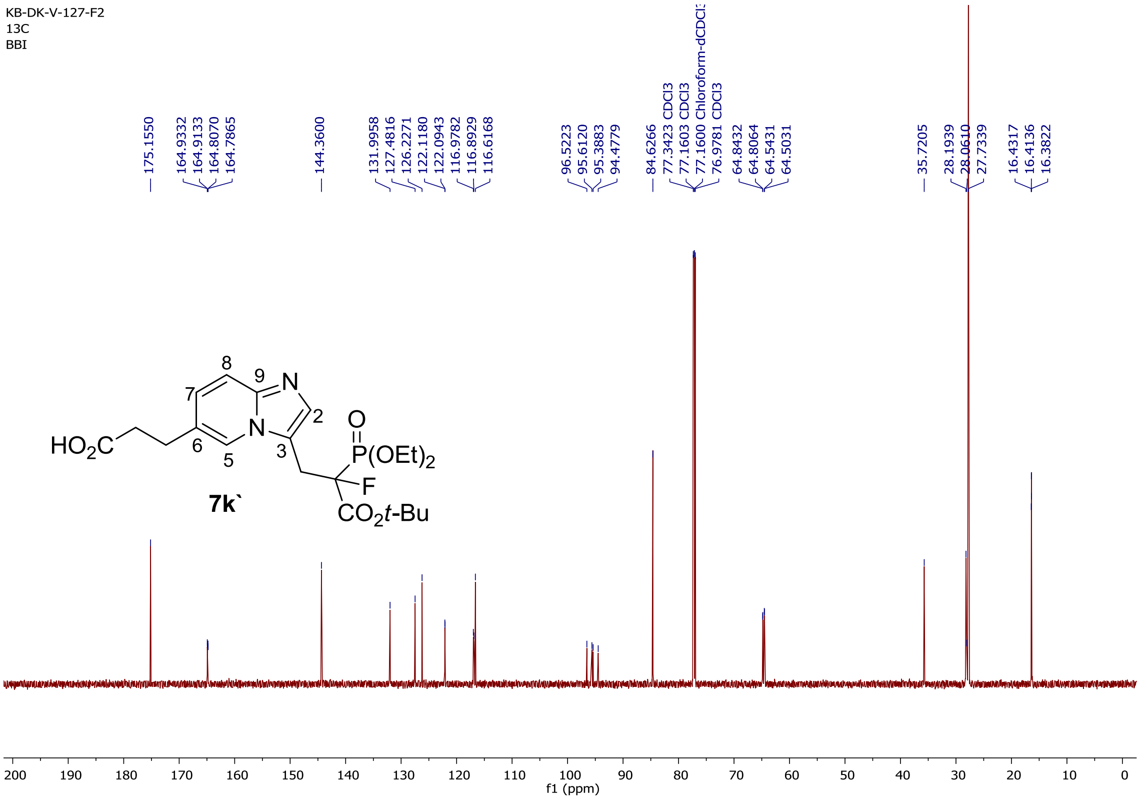

Figure S113. ^{13}C NMR of compound **7k`** (176 MHz, $CDCl_3$).

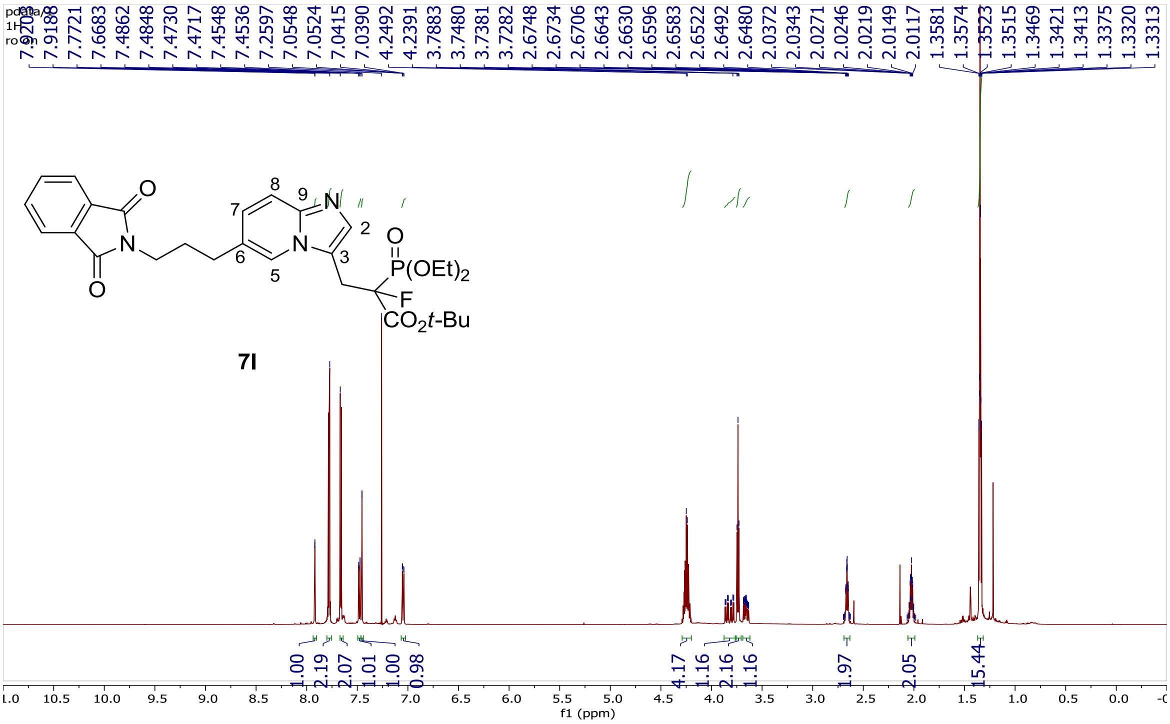

Figure S114. ^{1}H NMR of compound **7l** (700 MHz, $CDCl_3$).

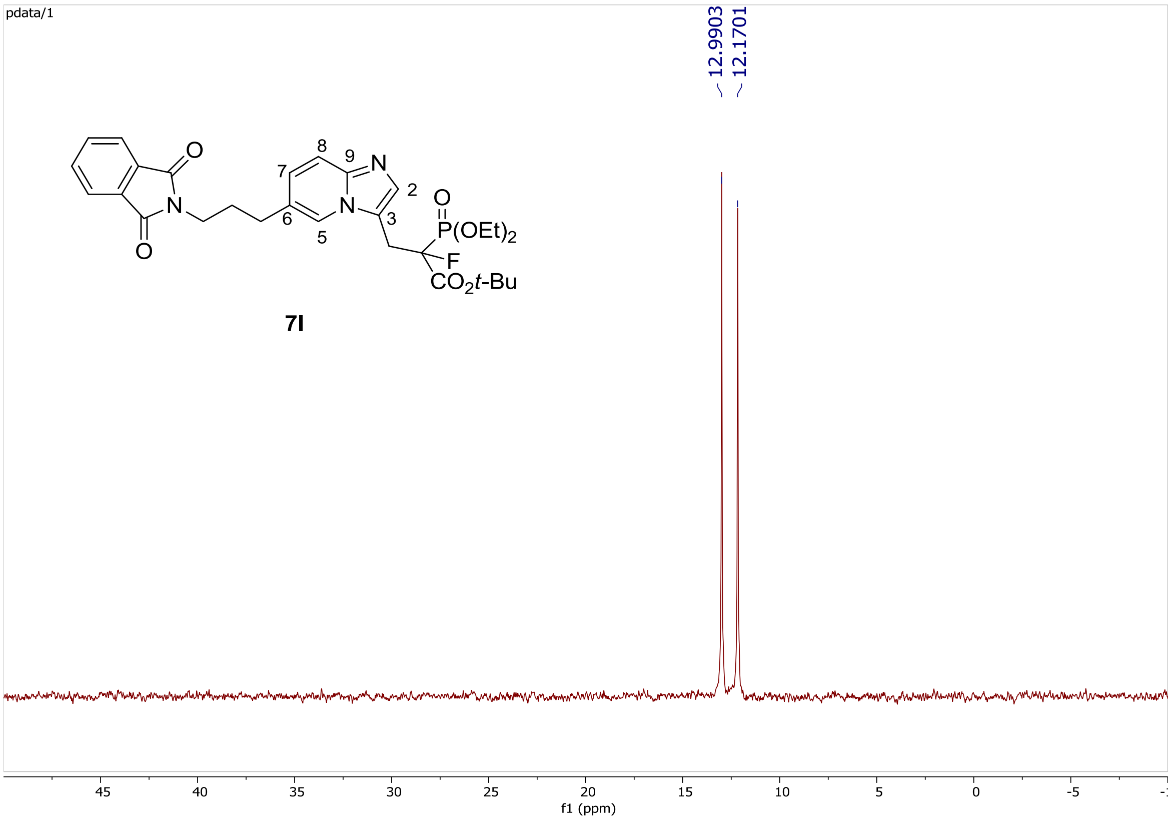

Figure S115. ^{31}P NMR of compound **7l** (283 MHz, $CDCl_3$).

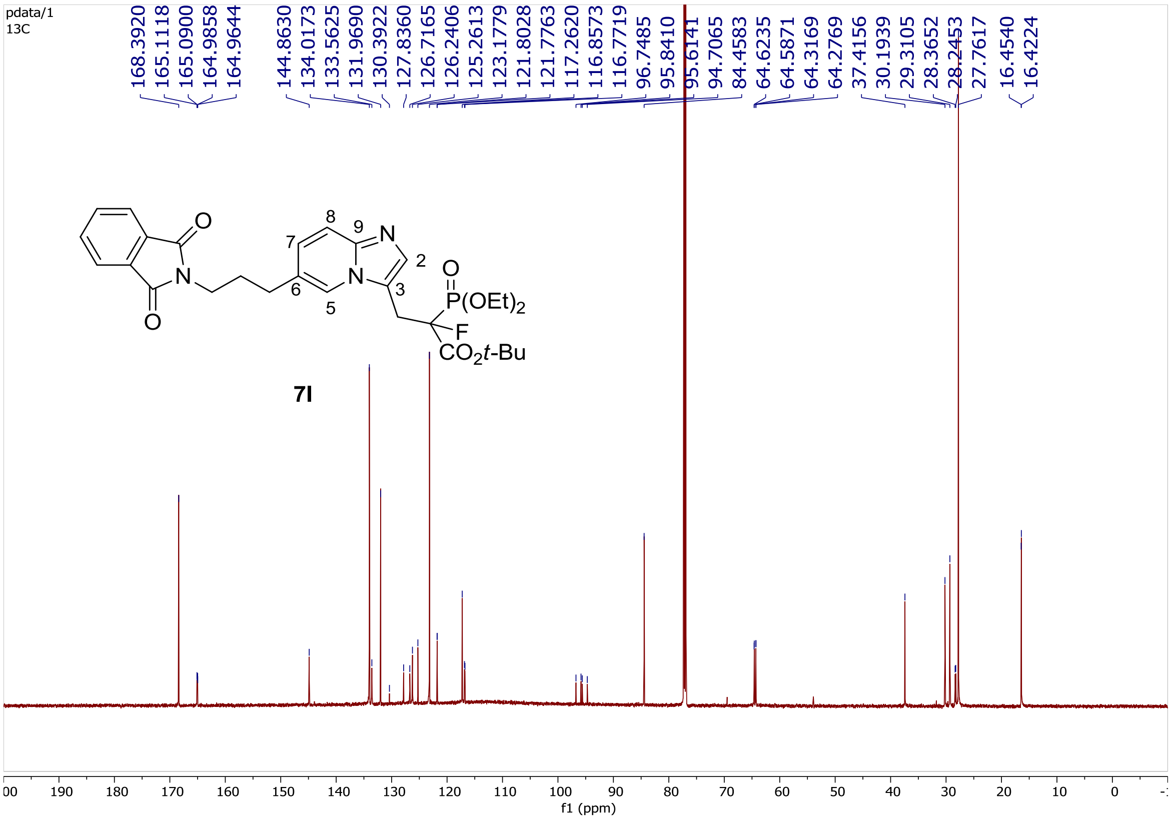

Figure S116. ^{13}C NMR of compound **7l** (176 MHz, $CDCl_3$).
